# *Nyana rooiwalensis*: South Africa's earliest near-complete sauropodomorph dinosaur skull from the Late Triassic of the Eastern Cape Province

**DOI:** 10.1098/rsos.261002

**Published:** 2026-11-11

**Authors:** Atashni Moopen, Jennifer Botha, Roger Benson, Kimberley E. J. Chapelle, Paul M. Barrett, Kathleen Dollman, Jonah N. Choiniere

**Affiliations:** ^1^ Evolutionary Studies Institute, University of the Witwatersrand, Johannesburg, South Africa; ^2^ School of Geosciences, University of the Witwatersrand, Johannesburg, South Africa; ^3^ GENUS: DSTI-NRF Centre of Excellence in Palaeosciences, University of the Witwatersrand, Johannesburg, South Africa; ^4^ Division of Paleontology, American Museum of Natural History, New York City, NY, USA; ^5^ Department of Anatomical Sciences, Stony Brook University, Stony Brook, NY, USA; ^6^ Fossil Reptiles, Amphibians and Birds Section, Natural History Museum, Cromwell Road, London SW7 5BD, UK; ^7^ European Synchrotron Radiation Facility, Grenoble, France

**Keywords:** Sauropodomorpha, lower Elliot Formation, Norian–Rhaetian, cranial anatomy, osteohistology

## Abstract

Triassic sauropodomorph cranial material is known primarily from Argentina, Brazil and Europe and is sparsely distributed across the phylogenetic tree. In southern Africa, only two Late Triassic taxa preserve cranial elements, *Mbiresaurus* and *Kholumolumo*, both represented by limited material. Here we describe *Nyana rooiwalensis*, a new early-branching sauropodomorph from the Upper Triassic lower Elliot Formation of South Africa, represented by the first well-preserved, near-complete skull to be recovered from the unit. The holotype comprises the braincase, palate, the hyoid apparatus and scleral rings, as well as associated postcranial elements. Body mass estimates predict *Nyana* was approximately 400 kg yet osteohistological analyses show at most two lines of arrested growth and a crenulated periosteal surface, indicating an early ontogenetic stage. These observations support the early evolution of rapid growth in Sauropodomorpha, foreshadowing the evolution of sauropod gigantism. Phylogenetic analyses place *Nyana* as the sister taxon to either the North American *Sarahsaurus* or the South African *Ngwevu* and is recovered as either an early-branching massopodan or nested within Massospondylidae. The exceptional preservation, early ontogenetic stage and phylogenetic position of *Nyana* provide critical insights into cranial evolution, postural and locomotory behaviours, body mass and growth dynamics during the early diversification of Sauropodomorpha.

## 1. Introduction

The Elliot Formation (EF) of South Africa records a 30-million-year interval spanning the Upper Triassic (Norian; approx. 220 Ma) to the Lower Jurassic (Hettangian–Sinemurian; approx. 192 Ma) that encompasses the end-Triassic mass extinction (ETE) event [1]. Nearly 200 years of palaeontological exploration have yielded evidence for a series of diverse faunal assemblages, including non-avian dinosaurs, crocodylomorphs, chelonians, temnospondyls, cynodont therapsids, including mammaliamorphs, and diverse floral remains [2–8]. Recent reviews have provided an updated geochronological and biostratigraphic framework for the EF and underscored its importance by documenting the abundance of various tetrapod groups [1,9,10]. These include reviews of the diversity of other archosaur groups such as aetosaurs and rauisuchians, including ichnotaxa [11–14]; assessments of early ornithischian evolution using occurrences of EF taxa [15,16]; and summaries of pseudosuchian diversity, distribution and abundance [17,18]. The EF is subdivided into two formal biostratigraphic zones—the *Scalenodontoides* Assemblage Zone and overlying *Massospondylus* Assemblage Zone—which correspond with two informal lithostratigraphic units, the lower and upper EF (lEF, uEF), respectively [1,9,10,19]. The different faunas recorded in the Upper Triassic and Lower Jurassic EF deposits provide critical evidence for changing ecosystems across the ETE.

Sauropodomorph dinosaurs are by far the most abundant vertebrate fossils in the EF, ranging from isolated bones to articulated skeletons, although postcranial elements dominate these assemblages [5,9,10]. Cranial material is scarce, and most EF cranial material (figure 1) derives from the Lower Jurassic uEF, including complete skulls of *Ngwevu intloko* [20] and *Massospondylus carinatus* [21–23] and incomplete skulls of *Massospondylus kaalae* [24], *Arcusaurus pereirabdalorum* [25] and *Aardonyx celestae* [26]. The much more limited material from the Upper Triassic lEF includes a pair of postorbitals referred to as *Kholumolumo ellenbergerorum* [27] from Lesotho. A near-complete sauropodomorph skeleton with a skull (NMQR 3314) thought to have come from the lEF and originally referred to as *Melanorosaurus readi* has since been shown to pertain to the lower part of the uEF [5,9] and removed from *Melanorosaurus* [28]. The only other Late Triassic sauropodomorph cranial material from southern Africa pertains to *Mbiresaurus raathi* [29] from the Pebbly Arkose Formation of Zimbabwe, which co-occurs with rhynchosaurs and cynodonts that support a Carnian age, predating the lowest strata of the EF. *Mbiresaurus* represents the oldest sauropodomorph cranial material from southern Africa and includes frontals, postorbitals, quadrate, maxilla, premaxilla, articular, surangular, dentary and teeth [29].

**Figure 1.**
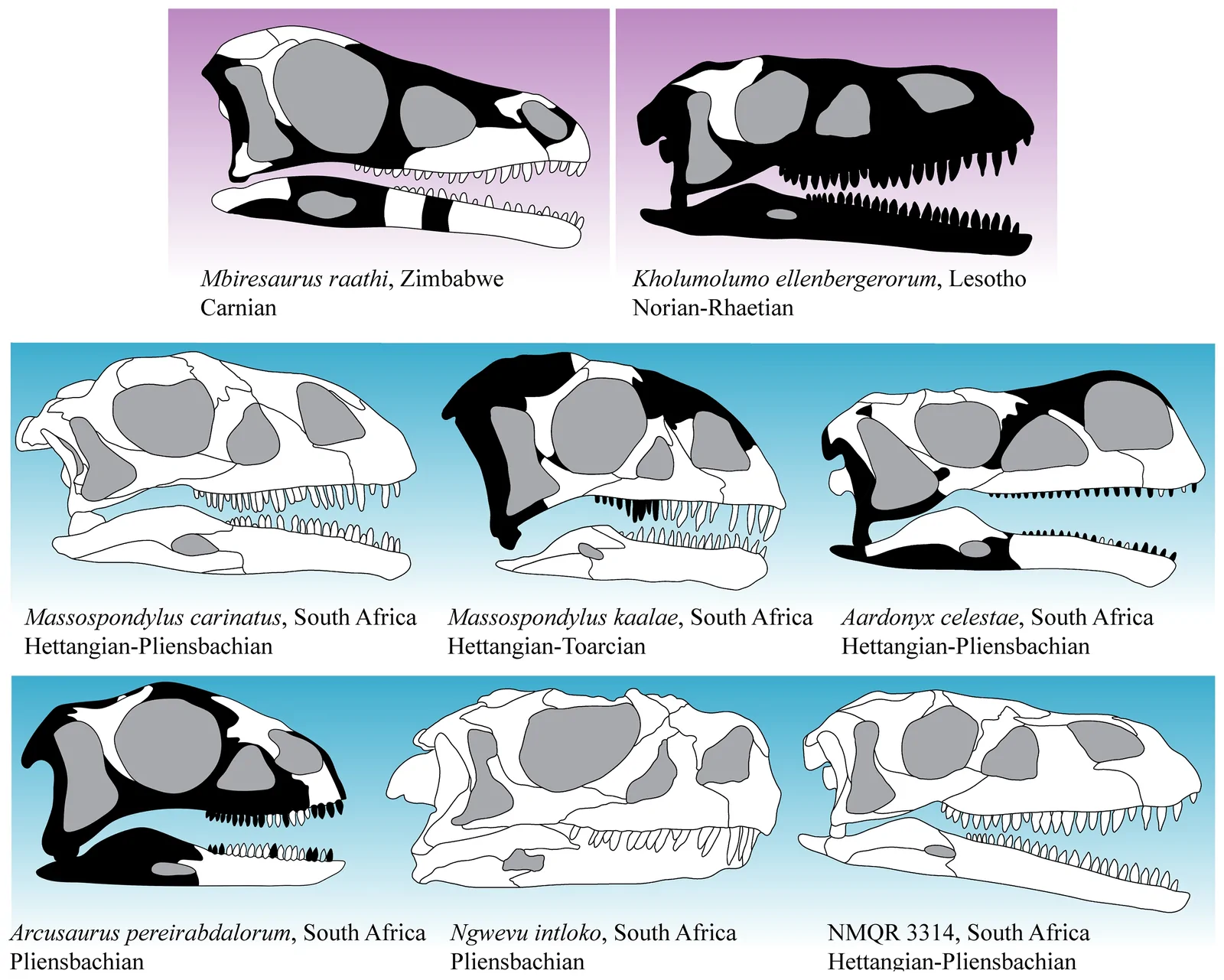
Early-branching sauropodomorph skulls from southern Africa. Preserved elements are indicated in white. Background colours represent the Upper Triassic (purple) and Lower Jurassic (blue). Images are displayed at original size and have not been scaled. See S4 in the supplemental material for references on which the skeletal drawings are based.

Late Triassic sauropodomorph cranial material is relatively scarce worldwide, with relatively complete skulls (at least approx. 50%) referred to some of the earliest-branching taxa, namely, *Buriolestes schultzi* (Carnian, Brazil [30]); *Eoraptor lunensis* (Carnian, Argentina [31]); *Panphagia protos* (Carnian, Argentina [32]); *Bagualosaurus agudoensis* (Carnian, Brazil [33]); *Pampadromaeus barberenai* (Carnian, Brazil [34]); *Saturnalia tupiniquim* (Carnian, Brazil [35]); *Pantydraco caducus* (Rhaetian, Wales [36]); and *Efraasia minor* (Norian, Germany [37]). Other examples occur in the Norian of South America and Europe, such as *Unaysaurus tolentinoi* (Norian, Brazil [38]); *Macrocollum itaquii* (Norian, Brazil [39]); *Plateosaurus trossingensis* (Norian–Rhaetian, Germany [40]); *Riojasaurus incertus* (Norian, Argentina [41]); *Coloradisaurus brevis* (Norian, Argentina [42]); *Issi saaneq* (Norian, Greenland [43]) and a recent unnamed massopodan (Norian, Switzerland [44]). This distribution emphasizes the disparate skull preservation among these taxa and the need for well-preserved cranial material from other early-branching sauropodomorphs (EBSMs), which is critical for clarifying patterns of cranial evolution and ontogenetic changes during the early evolution of sauropodomorphs.

Here, we present a new sauropodomorph from South Africa represented by a skull of a juvenile missing its rostrum, with associated axial and appendicular material. The fossil material was recovered from the Norian–Rhaetian strata of the lEF. This new specimen adds to the species richness of lEF faunal assemblages, helps to document the cranial morphology of EBSMs and is particularly important in improving our knowledge of the cranial anatomy of juvenile sauropodomorphs during the Late Triassic.

## 2. Material and methods

### 2.1. Description and comparative anatomy

We use non-standardized Romerian anatomical and directional terms (e.g. anterior/posterior rather than rostral/caudal). Vertebral laminae and fossae terminology follows Wilson [45] and Wilson *et al.* [46]. Table 1 lists the taxa and bibliographic references used for anatomical comparisons.

**Table 1.** List of comparative taxa and their sources and original publications.

taxa	specimen number	source	original publication
*Adeopapposaurus mognai*	PVSJ 610	Martínez [47]	Martínez [47]
*Anchisaurus polyzelus*	YPM 1883	Yates [48]	Hitchcock [49]
*Antetonitrus ingenipes*	BP/1/4952	McPhee *et al.* [50]	Yates & Kitching [51]
*Coloradisaurus brevis*	PVL 3967	Apaldetti *et al.* [42]	Bonaparte [52]
*Efraasia minor*	SMNS 13667	Bronzati & Rauhut [37]	Huene [53]
*Issi saaneq*	NHMD 164741, 164758	Beccari *et al.* [43]	Beccari *et al.* [43]
*Jingshanosaurus xinwaensis*	LFGT-ZLJ0113	Zhang *et al.* [54], Zhang & Yang [55]	Zhang & Yang [55]
*Kholumolumo ellenbergerorum*	MNHN.F.LES397	Peyre de Fabrègues & Allain [27]	Peyre de Fabrègues & Allain [27]
*Ledumahadi mafube*	BP/1/7120	McPhee *et al.* [56]	McPhee *et al.* [56]
*Leyesaurus marayensis*	PVSJ 706	Apaldetti *et al.* [57]	Apaldetti *et al.* [57]
*Lufengosaurus huenei*	IVPP V15	Barrett *et al.* [58]	Young [59]
*Macrocollum itaquii*	CAPPA/UFSM 0001	Müller [60]	Müller *et al.* [39]
*Massospondylus carinatus*	BP/1/5241	Chapelle & Choiniere [21], Barrett *et al.* [61]	Owen [62]
*Melanorosaurus readi*	SAM-PK-3449	Barrett & Choiniere [28]	Haughton [2]
*Ngwevu intloko*	BP/1/4779	Chapelle *et al.* [20]	Chapelle *et al.* [20]
NMQR 3314	NMQR 3314	Yates [63]	Galton *et al.* [64]
*Plateosauravus cullingworthi*	SAM-PK-3351	Haughton [2]	Haughton [2]
*Plateosaurus erlenbergiensis*	AMNH FARB	Prieto-Márquez & Norell [65]	Huene [66]
*Riojasaurus incertus*	ULR 56	Bonaparte & Pumares [41]	Bonaparte [67]
*Sarahsaurus aurifontanalis*	TMM 43646-2; MCZ 8893	Marsh & Rowe [68], Rowe *et al.* [69]	Rowe *et al.* [69]
*Unaysaurus tolentinoi*	UFSM 11069	McPhee *et al.* [38]	Leal *et al.* [70]
*Yunnanosaurus huangi*	NGMJ 004546	Barrett *et al.* [71], Young [72]	Young [72]
unnamed taxon	SMF 13.5.37	Lania *et al.* [44]	Lania *et al.* [44]

We used synchrotron radiation propagation-based phase contrast micro-computed tomography to examine the skull of *Nyana rooiwalensis*. Scanning was conducted at the European Synchrotron Radiation Facility (ESRF) in Grenoble, France, with private discretionary beam time on beamline BM18. The fossil was imaged with an isotropic voxel size of 20 µm. The synchrotron operated at a current of 200 mA, using a filtered white beam set-up with a 2000 µm thick LuAG scintillator coated with an aluminium reflective layer. The sample-to-detector distance was 20 m [73], and imaging was performed at an energy of 155 keV with molybdenum (1.3 mm) and tungsten (0.3 mm) attenuator filters.

Data acquisition was carried out using a PCO Edge 4.2 sCMOS camera (PCO, Kelheim, Germany), capturing 10 000 projections. To enhance the field of view, the centre of rotation was offset twice [74]. At a voxel size of 20 µm, the total exposure time per projection was 30 ms, consisting of three subframes of 10 ms each [75]. The final tomographic volume was reconstructed using PyHST2 [76] and processed with the single-distance Paganin phase retrieval method [77]. The specimen was then reconstructed in VGSTUDIO MAX v.3.2 (Volume Graphics, Heidelberg, Germany), and measurements of the cranial elements were taken from these digital images. Images of the postcranial elements were taken with a Canon EOS 800D, and measurements were taken manually with callipers.

### 2.2. Osteohistological analyses

Transverse sections from the diaphysis of the left ulna and tibia of BP/1/8468 were used for osteohistological analysis. The elements were photographed, drawn and measured prior to thin sectioning. The segments were taken from the proximal portion of the ulnar shaft and the distal portion of the tibial shaft as mid-shafts were not preserved. Thin sectioning methods follow those of Botha-Brink *et al.* [78]. All the osteohistological sections were produced at the Evolutionary Studies Institute, University of the Witwatersrand, Johannesburg, South Africa. This study was conducted under the Eastern Cape Provincial Heritage Resources Authority permit 1/1/APM-BG permit/21/08/001.

The sections were rendered under normal, polarised and cross-polarised light with a lambda compensator using a Nikon ECLIPSE Ci-POL polarizing microscope and DS-Fi3 digital camera controlled with NIS-Elements D 4.6 (Nikon Corp., Tokyo, Japan). Comparative osteohistological terminology follows that of Prondvai *et al.* [79] and de Buffrénil *et al.* [80].

### 2.3. Phylogenetic analyses

We assessed the phylogenetic relationships of *N. rooiwalensis* using the morphological character matrix from Barrett *et al.* [81] with the final version containing 418 characters and 83 taxa and maintained their charactering orderings (see supplementary material, S1).

We conducted parsimony analyses in TNT (Tree Analysis UsingNew Technology; v.1.5 [82]) using an initial heuristic search strategy of 10 000 replicates of Wagner trees, branch-swapping on these trees using tree-bisection reconnection (TBR), using a random seed of 1 and saving one tree per replication. An additional round of TBR branch-swapping was performed on the most parsimonious trees from the first search (stopping when max trees hit). We calculated Bremer support values [83] in TNT, saving 10 000 suboptimal trees of up to five extra steps, and obtained absolute bootstrap support values with 10 000 replicates. We used the iterPCR function in TNT to identify taxa for *a posteriori* removal. Consistency and retention indices were calculated within TNT. We then generated a reduced majority-rule consensus tree and imported it into RStudio (v.4.2.0) to generate a time-calibrated phylogeny using the strap [84] and geiger [85] packages. We also generated a strict consensus tree (see supplementary material, S1).

## 3. Institutional abbreviations

**AMNH**, American Museum of Natural History, New York, USA; **BP**, Evolutionary Studies Institute, Johannesburg, South Africa (formerly Bernard Price Institute); **IVPP**, Institute of Vertebrate Paleontology and Paleoanthropology, Beijing, China; **LFGT**, Bureau of Land and Resources of Lufeng County, Lufeng, China; **MCZ**, Museum of Comparative Zoology, Harvard University, Cambridge, Massachusetts, USA; **MNHN**, Muséum National d'Histoire Naturelle, Paris, France; **NGMJ**, Nanjing Geological Museum, Nanjing, China; **NMQR**, National Museum, Bloemfontein, South Africa; **PVL**, Paleontología de Vertebrados, Instituto ‘Miguel Lillo’, San Miguel de Tucumán, Argentina; **PVSJ**, Instituto y Museo de Ciencias Naturales, Universidad Nacional de San Juan, San Juan, Argentina; **SAM-PK**, Iziko South African Museum, Cape Town, South Africa; **SMNS**, Staatliches Museum für Naturkunde, Stuttgart, Germany; **TMM**, Vertebrate Palaeontology, Laboratory, University of Texas at Austin, Austin, Texas, USA; **ULR**, Museo de Ciencias Naturales, Universidad Nacional de La Rioja, La Rioja, Argentina; **YPM**, Peabody Museum of Natural History, Yale University, New Haven, USA.

## 4. Systematic palaeontology

**Dinosauria** Owen, 1842 [86]

**Saurischia** Seeley, 1888 [87]

**Sauropodomorpha** von Huene, 1932 [88]

***Nyana rooiwalensis*** gen. et. sp. nov.

Figures 3–59

**Holotype.** BP/1/8468 consists of the partial skeleton of a single individual, including a partial articulated skull with associated but disarticulated left and right maxillary fragments; the posterior half of the right mandible; both proatlases; four cervical vertebrae; two dorsal vertebrae; one dorsal neural arch; a sacral vertebral centrum; two caudal vertebral centra and fragments from the vertebral column; proximal and distal ends of the left ulna; a manual phalanx and ungual; the proximal and distal ends of a right femur; the distal end of a left tibia; and a pedal ungual.

**Type locality and horizon.** BP/1/8468 was collected in 2018 from the outskirts of Qhemegha Village, Sterkspruit District, ±2 km north of the village of Gcina, Rooiwal Tribal Area, Eastern Cape Province, South Africa (figure 2). The specimen was found in a mottled purple/red silty fine-grained sandstone with a nodular preservation that lies within the *Scalenodontoides* Assemblage Zone of the lower EF (lEF). BP/1/8468 was collected from the upper half of the lEF approximately 140 m above the Molteno Formation–EF contact. Correlations with radiometrically dated tuffaceous layers in the region [1], such as those from Quthing and Maphutseng, suggest that it is approximately Norian–Rhaetian in age.

**Figure 2.**
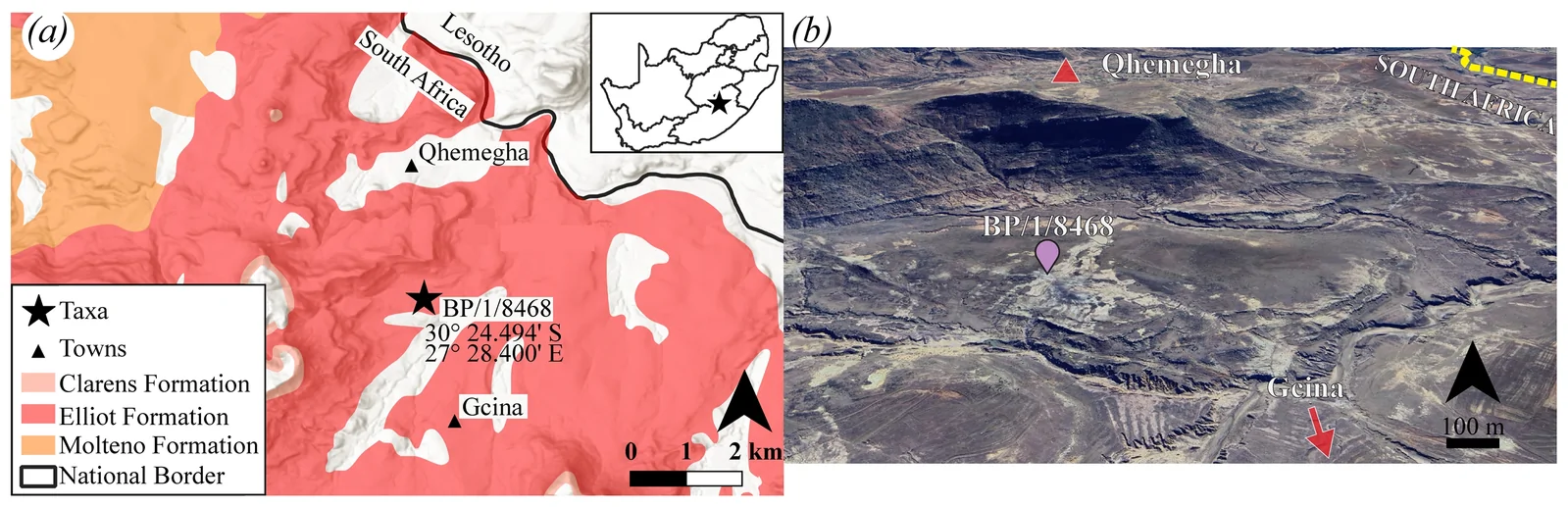
(*a*) Lithostratigraphy of the Qhemegha area. (*b*) Landscape view of the local geology at the site. Large black arrowheads indicate North.

**Diagnosis.** *Nyana rooiwalensis* differs from all other sauropodomorphs on the basis of a unique character combination and three autapomorphies (the latter indicated by ‘*’): at least 23 alveoli in the maxilla; the ascending ramus of the maxilla extends at a 40° angle from its long axis*; the postorbital is mediolaterally thickened with straight rami*; the supratemporal fossa is well-developed and expansive, encroaching on the posterior margin of the frontal; the quadrate ramus of the squamosal extends near vertically; a foramen is present on the posteromedial surface of the jugomaxillary ramus of the palatine*; the orbitosphenoid is ‘C’-shaped with an anteroposteriorly long ventral end; the mid-cerebral vein has a separate exit; the supraoccipital has a depression and passageway for the mid-cerebral vein; the supraoccipital is semilunate and mediolaterally wider than it is dorsoventrally tall; the paroccipital processes diverge from each other at 115° in dorsal view; the basipterygoid processes, basal tubera and basioccipital condyle lie in the same relative horizontal plane; the posterior margin of the dentary is angled posterodorsally; the mandibular fenestra is angled at 45° from the horizontal; the anteroposterior lengths of the anterior cervical centra are less than 2.6 times the dorsoventral heights of their anterior surfaces; and the mediolateral width of the distal tibia is greater than its anteroposterior length.

**Etymology.** From Xhosa and Sesotho, ‘nyana’ means son or reference to a child (Xhosa), and small or little (Sesotho), in reference to its early ontogenetic stage but comparably large body size. The specific name is in reference to Rooiwal, Eastern Cape Tribal Administrative Area 17, in which the fossil site is located.

ZooBank LSID: urn:lsid:zoobank.org:pub:F71DD6DA-11F5-4B2C-B981-B3615619187B.

## 5. Description

### 5.1. Cranial

#### 5.1.1. Overview and skull openings

The undistorted, well-preserved but incomplete cranium (figures 3–5) is missing most of the preorbital region, including the prefrontals, nasals and premaxillae. The partial right mandible lies ventral to the cranium with its lateral surface facing ventrally (figure 3*c*), and it lacks the anterior portion of the dentary and the articular. All cranial sutures remain unfused. The overall skull shape (figures 4 and 5) and proportions resemble those of *N. intloko* (BP/1/4779). As preserved, the maximum anteroposterior skull length of *Nyana* measures 213 mm, so a complete skull would exceed the length of the largest known skull of an adult *M. carinatus* (BP/1/4934; 218 mm). Scleral ossicles are preserved but are disarticulated and displaced.

**Figure 3.**
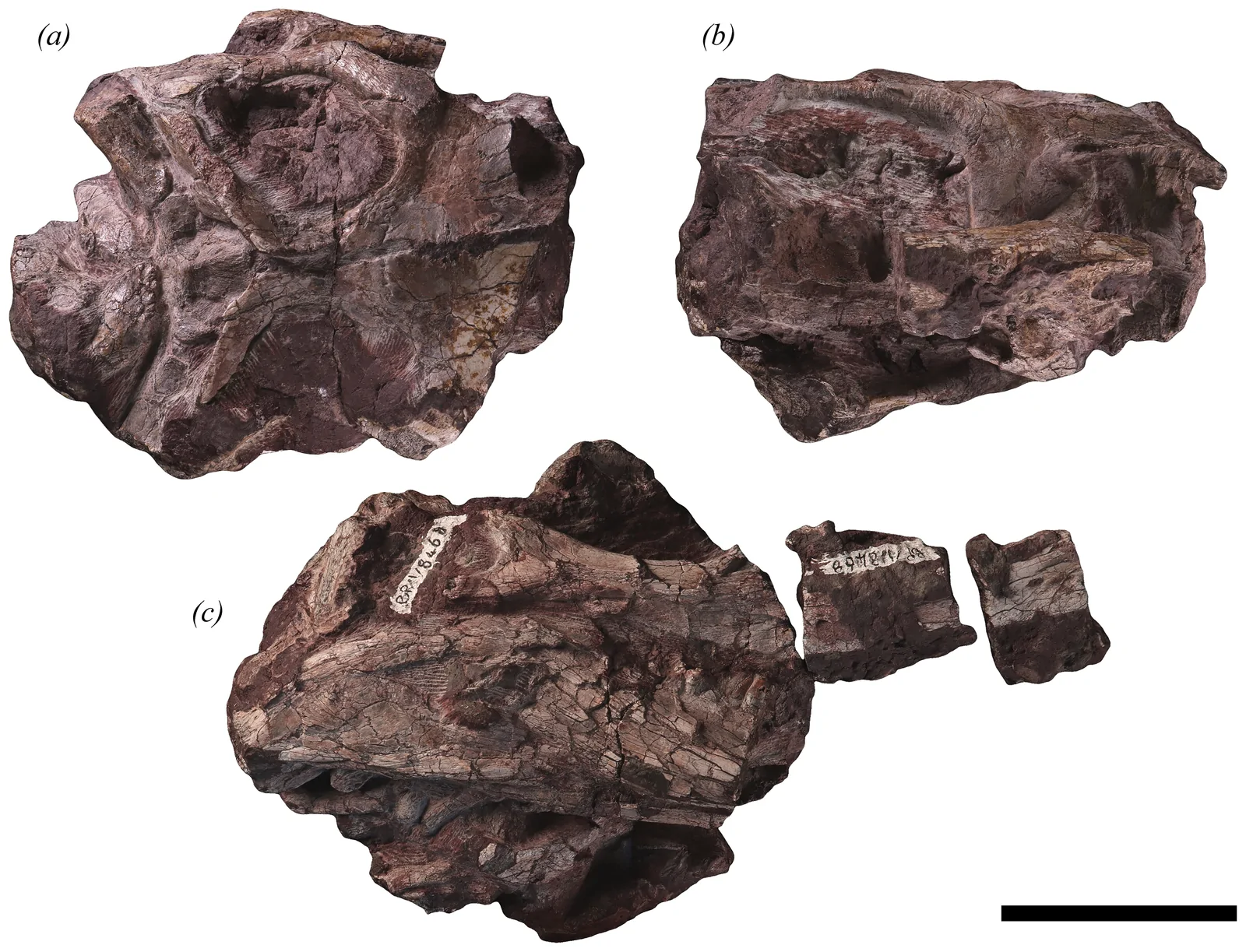
Photographs of the skull of *Nyana rooiwalensis*. (*a*) Dorsal, (*b*) left lateral and (*c*) ventral views. Scale bar = 5 cm.

**Figure 4.**
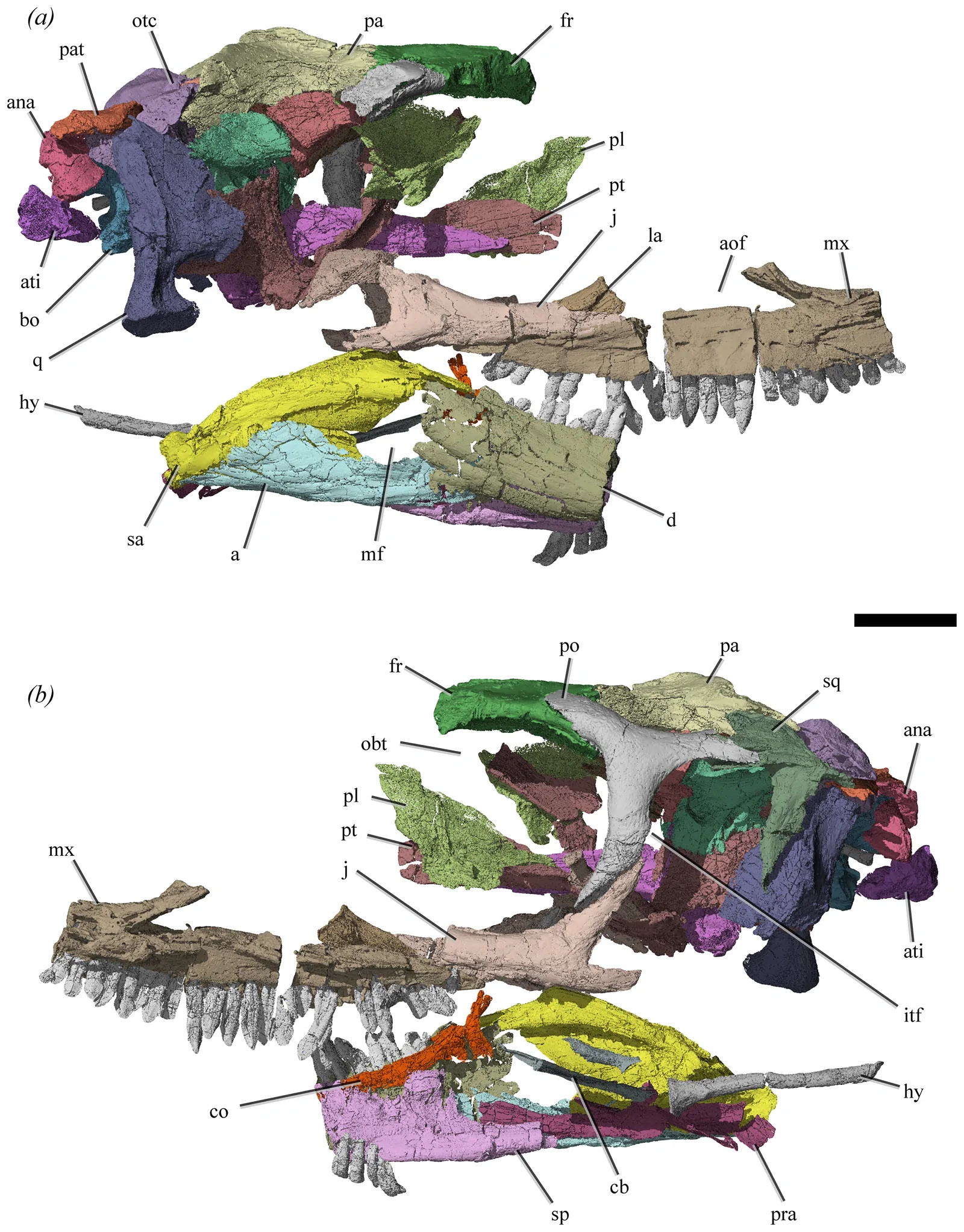
Reconstructed skull of *Nyana rooiwalensis*. (*a*) Right lateral and (*b*) left lateral views. Abbreviations: a, angular; ana, atlantal neural arch; aof, antorbital fenestra; ati, atlantal intercentrum; bo, basioccipital; cb, ceratobranchial; co, coronoid; d, dentary; fr, frontal; j, jugal; hy, hyoid; itf, infratemporal fenestra; j, jugal; la, lacrimal; mf, mandibular fenestra; mx, maxilla; obt, orbit; otc, otoccipital; pa, parietal; pat, proatlas; pl, palatine; po, postorbital; pra, prearticular; pt, pterygoid; q, quadrate; sa, surangular; sp, splenial; sq, squamosal. Scale bar = 25 mm.

**Figure 5.**
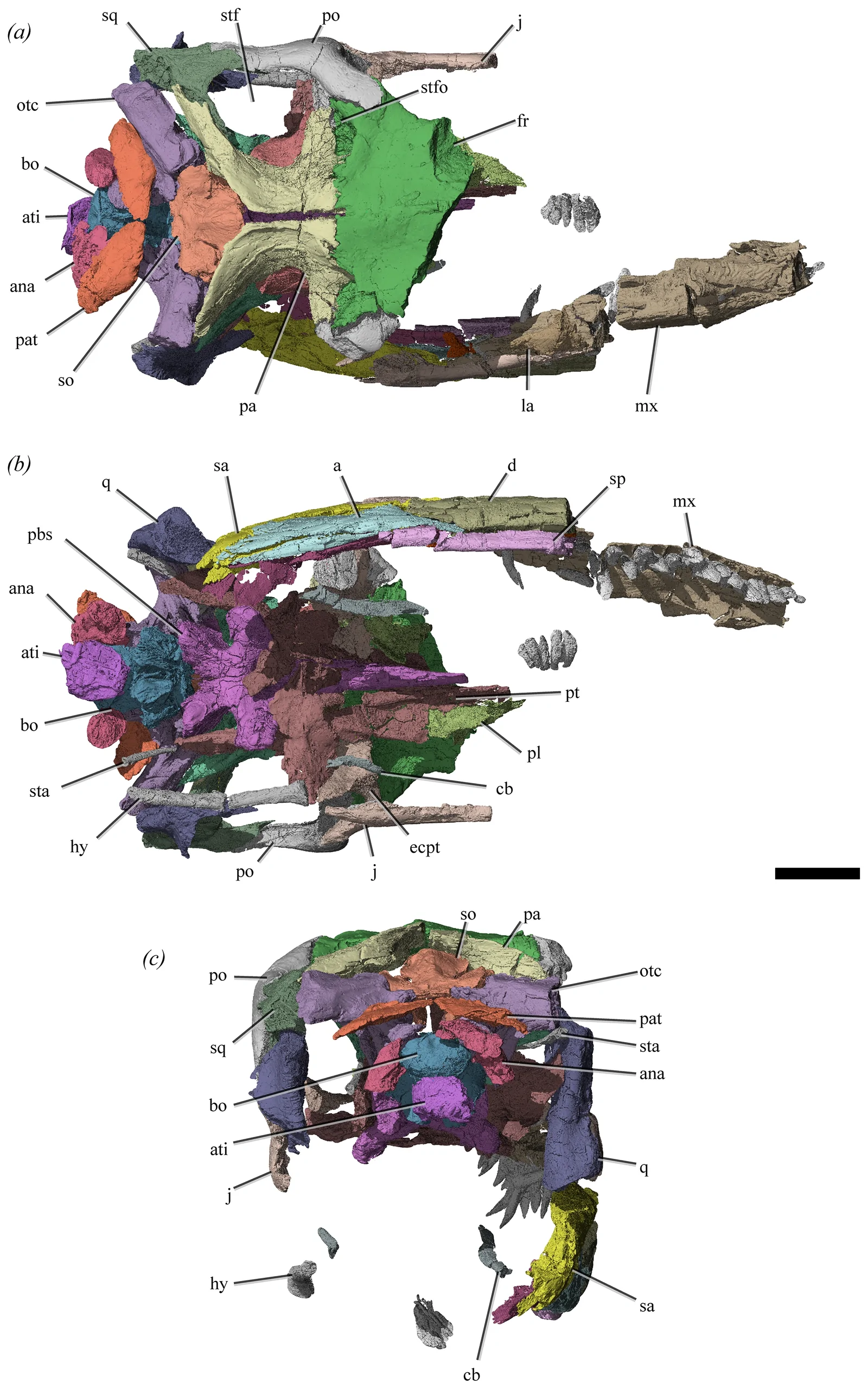
Reconstructed skull of *Nyana rooiwalensis*. (*a*) Dorsal, (*b*) ventral and (*c*) posterior views. Abbreviations: a, angular; ana, atlantal neural arch; ati, atlantal intercentrum; bo, basioccipital; cb, ceratobranchial; d, dentary; ecpt, ectopterygoid; fr, frontal; j, jugal; hy, hyoid; j, jugal; la, lacrimal; mx, maxilla; otc, otoccipital; pa, parietal; pat, proatlas; pbs, parabasisphenoid; pl, palatine; po, postorbital; pt, pterygoid; q, quadrate; sa, surangular; so, supraoccipital; sp, splenial; sq, squamosal; sta, stapes; stf, supratemporal fenestra; stfo, supratemporal fossa. Scale bar = 25 mm.

The preserved orbital margin is subcircular and bounded by the postorbital posteriorly, the frontal dorsally and the jugal ventrally. The supratemporal fenestra is triangular in dorsal view, with its apex pointing medially, and is bordered by the parietal anteriorly, medially and posteriorly, the postorbital anteriorly to laterally, and the squamosal posterolaterally. The elements bordering the preserved portion of the infratemporal fenestra include the postorbital anterodorsally, the squamosal posterodorsally and the jugal anteroventrally. The shape of the infratemporal fenestra is unclear as the quadratojugal is not preserved (see supplementary material, S2 for all cranial linear measurements).

#### 5.1.2. Maxilla

The left and right maxillae are incomplete. The posterior end of the jugal ramus of the left maxilla is preserved in two small fragments (with anteroposterior lengths of approx. 9 and approx. 20 mm). The right maxilla is preserved in three fragments, including the posterior jugal ramus, tooth-bearing ventral ramus and a section anterior to the jugal ramus, but lacks the contact for the premaxilla and nasal. Consequently, the description is based on the more complete right maxilla (figure 6). It contacts the jugal posteriorly and the lacrimal dorsally, but the contacts between the premaxilla, palatine and ectopterygoid are not preserved.

**Figure 6.**
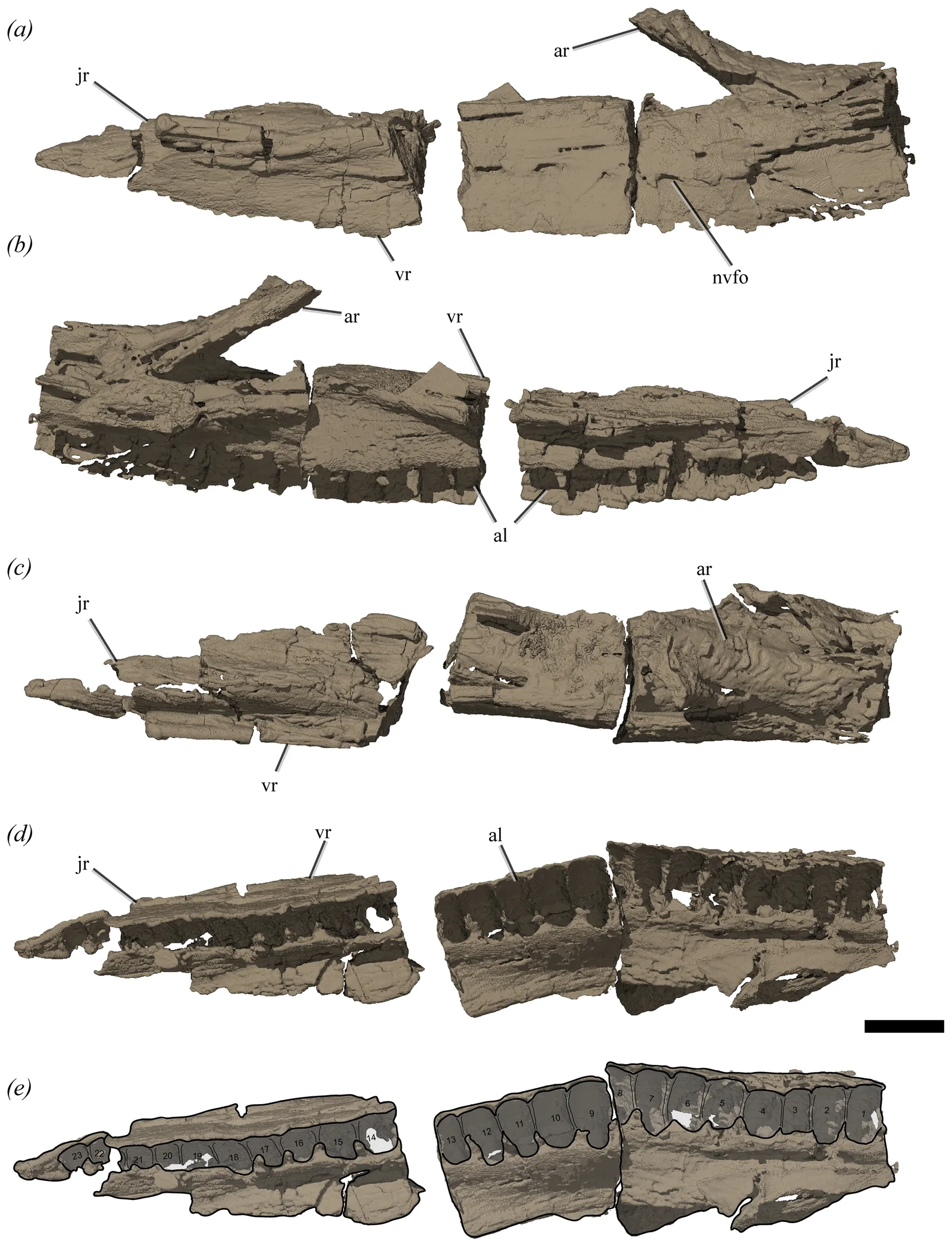
Reconstructed right maxilla of BP/1/8468. (*a*) Lateral, (*b*) medial, (*c*) dorsal and (*d*) ventral views. (*e*) A schematic of the maxilla in ventral view to show the location of the alveoli. Abbreviations: al, alveoli; ar, ascending ramus; jr, jugal ramus; nvfo, neurovascular foramina; vr, ventral ramus. Scale bar = 10 mm.

In lateral view (figure 6*a*), the ventral (tooth-bearing) ramus is straight and extends parallel to the main axis of the skull. Its lateral surface is flat to slightly concave, and at least four elliptical neurovascular foramina are preserved, which all open ventrolaterally. However, as the surface is poorly preserved, it is possible that more were present originally. Its medial surface (figure 6*b*) is flat to slightly convex with a medially projecting shelf of bone along the dorsal margin. This shelf grades into the posterior margin of the ramus, extends anteroposteriorly on the posterior half of the ramus and becomes mediolaterally wider as it extends anteriorly. The ventral ramus is dorsoventrally deepest anteriorly and tapers posteriorly to form the jugal process. Similarly, the maxilla reaches its maximum mediolateral width anteriorly and narrows posteriorly. The maxilla of *Nyana* contacts the lacrimal posterodorsally, whereas in *Riojasaurus* it does not contact the lacrimal. The tooth row is aligned laterally along its entire length.

The base of the ascending ramus is preserved and extends posterodorsally, forming an angle of approximately 40° with the ventral ramus in lateral view considered as a salient feature, whereas in *Ngwevu*, *Massospondylus*, *Unaysaurus* and *Macrocollum* this process is directed dorsally (i.e. approx. 90°) and in *Lufengosaurus* and *Plateosaurus* this angle is approximately 55°. In *Issi*, the ascending ramus extends dorsally and curves posteriorly. This process forms the anterior margin of the antorbital fenestra. Because of breakage, it is unclear whether there was maxillary contribution to a medial lamina backing the anterior of the antorbital fenestra (e.g. as occurs in *Coloradisaurus*). The base of the ascending ramus is anteroposteriorly short, and its gently concave anterior margin forms the posteroventral corner of the external naris.

The most salient feature of the maxilla is the large number of alveoli. A minimum of 23 alveoli is visible in medial and ventral views (figure 6*e*). The number of maxillary alveoli varies between taxa, such as in *Macrocollum* (21), *Adeopapposaurus* (15), *Coloradisaurus* (22), *Jingshanosaurus* (16), *Lufengosaurus* (20), *Massospondylus* (17), NMQR 3314 (19), *Plateosaurus* (25), *Riojasaurus* (24), *Sarahsaurus* (16, MCZ 8893) and *Yunnanosaurus* (15, estimated). As tooth counts vary ontogenetically [89,90], this comparison is based on maximum tooth counts. The alveoli are greatest in labiolingual diameter anteriorly and diminish in diameter posteriorly. They have subquadrate to subcircular margins in ventral view. Owing to breakage, it is not clear if the alveoli extend to the end of the jugal process or terminate before this point.

#### 5.1.3. Lacrimal

The ventral part of the right lacrimal is preserved in life position (figure 7). It contacts the jugal laterally and the maxilla medially. In lateral view (figure 7*a*), the preserved portion is subtriangular in outline but is broken dorsally, at a point ventral to the position of the nasolacrimal canal, so its articulation with the skull roof is missing. In lateral view, the posterior margin of the lacrimal is angled anterodorsally and is weakly concave. A pronounced, anterodorsally directed ridge is present on the anterolateral surface, originating close to its ventral border, and forms the posterior margin of the posteroventral corner of the antorbital fossa. A similarly developed ridge is present in *Yunnanosaurus*, *Leyesaurus*, *Ngwevu* and *Massospondylus*. A thin flange of bone that is medially inset from and anterior to this ridge would have formed at least a partial medial lamina, backing this section of the antorbital fossa. By contrast, the medial surface (figure 7*b*) of the lacrimal is smooth and featureless.

**Figure 7.**
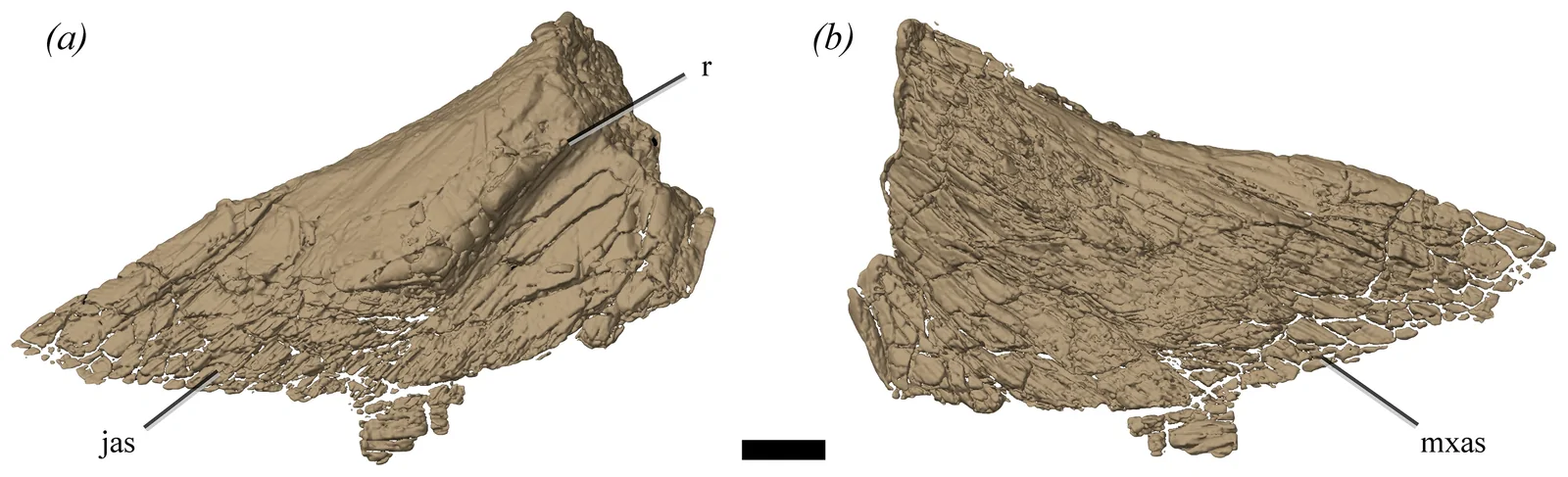
Reconstructed right lacrimal of BP/1/8468. (*a*) Lateral and (*b*) medial views. Abbreviations: jas, jugal articular surface; mxas, maxilla articular surface; r, ridge. Scale bar = 3 mm.

#### 5.1.4. Postorbital

A complete left and a partial right postorbital are preserved, which are undistorted and in life position (figure 8). The postorbital forms the posterior margin of the orbit, the anterolateral corner of the supratemporal fenestra and the anterodorsal corner of the infratemporal fenestra. It is a triradiate bone consisting of a frontal ramus, a jugal ramus and a squamosal ramus. It contacts the frontal anterodorsally, the jugal posteroventrally and the squamosal posteromedially.

**Figure 8.**
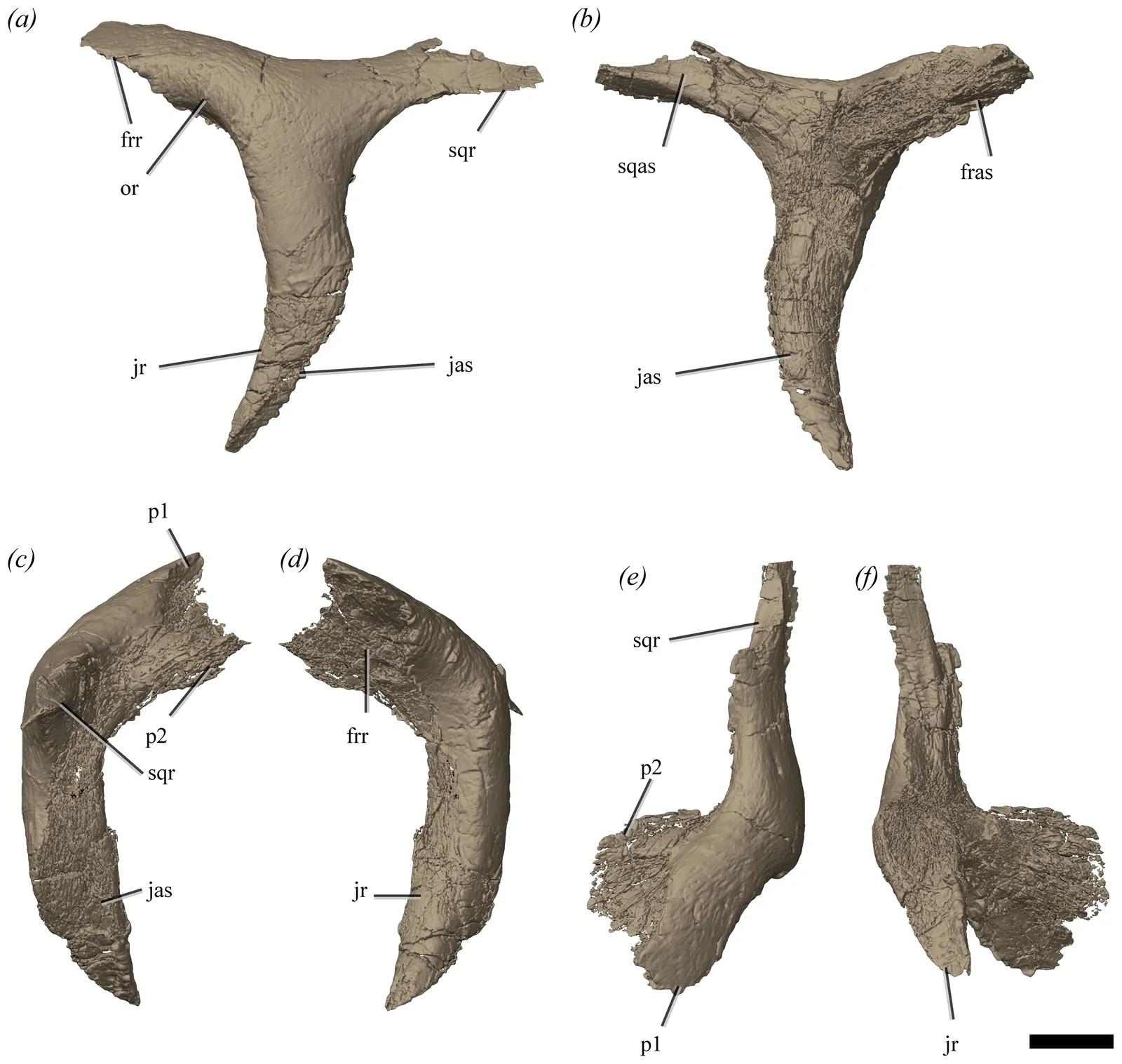
Reconstructed left postorbital of BP/1/8468. (*a*) Lateral, (*b*) medial, (*c*) posterior, (*d*) anterior, (*e*) dorsal and (*f*) ventral views. Abbreviations: fras, frontal articular surface; frr, frontal ramus; jas, jugal articular surface; jr, jugal ramus; p1, dorsal process of frontal ramus; p2, ventral process of frontal ramus; or, orbital rim; sqas, squamosal articular surface; sqr, squamosal ramus. Scale bar = 10 mm.

The postorbital is notably robust, with rami and a main body that are proportionally short anteroposteriorly and wide mediolaterally, similar to those of *Ngwevu*,* Lufengosaurus*,* Riojasaurus*,* Kholumolumo* and *Sarahsaurus*. This differs from other closely related sauropodomorphs that have anteroposteriorly longer and mediolaterally narrower rami, such as *Massospondylus*, *Adeopapposaurus*, *Leyesaurus*, *Coloradisaurus*, *Issi* and *Plateosaurus*. The postorbital of *Yunnanosaurus* is thicker mediolaterally and longer anteroposteriorly compared to *Nyana*. The postorbital rami of *Nyana* are relatively straight in lateral view (figure 8*a*), considered a salient feature of *Nyana*, similar to those of *Plateosaurus* and *Sarahsaurus*, differing from the arc-like or semicircular rami observed in other EBSMs (e.g. *Issi*, *Massospondylus*, SMF 13.5.37, *Adeopapposaurus*, *Lufengosaurus* and *Kholumolumo*).

The frontal ramus extends anteromedially, with a slight dorsal incline. Its dorsal margin is convex, and its ventral margin is slightly concave. The dorsal surface is smooth, whereas the ventral surface is faintly rugose. The frontal ramus is mediolaterally wider than its dorsoventral height at its base. It bifurcates anteriorly to clasp the posterodorsal corner of the frontal between its dorsal and ventral processes. This forked morphology is also present in other taxa, such as *Macrocollum*, *Adeopapposaurus*, *Coloradisaurus*, *Lufengosaurus*, *Sarahsaurus* and *Ngwevu*. The dorsal process of the frontal ramus (p1) contacts the frontal anteromedially, and its ventral process (p2) contacts the frontal and the parietal anteroventrally. In dorsal view (figure 8*e*), the ventral process of the frontal ramus forms the anterolateral border of the supratemporal fossa, together with the frontal and parietal. A key feature of the postorbital is that this forked frontal ramus contacts both the frontal and parietal, allowing for all three elements to contribute to the supratemporal fossa, distinguishing it from *Massospondylus* and SMF 13.5.37. This feature is widely distributed among other sauropodomorphs and is present in *Macrocollum*, *Adeopapposaurus*, *Ngwevu* and *Sarahsaurus*. The distal end of the frontal ramus is expanded in all dimensions and is not as dorsoventrally flattened as that of *Massospondylus*.

The jugal ramus is ventrally directed and arcs anteriorly at its distal end as it extends ventrally, such that its anterior margin is concave, while its posterior margin is convex (figure 8*a*). This condition is present in other EBSMs, such as *Macrocollum*, *Adeopapposaurus*, *Coloradisaurus*, *Leyesaurus*, *Lufengosaurus*, *Massospondylus*, *Anchisaurus* and NMQR 3314, but differs from *Kholumolumo* whose anterior margin is more strongly concave. The jugal ramus in *Nyana* is proportionally mediolaterally wider and anteroposteriorly longer than that of *Massospondylus*, but not to the extent seen in NMQR 3314. The mediolateral width and anteroposterior length of the jugal ramus remain consistent as it extends ventrally. It has an expansive jugal articular surface located on the medial surface of the ramus that is dorsoventrally tall. The lateral surface of the jugal ramus is weakly convex in anterior view with a smooth surface. The frontal ramus and the jugal ramus together form the posterior margin of the orbit.

The squamosal ramus is the mediolaterally narrowest of the three rami and forms part of the anterolateral border of the supratemporal fenestra and the anterodorsal corner of the infratemporal fenestra. It extends posteriorly from the dorsal portion of the postorbital and contacts the squamosal posteromedially. This differs from *Massospondylus* and *Yunnanosaurus*, in which the squamosal ramus extends posteriorly from the midpoint of the postorbital main body. The posterior end of the ramus forms a tab-like structure on its medial side that inserts into a cavity on the postorbital ramus of the squamosal. As the squamosal ramus extends posteriorly, it tapers distally. The squamosal ramus has a weakly convex dorsal margin in lateral view with a smooth, lateral surface.

#### 5.1.5. Squamosal

A complete left squamosal is well-preserved and undistorted (figure 9). It is a tetraradiate bone with a postorbital ramus, parietal ramus, exoccipital ramus and a quadrate ramus. It forms the posterodorsal corner of the skull. The squamosal contacts the postorbital anteromedially, the parietal dorsomedially, the otoccipital posteromedially and the quadrate ventromedially. The dorsal surface of the squamosal body is convex and smooth.

**Figure 9.**
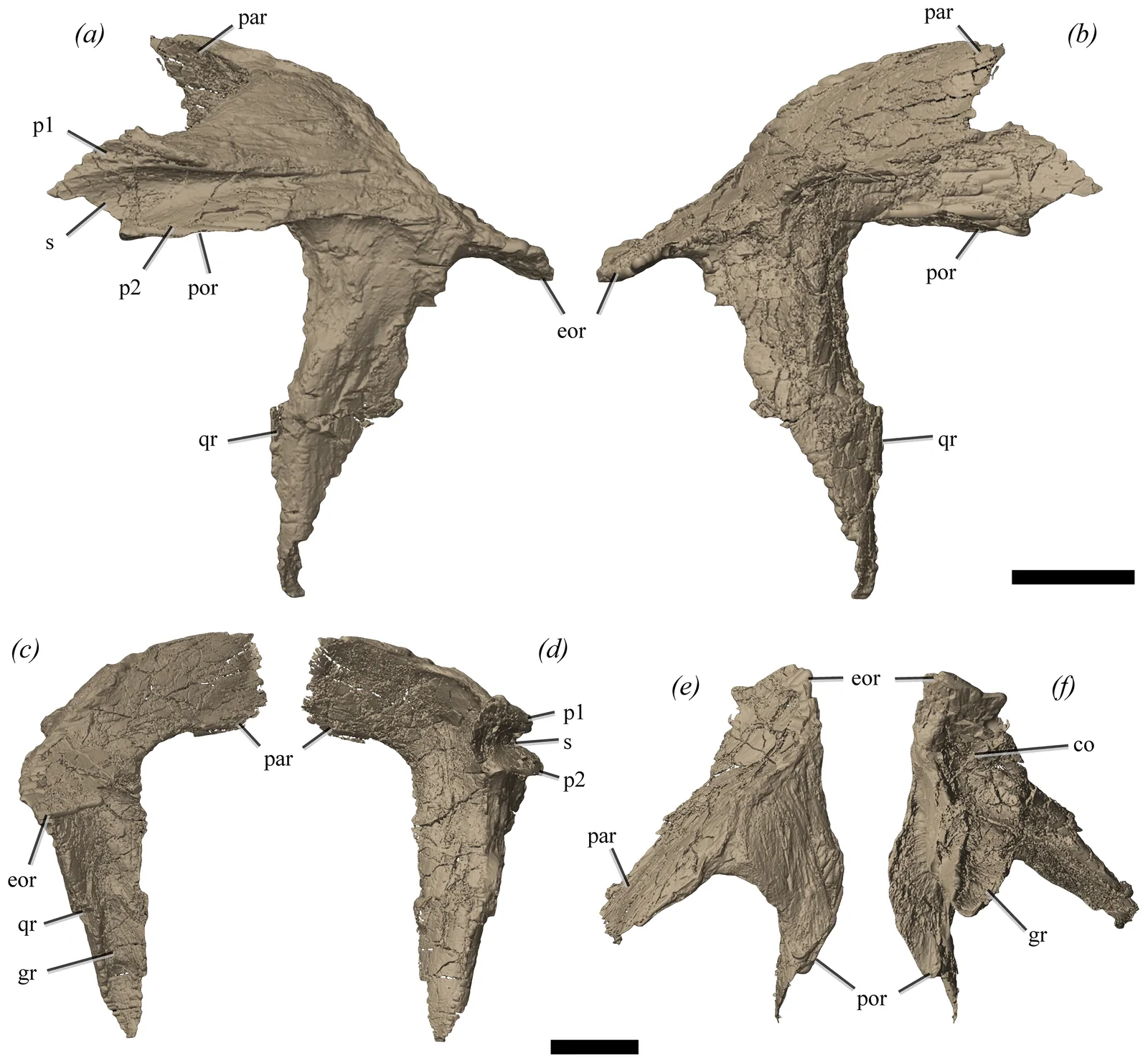
Reconstructed left squamosal of BP/1/8468. (*a*) Lateral, (*b*) medial, (*c*) posterior, (*d*) anterior, (*e*) dorsal and (*f*) ventral views. Abbreviations: co, cotyle; eor, exoccipital ramus; gr, groove; par, parietal ramus; por, postorbital ramus; qr, quadrate ramus; p1, dorsal process of postorbital ramus; p2, ventral process of postorbital ramus; s, sulcus. Scale bars = 10 mm.

The postorbital ramus extends anteriorly, expanding dorsoventrally and forking into dorsal (p1) and ventral (p2) processes as it terminates anteriorly (figure 9*a*,*d*). These processes are distinct, anteriorly tapering, mediolaterally expanded flanges that diverge to define a triangular sulcus. The medial and lateral surfaces of these flanges are smooth. This sulcus receives the squamosal ramus of the postorbital. A ridge that extends along the lateral margin of the ventral flange of the postorbital ramus grades into the squamosal body and onto the lateral margin of the quadrate ramus. The medial surface of the postorbital ramus faces strictly medially. This ramus is proportionately anteroposteriorly longer than those in *Massospondylus* and *Adeopapposaurus* but is not as anteroposteriorly long as that of *Plateosaurus*. The ventral margin of the ventral flange forms the posterodorsal corner of the infratemporal fenestra. The medial margin of the dorsal flange contributes to the posterolateral corner of the supratemporal fenestra.

The parietal ramus is dorsoventrally broad and extends anteromedially from the squamosal body. It contacts the parietal ventrally, but it is unclear whether it contacts the supraoccipital. This ramus is rectangular in medial view and has a dorsoventrally tall distal end. The medial and lateral surfaces of the parietal ramus are smooth. The angle between the postorbital and parietal rami in dorsal view (figure 9*e*) is 70°, which is greater than in *Massospondylus* (50°), similar to that seen in *Sarahsaurus* (approx. 65°), and less than the angle in *Ngwevu* (85°).

The exoccipital ramus projects posteriorly and is triangular in dorsal view. This ramus extends further posteriorly in *Issi* than in *Nyana*. It lies ventral to the otoccipital. It is angled ventrally at 35° to the dorsoventral axis of the skull. The dorsal and ventral surfaces of this ramus are smooth and featureless. A deep fossa is located on the ventral surface of the squamosal body between the otoccipital ramus and the quadrate ramus, forming the quadrate cotyle (figure 9*f*). It is bordered by prominent ridges that run along the posterior and medial margins of the quadrate ramus.

The quadrate ramus extends almost strictly ventrally and is triangular in transverse cross-section with its apex pointing anterolaterally. The ventral projection of the ramus is similar to the condition in *Lufengosaurus*, NMQR 3314, *Plateosaurus* and *Ngwevu* but differs from other EBSMs such as *Massospondylus* and *Anchisaurus* in which it is directed anteriorly. The quadrate ramus is the longest of the four rami. The dorsoventral height of the quadrate ramus is 2.3 times its anteroposterior length, contrasting markedly with those of *Massospondylus*, *Ngwevu* and *Plateosaurus* where it is greater than four times the length. In lateral view, the anterior margin of the quadrate ramus forms the posterior border of the infratemporal fenestra. As it descends, the quadrate ramus divides into two sheets of bone, one that is directed laterally and the other directed posteriorly. The medially facing groove between them forms the articular surface for the quadratojugal ramus of the quadrate. The anterior margin of the quadrate ramus is directed anteriorly, flat and smooth.

#### 5.1.6. Jugal

Nearly complete left and right jugals are preserved and located slightly out of life position (figure 10). The jugals display a rotated ‘Y’-shaped morphology in lateral view (figure 10*a*,*d*), with the opening of the ‘Y’ directed posteriorly, as is typical of many other EBSMs. The jugal consists of three rami: the maxillary ramus, the postorbital ramus and the quadratojugal ramus. The jugal contacts the maxilla anteroventrally, the lacrimal anterodorsally and the postorbital posterodorsally. The maxillary and postorbital rami form the ventral and posteroventral margins of the orbit, respectively. The quadratojugal and postorbital rami form the anteroventral corner of the infratemporal fenestra.

**Figure 10.**
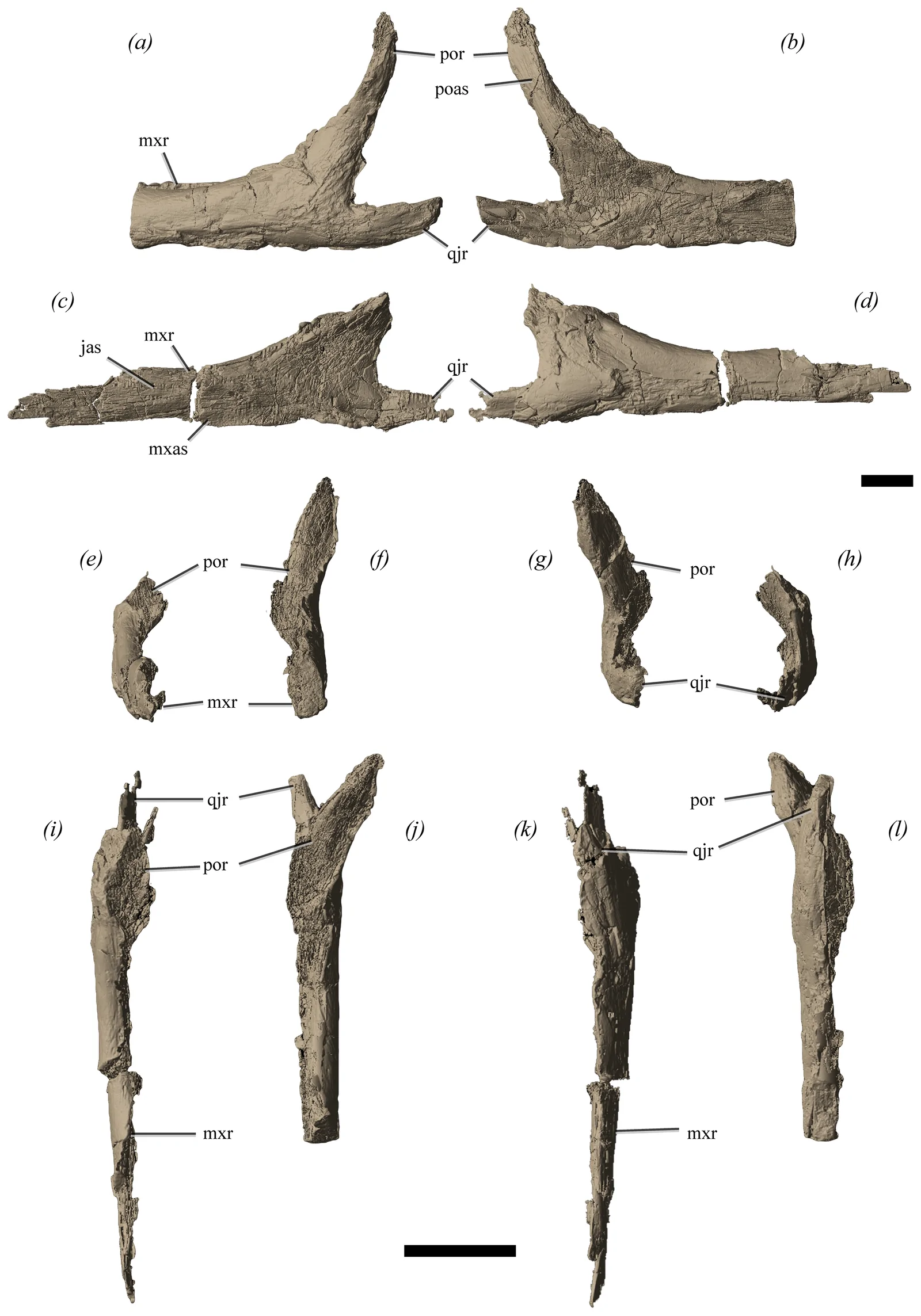
Reconstructed left and right jugals of BP/1/8468. (*a,d*) Lateral, (*b,c*) medial, (*e,f*) anterior, (*g,h*) posterior, (*i,j*) dorsal and (*k,l*) ventral views. Abbreviations: jas, jugal articular surface; mxr, maxillary ramus; poas, postorbital articular surface; por, postorbital ramus; qjr, quadratojugal ramus. Scale bars: (*a–h*) = 8 mm, (*i–l*) = 15 mm.

The jugal is anteroposteriorly elongated, and its maxillary ramus tapers anteriorly. The maxillary ramus is not as dorsoventrally deep as in *Lufengosaurus*. The overlapping contact between the jugal and maxilla accounts for approximately 65% of the anteroposterior length of the right jugal. The lateral surface of the maxillary ramus is convex and smooth, while its medial surface is flat and smooth.

The postorbital ramus is directed posterodorsally and is the mediolaterally thickest ramus. Its medial surface is flat, and its lateral surface is convex. The postorbital ramus underlaps the jugal ramus of the postorbital. This contact surface is dorsoventrally expanded and directed anteriorly. The medial and lateral margins of the postorbital ramus are bordered by scarp-like ridges, similar to those of *Plateosaurus*.

The quadratojugal ramus is directed dorsally and angled slightly posteriorly, differing from the posterodorsally directed rami of *Ngwevu* and *Massospondylus* and the posteroventrally directed ramus of SMF 13.5.37. It is mediolaterally thin and anteroposteriorly short with smooth medial and lateral surfaces. An ovoid fossa on the medial side of the ramus is shallow, with its long axis extending anteroposteriorly, and is bordered by weak dorsal and ventral margins. The angle formed between the postorbital and quadratojugal ramus in lateral view is approximately 70°, similar to that in *Plateosaurus*, *Massospondylus* and *Ngwevu*.

#### 5.1.7. Quadrate

A partial left (figure 11) and near-complete right quadrate (figure 12) are preserved close to their life positions. The left quadrate preserves the pterygoid and quadratojugal rami, and the right quadrate preserves the pterygoid ramus, quadratojugal ramus and the ventral condyles. The quadrate head is not preserved in either element. The quadrate contacts the squamosal anterodorsally and the pterygoid medially, and probably contacts the quadratojugal, but this is not preserved in BP/1/8468.

**Figure 11.**
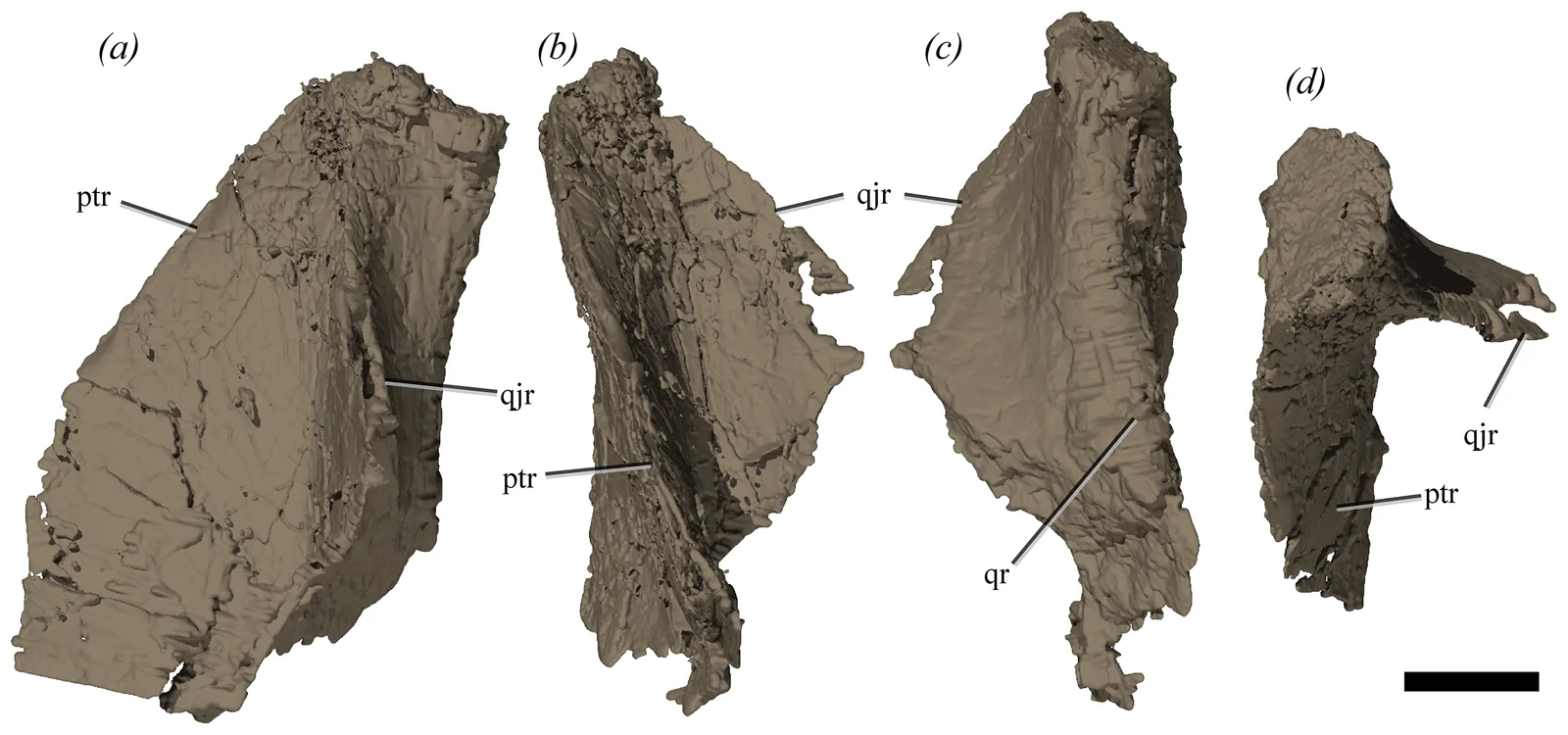
Reconstructed left quadrate of BP/1/8468. (*a*) Lateral, (*b*) anterior, (*c*) posterior and (*d*) dorsal views. Abbreviations: ptr, pterygoid ramus; qjr, quadratojugal ramus; qr, quadrate ridge. Scale bar = 8 mm.

**Figure 12.**
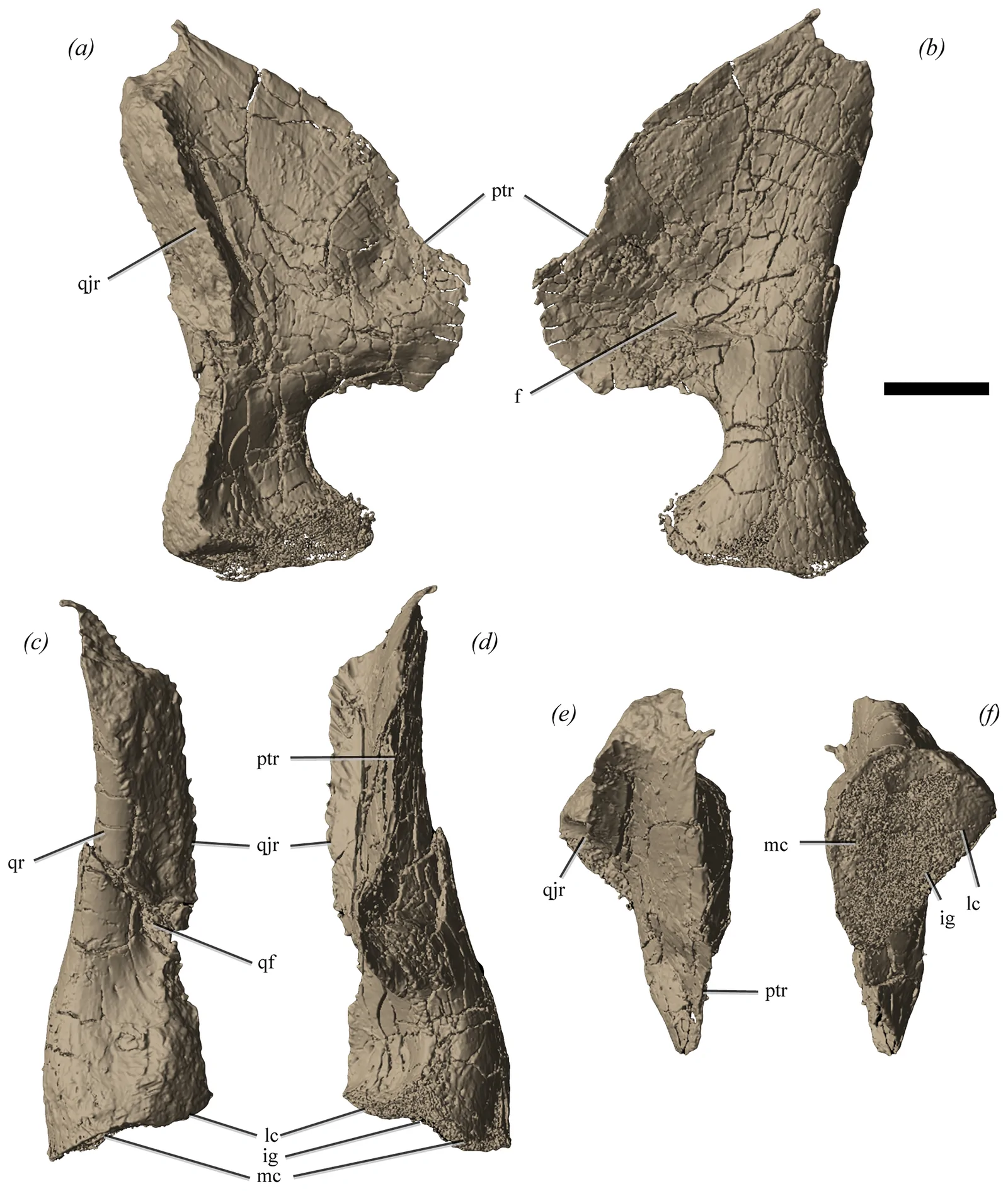
Reconstructed right quadrate of BP/1/8468. (*a*) Lateral, (*b*) medial, (*c*) posterior, (*d*) anterior, (*e*) dorsal and (*f*) ventral views. Abbreviations: f, fossa; ig, intercondylar groove; lc, lateral condyle; mc, medial condyle; ptr, pterygoid ramus; qf, quadrate foramen; qjr, quadratojugal ramus; qr, quadrate ridge. Scale bar = 10 mm.

The quadrate of *Nyana* is proportionally dorsoventrally shorter and anteroposteriorly longer than the slender quadrate of *Macrocollum*, *Massospondylus* or *Plateosaurus* but is proportionally mediolaterally narrower than the mediolaterally thick quadrate seen in NMQR 3314. A quadrate foramen (figure 12*c*) is located at the base of the quadratojugal ramus on the posterolateral surface of the quadrate shaft. This is a widespread feature that occurs in *Macrocollum*, *Adeopapposaurus*, *Coloradisaurus*, *Jingshanosaurus*, *Leyesaurus*, *Massospondylus*, *Ngwevu* and *Sarahsaurus.* By contrast, *Plateosaurus* and *Riojasaurus* do not have a quadrate foramen.

In posterior view (figure 12*c*), the quadrate is pillar-like but has a slightly sigmoidal outline with its dorsal portion directed laterally and its ventral portion directed medially. The quadrate ridge, as described by Chapelle & Choiniere [21], extends along the posterodorsal to posteromedial margin of the quadrate and is mediolaterally thicker in *Nyana* than in *Massospondylus*. The shaft has a convex medial margin and concave lateral margin in posterior view. The main shaft is proportionally dorsoventrally shorter than in *Massospondylus* and *Leyesaurus*, but it is similar to that of *Ngwevu*. Ventral to the quadratojugal ramus, a ridge extends along the lateral margin of the shaft to merge with the lateral condyle.

The pterygoid ramus is an anteriorly expanded, mediolaterally thin sheet. In lateral view, it extends anteriorly to a point well beyond the margin of the quadrate condyles. Its dorsoventral height occupies approximately 70% of the total dorsoventral height of the quadrate. The pterygoid ramus has a subtriangular outline in lateral view and is subequal in dorsoventral height to anteroposterior length, similar to the condition seen in *Sarahsaurus* and differing from the dorsoventrally tall ramus of *Massospondylus* and *Plateosaurus*. The ventral margin of the pterygoid ramus and the anterior margin of the shaft define a deep concavity that is visible in lateral view. A broad fossa is located on the medial surface of the base of the pterygoid ramus, bordered posteriorly by the quadrate shaft and ventrally by a low shelf. This fossa is probably continuous with a similar fossa on the medial surface of the quadrate ramus of the pterygoid. The medial surface of the ramus is weakly concave, and its lateral surface is flat.

The quadratojugal ramus is present but lacks its anterior margin. In ventral view, the quadratojugal and pterygoid rami diverge from each other at approximately 95° similar to *Ngwevu*, but differing from the acute angle in *Massospondylus* and *Plateosaurus*. It is a triangular sheet in posterior view, and both its anterior and posterior surfaces are smooth.

In ventral view (figure 12*f*), the articular condyles are separated by a shallow, anteromedially oriented intercondylar groove. The medial condyle is anteroposteriorly longer than the triangular lateral condyle, but both condyles are similar in mediolateral width. The medial condyle extends further ventrally than the lateral condyle. As a result, the ventral margin of the condyles is angled ventromedially. The distal end is anteroposteriorly longer than the quadrate shaft, unlike in *Plateosaurus* where the distal end is mediolaterally wider than the shaft.

### 5.2. Palate

The preserved palate includes the pterygoid, ectopterygoid and palatine (figures 13 and 14). The vomers are missing. The palate is well-preserved and articulated in near life position, although the anterior portions of the right pterygoid and right palatine are dorsally displaced.

**Figure 13.**
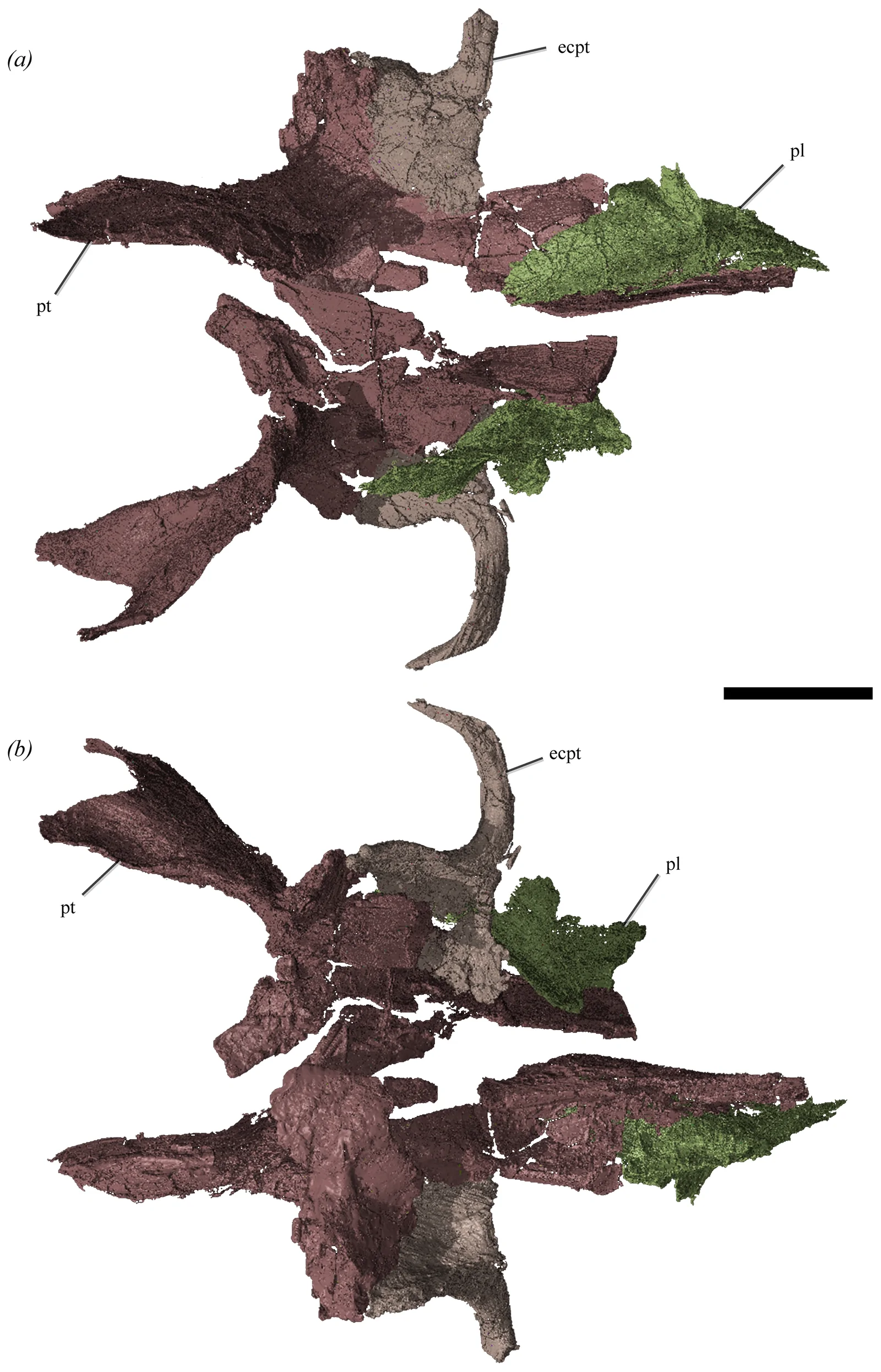
Reconstructed palate of BP/1/8468. (*a*) Dorsal and (*b*) ventral views. Abbreviations: ecpt, ectopterygoid; pl, palatine; pt, pterygoid. Scale bar = 20 mm.

**Figure 14.**
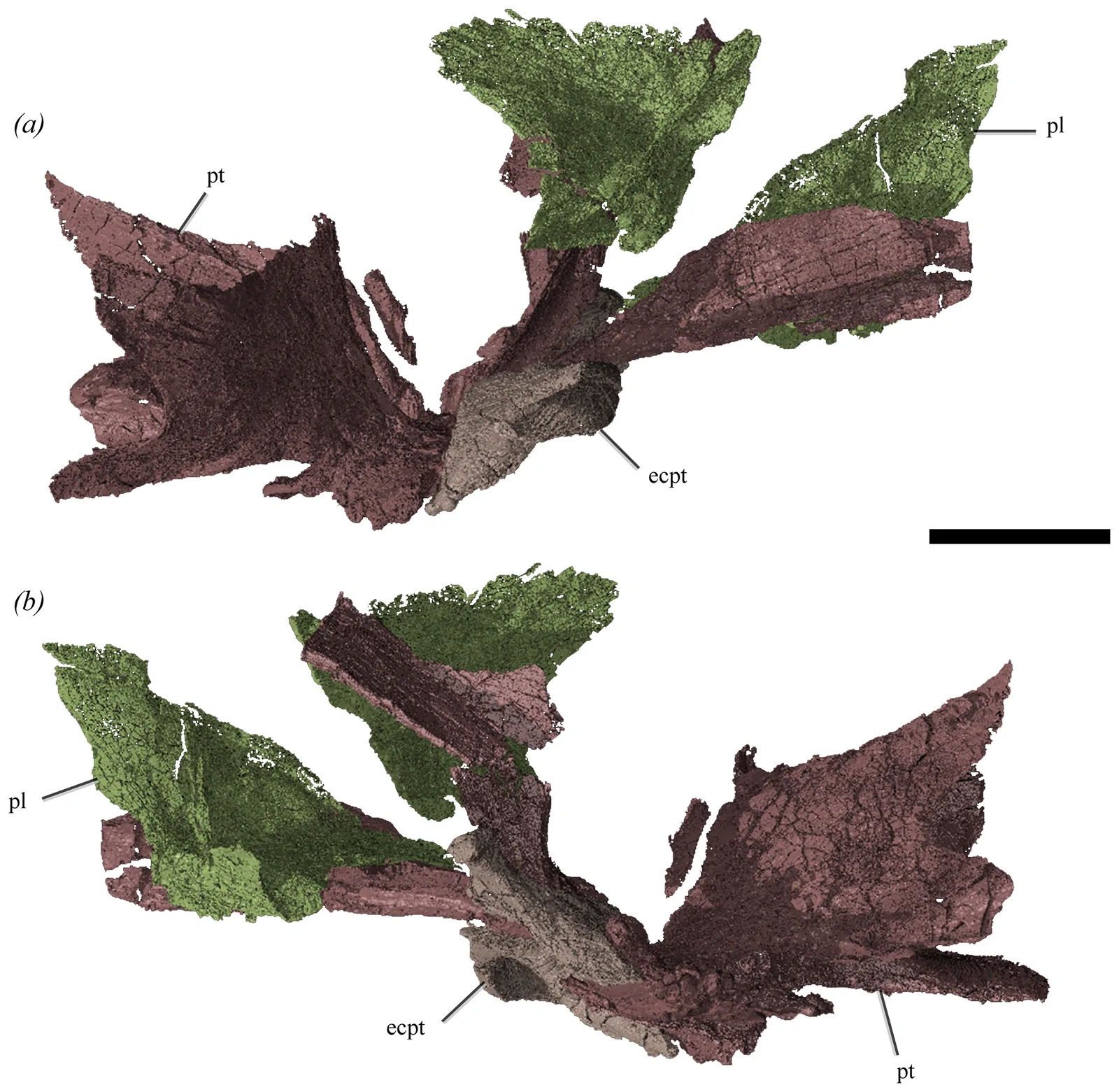
Reconstructed palate of BP/1/8468. (*a*) Right lateral and (*b*) left lateral views. Abbreviations: ecpt, ectopterygoid; pl, palatine; pt, pterygoid. Scale bar = 20 mm.

#### 5.2.1. Pterygoid

Both pterygoids are generally well-preserved and in life position, but they exhibit some distortion and are incomplete (figure 15). The anterior portion of the right pterygoid is angled dorsally. It can be subdivided into the pterygoid body, quadrate ramus, basisphenoid ramus, palatal ramus and the ectopterygoid ramus. The pterygoid contacts the quadrate posterolaterally, the parabasisphenoid posteromedially and the palatine anterolaterally. In dorsal view, the pterygoids in *Nyana* are proportionally mediolaterally wide and anteroposteriorly short, similar to the proportions of *Ngwevu*, *Adeopapposaurus* and *Sarahsaurus*, whereas in *Massospondylus* and *Plateosaurus* the pterygoids are mediolaterally narrower and anteroposteriorly longer.

**Figure 15.**
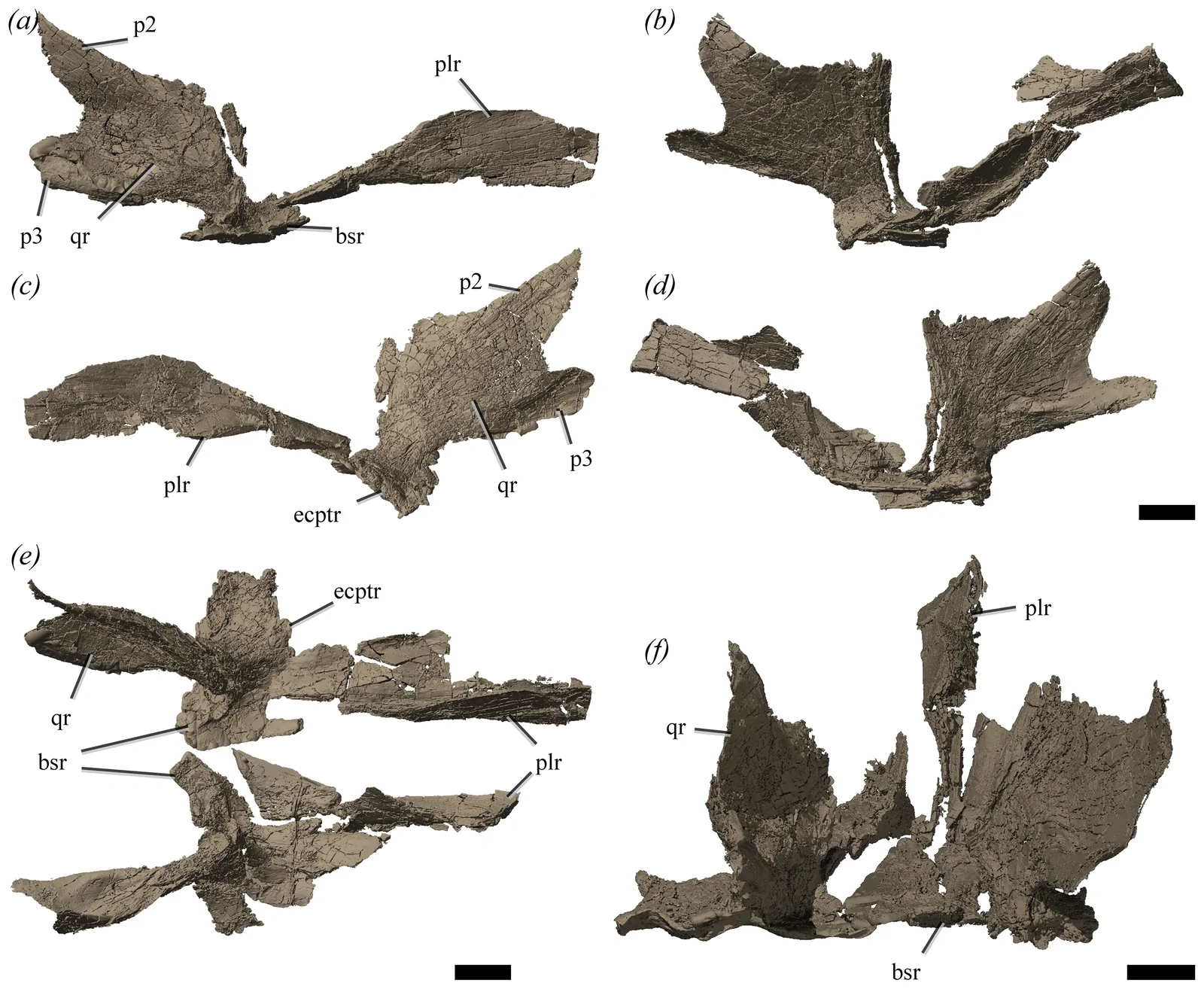
Reconstructed left and right pterygoids of BP/1/8468. (*a, d*) Medial, (*b, c*) lateral, (*e*) dorsal and (*f*) posterior views. Abbreviations: bsr, basipterygoid ramus; ecptr, ectopterygoid ramus; p, process; plr, palatal ramus; qr, quadrate ramus. Scale bars: (*a–e*) = 10 mm, (*f*) = 9 mm.

The main body of the pterygoid is a compressed lamina that is positioned ventral to all the other pterygoid rami. A thin sheet, the palatal ramus, extends anteriorly from the main pterygoid body. It is oriented horizontally as it extends from the main body and rotates 90° as it extends anteriorly.

The quadrate ramus is a large, mediolaterally thin sheet that expands dorsoventrally and posterolaterally. It is connected to the main body by an anteroposteriorly short, mediolaterally narrow neck-like structure and is directed dorsally as it extends posteriorly. Posteriorly, the quadrate ramus forks into two processes, referred to as p2 and p3, consistent with *Massospondylus* [21]. Both of these processes contact the pterygoid ramus of the quadrate. The dorsal process extends posterodorsally from the quadrate ramus at 45° from the vertical axis, and the ventral process extends posteriorly from the ventral margin of the ramus. In lateral view, the dorsal process tapers towards its distal tip, and the apex of the ventral process is rounded and tab-like. An additional process that is sometimes present in other taxa (e.g. *Massospondylus*, *Ngwevu* and *Plateosaurus*), arising from the dorsalmost margin of the ramus, is either broken or absent in BP/1/8468. The medial surface of the quadrate ramus is gently depressed between the dorsal and ventral processes with the long axis directed anteroventrally to posterodorsally. The rest of the medial surface is gently concave, and the lateral surface of the quadrate ramus is convex.

The basisphenoid ramus arises from the medial margin of the pterygoid and is better preserved on the left pterygoid. It extends posteriorly and is ovoid in dorsal view. This ramus is connected to the pterygoid body by a distinct lamina that extends ventrally from the ventral margin of the pterygoid body and contacts the ramus on its dorsal margin. The basisphenoid ramus displays the same hook-like morphology present in other EBSMs (e.g. *Massospondylus*, *Ngwevu*, *Adeopapposaurus*, *Plateosaurus*, NMQR 3314, *Jingshanosaurus* and *Lufengosaurus*) and clasps the basipterygoid process of the parabasisphenoid. This ramus has a weakly concave dorsal surface and a weakly convex ventral surface.

The ectopterygoid ramus is located on the lateral side of the main pterygoid body. It is a rectangular flange that lies anterior to the quadrate ramus. The ramus is subequal in mediolateral width and anteroposterior length.

#### 5.2.2. Ectopterygoid

Both ectopterygoids are well-preserved, partially complete and lie close to their original position (figure 16). The left ectopterygoid lacks its jugal ramus. In ventral view (figure 16*b*), the ectopterygoid is hook-shaped and consists of a jugal and pterygoid ramus. It is dorsoventrally flattened and is positioned on the lateral margins of the palate. Its internal structure is solid.

**Figure 16.**
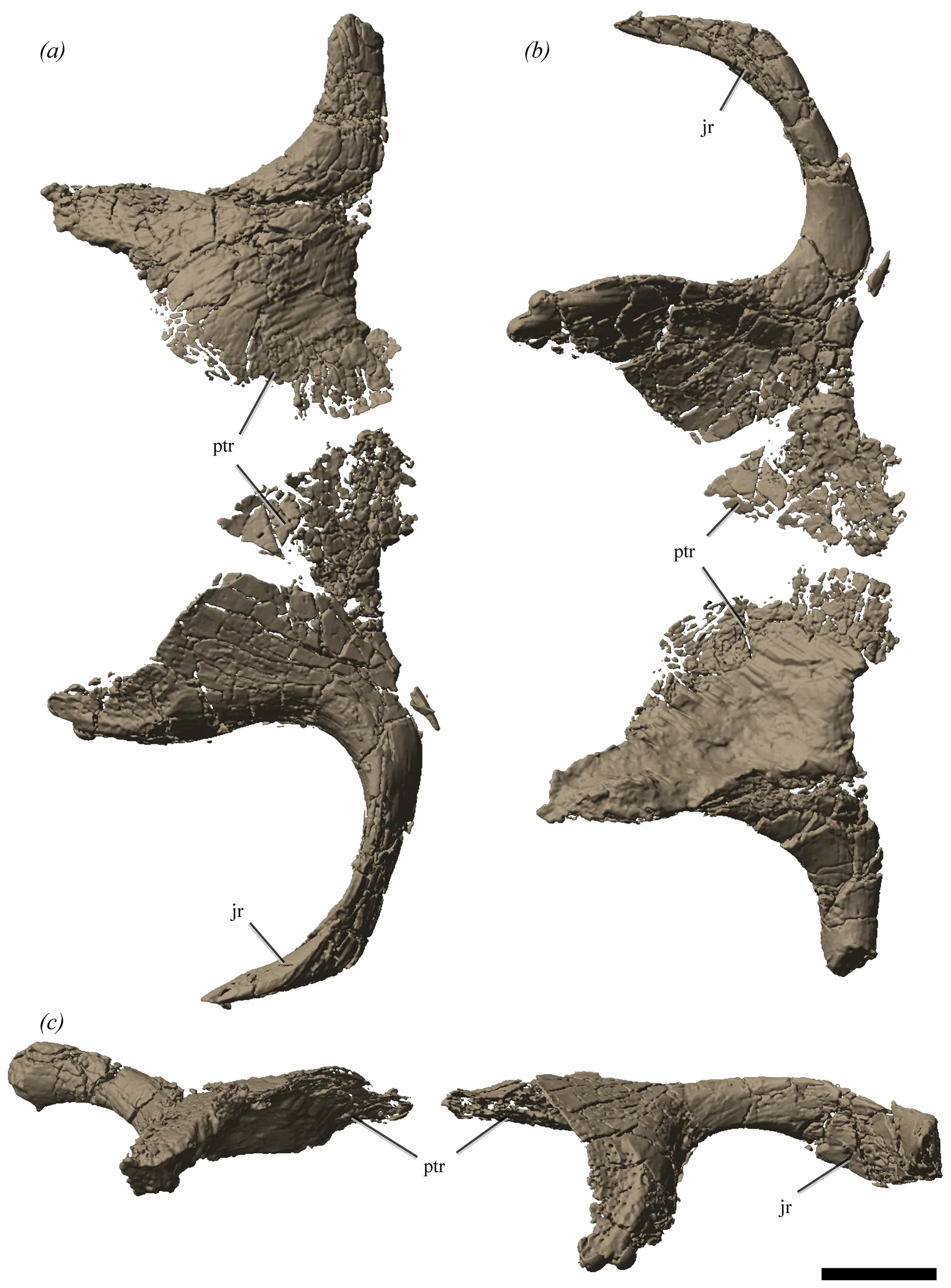
Reconstructed left and right ectopterygoids of BP/1/8468. (*a*) Dorsal, (*b*) ventral and (*c*) posterior views. Abbreviations: jr, jugal ramus; ptr, palatal ramus. Scale bar = 8 mm.

The jugal ramus is strongly recurved in ventral view, producing the hook-like morphology of the ectopterygoid. It is dorsoventrally flattened at its proximal end, twists as it extends to form its hook and becomes anteroposteriorly compressed as it extends distally, similar to that of *Issi*. The jugal ramus of *Nyana* appears slender compared to the robust jugal ramus in *Ngwevu*. The tapered apex of the ramus is directed posteriorly. Its dorsal and ventral surfaces are smooth.

The pterygoid ramus forms the base of the ectopterygoid. It has a convex dorsal surface and a concave ventral surface. The ramus is anteroposteriorly longer than it is mediolaterally wide. The pterygoid ramus has a sigmoidal medial surface where it contacts the pterygoid, whereas this surface is straight in *Massospondylus*.

#### 5.2.3. Palatine

Both palatines are partially preserved, close to their life positions (figure 17). The right palatine is dorsally displaced but is preserved in the correct position relative to the (also displaced) right pterygoid. Both palatines preserve a partial vomerine ramus, a pterygoid ramus and a jugomaxillary ramus. It contacts the jugal dorsolaterally, the maxilla ventrolaterally and the pterygoid posteriorly. The contact between the palatine and maxilla is unclear, and the anterior contact with the vomer is missing.

**Figure 17.**
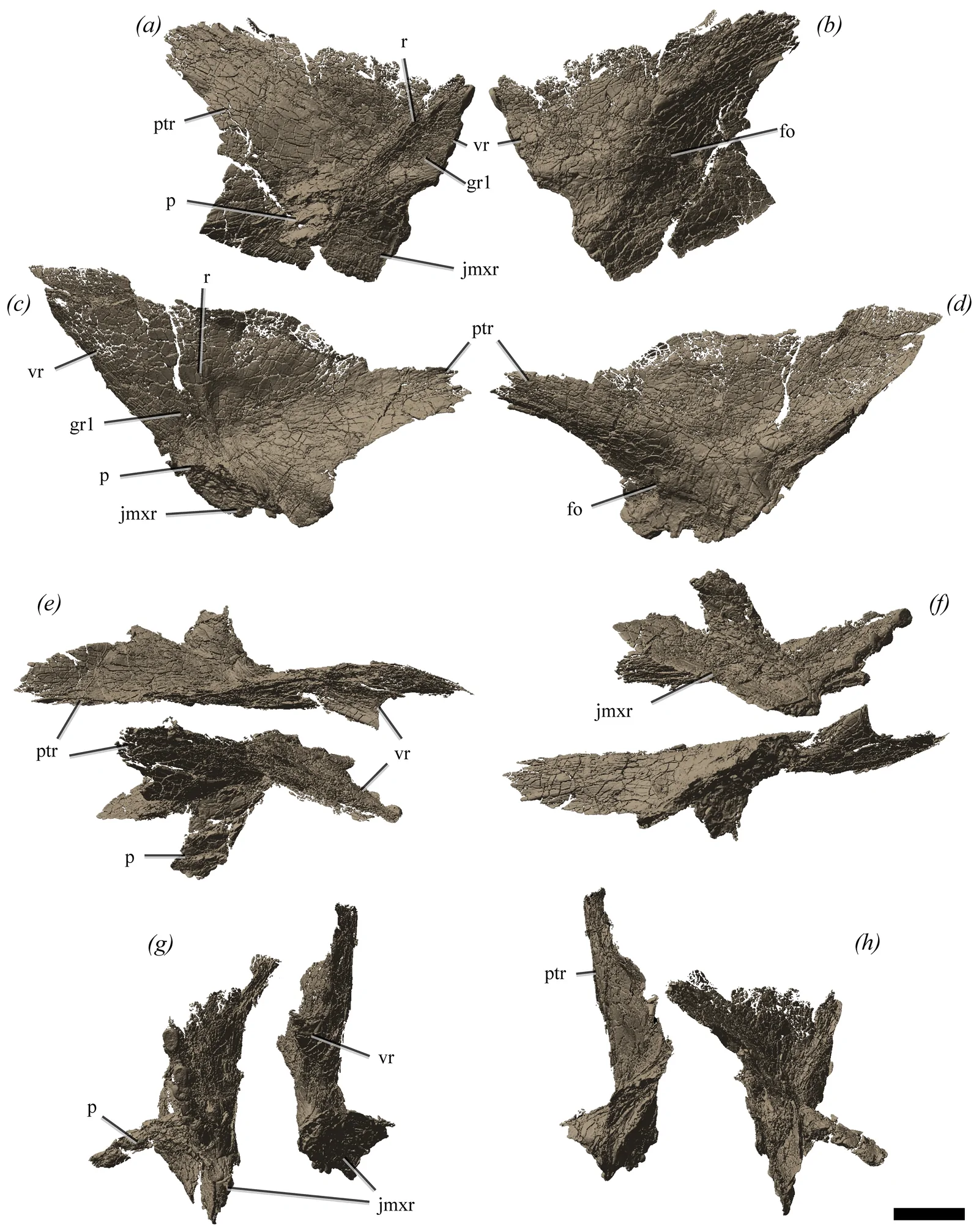
Reconstructed (*a*) right and (*c*) left palatines of BP/1/8468. (*a,c*) Lateral, (*b,d*) medial, (*e*) dorsal, (*f*) ventral, (*g*) anterior and (*h*) posterior views. Abbreviations: fo, foramen; gr, groove; jmxr, jugomaxillary ramus; p, peg; ptr, pterygoid ramus; r, ridge; vr, vomerine ramus. Scale bar = 8 mm.

The vomerine ramus extends anterodorsally, and its anterior margin is angled at 30° from its vertical axis. A web of bone connects the anterodorsal margin of the vomerine ramus to the posteriormost margin of the pterygoid ramus. The two lateral ridges defining a shallow groove described in *Massospondylus* by Chapelle & Choiniere [21] are seen at the base of the vomerine ramus on both palatines. The posteriorly located groove (figure 17*a*,*c*) is distinct and laterally extending. On the anteromedial surface of the left vomerine ramus, located at mid-height, is a medially curving lamina (figure 17*e*). It is unclear whether this is incomplete or distorted, but this feature is not observed in other EBSMs such as *Massospondylus* or *Plateosaurus*.

The jugomaxillary ramus forms the ventrolateral surface of the palatine and is located between the vomerine and pterygoid rami. It is a vertically oriented sheet. A peg-like process extends laterally from the lateral surface of the ramus and has a rounded distal margin. It is angled anterodorsally to posteroventrally in lateral view and is directed posteriorly. A salient feature of the palatine is the presence of a foramen that exits posteriorly on the medial surface of the ramus, whereas *Massospondylus* and *Ngwevu* lack this feature. On its ventral surface is a ventrally opening, anteroposteriorly oriented fossa.

The pterygoid ramus is a subhorizontal sheet that extends posteriorly, and its lateral margin flares laterally. Its medial surface is concave, and the lateral surface is convex with a smooth surface.

### 5.3. Neurocranium

The neurocranium of *Nyana* (figures 18 and 19) consists of the frontals, parietals and the braincase, the latter consisting of the basioccipital, parabasisphenoid, orbitosphenoids, laterosphenoids, prootics, otoccipitals and supraoccipital. All elements are preserved close to their life positions. Sutures between elements are unfused except in the otoccipitals (exoccipital and opisthotic) and parabasisphenoid (basisphenoid and parasphenoid).

**Figure 18.**
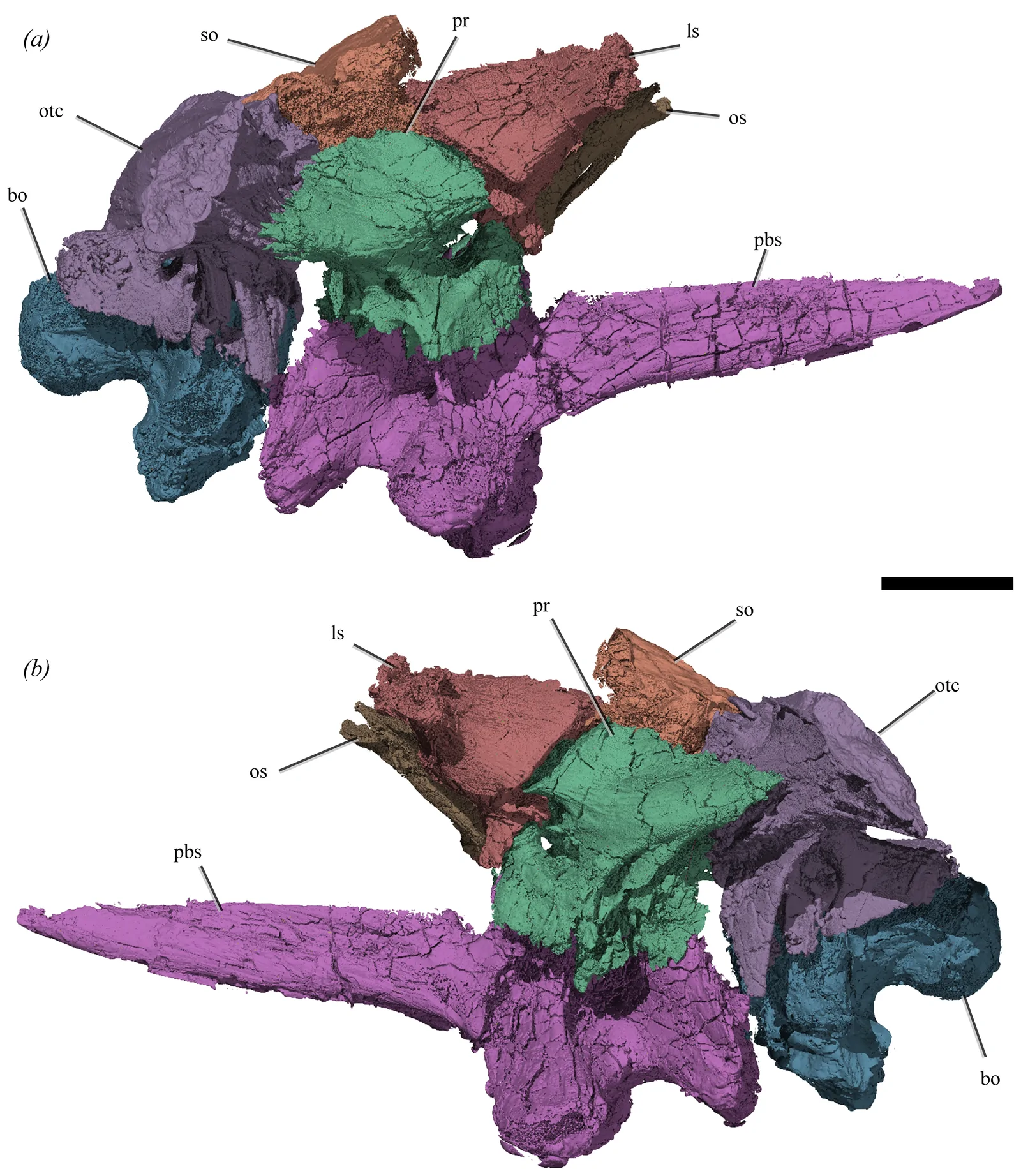
Reconstructed braincase of BP/1/8468. (*a*) Right lateral and (*b*) left lateral views. Abbreviations: bo, basioccipital; ls, laterosphenoid; os, orbitosphenoid; otc, otoccipital; pbs, parabasisphenoid; pr, prootic; so, supraoccipital. Scale bar = 15 mm.

**Figure 19.**
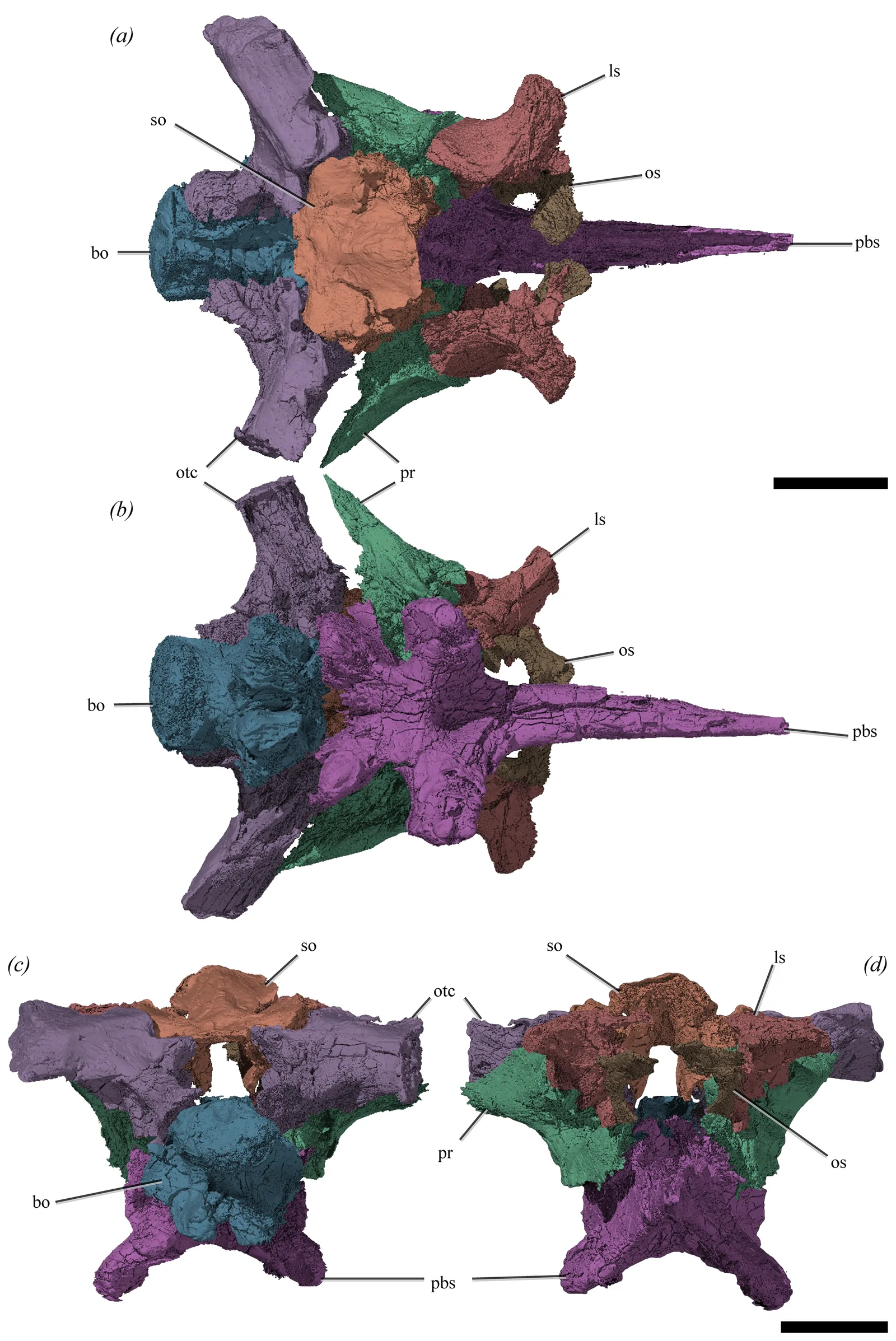
Reconstructed braincase of BP/1/8468. (*a*) Dorsal, (*b*) ventral, (*c*) posterior, and (*d*) anterior views. Abbreviations: bo, basioccipital; ls, laterosphenoid; os, orbitosphenoid; otc, otoccipital; pbs, parabasisphenoid; pr, prootic; so, supraoccipital. Scale bars = 20 mm.

#### 5.3.1. Frontal

The frontals are partially preserved, lacking their anterior portions, unfused and are in life position (figure 20). The frontal consists of the main body, a prefrontal ramus and a postorbital ramus. As preserved, it contacts the postorbital posterolaterally, the prefrontal anterodorsally and the parietal posteriorly. The midline symphysis is an interdigitating contact. The dorsal and ventral surfaces (figure 20*a*,*b*) of the frontal are smooth, and the articular surfaces for the parietals are rugose (figure 20*f*).

**Figure 20.**
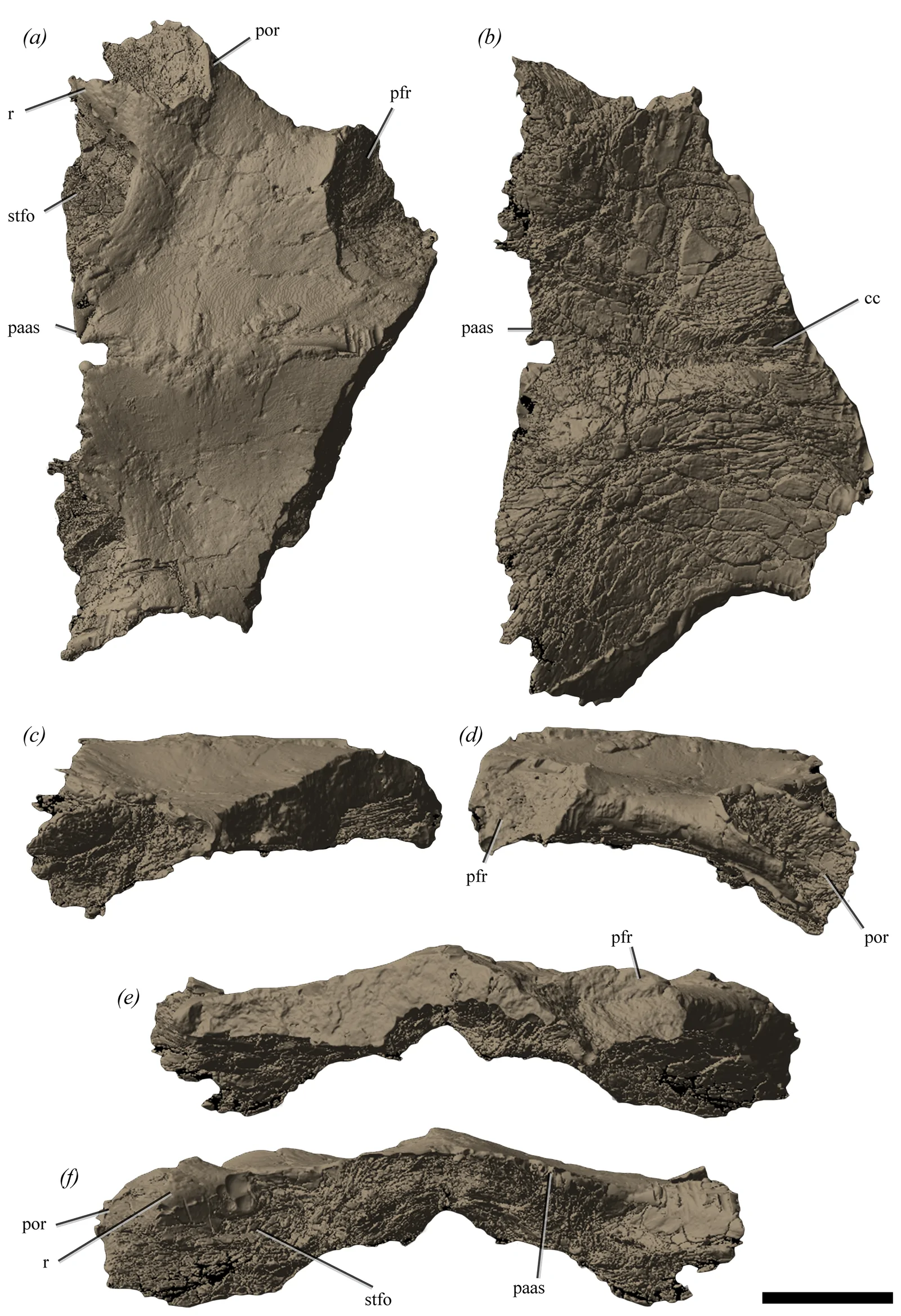
Reconstructed left and right frontals of BP/1/8468. (*a*) Dorsal, (*b*) ventral, (*c*) right lateral, (*d*) left lateral, (*e*) anterior and (*f*) posterior views. Abbreviations: cc, cerebral cavity; paas, parietal articular surface; pfr, prefrontal ramus; por, postorbital ramus; r, ridge; stfo, supratemporal fossa. Scale bar = 15 mm.

The anteroposterior axis of the frontal lies in the same plane as the main axis of the skull. The dorsal surface is flat, unlike that of *Massospondylus* where the region contributing to the orbital rim arcs dorsally. There is no midline crest. In *Nyana*, the frontal lacks the midline boss located on the dorsal surface in *Yunnanosaurus*. The lateral margin of the frontal contributes to the dorsal margin of the orbit. The combined ventral surfaces of the frontals bear an hourglass-shaped depression that extends anteroposteriorly along the midline, an impression of the endocranial cavity. This depression continues posteriorly onto the parietal. The longitudinal ridges on the ventral surface of the frontal that usually separate the orbital and endocranial cavities (the crista cranii) are absent in BP/1/8468.

The prefrontal ramus extends from the anterolateral surface of the frontal. It bears an ovoid articular surface for the prefrontal that is directed dorsally and is defined by distinct ridges. It is mediolaterally wider than it is dorsoventrally tall. The ramus appears to extend strictly laterally, similar to that seen in *Sarahsaurus*, but differing from the arched prefrontal ramus seen in *Massospondylus*.

The postorbital ramus extends laterally from the posterolateral corner of the frontal. It is triangular in dorsal view. The postorbital ramus is dorsoventrally thicker than the main body of the frontal, similar to *Ngwevu*, but differing from the dorsoventrally thinner ramus seen in other EBSMs. The ventral margin of the postorbital ramus slopes ventrally. It bears an anterolateral recess for the articulation of the postorbital. This recess is anteroposteriorly long and mediolaterally deep.

Medial to the postorbital ramus, a supratemporal fossa excavates the dorsal surface of the frontal, to form an anteroposteriorly long, mediolaterally wide recess. This recess is triangular in outline with its apex directed anteriorly. It is separated from the postorbital ramus by a thin ridge. The supratemporal fossa also extends onto the medial margin of the postorbital and the anterolateral margin of the parietal. A similar supratemporal fossa is present in *Macrocollum*, *Plateosaurus*, *Adeopapposaurus*, *Leyesaurus*, *Lufengosaurus*, *Jingshanosaurus*, *Ngwevu* and *Sarahsaurus*, but its presence is unclear in NMQR 3314. The supratemporal fossa is absent in *Yunnanosaurus*. In *Nyana*, the frontal does not contribute to the margin of the supratemporal fenestra, similar to those of *Adeopapposaurus*, *Anchisaurus*, *Jingshanosaurus*, *Lufengosaurus*, *Massospondylus*, *Ngwevu*, *Plateosaurus*, *Yunnanosaurus* and *Riojasaurus*.

#### 5.3.2. Parietal

Both parietals are complete, unfused and preserved close to their life positions (figure 21). In lateral view, the sagittal axis of the parietal is horizontal and aligned with the main axis of the skull. The parietal consists of a main body, a postorbital ramus and a squamosal ramus. It contacts the frontal anteriorly, the postorbital anterolaterally, the squamosal posterolaterally, the supraoccipital posteromedially and the laterosphenoid and prootic anteroventrally. The dorsal surface of the parietals is smooth, whereas the ventral surface is irregular owing to impressions from the underlying endocranial cavity.

**Figure 21.**
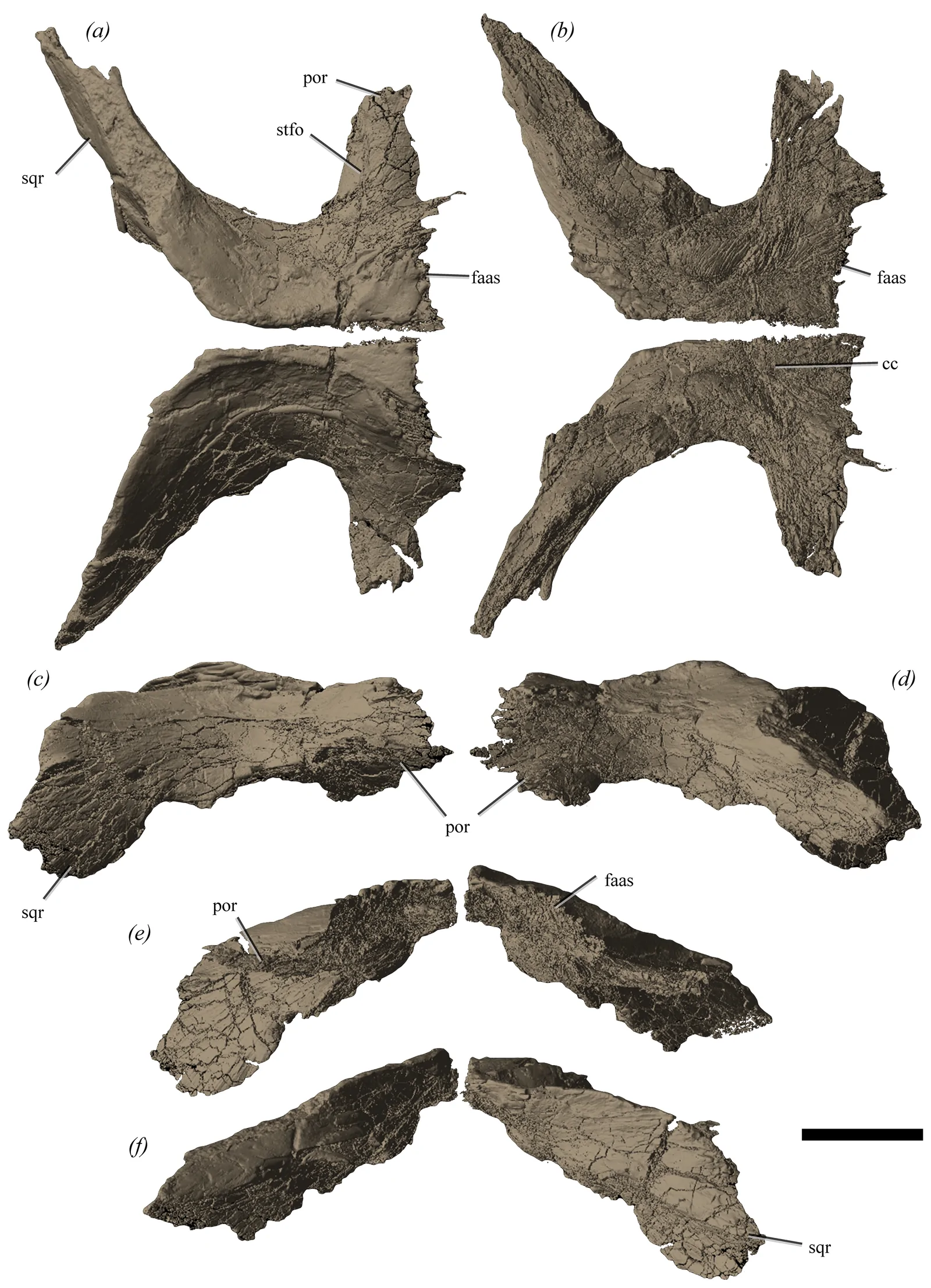
Reconstructed left and right parietals of BP/1/8468. (*a*) Dorsal, (*b*) ventral, (*c*) right lateral, (*d*) left lateral, (*e*) anterior and (*f*) posterior views. Abbreviations: cc, cerebral cavity; faas, frontal articular surface; por, postorbital ramus; sqr, squamosal ramus; stfo, supratemporal fossa. Scale bar = 15 mm.

The main body of the parietal is medially constricted by the supratemporal fenestra, giving the paired parietals an hourglass-shaped outline in dorsal view (figure 21*a*). The anterior contact with the frontal is interdigitating and appears straight in dorsal view. The parietal flares laterally at its anterolateral corner to form the postorbital rami, which do not extend as far laterally as the squamosal rami. A dorsal midline boss is absent, differing from the condition in *Yunnanosaurus*. The supratemporal fossa occupies the anterolateral margins where the parietal, the frontal and postorbital meet.

The postorbital ramus forms the anterior margin of the supratemporal fenestra and extends perpendicularly from the anterolateral corner of the parietal main body. The lateral margins of the main body that form the medial borders of the supratemporal fenestra are concave and slope ventrally. A sagittal crest is absent. The ventral surface (figure 21*b*) is rugose and bears a continuation of the endocranial impression from the frontal, forming an hourglass-shaped depression. Posteriorly, the supraoccipital inserts between the squamosal rami, while the squamosal rami contact the paroccipital processes posterolaterally.

The squamosal ramus is anteroposteriorly short and mediolaterally wide, and dorsoventrally oriented. In lateral view, it slopes ventrally as a thin sheet and terminates in a pointed distal end. Posteriorly, it overlaps the parietal ramus of the squamosal. Medially directed bony ridges extend along much of the medial surface of the squamosal ramus. The anterolateral margin forms the posteromedial border of the supratemporal fenestra. The medial surface is weakly concave, and the lateral surface is flat.

The parietals of *Nyana* resemble those of *Ngwevu*, *Sarahsaurus*, *Yunnanosaurus*, *Lufengosaurus*, *Riojasaurus* and *Adeopapposaurus* in having mediolaterally expanded anterior and posterior margins and an hourglass-shaped dorsal outline. In contrast, *Plateosaurus* and *Massospondylus* possess parietals that have proportionately wider posterior margins and prominent postorbital rami that produce a ‘V’-shaped dorsal outline.

#### 5.3.3. Basioccipital

The basioccipital is complete, well-preserved and undistorted (figure 22). Although slightly displaced, it retains a subhorizontal orientation aligned with the long axis of the skull. It forms most of the occipital condyle, the posterior portions of the basal tubera and the floors of the foramen magnum and posterior endocranial cavity. It contacts the parabasisphenoid anteriorly and the otoccipitals dorsally.

**Figure 22.**
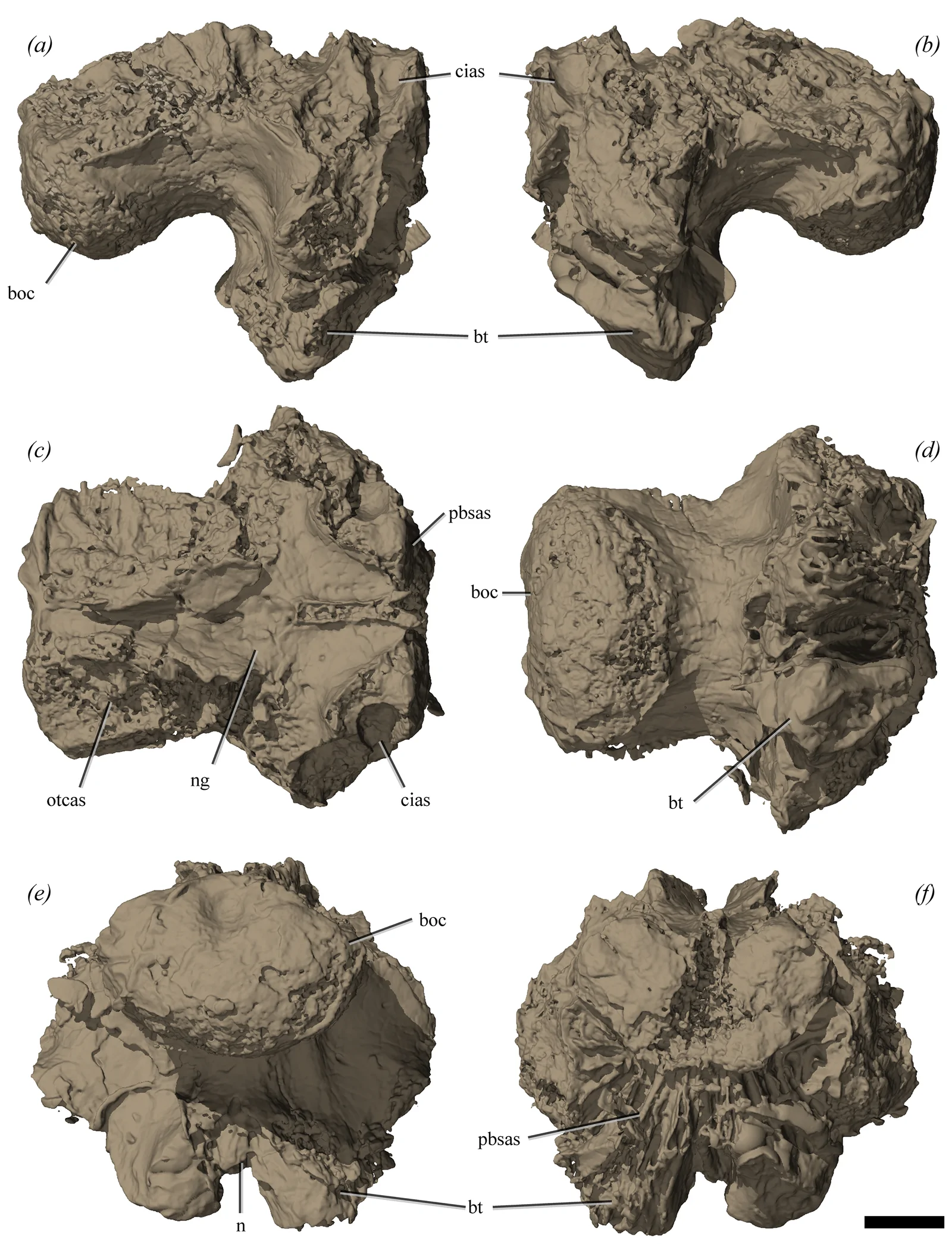
Reconstructed basioccipital of BP/1/8468. (*a*) Right lateral, (*b*) left lateral, (*c*) dorsal, (*d*) ventral, (*e*) posterior and (*f*) anterior views. Abbreviations: cias, crista interfenestralis articular surface; boc, basioccipital condyle; bt, basal tubera; n, notch; ng, neural groove; otcas, otoccipital articular surface; pbsas, parabasisphenoid articular surface. Scale bar = 6 mm.

The occipital condyle projects posteriorly and is separated from the basal tubera by a distinct neck with a mediolaterally concave ventral surface. A shallow midline trough is present on the posterodorsal surface of the condyle. The condylar neck is proportionately shorter anteroposteriorly than in *Macrocollum* and *Massospondylus* but comparable in length to those of *Unaysaurus*, *Sarahsaurus*, *Coloradisaurus*, *Lufengosaurus* and *Ngwevu*. In lateral view (figure 18), the ventral margin of the occipital condyle lies at the same level as the distal tip of the cultriform process. The condyle is mediolaterally wider than the neck in ventral view, and in posterior view, it has a reniform outline.

The basioccipital condyle contributes to the posterior halves of the basal tubera, which it forms equally with the parabasisphenoid. Its contribution extends ventrally from the basioccipital body, and the paired tubera are separated by a ventral ‘U’-shaped trough. This differs from *Efraasia* and NMQR 3314, which lack separation of the basal tubera. A trough arising from this notch extends anteriorly along the ventral margin of the basioccipital and continues onto the parabasisphenoid, widening mediolaterally towards the anterior end. The basal tubera are directed posteroventrally in lateral view. The articular surface for the parabasisphenoid is weakly convex, rugose and interdigiting. The basioccipital–parabasisphenoid suture is angled anteroventrally.

The neural groove forming the floor of the foramen magnum (figure 22*c*) is mediolaterally narrow posteriorly and widens anteriorly, separating the otoccipitals. The basioccipital–otoccipital contact is an irregular butt joint oriented dorsolaterally with a slight anterior inclination.

#### 5.3.4. Basisphenoid–parasphenoid

The fused parabasisphenoid is complete and well-preserved (figures 23–25). Minor distortion has displaced the cultriform process slightly to the left. The parabasisphenoid is composed of a main body, the anterior components of the basal tubera, the basipterygoid processes and the cultriform process (figure 23). It contacts the basioccipital posteriorly, the prootic dorsally and the pterygoid ventrally, although the latter articulation is poorly preserved.

**Figure 23.**
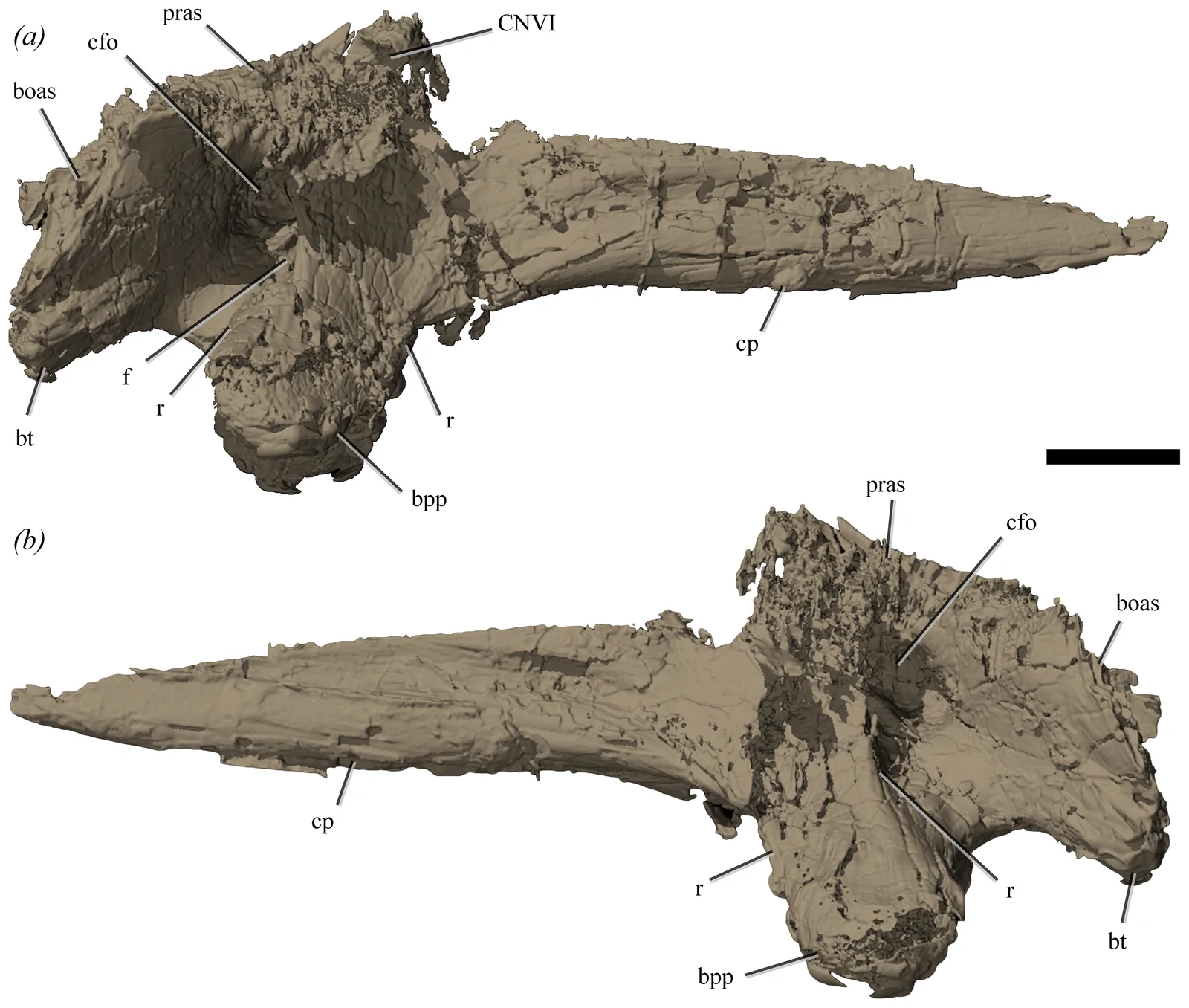
Reconstructed parabasisphenoid of BP/1/8468. (*a*) Right lateral and (*b*) left lateral views. Abbreviations: boas, basioccipital articular surface; bpp, basipterygoid processes; bt, basal tubera; cfo, carotid foramen; CNVI, cranial nerve passage; cp, cultriform process; f, fossa; pras, prootic articular surface; r, ridge. Scale bar = 10 mm.

**Figure 24.**
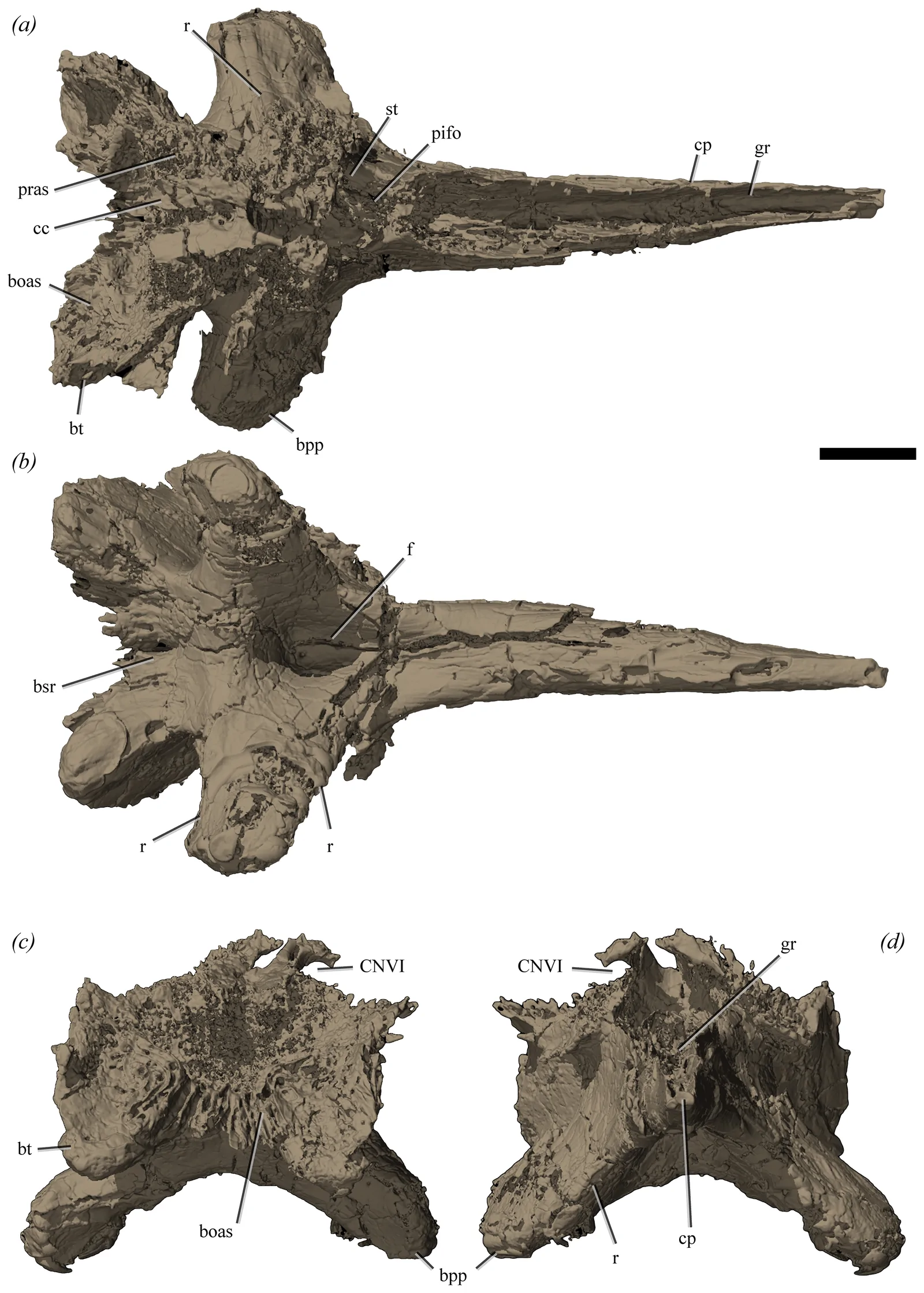
Reconstructed parabasisphenoid of BP/1/8468. (*a*) Dorsal, (*b*) ventral, (*c*) posterior and (*d*) anterior views. Abbreviations: boas, basioccipital articular surface; bpp, basipterygoid processes; bsr, basisphenoid recess; bt, basal tubera; cc, cranial cavity; CNVI, cranial nerve passage; cp, cultriform process; f, fossa; gr, groove; pifo, pituitary fossa; pras, prootic articular surface; r, ridge; st, sella turcica. Scale bar = 10 mm.

**Figure 25.**
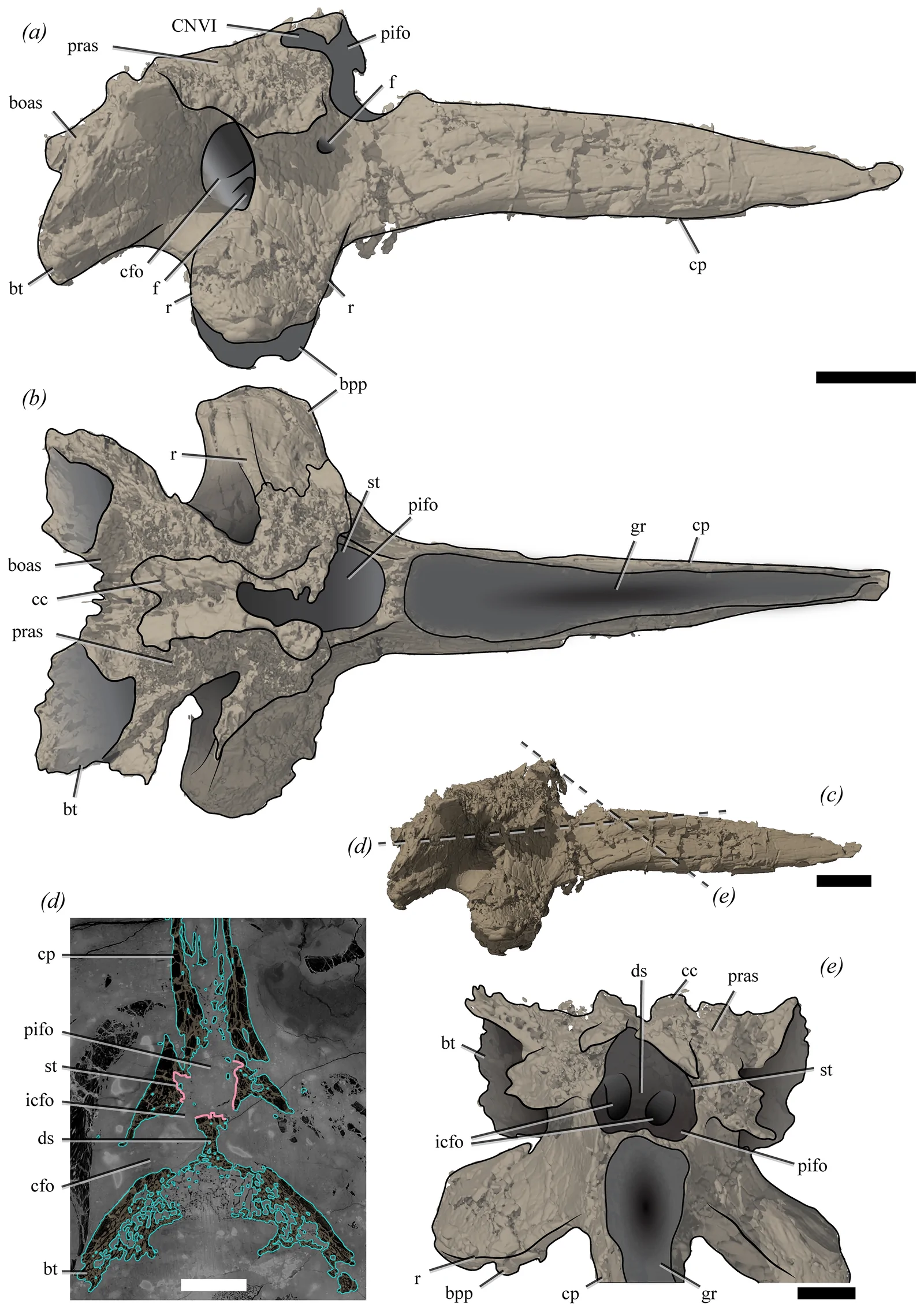
Simple schematics showing the internal structures of the reconstructed parabasisphenoid of BP/1/8468. (*a*) Right lateral and (*b*) dorsal views. (*c*) Right lateral view with dashed lines indicating cross-sections and views. (*d*) Transverse cross-section of the parabasisphenoid showing the internal structures. The pink lines resemble the sella turcica. (*e*) A posteroventral view of the structures within the pituitary fossa. Abbreviations: boas, basioccipital articular surface; bpp, basipterygoid processes; bt, basal tubera; cc, cranial cavity; cfo, carotid foramen; CN, cranial nerve passage; cp, cultriform process; ds, dorsum sellae; f, fossa; gr, groove; icfo, intercarotid foramen; pifo, pituitary fossa; pras, prootic articular surface; r, ridge; st, sella turcica. Scale bars: (*a, b, c*) = 10 mm, (*d*) = 8 mm, (*e*) = 6 mm.

The dorsal surface of the main body forms the anterior floor of the endocranial cavity (figure 24). A deep, circular pituitary fossa occupies its anterior region and is directed anterodorsally, positioned between the base of the cultriform process and the parabasisphenoid–prootic contact. The sella turcica forms the lateral and posterior walls of the pituitary fossa, while the dorsum sellae divides the posterior wall along its midline separating the two internal carotid foramina (figure 25*d*). Dorsal to the pituitary fossa, paired anteriorly directed projections are separated by a narrow, ‘U’-shaped gap along the midline. Small openings on the lateral sides represent the exit for the abducens nerve (CNVI). These openings are infrequently preserved but have been identified in *Plateosaurus*. A shallow, rectangular basisphenoid recess occupies the posteroventral surface and continues onto the ventral surface of the basioccipital and the basal tubera. This recess is bounded anteriorly by a web of bone connecting the medial sides of the bases of the basipterygoid processes. Dorsoventrally elongated elliptical cavities on the lateral surfaces of the main body, between the basipterygoid processes and basal tubera, house the carotid foramen, which open into canals entering the sella turcica posteriorly (figure 25*d*,*e*). A small accessory foramen of uncertain function is present on the right lateral surface of the parabasisphenoid, ventral to the carotid foramen. The parabasisphenoid–prootic contact is a horizontally oriented butt joint with an irregular articular surface.

The parabasisphenoid forms the anterior halves of the basal tubera. Together with their basioccipital component, the tubera are smooth, rounded protuberances directed posteriorly and ventrolaterally. Their morphology resembles those seen in *Adeopapposaurus* and *Leyesaurus* but differs from the tetrahedral protuberances present in *Massospondylus*. The lateral surfaces of the basal tubera are convex and smooth. The irregular posterior surface of the parabasisphenoid is angled posterodorsally, is concave and complements the articular surface of the basioccipital.

In lateral view (figure 23), the basipterygoid processes are angled posteriorly and are directed ventrolaterally in posterior view, differing from the anteriorly directed basipterygoid processes of *Efraasia* and *Sarahsaurus*, and the ventrally directed processes of *Massospondylus* and *Plateosaurus*. The basipterygoid processes are mediolaterally compressed, ovoid in transverse cross-section, and terminate in convex distal ends. In posterior view, they diverge at approximately 120°, a greater angle than in *Massospondylus* (30°) and *Ngwevu* (91°), but comparable to that in *Adeopapposaurus* (100°). The basipterygoid processes are dorsoventrally longer than the basal tubera, as seen in many EBSMs (e.g. *Unaysaurus*, *Sarahsaurus*, *Plateosaurus*, *Efraasia*, *Coloradisaurus*, *Adeopapposaurus*, NMQR 3314, *Ngwevu* and *Massospondylus*). A ridge extends along the anterior margin of each basipterygoid process and grades into the ventral margin of the cultriform process. A lamina is present on the posterolateral surface of each basipterygoid process that extends dorsally along the lateral surface of the basisphenoid body and forms the anterior border of the cavity containing the carotid foramen. The ventral surfaces of the basipterygoid processes are smooth. In lateral view, the basipterygoid processes are well-separated from the basal tubera by a wide, posterodorsally angled, ‘U’-shaped depression. *Plateosaurus*, and possibly *Coloradisaurus*, have a ‘stepped’ braincase where, in lateral view, the basioccipital condyle lies dorsal to the cultriform process, but in *Nyana*, the basioccipital condyle is aligned with the cultriform process in lateral view.

The cultriform process tapers anteriorly and is similar in anteroposterior length to the combined anteroposterior length of the basioccipital and parabasisphenoid main bodies, similar to the condition in *Adeopapposaurus*. This differs from *Massospondylus*, in which it is shorter than its combined anteroposterior length, and *Plateosaurus*, in which it is longer than its combined anteroposterior length. In lateral view, the cultriform process is slightly dorsally inclined with a straight ventral margin and a convex dorsal margin. The dorsal surface of the cultriform process has a deep longitudinal groove resulting in a ‘V’-shaped cross-section with its apex directed ventrally. This deep groove is laterally bounded by thin, tall laminae. The cultriform process is separated from the pituitary fossa by a dorsoventrally low wall of bone. The proximal end of the ventral surface of the cultriform process bears a deep, circular fossa that shallowly grades into the cultriform process as it extends anteriorly.

#### 5.3.5. Orbitosphenoid

Both orbitosphenoids are preserved, but they are incomplete, unfused and displaced from their life positions (figure 26). They are mediolaterally thin, smooth elements that would have contacted the laterosphenoids posteriorly, although these articulations are unclear. Three processes can be identified on its posterodorsal, ventral and anterior margins (p1, p2 and p3, respectively). Orbitosphenoids are rarely preserved in EBSMs, limiting comparisons.

**Figure 26.**
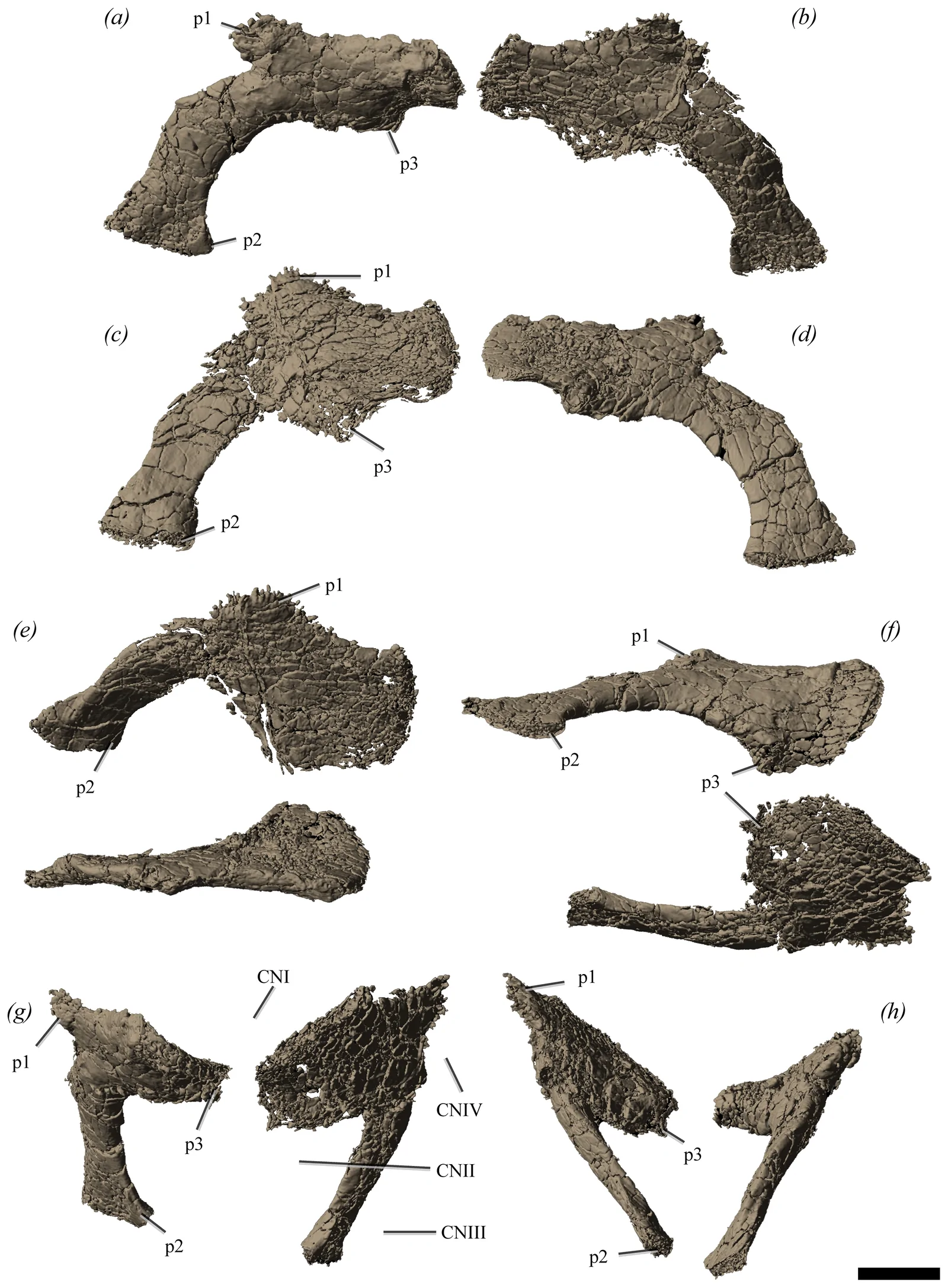
Reconstructed right and left orbitosphenoids of BP/1/8468. (*a,b*) Lateral, (*c,d*) medial, (*e*) dorsal, (*f*) ventral, (*g*) anterior and (*h*) posterior views. CNVIII and CNIV are formed together with the laterosphenoids, and their positions are indicated here. Abbreviations: CN, cranial nerve passages; p, process. Scale bar = 4.5 mm.

The dorsal process (p1) extends posteriorly and is angled dorsolaterally in anterior view. It is short mediolaterally and rectangular in lateral view. This process and the dorsal margin of the orbitosphenoids contribute to the anterior margin of the opening for the olfactory nerve (CNI).

The ventral process (p2) is rectangular with a flat ventral margin in medial view. This differs from the tab-like structure seen in *Massospondylus*. This process is directed posteromedially, angled towards each other and possibly contacted the anteroventral margin of the laterosphenoid.

The anterior process (p3) is located on the ventromedial margin of the orbitosphenoid. It is directed ventromedially as a pointed process. This process contacts its antimere on the midline. The passage created between the anterior process and the ventral process forms the exit for the optic nerve (CNII).

Notably, the main body of the orbitosphenoids is ‘C’-shaped in lateral view (figure 26*a*,*c*), similar to the condition in *Ngwevu* but differing from the ‘S’-shaped condition of *Massospondylus*. The dorsal portion is anteroposteriorly longer than its dorsoventral height, and the ventral portion is dorsoventrally taller than its anteroposterior length. In anterior view, the orbitosphenoids are angled laterally from the skull midline.

#### 5.3.6. Laterosphenoid

Both laterosphenoids are complete, well-preserved and undistorted (figures 27 and 28). They are unfused, and the right laterosphenoid is slightly displaced from its life position. The laterosphenoid is composed of an anteromedial frontal ramus, an anterior orbitosphenoid ramus, a lateral postorbital ramus, a posterior supraoccipital ramus and a ventral prootic ramus. The supraoccipital ramus is not well-preserved. The laterosphenoid contacts the frontals along its anterodorsal margin, the orbitosphenoid anteriorly, the postorbital anterolaterally, the parietal dorsally, the supraoccipital posteromedially and the prootic posteriorly. The laterosphenoids of *Nyana* do not contact the parabasisphenoid.

**Figure 27.**
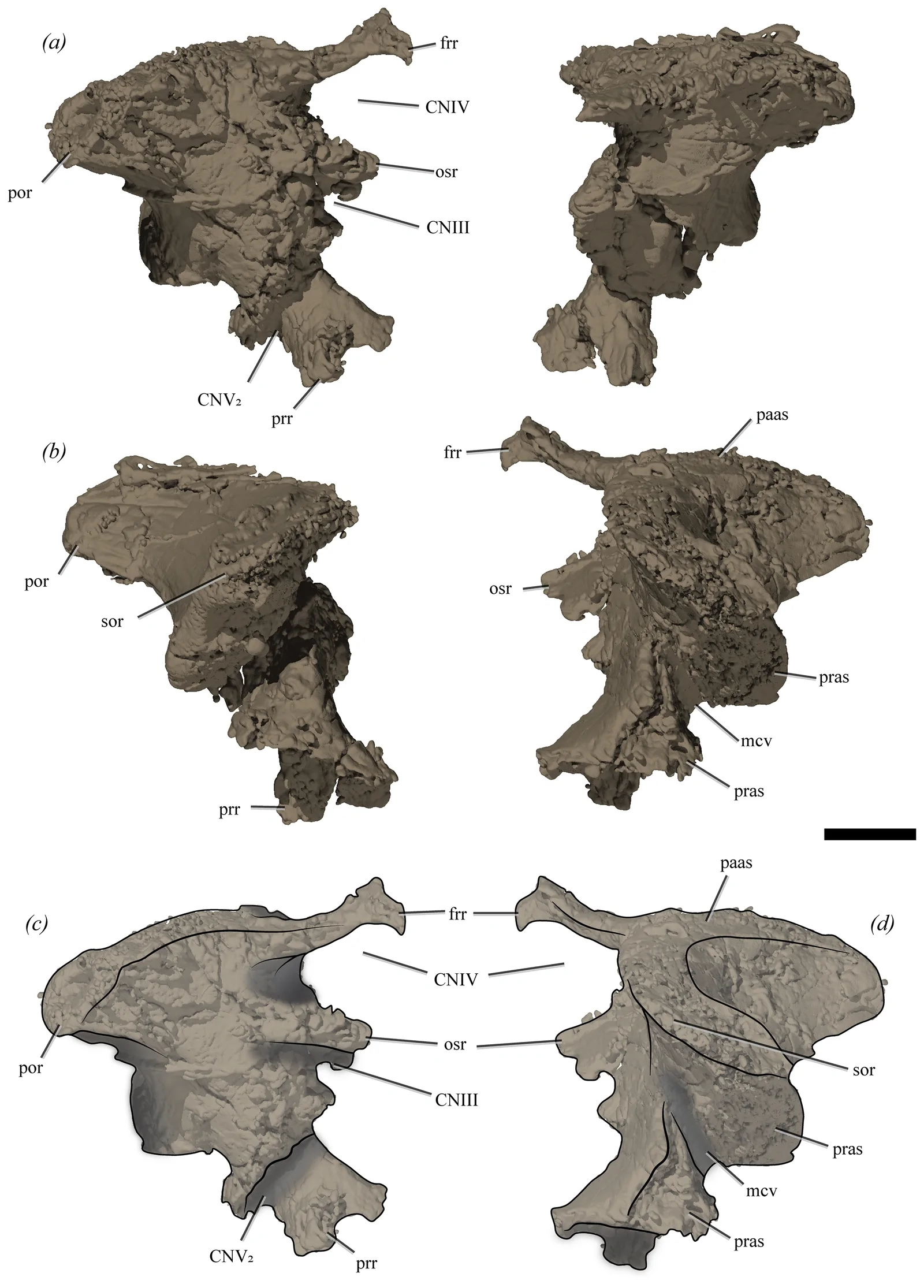
Reconstructed right and left laterosphenoids of BP/1/8468. (*a*) Anterior and (*b*) posterior views. A schematic to show the cranial nerve passages in (*c*) anterior and (*d*) posterior views. Abbreviations: CN, cranial nerve passages; frr, frontal ramus; mcv, mid-cerebral vein; osr, orbitosphenoid ramus; paas, parietal articular surface; por, postorbital ramus; pras, prootic articular surface; prr, prootic ramus; sor, supraoccipital ramus. Scale bar = 6 mm.

**Figure 28.**
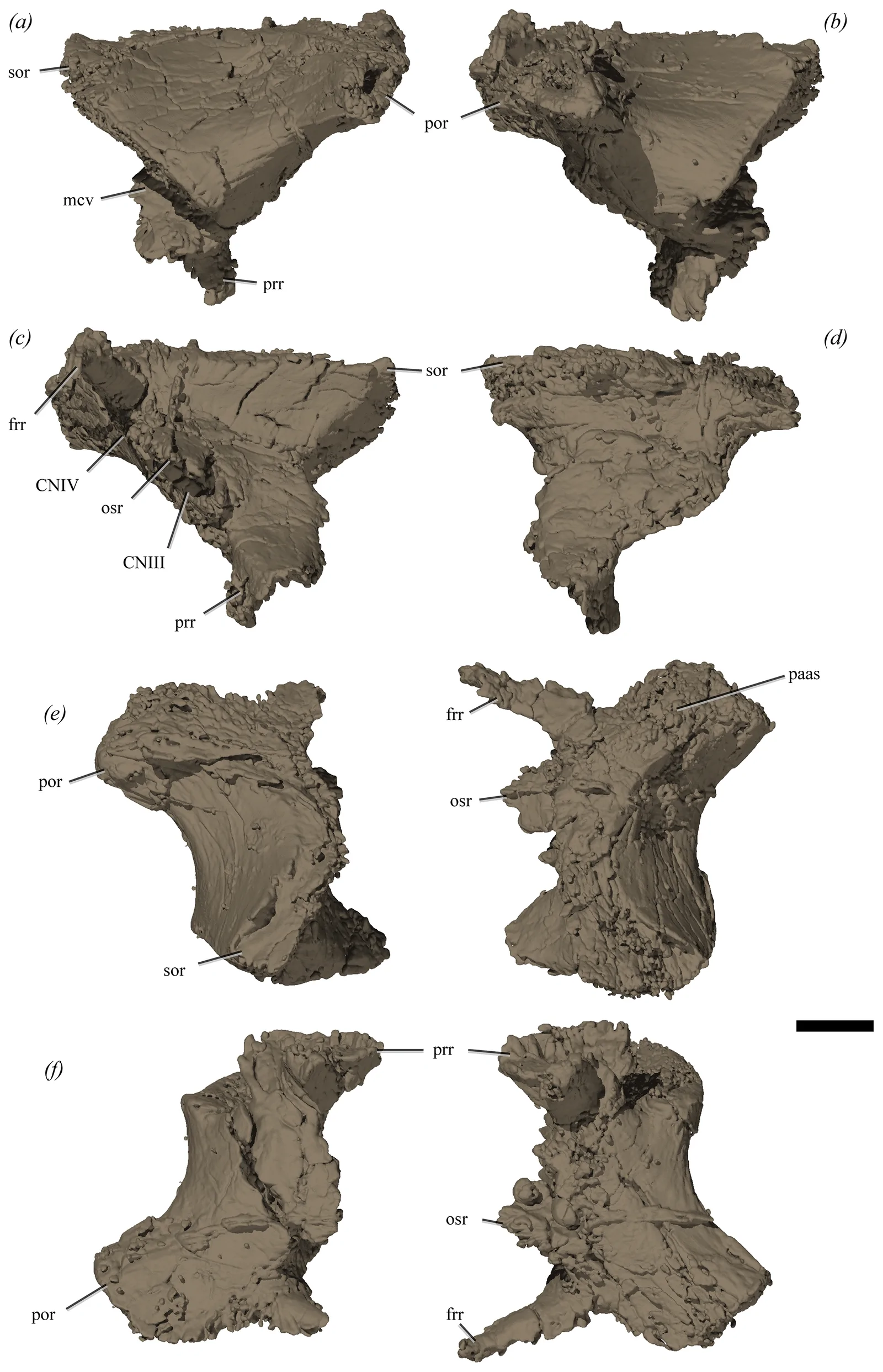
Reconstructed right and left laterosphenoids of BP/1/8468. (*a,b*) Lateral, (*c,d*) medial, (*e*) dorsal and (*f*) ventral views. Abbreviations: CN, cranial nerve passages; frr, frontal ramus; mcv, mid-cerebral vein; osr, orbitosphenoid ramus; paas, parietal articular surface; por, postorbital ramus; prr, prootic ramus; sor, supraoccipital ramus. Scale bar = 6 mm.

The frontal ramus is a thin projection preserved on the anterodorsal margin of the right laterosphenoid. It is directed anteromedially in dorsal view with a pointed distal end (figure 27*a*). The distal end has two small projections, divided into dorsal and ventral projections. The dorsal projection is rounded, and the ventral projection tapers anteroventrally. This ramus contacts the ventromedial surface of the posterior end of the frontal.

The orbitosphenoid ramus is not well-preserved, but the medial portion is present on the right laterosphenoid. The base suggests that it is forked with a dorsal and ventral process that are flattened anteroposteriorly. The orbitosphenoid ramus of *Nyana* extends medially, similar to that of *Ngwevu*, and is directed dorsally, similar to that of *Massospondylus*.

The postorbital ramus flares laterally, directed anterodorsally and rectangular in cross-section. This ramus in *Massospondylus* flares further laterally than in *Nyana* and differs markedly from *Plateosaurus* as the ramus is proportionally anteroposteriorly longer but also curves laterally. The postorbital ramus contacts the postorbital at its anterior margin with a rugose butt joint. It has smooth dorsal, lateral and ventral surfaces. The passage for the ophthalmic branch of the trigeminal nerve (CNV_1_) is not visible in *Nyana*.

The supraoccipital ramus is not well-preserved on the left laterosphenoid, but the proximal portion of this ramus is preserved on the right laterosphenoid. The proximal portion of the ramus suggests it is directed dorsomedially. It has a convex posterior surface, a concave lateral surface and a rugose dorsal surface. This ramus contacts the ventromedial corner of the anterior margin of the supraoccipital.

The prootic ramus is oriented ventrally, directed medially in anterior view and has a rectangular cross-section. Its dorsoventral height is equivalent to approximately one-third of that of the entire laterosphenoid, similar to the condition in *Massospondylus*. The prootic ramus in *Plateosaurus* is distinct and proportionally mediolaterally narrower. The contact between the prootic and the laterosphenoid is a posteroventrally angled butt joint with an irregular surface. The laterosphenoid contacts three regions of the anterior surface of the prootic that are separated by a notch on the anterior margin of the prootic and by a notch on the posterior surface of the laterosphenoid. The laterosphenoid forms the anterior margin of the large, circular trigeminal foramen (CNV), which is located ventral to the opening for the middle cerebral vein (vena cerebralis media). Some EBSMs have a combined foramen for the trigeminal nerve and the mid-cerebral vein, such as *Coloradisaurus* and *Ngwevu*. The passage for the maxillary branch of the trigeminal nerve (CNV_2_) is dorsal to the prootic ramus and is faintly preserved on the lateral surface of the right laterosphenoid.

In *Nyana*, the main body of the laterosphenoid is proportionally mediolaterally wider than those of *Massospondylus* and *Plateosaurus*. The laterosphenoid creates two notches along its anterior surface when in contact with the orbitosphenoid. The dorsal notch is dorsoventrally tallest, bordered posterodorsally by the frontal ramus and anteroventrally by the orbitosphenoid ramus. This notch is the exit for the trochlear nerve (CNIV). The ventral notch is bordered dorsally by the orbitosphenoid ramus and posteroventrally by the prootic ramus. This notch is the passage for the oculomotor nerve (CNIII). The laterosphenoid is triangular in lateral view with its apex directed anterodorsally. Its medial and dorsal surfaces are concave, and its lateral surfaces are convex. The posterodorsal margin of the laterosphenoid contacts and follows the lateral flaring of the parietal. This forms the anteromedial corner of the supratemporal fenestra.

#### 5.3.7. Prootic

Both prootics are well-preserved, complete, unfused and lie near life position (figures 29–31). The prootic contacts the laterosphenoid anterodorsally, the parabasisphenoid ventrally, the parietal dorsally, the supraoccipital posterodorsally and the otoccipitals posteromedially. The prootic can be divided into an elliptical dorsal process and a ventral process that are separated by the foramen for the trigeminal nerve (CNV; figures 30*a* and 31*a*). The contact between the prootic and laterosphenoid includes the entire anterior surface of the prootic but is interrupted by the trigeminal foramen and a dorsal notch for the mid-cerebral vein.

**Figure 29.**
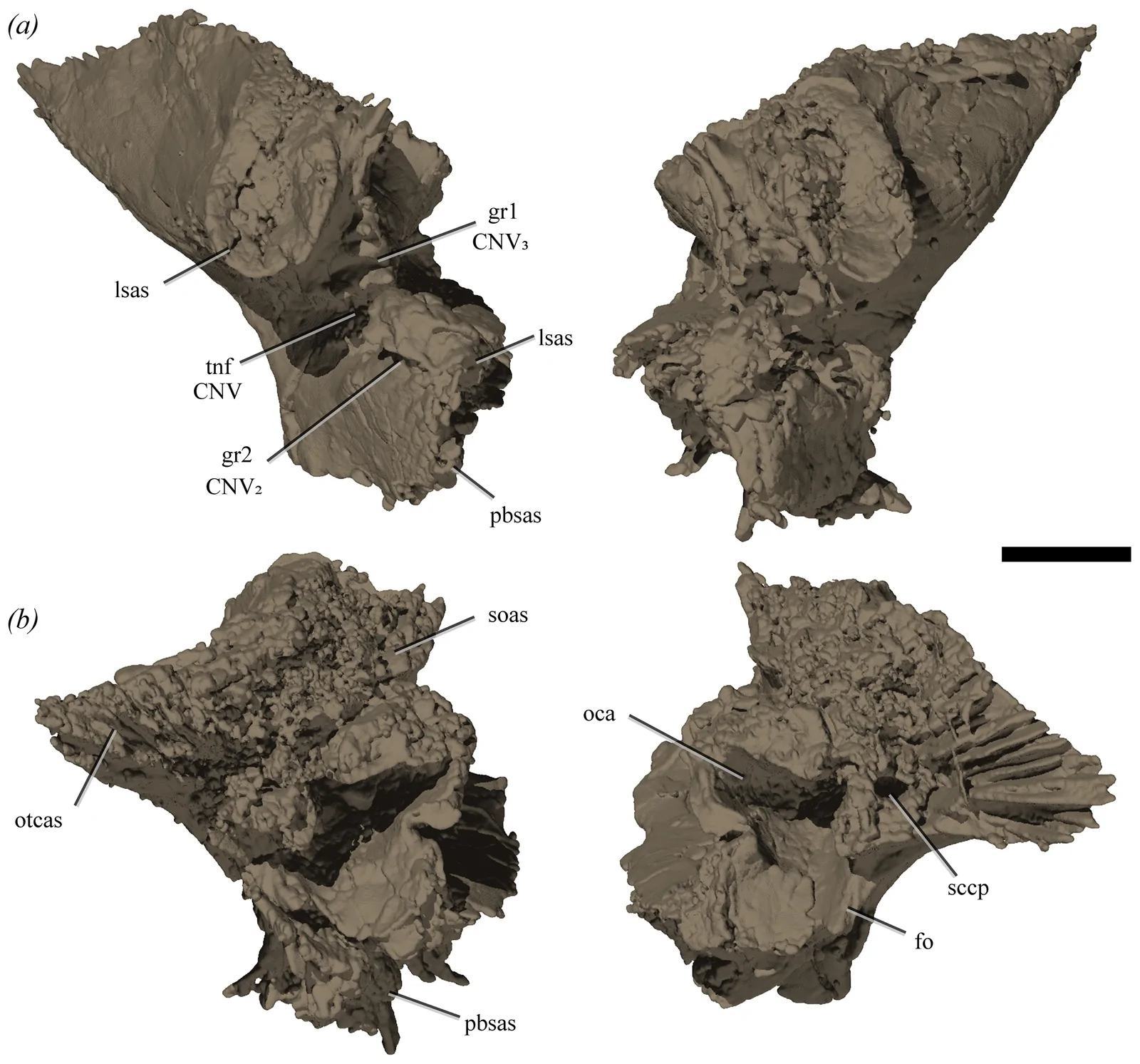
Reconstructed right and left prootics of BP/1/8468. (*a*) Anterior and (*b*) posterior views. Abbreviations: CN, cranial nerve passages; fo, foramen ovale; gr, groove; lsas, laterosphenoid articular surface; oca, otic capsule; otcas, otoccipital articular surface; pbsas, parabasisphenoid articular surface; sccp, semicircular canal passage; soas, supraoccipital articular surface; tnf, trigeminal foramen. Scale bar = 8 mm.

**Figure 30.**
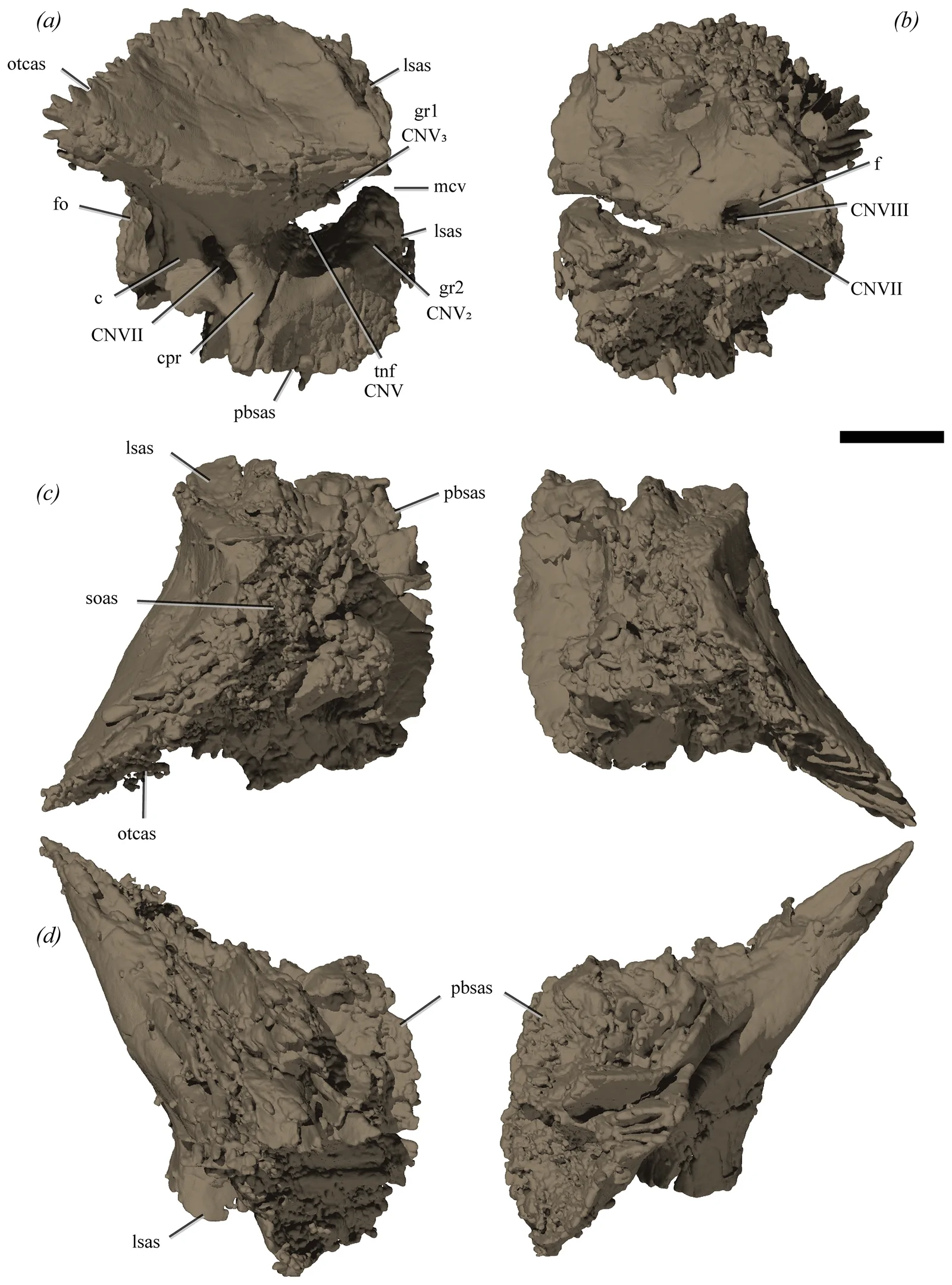
Reconstructed right and left prootics of BP/1/8468. (*a*) Right lateral, (*b*) right medial, (*c*) dorsal and (*d*) ventral views. Abbreviations: c, crista; CN, cranial nerve passages; cpr, crista prootica; f, foramen; fo, foramen ovale; gr, groove; lsas, lateropsphenoid articular surface; mcv, mid-cerebral vein; otcas, otoccipital articular surface; pbsas, parabasisphenoid articular surface; soas, supraoccipital articular surface; tnf, trigeminal foramen. Scale bar = 8 mm.

**Figure 31.**
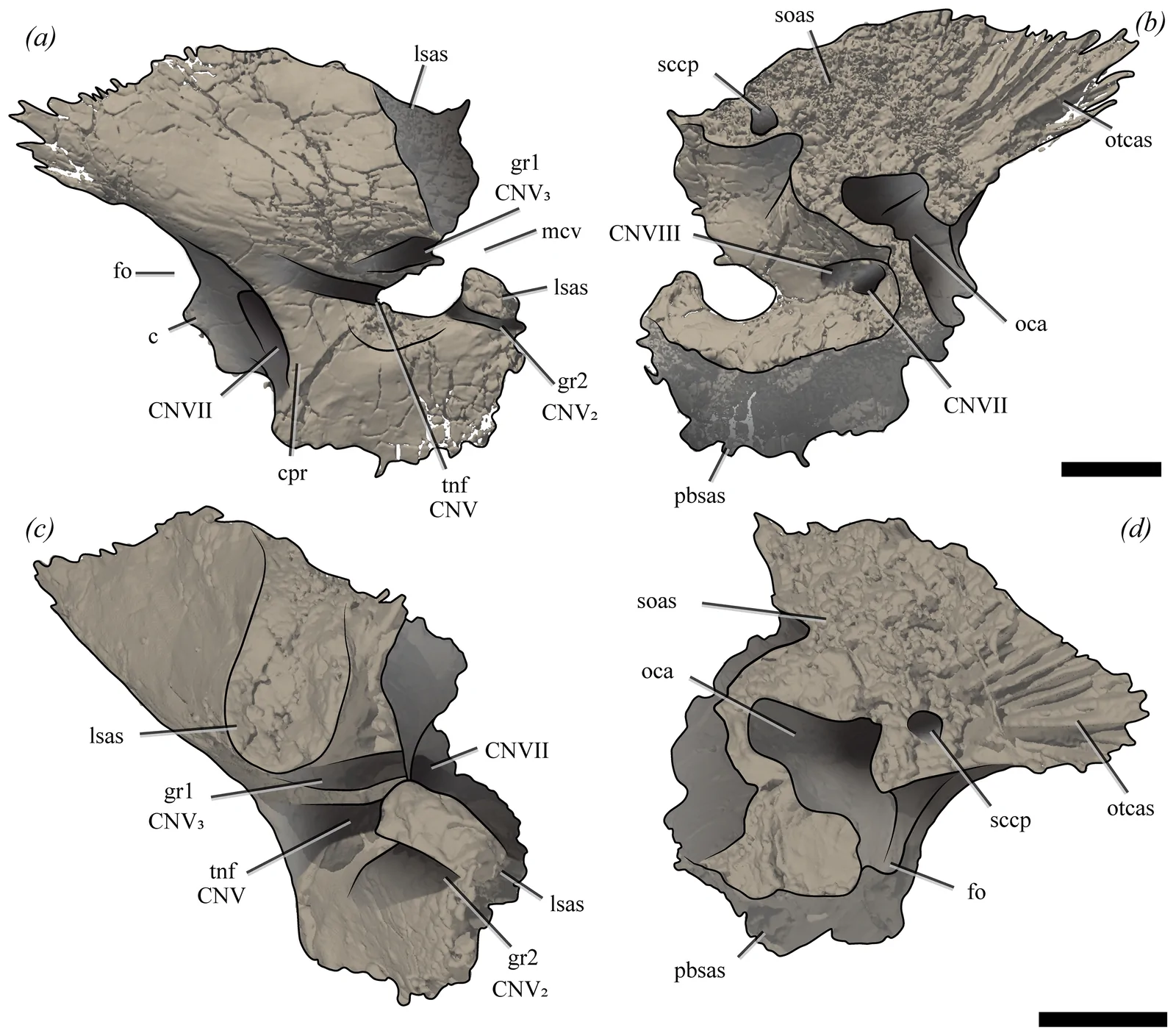
Simple schematics showing the openings of the reconstructed right prootic of BP/1/8468. (*a*) Lateral, (*b*) medial, (*c*) anterior and (*d*) posterior views. Abbreviations: c, crista; CN, cranial nerve passages; cpr, crista prootica; fo, foramen ovale; gr, groove; lsas, laterosphenoid articular surface; mcv, mid-cerebral vein; oca, otic capsule; otcas, otoccipital articular surface; pbsas, parabasisphenoid articular surface; sccp, semicircular canal passage; soas, supraoccipital articular surface; tnf, trigeminal foramen. Scale bars: (*a, b*) = 7 mm, (*c, d*) = 8 mm.

The trigeminal foramen is a large, posteroventrally oriented notch that is surrounded mainly by the prootic. It lies at approximately mid-height on the prootic in lateral view. There are two grooves visible along the margin of the trigeminal foramen (figure 31*a*). A groove located on the posterodorsal (gr1) margin and the second (gr2) along the anteroventral margin of the foramen that continues onto the laterosphenoid. These appear to represent the pathways of the mandibular (CNV_3_) and maxillary branches (CNV_2_), respectively (figure 31*a*,*c*) [91]. The groove for CNV_3_ is not as clearly defined and is located along the posterolateral surface of the trigeminal foramen. The groove for CNV_2_ extends along the anterolateral margin of the prootic and onto the posterolateral margin of the laterosphenoid (see above). The foramen for the facial nerve (CNVII) is located posterior and ventral to the trigeminal opening. The circular CNVII foramen lies within a groove that is defined anteriorly by the crista prootica and posteriorly by a smaller, less defined ridge. This groove is near vertical with a slight anteroventral slope in lateral view, differing from *Massospondylus* and *Ngwevu* in which these grooves have a strong anteroventral slope. In medial view, the facial nerve foramen can be seen opening into the endocranial cavity ventral to the vestibular region.

The prootic encloses the anterior and lateral semicircular canals. In medial view, the anterior canal enters through the anterodorsal surface of the prootic and extends vertically through the bone. The canal expands before exiting the prootic at approximately mid-height at the posterior end of its medial surface. As it expands, the anterior canal joins the sacculus. The lateral semicircular canal extends horizontally through the prootic. The entrance into the inner ear includes the passage for the vestibulocochlear nerve (CNVIII) and is located directly anterodorsally to the facial nerve foramen in medial view.

The lateral surfaces of the dorsal process of the prootic flare laterally with a concave dorsal surface and convex lateral surface. This process flares further laterally than the ventral process in dorsal view. The prootic of *Nyana* is proportionally dorsoventrally shorter than that of *Adeopapposaurus*. The anteroventral surface of the paroccipital processes of the otoccipital contacts the posterior surfaces of the prootic. This contact is rugose, interdigiting and at an angle of 45° relative to the horizontal axis in medial view. The otoccipital articular surface is proportionally anteroposteriorly longer in lateral view than in *Massospondylus*. The contact between the prootic and supraoccipital is mediolaterally angled in posterior view. The ventral process of the prootic is rectangular in lateral view. It contacts the parabasisphenoid dorsally and slightly posteriorly. The contact surface with the parabasisphenoid is irregular and horizontal. This differs from the anteroventrally directed articular surface in *Massospondylus*.

#### 5.3.8. Exoccipital–opisthotic

The exoccipitals are fused indistinguishably to the opisthotics to form otoccipitals. Both elements are nearly complete, well-preserved and in life position (figures 32–34). The distal ends of the paroccipital processes are missing. The otoccipital is composed of the paroccipital processes, contributions to the occipital condyle, the crista interfenestralis and the lateral margins of the foramen magnum. The otoccipital also contains the passage for the posterior semicircular canal of the inner ear and contributes to the border of the foramen ovale for the reception of the stapes. The otoccipital contacts the supraoccipital dorsomedially, the prootic anteriorly, the crista interfenestralis contacts the parabasisphenoid laterally, the basioccipital ventromedially, and the squamosal posterolaterally. The contact between the otoccipital and the basioccipital is a rugose, horizontal butt joint.

**Figure 32.**
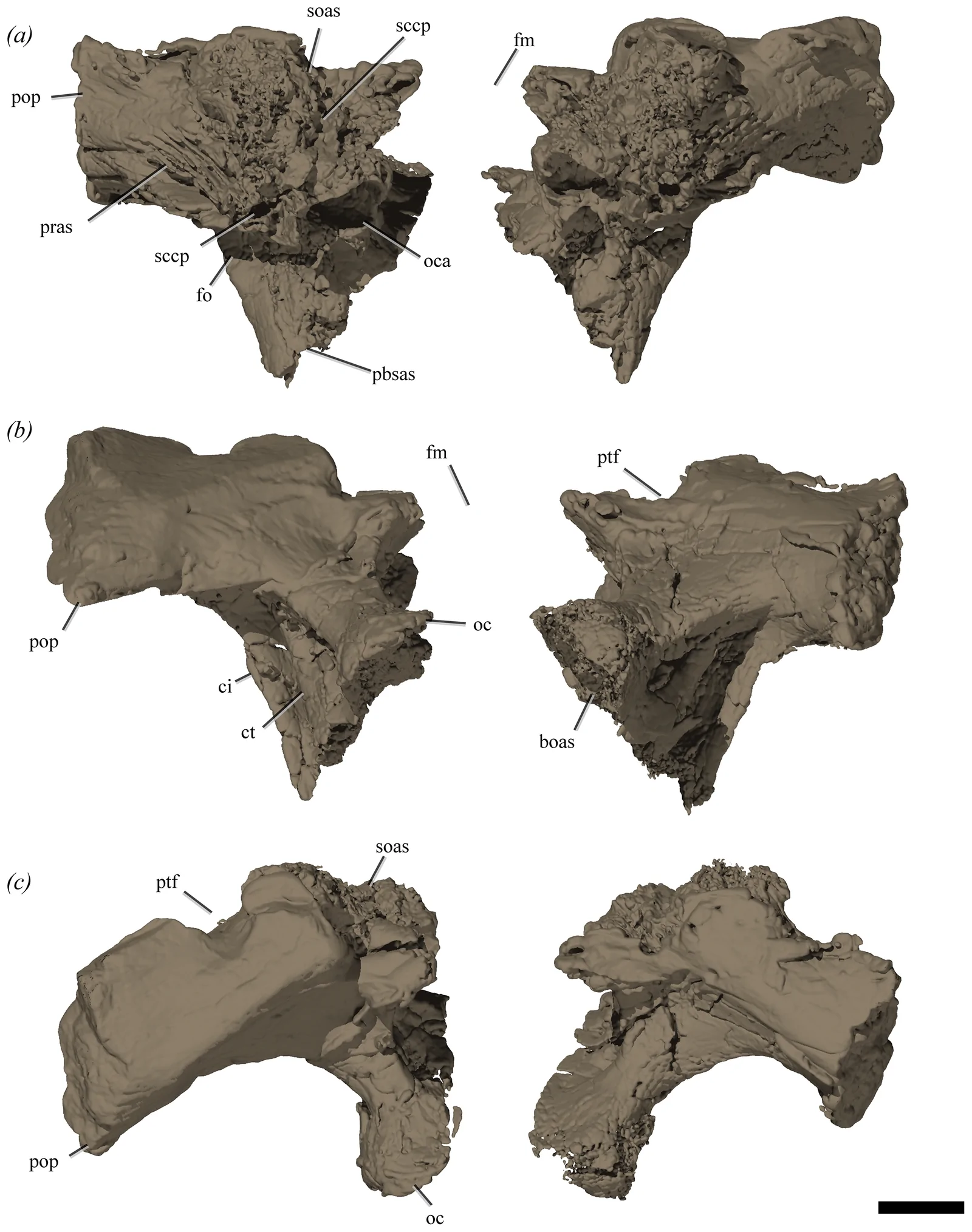
Reconstructed right and left otoccipitals of BP/1/8468. (*a*) Anterior, (*b*) posterior and (*c*) dorsal views. Abbreviations: boas, basioccipital articular surface; ci, crista interfenestralis; ct, crista tuberalis; fm, foramen magnum; fo, foramen ovale; oc, occipital condyle; oca, otic capsule; pbsas, parabasisphenoid articular surface; pop, paroccipital process; pras, prootic articular surface; ptf, post-temporal fenestra; sccp, semicircular canal passage; soas, supraoccipital articular surface. Scale bar = 8 mm.

**Figure 33.**
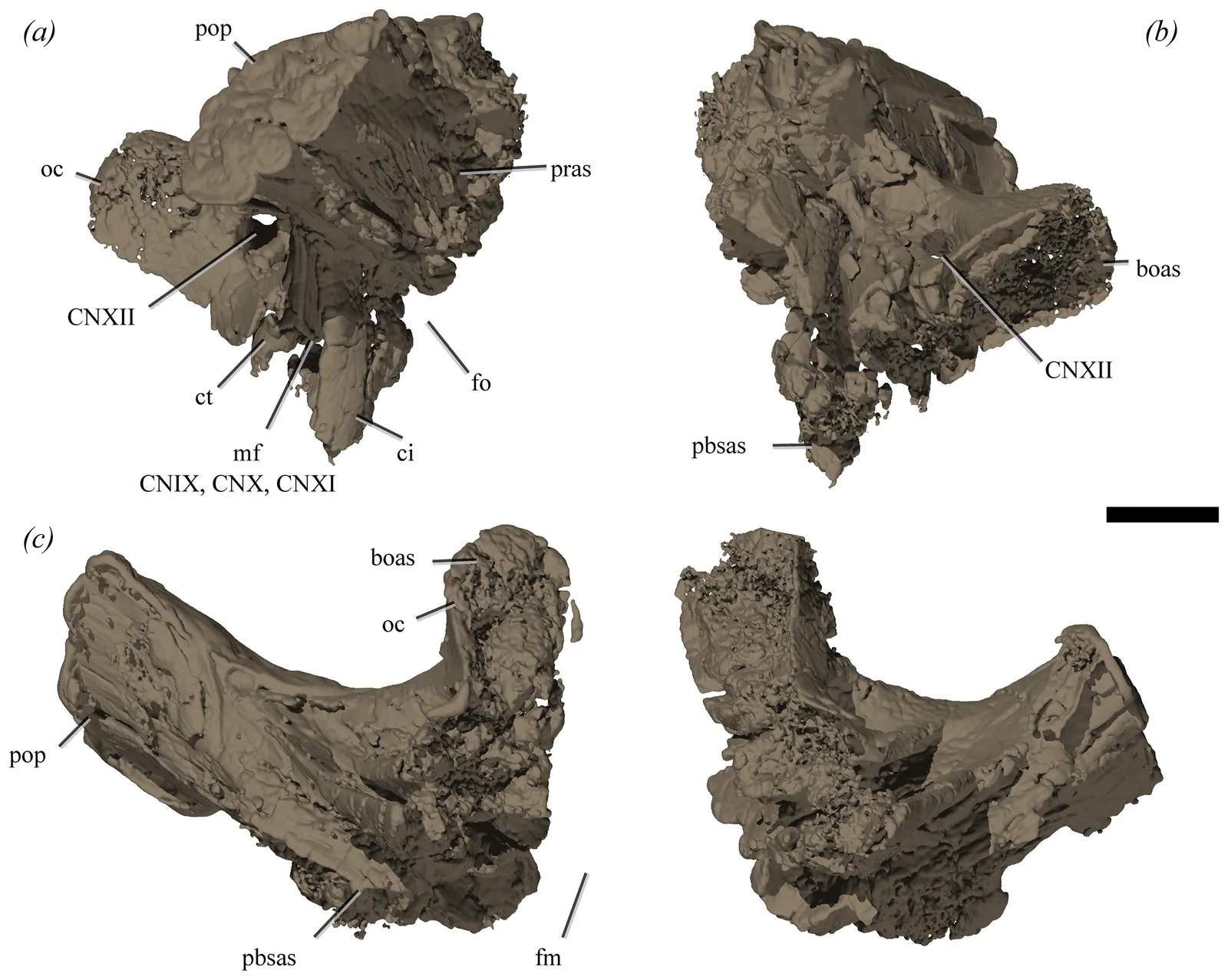
Reconstructed right and left otoccipitals of BP/1/8468. (*a*) Right lateral, (*b*) right medial and (*c*) ventral views. Abbreviations: boas, basioccipital articular surface; ci, crista interfenestralis; CN, cranial nerve passages; ct, crista tuberalis; fm, foramen magnum; fo, foramen ovale; mf, metotic fissure; oc, occipital condyle; pbsas, parabasisphenoid articular surface; pop, paroccipital process; pras, prootic articular surface. Scale bar = 8 mm.

**Figure 34.**
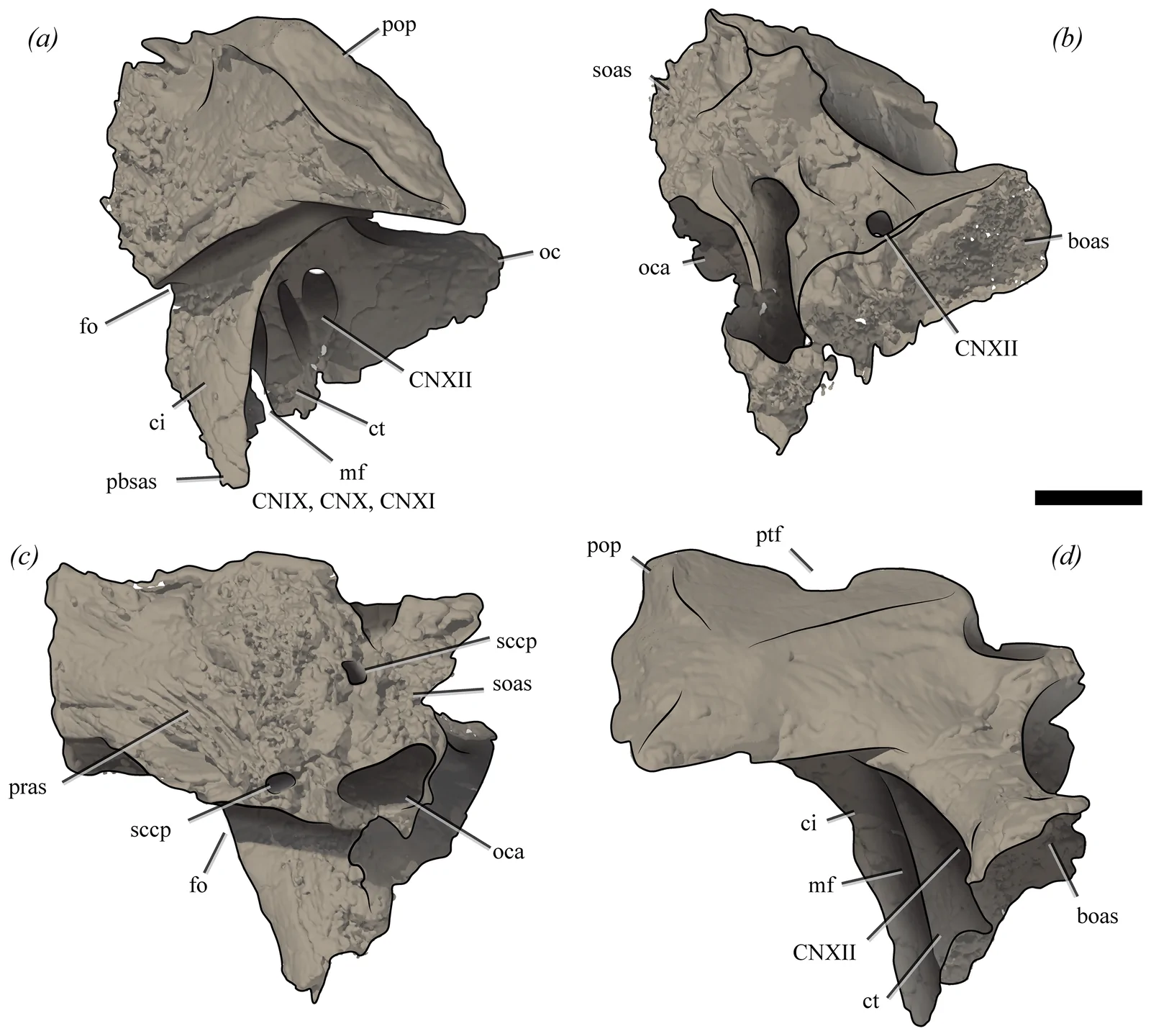
Simple schematics showing the openings of the reconstructed left otoccipital of BP/1/8468. (*a*) Lateral, (*b*) medial, (*c*) anterior and (*d*) posterior views. Abbreviations: boas, basioccipital articular surface; ci, crista interfenestralis; CN, cranial nerve passages; ct, crista tuberalis; fo, foramen ovale; mf, metotic fissure; oc, occipital condyle; oca, otic capsule; pbsas, parabasisphenoid articular surface; pop, paroccipital process; pras, prootic articular surface; ptf, post-temporal fenestra; sccp, semicircular canal passage; soas, supraoccipital articular surface. Scale bar = 8 mm.

The preserved portions of the paroccipital processes are wing-like, flare laterally and are directed posteroventrally. They are angled at 45° from the horizontal axis in lateral view. This is similar to the condition in *Coloradisaurus* and *Ngwevu*, whereas in *Massospondylus*, *Adeopapposaurus* and *Plateosaurus* they are near vertical. The paroccipital processes have convex dorsal and ventral margins, resulting in an elliptical cross-section. The distal end of the paroccipital processes is dorsoventrally expanded. The paroccipital processes (figure 32*c*) diverge from each other at an angle of 115° in dorsal view. This is similar to *Coloradisaurus* (105°), *Adeopapposaurus* (approx. 120°), *Yunnanosaurus* (110°) and NMQR 3314 (105°), but much wider than in *Plateosaurus* (60°), *Massospondylus* (67°), *Sarahsaurus* (approx. 90°) and *Lufengosaurus* (70°). A distinct notch is located on the anterior margin of the paroccipital processes, lateral to the supraoccipital articular surface, and forms a gap between the paroccipital processes and the posterior margin of the squamosal ramus of the parietal. This gap is interpreted as a small post-temporal fenestra (figure 34*d*), which is also present in *Macrocollum*, *Massospondylus*, *Adeopapposaurus*, *Ngwevu* and weakly in *Sarahsaurus*.

The crista interfenestralis forms the anteroventral margin of the otoccipital and is a thin, tapering, ventrally directed bony ridge that separates the foramen ovale (anteriorly) from the metotic foramen (posteriorly) (figure 34). Ventrally, it contacts the dorsolateral margin of the parabasisphenoid. The foramen ovale is bordered by the otoccipital (crista interfenestralis) posteriorly, the prootic anteriorly and anteroventrally by the parabasisphenoid. The foramen ovale is where the stapes interacts with the sacculus of the inner ear. The metotic fissure is bordered by the crista interfenestralis anteriorly and the crista tuberalis posteriorly. This foramen is the passage for the glossopharyngeal (CNIX), vagus (CNX) and the accessory (CNXI) nerves as well as the jugular vein (figure 34*a*).

The dorsoventral height of the otoccipital body is similar to that of *Sarahsaurus*, *Adeopapposaurus* and *Ngwevu*, but proportionately shorter than in *Massospondylus*. The contact surface between the otoccipital and prootic is a rugose butt joint that is directed anteriorly. The dorsal surface of the otoccipital is smooth. The supraoccipital contacts a small region on the dorsal and anteromedial margins of the otoccipital. This contact is rectangular in medial view, near vertical and directed anteromedially. The posteroventral projections of the otoccipital form the dorsolateral corners of the occipital condyle. They are directed medially and are triangular in lateral view. The posterior margin of the otoccipital's contribution to the condyle is continuous with the dorsal margin of the basioccipital condyle. This is similar to the condition in *Massospondylus*, *Sarahsaurus* and *Ngwevu* but differs markedly from *Macrocollum*, *Adeopapposaurus*, NMQR 3314, *Jingshanosaurus* and *Plateosaurus* where the otoccipital contributions to the condyle are visible as round protuberances on top of the condyle.

The crista tuberalis borders the metotic foramen posteriorly and separates it from the passage for the hypoglossal nerve (CNXII). The CNXII foramen is large, elliptical and oriented dorsoventrally. It houses two visible canals in the left otoccipital and three in the right otoccipital but exits through a single foramen visible on the medial and lateral surfaces. See figure 35 for a schematic of the reconstructed braincase of *Nyana* illustrating all the cranial openings and nerve passages. The foramen magnum is mediolaterally wider than it is dorsoventrally tall in posterior view, similar to those of NMQR 3314, *Ngwevu*, *Sarahsaurus*, *Adeopapposaurus*, *Coloradisaurus* and *Lufengosaurus*. This differs markedly from the dorsoventrally tall and mediolaterally narrow foramen magnum seen in *Macrocollum*, *Massospondylus*, *Yunnanosaurus* and *Plateosaurus*.

The otoccipital houses the posterior semicircular canal. The canal enters through the anterior surface of the otoccipital (the supraoccipital articular surface) and extends horizontally through the bone. It exits ventrally from the prootic articular surface to join the sacculus/lagena.

#### 5.3.9. Supraoccipital

The supraoccipital is complete, well-preserved and in life position (figure 36). The sagittal axis of the bone is angled 30° from the long axis of the skull in lateral view, similar to *Sarahsaurus*. This differs markedly from *Adeopapposaurus* where the supraoccipital is near vertical relative to the horizontal axis of the skull. The supraoccipital slopes posteroventrally and forms the roof of the foramen magnum. The supraoccipital contacts the parietal along its anterolateral margins, the laterosphenoid along its anteroventral corner, the prootic ventrally and the otoccipital posterolaterally.

**Figure 35.**
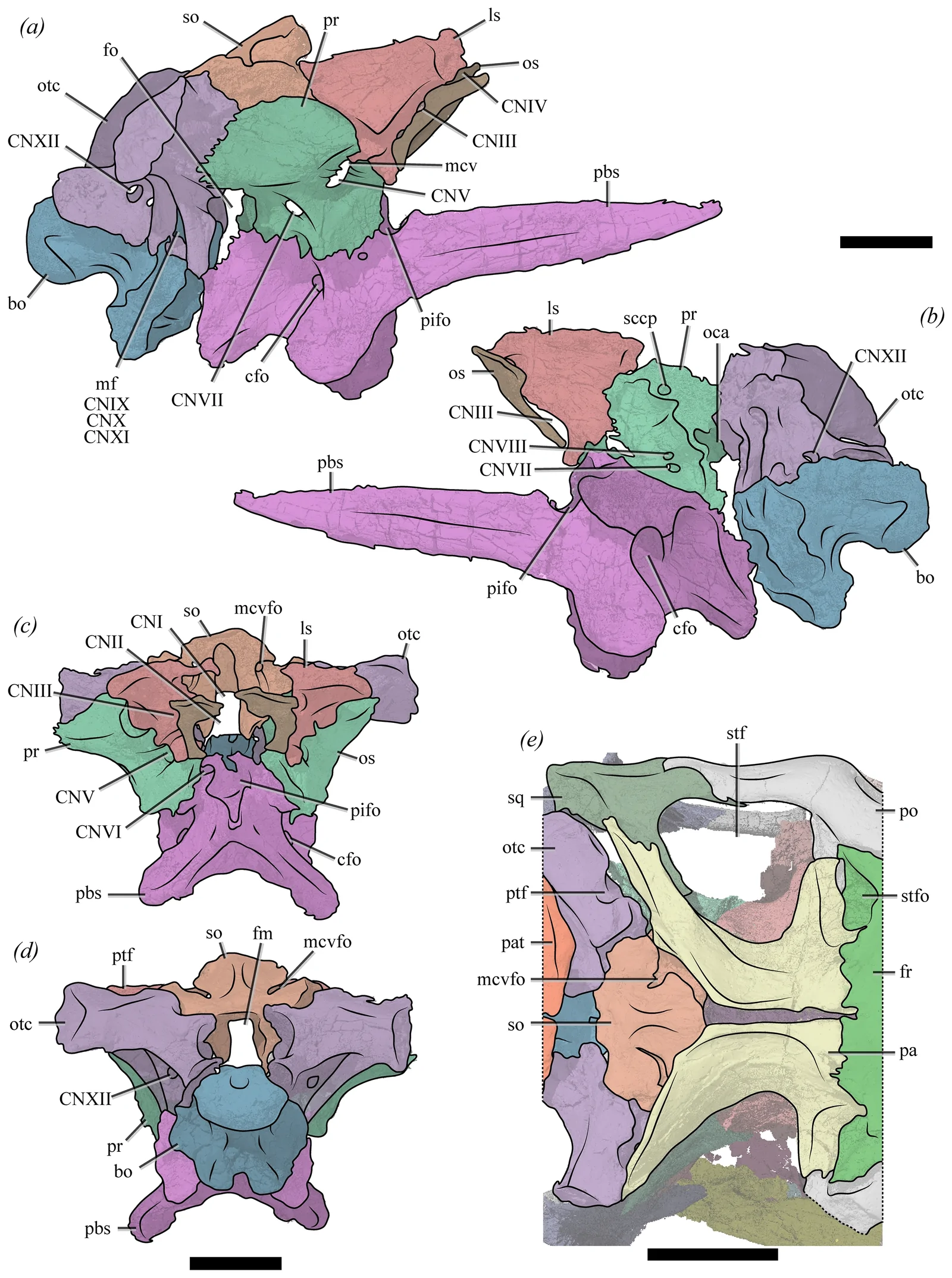
Illustrative drawing showing the major cranial openings and passages of BP/1/8468. (*a*) Right lateral, (*b*) right medial, (*c*) anterior and (*d*) posterior views. (*e*) Dorsal view of the skull. Abbreviations: bo, basioccipital; cfo, carotid foramen; CN, cranial nerve passages; fm, foramen magnum; fo, foramen ovale; fr, frontal; ls, laterosphenoid; mcv, mid-cerebral vein; mcvfo, mid-cerebral vein foramen; mf, metotic fissure; oca, otic capsule; os, orbitosphenoid; otc, otoccipital; pa, parietal; pat, proatlas; pbs, parabasisphenoid; pifo, pituitary fossa; po, postorbital; pr, prootic; ptf, post-temporal fenestra; sccp, semicircular canal passage; so, supraoccipital; sq, squamosal; stf, supratemporal fenestra; stfo, supratemporal fossa. Scale bars: (*a,b*) = 15 mm, (*c,d*) = 20 mm, (*e*) = 25 mm.

**Figure 36.**
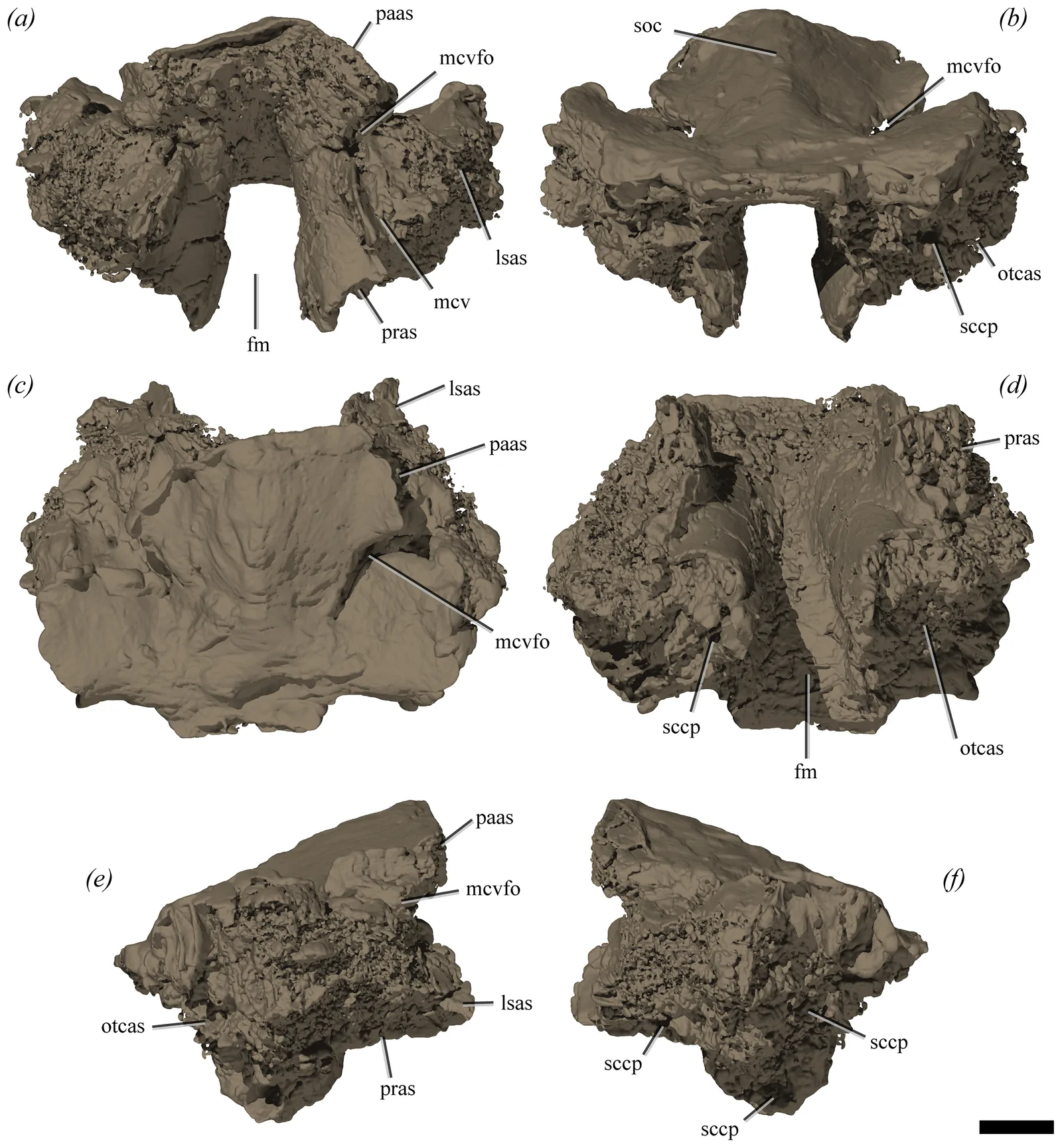
Reconstructed supraoccipital of BP/1/8468. (*a*) Anterior, (*b*) posterior, (*c*) dorsal, (*d*) ventral, (*e*) right lateral and (*f*) left lateral views. Abbreviations: fm, foramen magnum; lsas, laterosphenoid articular surface; mcv, mid-cerebral vein passage; mcvfo, mid-cerebral vein foramen; otcas, otoccipital articular surface; paas, parietal articular surface; pras, prootic articular surface; sccp, semicircular canal passage; soc, supraoccipital crest. Scale bar = 5.5 mm.

The supraoccipital is rectangular in dorsal view (figure 36*c*) with a supraoccipital crest on the midline of its dorsal surface. The dorsal surface is sinuous in posterior view, so that it is convex along the midline, then is concave at the passage for the mid-cerebral vein and slopes posterodorsally towards the lateral margins. The posterior corners of the supraoccipital extend laterally and articulate with the bases of the paroccipital processes along butt joints. This contact is angled ventrolaterally and triangular in posterior view. The otoccipital articular surface extends across the entire dorsoventral height of the supraoccipital. The posterior margin of the supraoccipital is mediolaterally wider than its anterior margin in dorsal view. This differs from the diamond-shaped supraoccipital of NMQR 3314 and the mediolaterally expansive supraoccipital of *Yunnanosaurus*.

The supraoccipital contacts the prootics along a rugose, anterolaterally oriented butt joint that is triangular in posterior view and its apex directed posteroventrally. The supraoccipital contacts the laterosphenoids via a small, triangular tab with its apex directed dorsolaterally and its articular surface oriented anteroventrally.

The contact between the supraoccipital and the parietal is a thin, dorsally directed, rugose butt joint. There is a sulcus on the dorsal surface between the anterolateral margin of the supraoccipital and the squamosal ramus of the parietal, forming the passage for the mid-cerebral vein. This feature is located lateral to the supraoccipital crest. This sulcus leads anteriorly to the mid-cerebral vein foramen which then extends medially and ventrally along the anterior surface of the supraoccipital. The ventral margin of the supraoccipital forms the roof of the foramen magnum. It has an inverted, deep, ‘U’-shaped cross-section in anterior view. In ventral view, this cavity is mediolaterally constricted along its median and is mediolaterally expanded both anteriorly and posteriorly. This weakly hourglass-shaped cavity is similar to that of *Ngwevu* but differs from the strongly, mediolaterally constricted centre seen in *Plateosaurus*, and also from *Massospondylus*, which has a mediolaterally narrow posterior end and a mediolaterally wider anterior end.

The junction between the posterior and anterior semicircular canals is located in the interior of the supraoccipital. The posterior canal enters the supraoccipital through the otoccipital articular surface, and the anterior canal enters through the prootic articular surface. The anterior and posterior canals meet near the lateral walls of the foramen magnum formed by the supraoccipital where they extend ventrally to join the sacculus.

### 5.4. Otic region

#### 5.4.1. Stapes

Both stapes are well-preserved but have undergone minor deformation and dorsoventral crushing (figure 37*j*–*l*). The proximal end of the left stapes is broken and separated from the shaft, and the right stapes is complete and close to life position. The stapedial shafts are somewhat dorsoventrally crushed towards their distal ends, but the proximal ends are intact, slender and dorsoventrally compressed. The shafts curve weakly medially in dorsal view. The stapes is angled anteromedially to posterolaterally and is lying across the anterolateral surface of the otoccipital where it enters the fenestra ovalis to contact the lagena which emerges from the sacculus. The long axis of the stapes lies along the main axis of the skull. The stapedial footplate that contacts the sacculus is semicircular in proximal view. It has a convex dorsal margin and a straight ventral margin and is bordered by a distinct raised rim. The stapedial foramen is located on the dorsal surface of the footplate.

**Figure 37.**
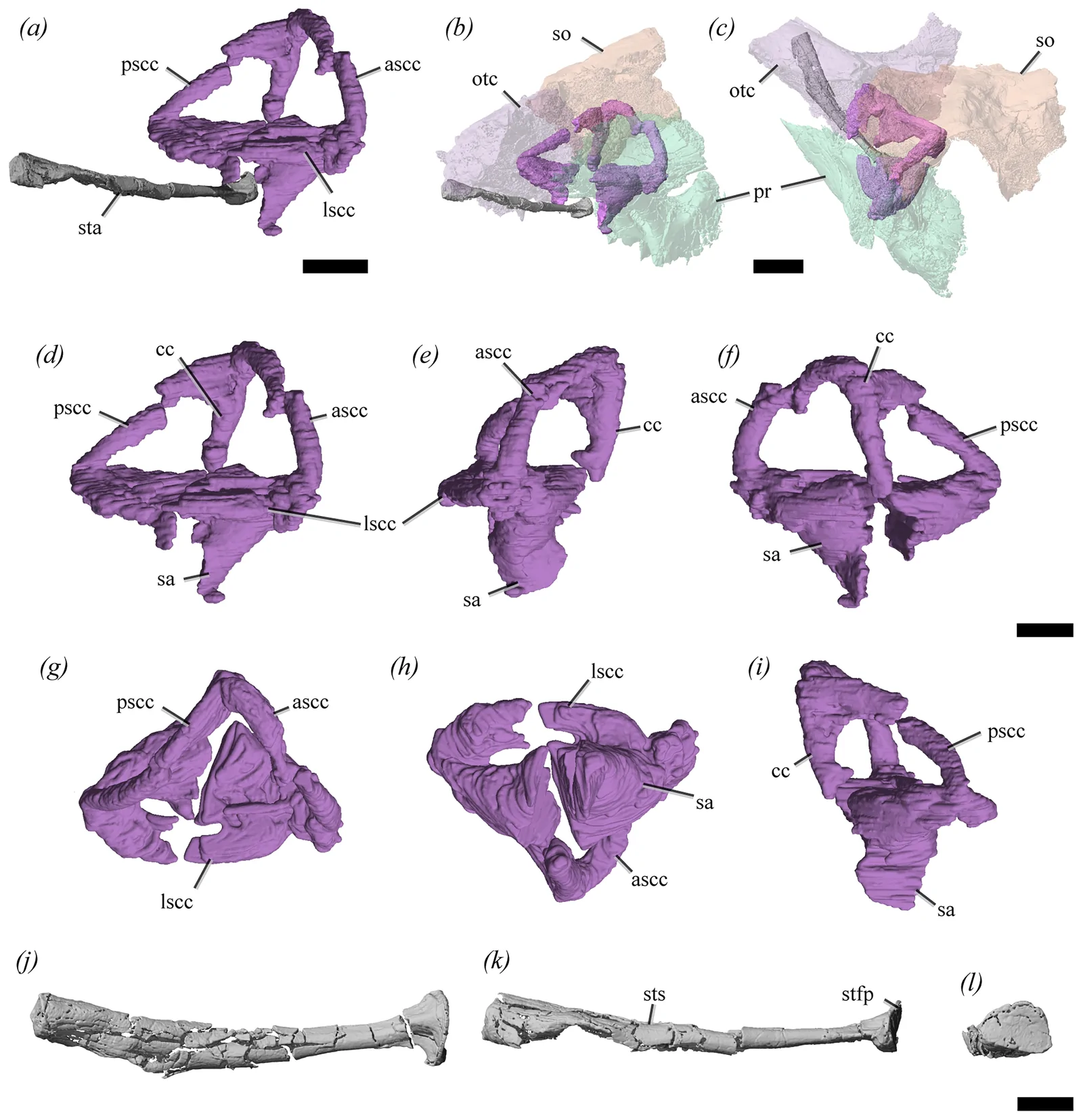
Reconstructed right semicircular canals and stapes of BP/1/8468. (*a*) Right semicircular canals and stapes in lateral view showing their contact. Right semicircular canals, stapes and semi-transparent encasing bones in (*b*) lateral view and (*c*) dorsal views. Right semicircular canal in (*d*) lateral, (*e*) anterior, (*f*) medial, (*g*) dorsal, (*h*) ventral and (*i*) posterior views. Right stapes in (*j*) dorsal, (*k*) lateral and (*l*) proximal views. Abbreviations: ascc, anterior semicircular canal; cc, common crus; lscc, lateral semicircular canal; otc, otoccipital; pr, prootic; pscc, posterior semicircular canal; sa, sacculus; so, supraoccipital; sta, stapes; stfp, stapedial footplate; sts, stapedial shaft. Scale bars: (*a*) = 7 mm, (*b,c*) = 8.5 mm, (*d–i*) = 5 mm, (*j–l*) = 4 mm.

#### 5.4.2. Inner ear

The passages for the semicircular canals are preserved in both sides of the neurocranium (figure 37*a*–*i*; see above). The semicircular canals pass through the prootic, otoccipital and supraoccipital. The lateral semicircular canal is the shortest of the three inner ear canals. The anterior, posterior and lateral canals are ovoid in cross-section. The posterior and anterior canals join dorsomedially, and the anterior canal is dorsally elevated to the posterior canal. The posterior and anterior canals join medially and form the common crus. The sacculus is incomplete but appears conical with its apex directed ventrally. The semicircular canals are anteroposteriorly longer than their dorsoventral height. The semicircular canals of *Nyana* are proportionally anteroposteriorly longer than other reconstructed canals of sauropodomorphs such as *Massospondylus*, *Sarahsaurus*, *Saturnalia*, *Thecodontosaurus*, including those of sauropods such as *Spinophorosaurus* [92] and *Europasaurus* [93]. This could be considered a potential autapomorphy. Refer to S3 in the supplementary material for detailed quantitative information on the semicircular canals.

### 5.5. Mandible

The right mandible (figure 38) is partially preserved and includes a partial dentary, surangular, angular, coronoid, splenial and prearticular. All these elements are preserved in life position relative to each other.

**Figure 38.**
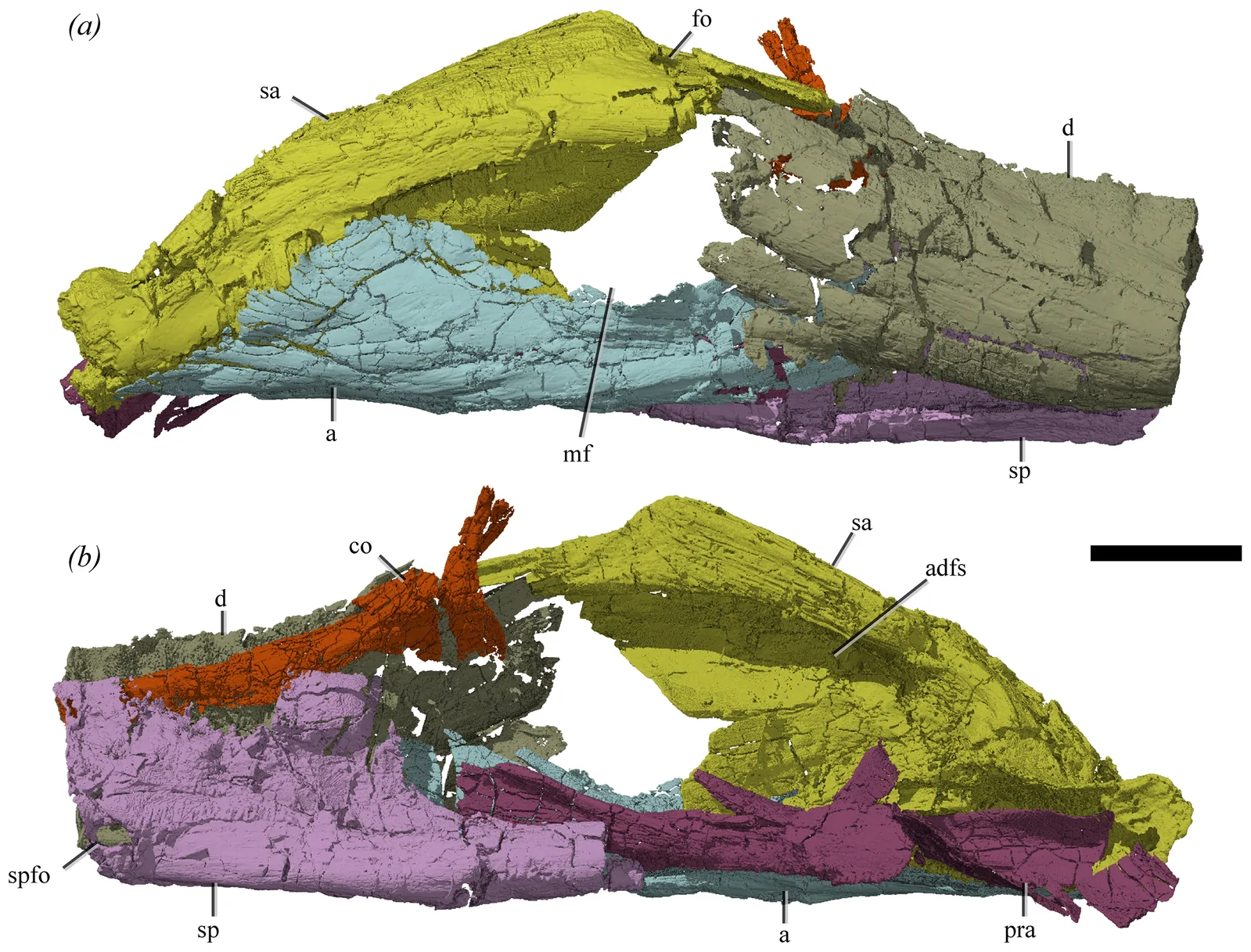
Reconstructed right mandible of BP/1/8468. (*a*) Lateral and (*b*) medial views. Abbreviations: a, angular; adfs, adductor fossa; co, coronoid; d, dentary; fo, foramen; mf, mandibular fenestra; pra, prearticular; sa, surangular; sp, splenial; spfo, splenial foramen. Scale bar = 15 mm.

#### 5.5.1. Dentary

The posterior portion of the right dentary is preserved and contains seven alveoli. The dentary contacts the coronoid dorsomedially, the splenial ventromedially, the angular posteromedially and ventrally and the surangular posterodorsally. Its lateral surface is smooth and convex. The dorsal margin of the dentary is weakly concave, and the ventral margin is convex, resulting in a weakly dorsally curving dentary in lateral view. This is similar to that of *Coloradisaurus* and *Sarahsaurus*. The posterior margin of the dentary is angled posterodorsally in lateral view, differing from other sauropodomorphs that have a posteriorly facing margin such as *Ngwevu*, *Adeopapposaurus*, *Plateosaurus*, *Lufengosaurus*, *Yunnanosaurus* and NMQR 3314. The ventral margin of the dentary folds medially, contacting the splenial along its medial margin of the ventral surface. The medial surface is an anteroposteriorly long sheet forming the medial margin of the alveoli. It is dorsoventrally shorter than the lateral surface. The alveoli decrease in mediolateral width posteriorly, but the third alveolus from the posterior end is the mediolaterally widest. The dentary teeth are medially inset. The dentary forms the anterior margin of the mandibular fenestra in lateral view. It is unclear if any interdental plates or nutrient foramina are present.

#### 5.5.2. Surangular

The surangular is complete and relatively well-preserved. It contacts the dentary anteroventrally along its lateral margin, the coronoid anterodorsally along its medial margin, the angular ventrolaterally and the prearticular ventromedially. It forms the dorsal and posterior margins of the mandibular fenestra. The surangular is anteroposteriorly longer than it is dorsoventrally tall. The dorsal margin is convex, and the ventral margin is straight. The lateral surface is convex, and the medial surface is concave. The posterior margin is the dorsoventrally shortest region of the surangular.

The dorsal margin forms an overhang on its medial side that mediolaterally widens as it extends anteriorly. A foramen enters this overhang from the anterodorsal margin of the surangular and exits into a deep trough formed by the overhang and into the adductor fossa. This anterodorsal margin extends over the dorsal margin of the dentary for a short distance. A cup-shaped embayment is located posterodorsally on the dorsal margin of the surangular, which is continuous with a medially expanding flange; together these features form part of the cotyle that would have received the quadrate.

The mandibular fenestra is dorsoventrally taller than its anteroposterior length. It is oriented at 45° dorsally from the horizontal axis, similar to that of *Plateosaurus*.

#### 5.5.3. Angular

The angular is complete and well-preserved. It is a smooth, mediolaterally thin plate that forms the ventral margin of the posterior end of the mandible. The angular contacts the surangular posteromedially and the dentary anterolaterally, and the ventral margin folds medially to contact the prearticular. An anteroposteriorly long foramen is formed along this contact with the prearticular and is located anteriorly. It forms the ventral margin of the mandibular fenestra. In lateral view, the dorsal margin is ‘S’-shaped with a convex margin on its posterior end and a concave margin at its anterior end. Its surfaces are smooth with a convex lateral surface and a concave medial surface.

#### 5.5.4. Coronoid

The coronoid is relatively well-preserved. It is an anteroposteriorly long plate that forms part of the dorsal margin in the mandible. It contacts the dentary laterally and the splenial medially. It is mediolaterally thin with smooth surfaces. Its dorsal and ventral margins are straight in lateral view. The posterior end of the coronoid diverges into two processes, a larger dorsal and smaller ventral process. The dorsal process contacts the anterodorsal margin of the surangular.

#### 5.5.5. Splenial

The splenial is a mediolaterally thin plate forming the medial portion of the mandibular ventral margin. The ventral margin of the splenial folds laterally to meet the dentary on its lateral side, contacts the coronoid dorsally, and meets the prearticular posteriorly. Its dorsoventral height is similar to that of the dentary. An elliptical splenial foramen is preserved at its anteroventral end. Its posterior margin is recessed with a concave dorsal margin, forming a dorsoventrally low posterior process that borders the adductor fossa ventrally and lies medial to the prearticular. Its medial surface is smooth with no notable features.

#### 5.5.6. Prearticular

The prearticular is fragmentary but preserved in life position. It lies medial to the surangular and posterior to the splenial and forms the ventral margin of the adductor fossa. It is anteroposteriorly long and dorsoventrally short, becoming rod-like at its midline and plate-like towards its anterior and posterior ends. The anterior and posterior ends are dorsoventrally expanded with respect to the main body. A notch on the anteroventral margin forms the medial border of the foramen it shares with the angular. At the midline of the prearticular is a plate of bone that extends anterodorsally and posterodorsally. The prearticular articular surface of the surangular contacts this plate. The posterior end widens posteriorly to make contact with the articular.

### 5.6. Dentition

There are 23 alveoli preserved in the partial right maxilla and 7 in the incomplete right dentary (figure 39). There are seven erupted teeth and four partial replacement teeth in the right dentary; five erupted teeth and two partial replacement teeth in the left maxilla; and 24 erupted teeth and six replacement teeth in the right maxilla; and one crown is displaced posteriorly. The teeth are well-preserved and in relative life position with a few of them slightly displaced. Most of the teeth have intact roots with a few of the replacement teeth preserving the crowns. The replacement teeth are positioned lingual to the erupted teeth and have denticles preserved.

**Figure 39.**
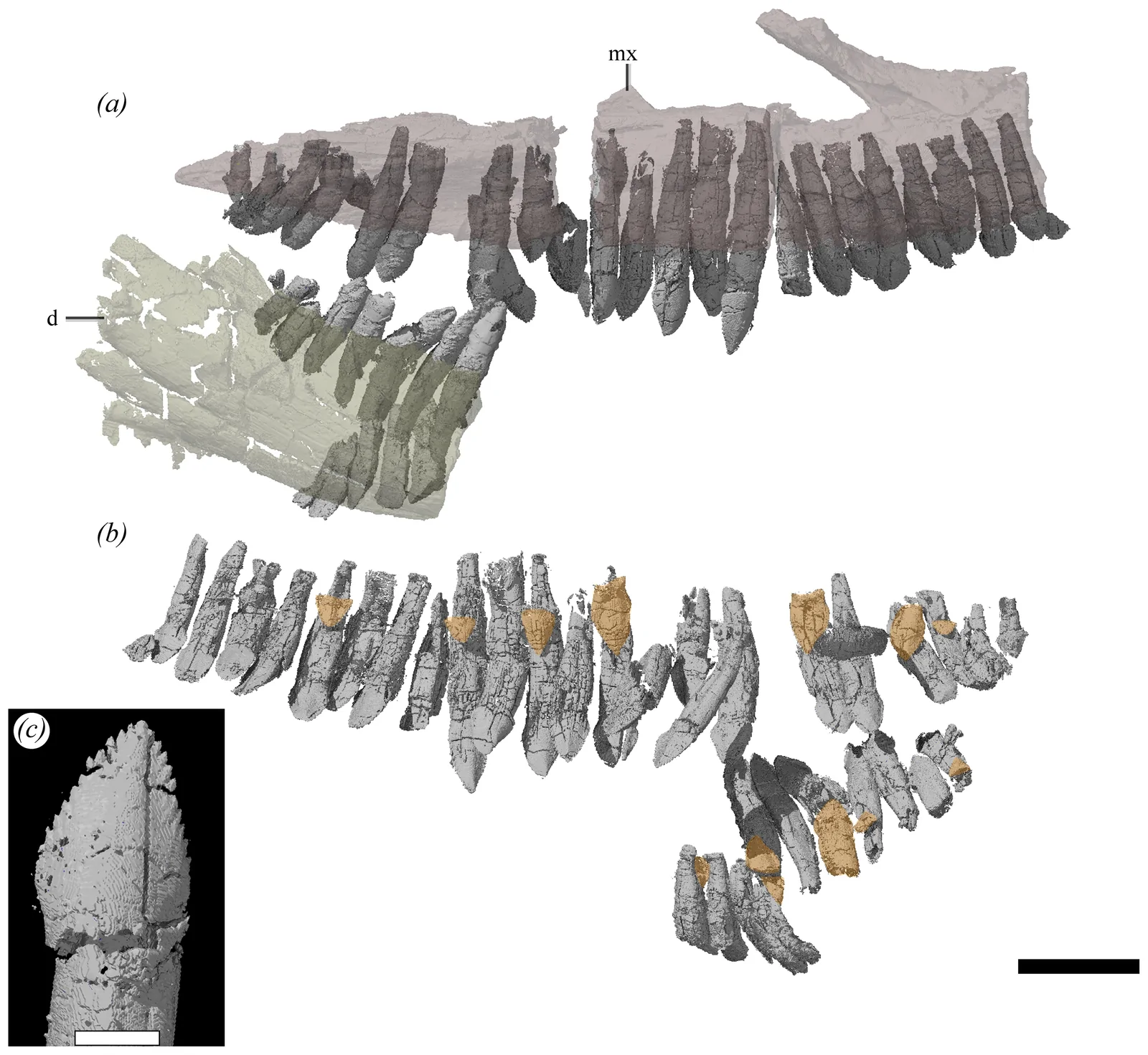
Reconstructed erupted and replacement teeth of BP/1/8468. (*a*) Lateral view with semi-transparent maxilla and dentary to show the location of the teeth and the extent of the roots. (*b*) Teeth in medial view to show the location of the replacement teeth (highlighted in orange). (*c*) Close-up of a tooth. Abbreviations: d, dentary; mx, maxilla. Scale bars: = (*a,b*) 15 mm, (*c*) = 2.5 mm.

Each tooth overlaps the following tooth mesially in an en echelon arrangement. All maxillary and dentary tooth crowns are spatulated and lingually recurved. The teeth bear denticles along their mesial and distal carinae (approx. 11 denticles on both sides) that extend for the entire apicobasal height of the tooth (figure 39*c*), differing from those of massospondylids that have denticles restricted to the apical third of the tooth crown (e.g. *Massospondylus*, *Ngwevu*, *Adeopapposaurus* and SMF 13.5.37). The denticles are pointed, extending apically at an angle of 45° from the tooth margin, and are spaced with two denticles per millimetre. The tooth crowns decrease in apicobasal height posteriorly. The roots are elliptical in cross-section and represent approximately 70% of the entire apicobasal height of the teeth. The maxillary and dentary replacement teeth are in alternating tooth positions and positioned lingual to the erupted teeth. The replacement teeth also bear denticles along their mesial and distal carinae, resembling the morphology of erupted teeth.

### 5.7. Hyoid apparatus

A partial left hyoid is preserved in two fragments close to life position (figure 40*a*–*d*). It is a rod-like structure with a smooth surface. The shaft maintains its dimensions and expands towards the proximal end. The proximal end is triangular and has a flat proximal surface.

**Figure 40.**
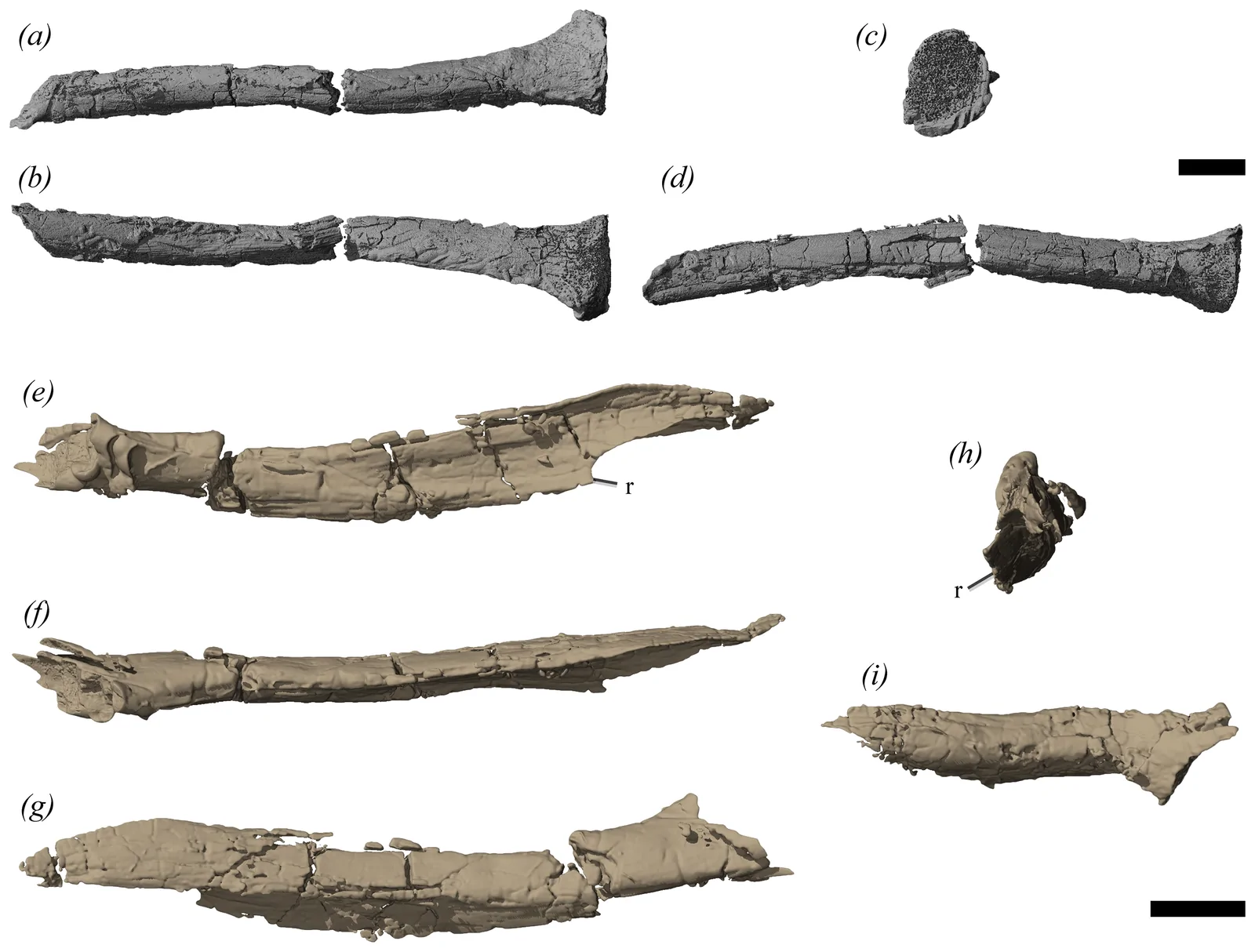
Reconstructed (*a–d*) left hyoid and (*e–i*) ceratobranchials of BP/1/8468. (*a*–*d*) Left hyoid in (*a*) lateral, (*b*) medial, (*c*) proximal and (*d*) dorsal views. (*e*–*i*) Right ceratobranchial in (*e*) lateral, (*f*) dorsal, (*g*) medial and (*h*) anterior views. Left ceratobranchial in (*i*) dorsal view. Abbreviations: r, ridge. Scale bar (*a–d*) = 6 mm, (*e–i*) = 4.5 mm.

Partial rod-like structures are preserved medial to the mandibles (figure 40*e*–*i*). They are weakly sigmoidal in medial and lateral views. Both are mediolaterally compressed and dorsoventrally tall at their posterior ends resulting in an elliptical cross-section. A ridge grades out of the lateral surface as it extends anteriorly creating a ventrally facing furrow. This creates a ‘U’-shaped cross-section towards the anterior end. The topological and morphological similarities of this element with those in other dinosaurs [94,95] suggest that these elements represent a pair of second ceratobranchials. The partially preserved hyoid apparatus in *Nyana* is similar to the few that are preserved and visible in other taxa, such as those of *Buriolestes* [96] and *Macrocollum* [60]. Based on the morphology of these elements, they can be attributed to the hyoid and second ceratobranchials, but it is possible that they could represent other elements of the hyoid apparatus.

### 5.8. Scleral rings

The scleral ossicles are disarticulated and out of life position (figure 41). There are at least 34 preserved ossicles, with a few overlapping adjacent ossicles to form a partial ring. Individual ossicles are subrectangular in outline and very thin. Their surfaces are variably convex and concave. The ossicles that form the outer circumference of the eye display a rectangular tab. The orientations of the ossicles are unknown and difficult to determine.

**Figure 41.**
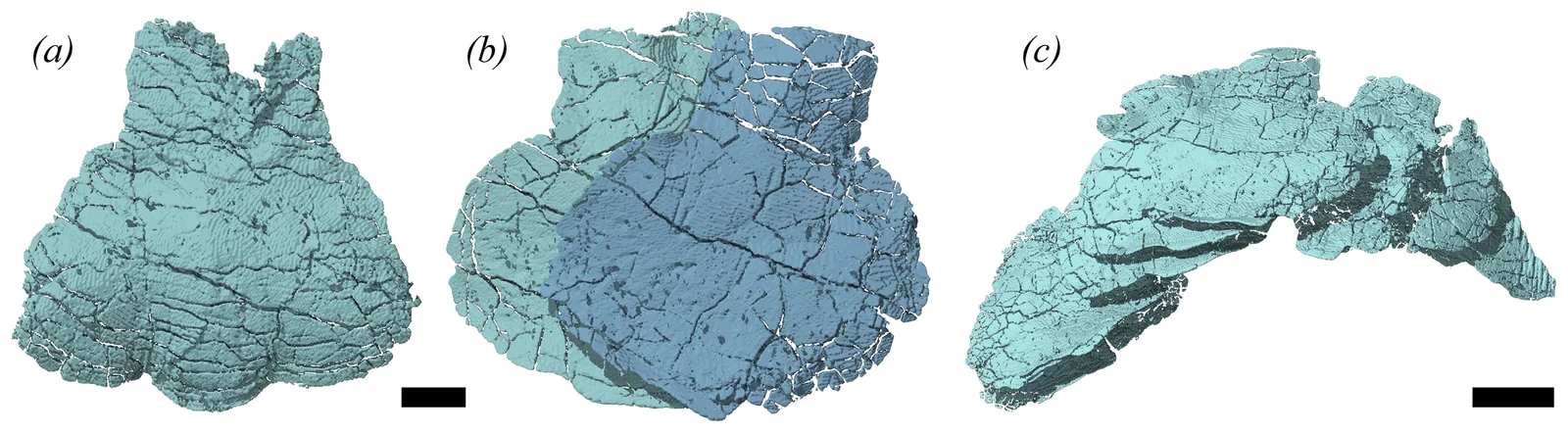
Reconstructed scleral ossicles of BP/1/8468. (*a*) A scleral ossicle. (*b*) Scleral ossicles overlapping each other. (*c*) A few of the preserved scleral ossicles overlapping each other and forming an arch. All scleral ossicles are oriented in such a way that the centre of the orbit would be below. Scale bars: (*a,b*) = 2 mm, (*c*) = 4 mm.

### 5.9. Postcranial material

*Nyana rooiwalensis* is also represented by disarticulated, partially preserved postcranial element (figure 42–figure 59), including both proatlases; atlantal neural arches; the atlantal intercentrum; various vertebrae, including four cervicals, one cervicodorsal, one dorsal, one dorsal neural arch, primordial sacral one and two caudals; the proximal end of a left ulna; a manual phalanx; a manual ungual; the proximal and distal end of a right femur; the distal end of a left tibia; and a pedal ungual. The neurocentral sutures are unfused in all the preserved vertebrae, and there is no indication of pneumatization.

**Figure 42.**
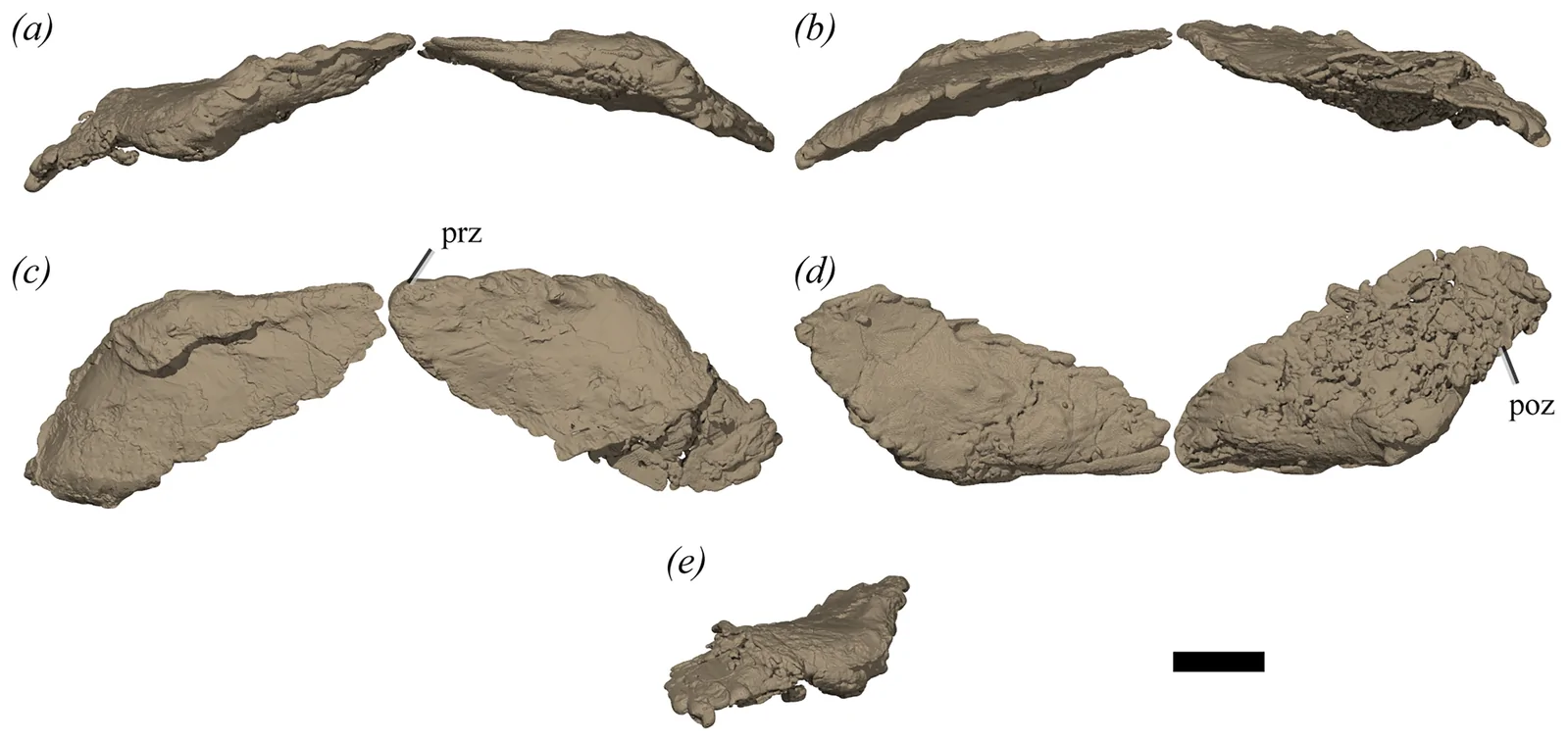
The proatlas of BP/1/8468 in (*a*) anterior, (*b*) posterior, (*c*) dorsal, (*d*) ventral and (*e*) lateral views. Abbreviations: poz, postzygapophysis; prz, prezygapophysis. Scale bar = 7 mm.

**Figure 43.**
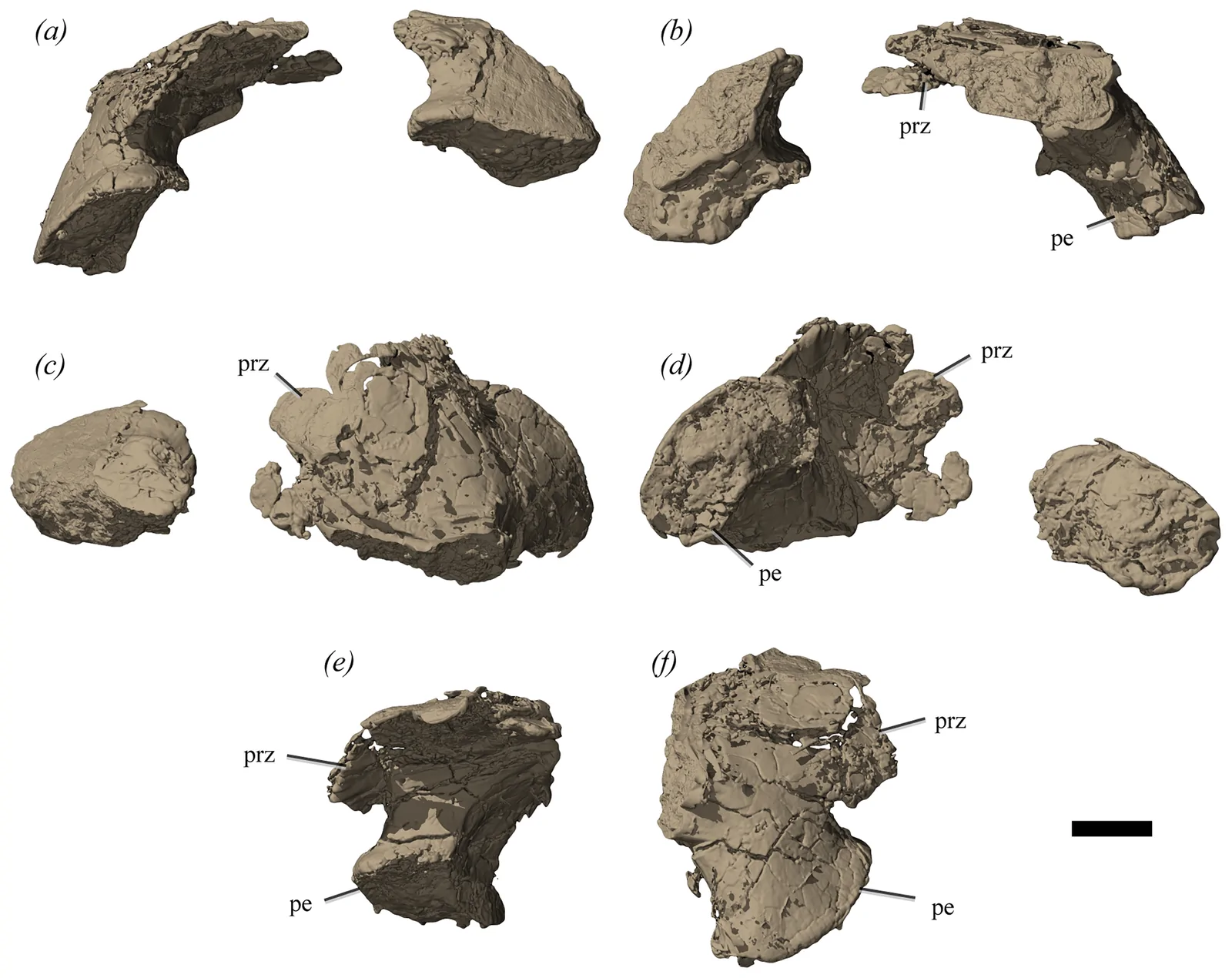
Atlantal neural arches of BP/1/8468 in (*a*) anterior, (*b*) posterior, (*c*) dorsal, (*d*) ventral and (*e*) medial view of the right neural arch, and (*f*) lateral view of the right neural arch. Abbreviations: pe, pedicel; prz, prezygapophysis. Scale bar = 6 mm.

**Figure 44.**
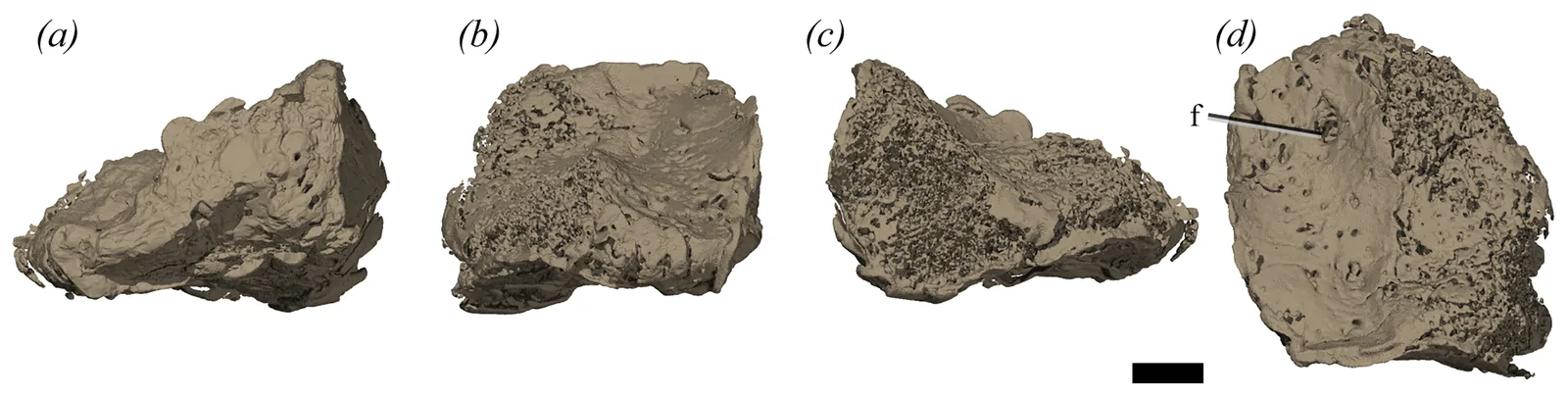
Atlantal intercentrum of BP/1/8468 in (*a*) left lateral, (*b*) anterior, (*c*) right lateral and (*d*) dorsal views. Abbreviations: fo, foramen. Scale bar = 4 mm.

**Figure 45.**
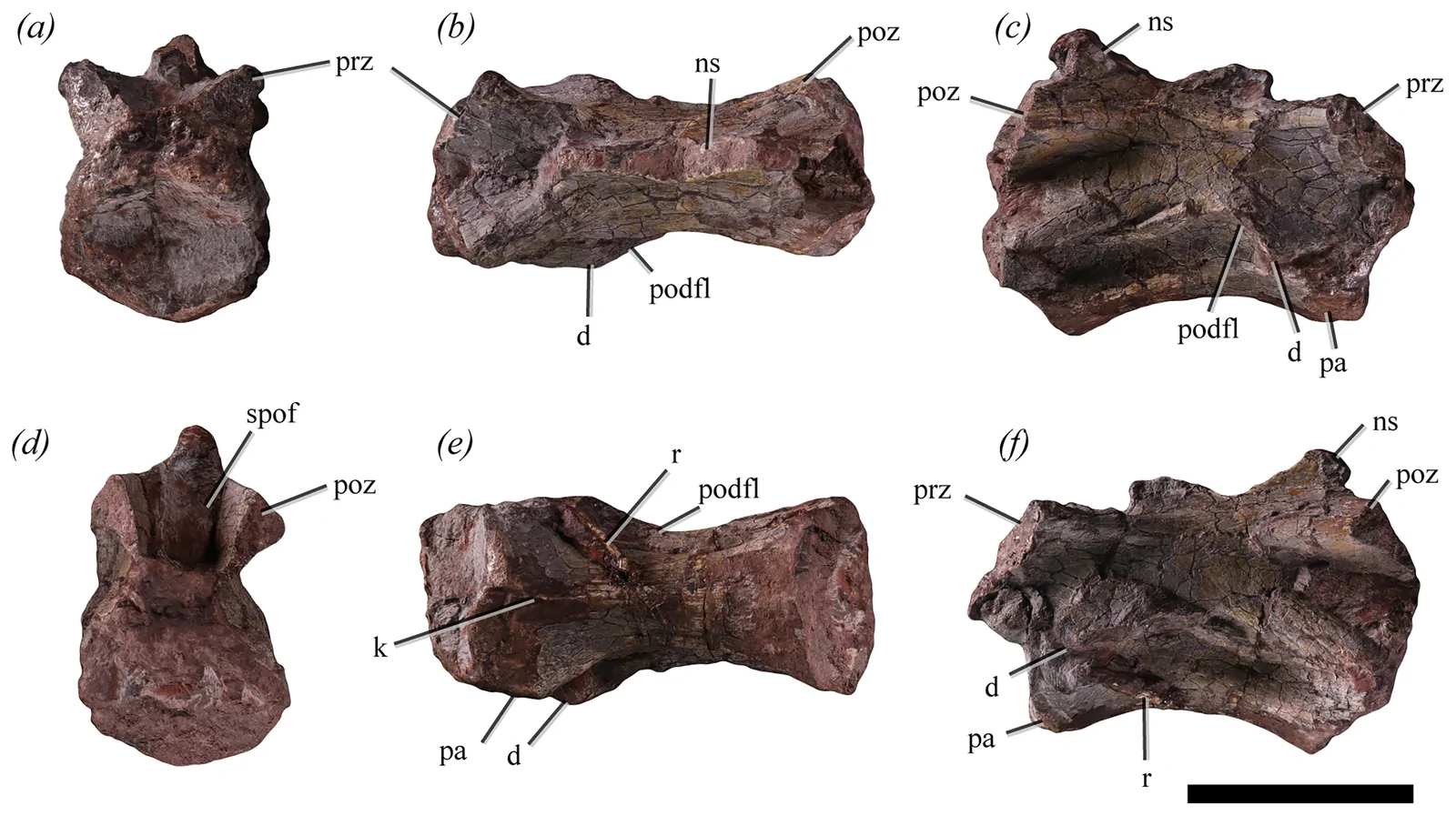
Cervical 4 of BP/1/8468 in (*a*) anterior, (*b*) dorsal, (*c*) right lateral, (*d*) posterior, (*e*) ventral and (*f*) left lateral views. Abbreviations: d, diapophysis; k, ventral keel; ns, neural spine; pa, parapophysis; podfl, postdiapophyseal flange; poz, postzygapophysis; prz, prezygapophysis; r, rib; spof, spinopostzygapophyseal fossa. Scale bar = 5 cm.

**Figure 46.**
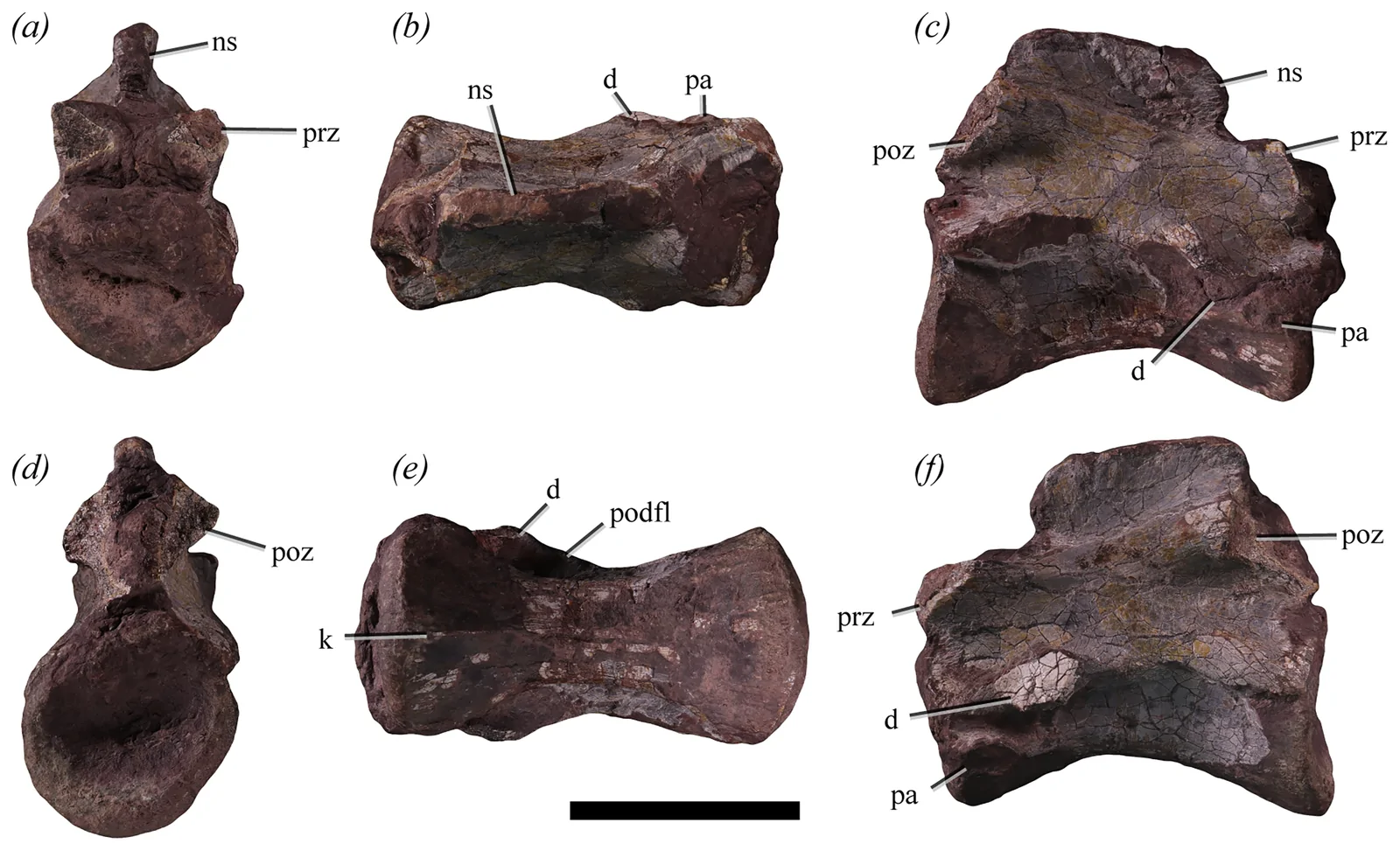
Cervical 6 of BP/1/8468 in (*a*) anterior, (*b*) dorsal, (*c*) right lateral, (*d*) posterior, (*e*) ventral and (*f*) left lateral views. Abbreviations: d, diapophysis; k, ventral keel; ns, neural spine; pa, parapophysis; podfl, postdiapophyseal flange; poz, postzygapophysis; prz, prezygapophysis. Scale bar = 5 cm.

**Figure 47.**
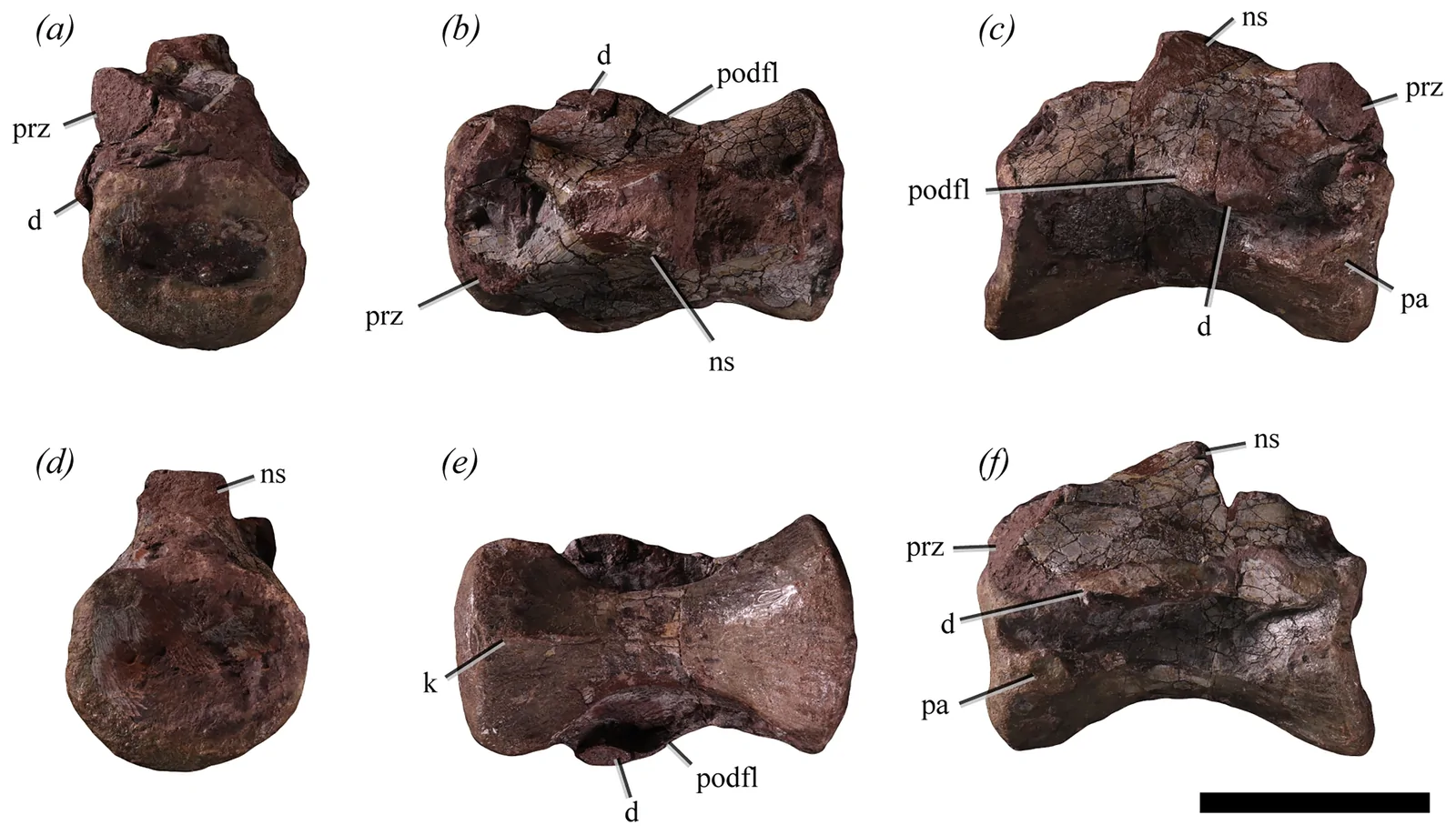
Cervical 7 of BP/1/8468 in (*a*) anterior, (*b*) dorsal, (*c*) right lateral, (*d*) posterior, (*e*) ventral and (*f*) left lateral views. Abbreviations: d, diapophysis; k, ventral keel; ns, neural spine; pa, parapophysis; podfl, postdiapophyseal flange; prz, prezygapophysis. Scale bar = 5 cm.

**Figure 48.**
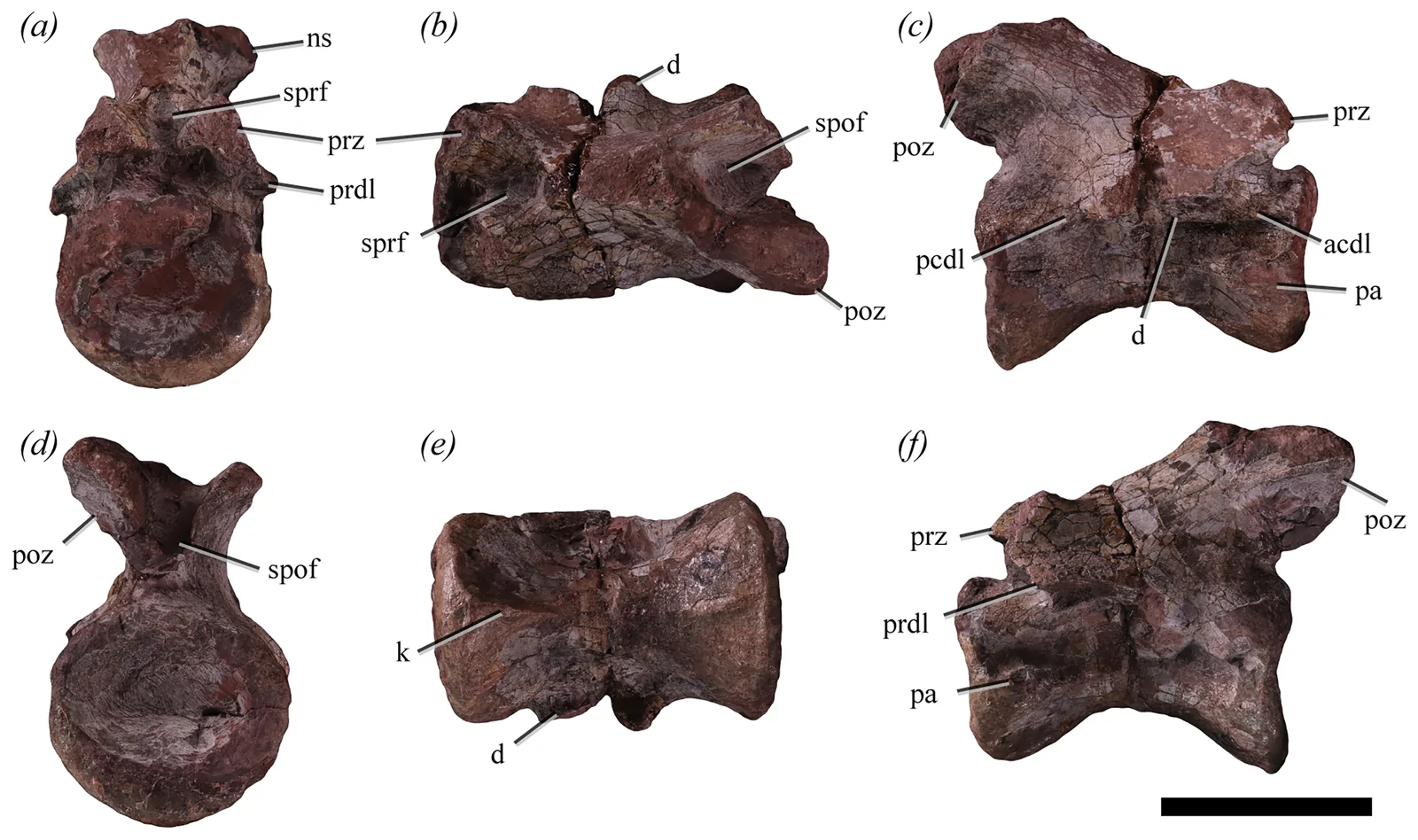
Cervical 9 of BP/1/8468 in (*a*) anterior, (*b*) dorsal, (*c*) right lateral, (*d*) posterior, (*e*) ventral and (*f*) left lateral views. Abbreviations: acdl, anterior centrodiapophyseal lamina; d, diapophysis; k, ventral keel; ns, neural spine; pa, parapophysis; pcdl, posterior centrodiapophyseal lamina; prdl, prezygodiapophyseal lamina; poz, postzygapophysis; prz, prezygapophysis; spof, spinopostzygapophyseal fossa; sprf, spinoprezygapophyseal fossa. Scale bar = 5 cm.

**Figure 49.**
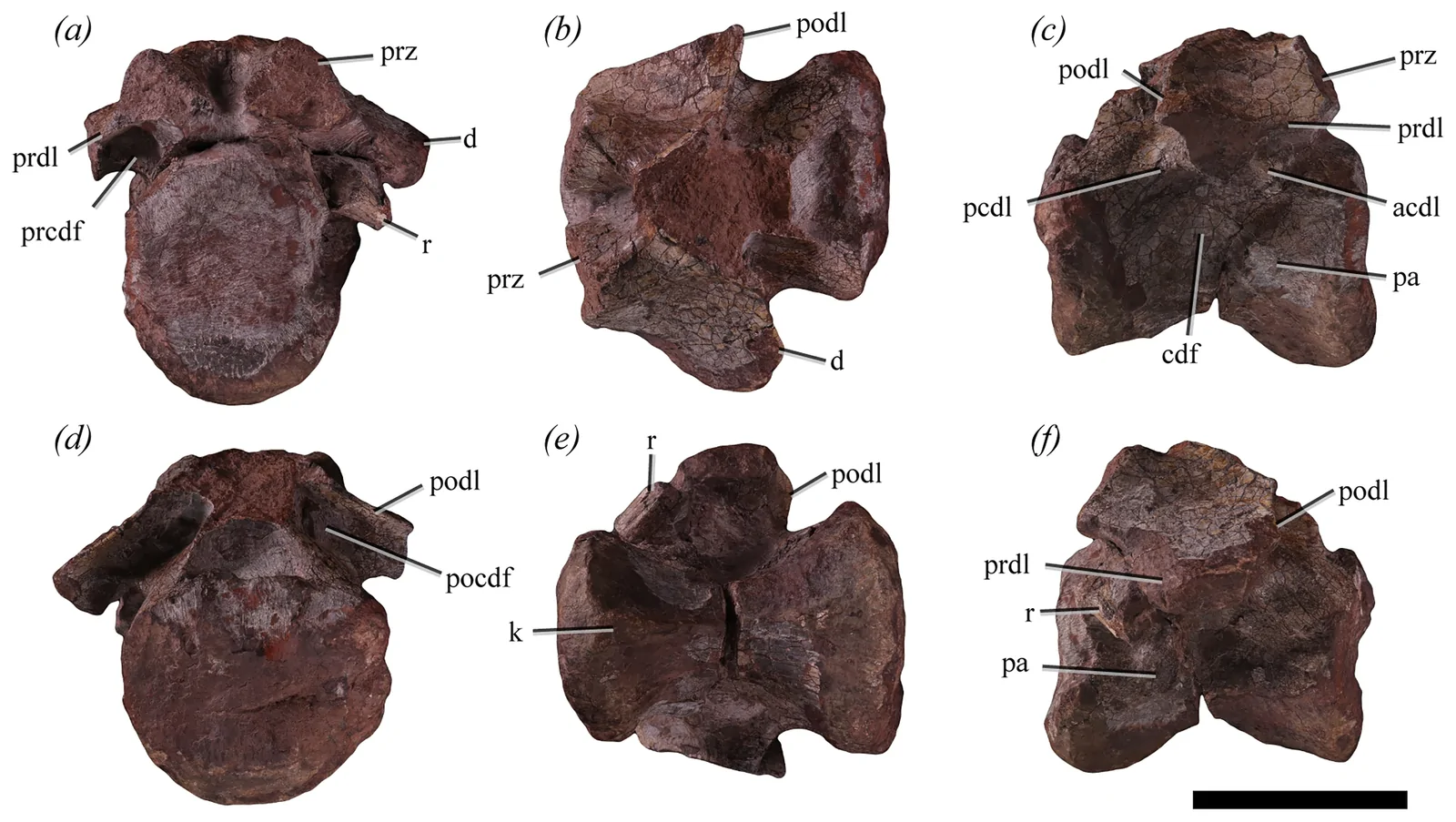
Cervicodorsal of BP/1/8468 in (*a*) anterior, (*b*) dorsal, (*c*) right lateral, (*d*) posterior, (*e*) ventral, and (*f*) left lateral views. Abbreviations: acdl, anterior centrodiapophyseal lamina; cdf, centrodiapophyseal fossa; d, diapophysis; k, ventral keel; pa, parapophysis; pcdl, posterior centrodiapophyseal lamina; pocdf, postzygocentrodiapophyseal fossa; podl, postzygodiapophyseal lamina; prcdf, prezygocentrodiapophyseal fossa; prdl, prezygodiapophyseal lamina; prz, prezygapophysis; r, rib. Scale bar = 5 cm.

**Figure 50.**
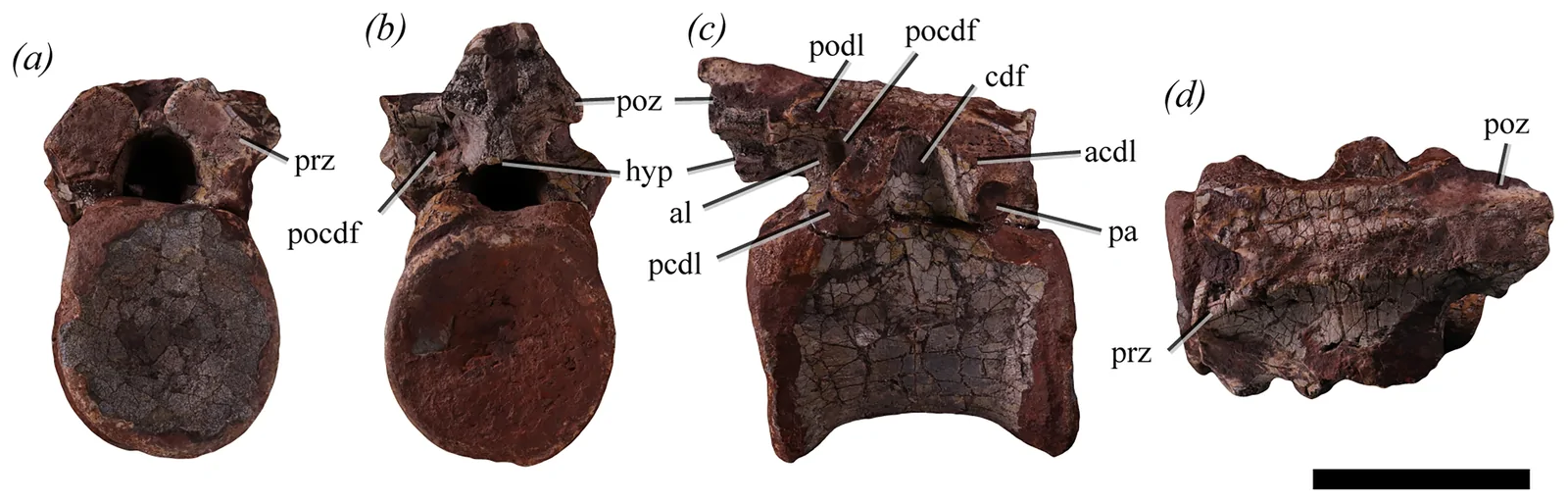
Anterior-to-middle dorsal of BP/1/8468 in (*a*) anterior, (*b*) posterior, (*c*) right lateral and (*d*) dorsal views. Abbreviations: acdl, anterior centrodiapophyseal lamina; al, accessory lamina; cdf, centrodiapophyseal fossa; hyp, hyposphene; pa, parapophysis; pcdl, posterior centrodiapophyseal lamina; pocdf, postzygodiapophyseal fossa; podl, postzygodiapophyseal lamina; poz, postzygapophysis; prz, prezygapophysis. Scale bar = 5 cm.

**Figure 51.**
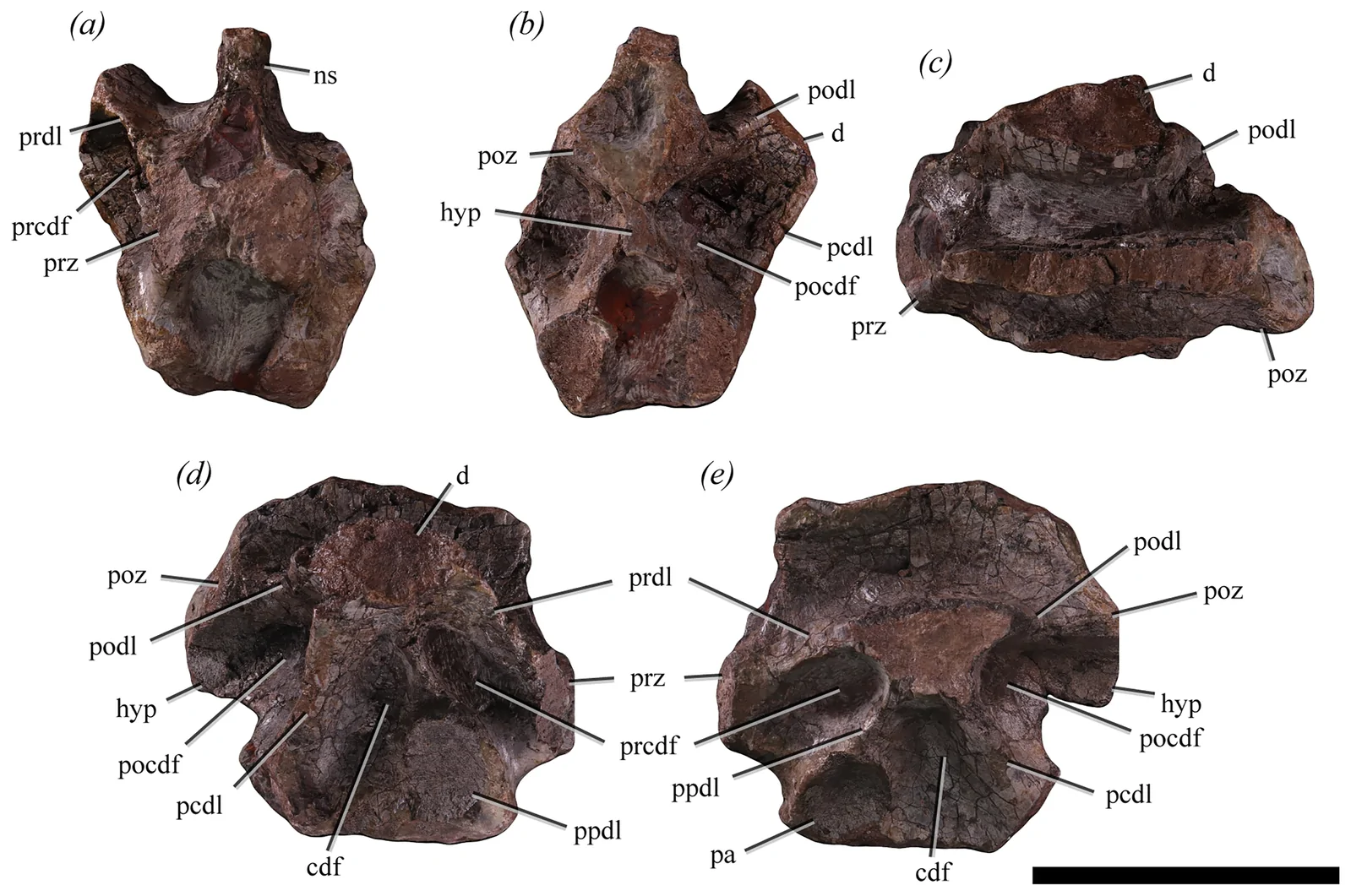
Posterior dorsal neural arch of BP/1/8468 in (*a*) anterior, (*b*) posterior, (*c*) dorsal, (*d*) right lateral and (*e*) left lateral views. Abbreviations: cdf, centrodiapophyseal fossa; d, diapophysis; hyp, hyposphene; ns, neural spine; pcdl, posterior centrodiapophyseal lamina; pocdf, postzygocentrodiapophyseal fossa; podl, postzygodiapophyseal lamina; ppdl, paradiapophyseal lamina; prcdf, prezygocentrodiapophyseal fossa; prdl, prezygodiapophyseal lamina; poz, postzygapophysis; prz, prezygapophysis. Scale bar = 5 cm.

**Figure 52.**
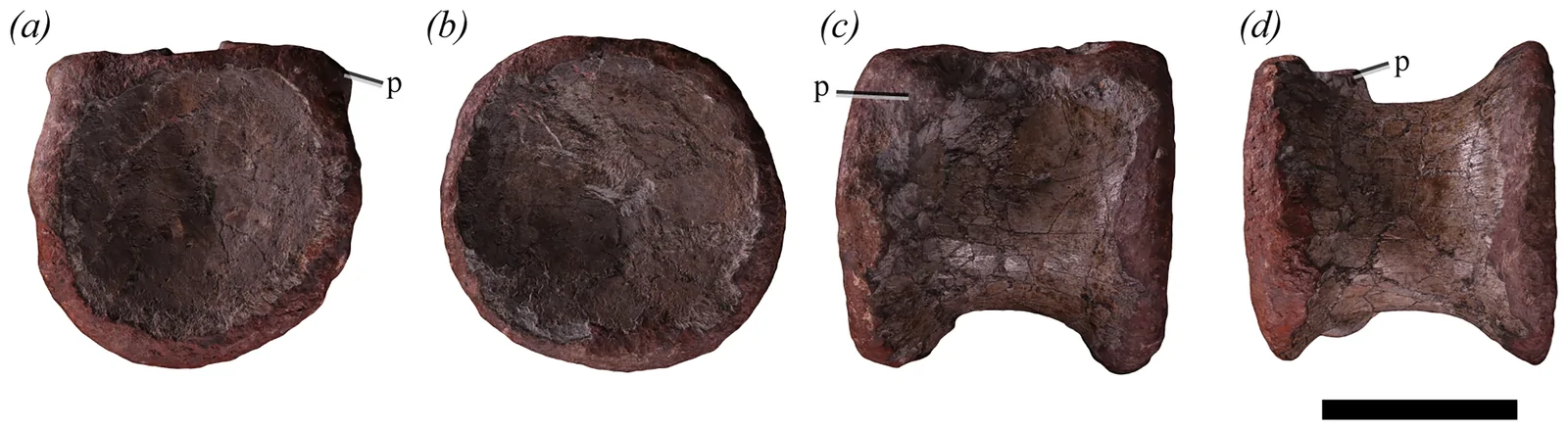
Sacral centrum of BP/1/8468 in (*a*) anterior, (*b*) posterior, (*c*) left lateral and (*d*) ventral views. Abbreviations: p, protuberance. Scale bar = 5 cm.

**Figure 53.**
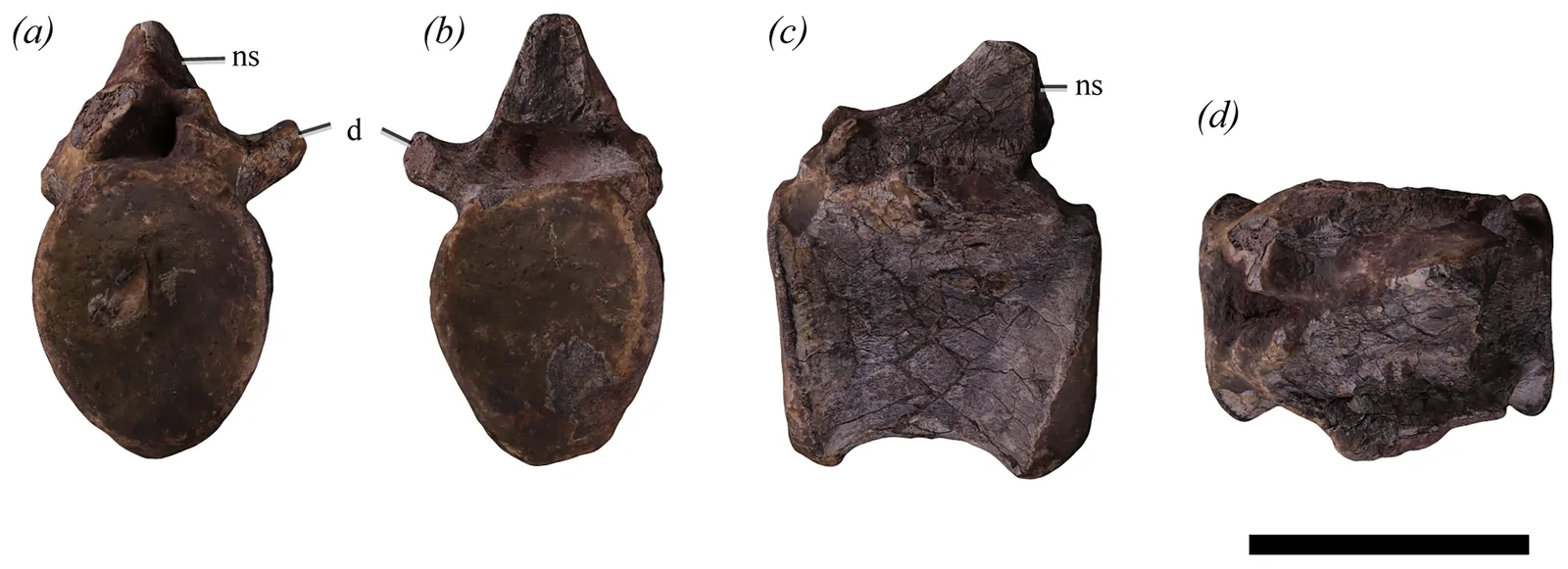
Middle-to-posterior caudal of BP/1/8468 in (*a*) anterior, (*b*) posterior, (*c*) left lateral and (*d*) dorsal views. Abbreviations: d, diapophysis; ns, neural spine. Scale bar = 5 cm.

**Figure 54.**
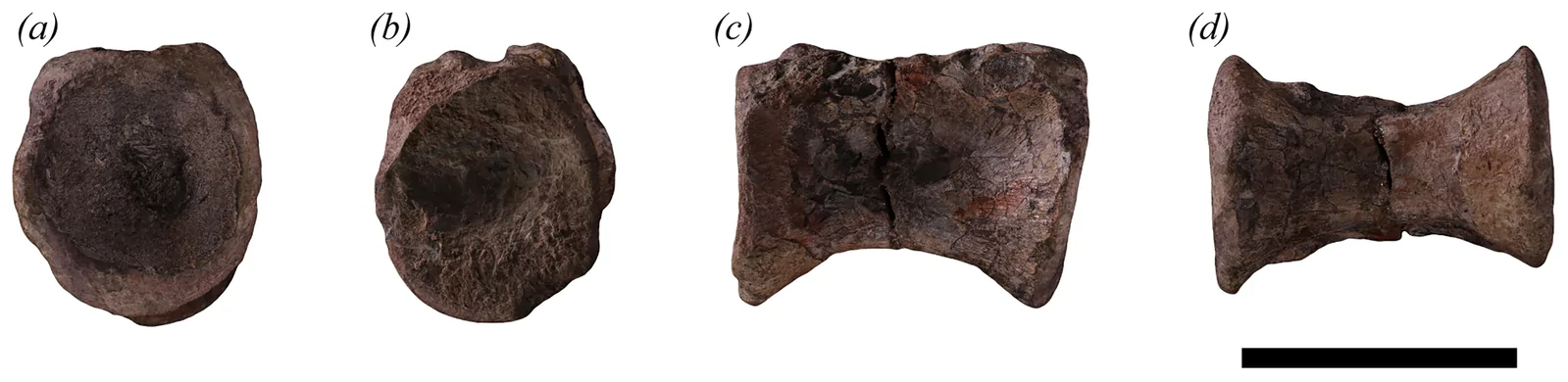
Posterior caudal of BP/1/8468 in (*a*) anterior, (*b*) posterior, (*c*) right lateral and (*d*) ventral views. Scale bar = 5 cm.

**Figure 55.**
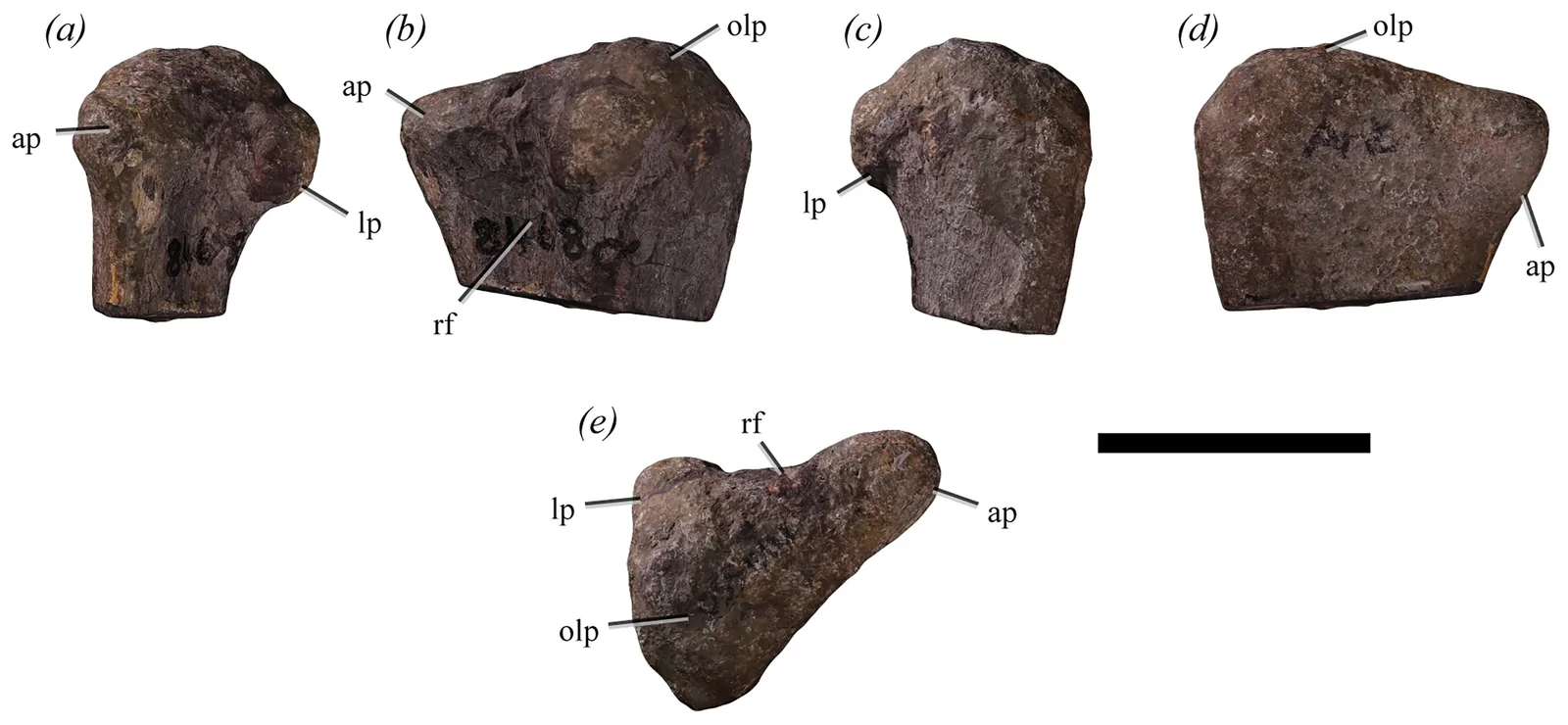
Left ulna of BP/1/8468 in (*a*) anterior, (*b*) lateral, (*c*) posterior, (*d*) medial and (*e*) proximal views. Abbreviations: ap, anterior process; lp, lateral process; olp, olecranon process; rf, radial fossa. Scale bar = 5 cm.

**Figure 56.**
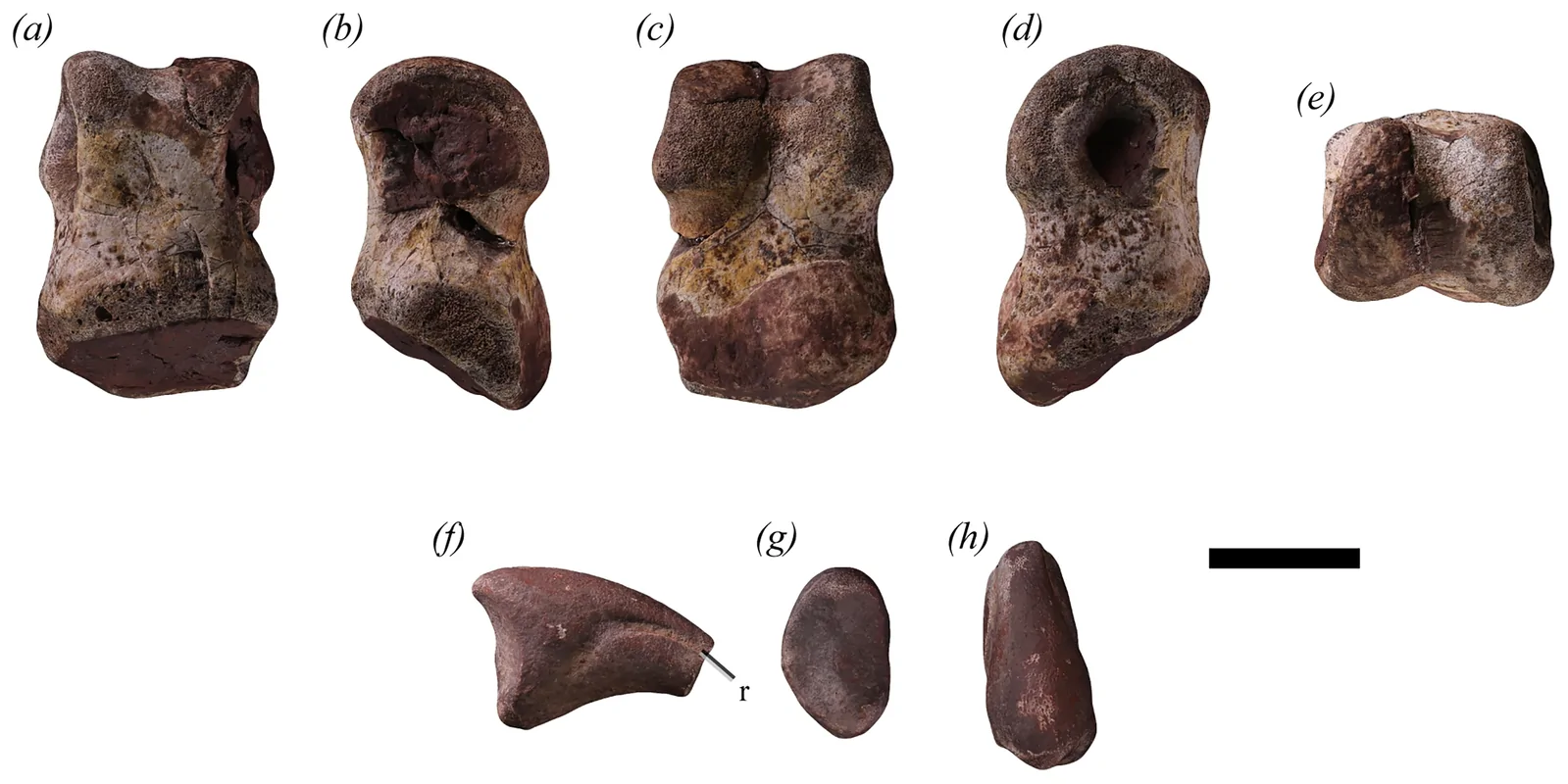
Manual elements of BP/1/8468. The manual phalanx in (*a*) dorsal, (*b*) right lateral, (*c*) ventral, (*d*) left lateral and (*e*) distal views. The manual ungual in (*f*) right lateral, (*g*) proximal and (*h*) dorsal views. Abbreviations: r, ridge. Scale bar = 2 cm.

**Figure 57.**
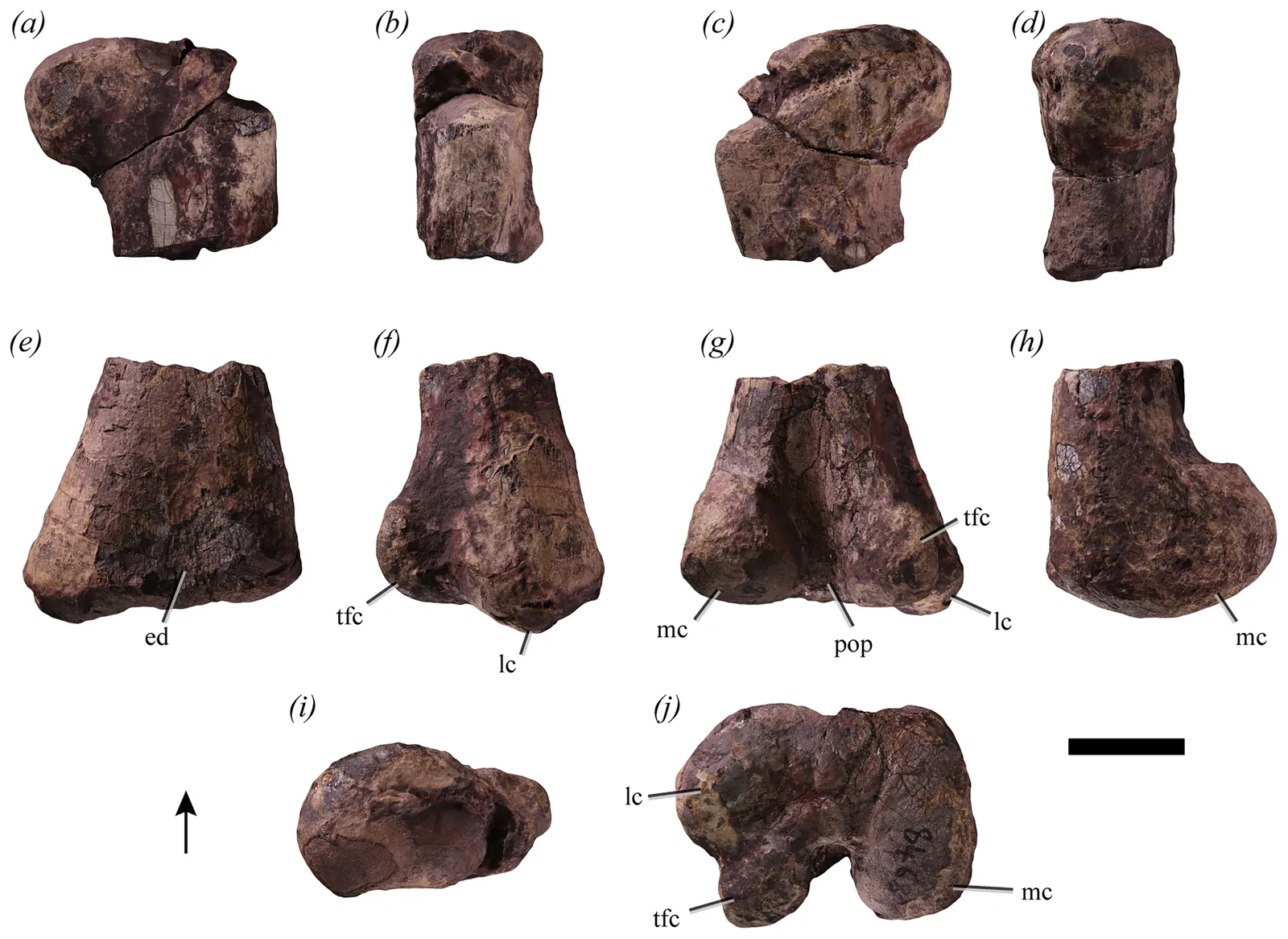
Right proximal and distal femur of BP/1/8468 in (*a,e*) anterior, (*b,f*) lateral, (*c,g*) posterior, (*d,h*) medial and (*i,j*) proximal views. Abbreviations: ed, extensor depression; lc, lateral condyle; mc, medial condyle; pop, popliteal fossa; tfc, tibiofibular crest. Arrow is pointed anteriorly. Scale bar = 5 cm.

**Figure 58.**
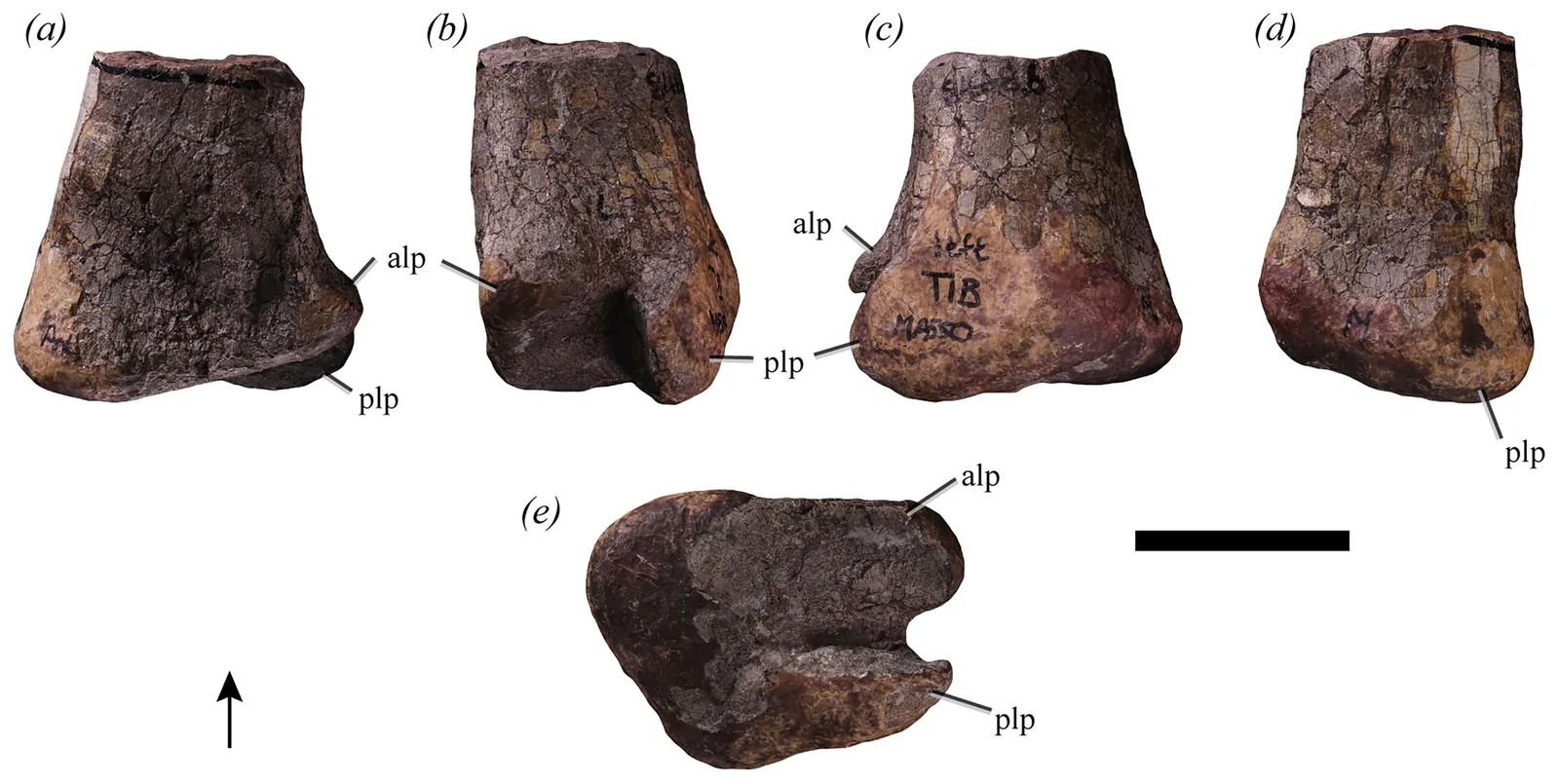
Distal end of the left tibia of BP/1/8468 in (*a*) anterior, (*b*) lateral, (*c*) posterior, (*d*) medial and (*e*) proximal views. Abbreviations: alp, anterolateral process; plp, posterolateral process. Arrow is pointed anteriorly. Scale bar = 5 cm.

**Figure 59.**
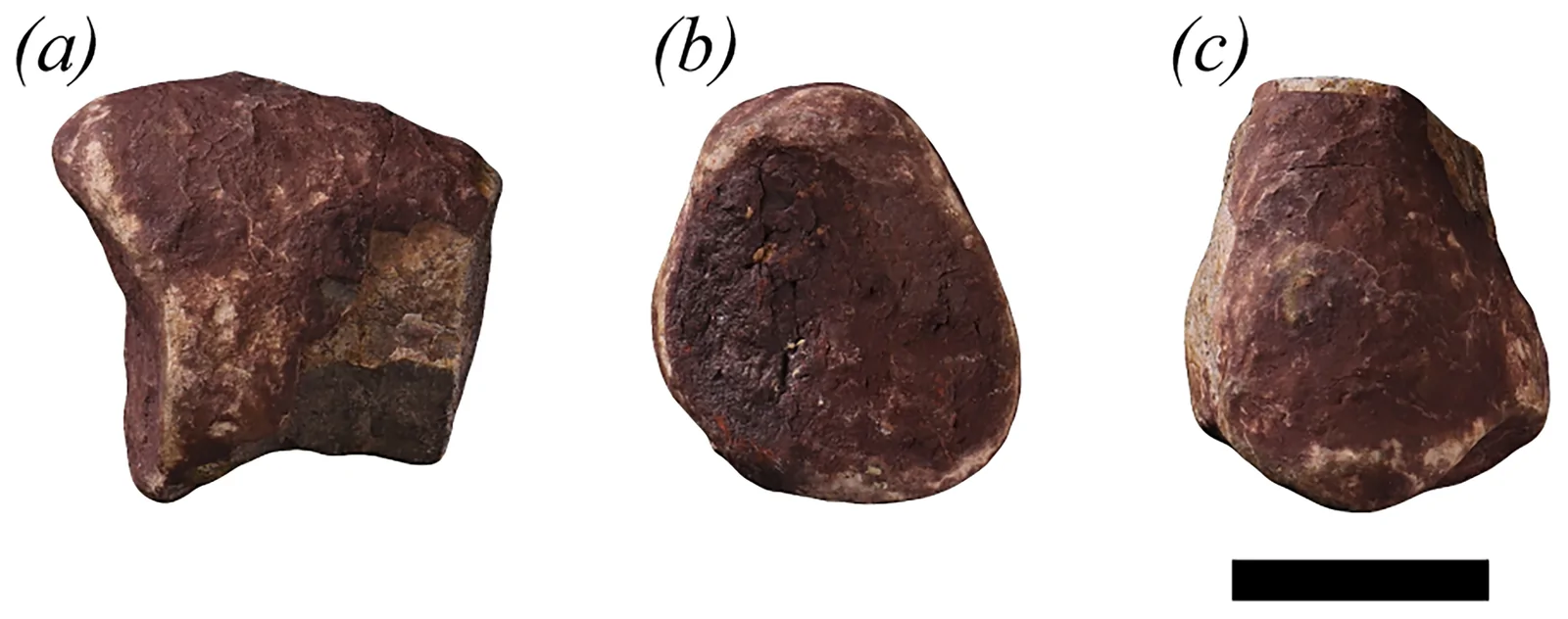
Pedal ungual of BP/1/8468 in (*a*) right lateral, (*b*) proximal and (*c*) dorsal views. Scale bar = 2 cm.

#### 5.9.1. Proatlas

Both proatlases are preserved in close association with each other but are not in life position (figure 42). They lie lateral to the basioccipital condyle and are dorsoventrally flattened, triangular discs that form part of the atlas–axis complex. The long axis of the proatlas is oriented anteromedially to posterolaterally with its apex directed ventrally. The medial margin is the longest and thinnest region of the proatlas and mediolaterally expands towards the apex. The postzygapophyses are located on their posteromedial surfaces and are weakly concave. The anterior margins are rounded with smooth surfaces and no distinct features.

#### 5.9.2. Atlantal neural arches

Both atlantal neural arches are partially preserved and out of life position (figure 43). They would have been positioned dorsolateral to the occipital condyle, dorsal to the atlantal intercentrum and between the proatlas and axis. Both neural arches lack postzygapophyses. The left neural arch consists of the pedicel, and the right neural arch includes the pedicel and prezygapophysis.

The pedicel of the atlantal neural arch projects anteromedially to articulate with the intercentrum and the occipital condyle. The articular surface of the pedicel is expanded as it extends medially from a small shaft and is bean-shaped in medial view. The prezygapophysis is positioned on the lateral surface of the neural arch and extends dorsally into a thin, anteroposteriorly expanded fan-shaped process. It articulates with the postzygapophysis on the proatlas. Its lateral surface is convex, and its medial surface is concave.

#### 5.9.3. Atlantal intercentrum

A partial atlantal intercentrum is preserved in life position (figure 44). The right half is preserved. The intercentrum would have been situated ventral to the occipital condyle and articulated with the pedicels of the atlantal neural arches lateral to the condyle. Its anterodorsal surface is concave and smooth. A small foramen is located on the ventral portion of the articular surface. The lateral margin is rugose for the articulation with atlantal neural arch pedicel. The intercentrum is triangular in lateral view with its apex directed posteroventrally.

#### 5.9.4. Cervical vertebra 4

A nearly complete anterior cervical 4 (figure 45) is preserved missing its neural spine, the distal ends of its pre- and postzygapophyses and the ventral portion of the posterior centrum surface. Vertebral laminae are absent.

The centrum is amphicoelous and hourglass-shaped in ventral view. The articular surfaces of the centrum are mediolaterally wider than they are dorsoventrally tall. The centrum is anteroposteriorly longer than it is dorsoventrally tall with a length-to-height ratio of 2.6. This is much lower than the ratio in the elongated cervicals of massospondylids (*Massospondylus*, approx. 7.6; *Adeopapposaurus*, approx. 5.0; *Leyesaurus*, approx. 5.0; *Lufengosaurus*, approx. 3.3; *Coloradisaurus*, approx. 3.8; *Ngwevu*, approx. 7.15), but similar to other EBSMs. A ventral keel is present on the anterior portion of the centrum but does not extend to the posterior end. The ventral margin of the centrum is concave in lateral view.

The diapophysis is positioned posterior to the anterior margin of the centrum and is located posterodorsally to the parapophysis. The diapophyses project anterolaterally to form a wing-like postdiapophyseal flange. Distinct parapophyses are present on the anterolateral corners of the centrum. They form circular protuberances that are twice as large as the diapophysis. The proximal portion of a cervical rib is preserved in articulation with the left parapophysis.

The bases of the postzygapophyses have a dorsal elevation of approximately 20° from the horizontal plane. An anteroposteriorly deep spinopostzygapophyseal fossa is present at the base of the neural spine.

#### 5.9.5. Cervical vertebrae 6 and 7

Both of these middle cervical vertebrae are nearly complete, missing only the distal ends of their pre- and postzygapophyses. Cv 7 also lacks its neural spine. Cv 6 displays weak shearing along the dorsoventral axis and is best observed in posterior view. Both cervical vertebrae lack vertebral laminae. The following description applies to both Cv 6 (figure 46) and Cv 7 (figure 47) with differences specified where necessary.

The centrum is amphicoelous. Its anterior articular surface is mediolaterally wider than dorsoventrally tall, and its posterior articular surface is circular with subequal dimensions. This differs from other taxa that possess dorsoventrally tall middle cervical centra, such as *Melanorosaurus* and *Kholumolumo*. A ventral keel is present on the anterior portion of the centrum. The centrum has an hourglass-shaped outline in ventral view and a concave ventral margin in lateral view. The neural spine of Cv 6 is anteroposteriorly longer than its dorsoventral height and is rectangular in lateral view. The neural spine maintains the same mediolateral width throughout its height and has a straight dorsal margin.

In Cv 6, the diapophysis is located near the anterior margin of the centrum and posterodorsal to the parapophysis. In Cv 7, the diapophyses are located posterodorsally further onto the neural arch and flare laterally. The distal ends of the diapophyses are anteroposteriorly elongated and dorsoventrally flattened, resulting in an elliptical outline in both vertebrae. In Cv 7, a postdiapophyseal flange connects the posterior end of diapophysis to the centrum with shallow, anteroposteriorly elongated depressions ventral to this flange. The parapophyses are distinct from the diapophyses and are located on anterolateral corners of both centra. They have elliptical articular facets that are larger than the diapophyses.

#### 5.9.6. Cervical vertebra 9

A nearly complete posterior cervical vertebra (figure 48) is preserved missing its neural spine, the distal end of the diapophyses, the distal ends of the prezygapophyses and the distal end of the right postzygapophysis.

The centrum is amphicoelous, and both articular surfaces are mediolaterally wider than they are dorsoventrally tall. A ventral keel is present only on the anterior and posterior portions of the centrum in *Nyana* but does not extend for the entire centrum length compared to *Ngwevu*, *Adeopapposaurus* and *Leyesaurus*. The centrum has a concave ventral margin in lateral view and is hourglass-shaped in ventral view.

The diapophyses are located near the midline on the neural arch and contribute to the prezygadiapophyseal, anterior centrodiapophyseal and posterior centrodiapophyseal laminae. These laminae are prominent and distinct in Cv 9 of *Nyana*, whereas vertebral lamination of Cv 9 is weak or absent in *Massospondylus*, *Ngwevu* and *Sarahsaurus*. The postzygodiapophyseal laminae are weakly developed, obscured or not present in this vertebra. The parapophyses are distinct elliptical protuberances located at mid-height on the lateral surface and at the anterior margin of the centrum.

At the base of the prezygapophyses, a deep spinoprezygapophyseal fossa is present. The postzygapophyses are directed posterodorsally and laterally. They are directed approximately 30° dorsally from the horizontal in lateral view. A deep spinopostzygapophyseal fossa is buttressed by the base of the postzygapophyses. The postzygapophyseal articular facets are ovoid and are directed ventrolaterally. In lateral view, the postzygapophyses extend posteriorly beyond the margin of the centrum.

#### 5.9.7. Cervicodorsal vertebra

A nearly complete cervicodorsal vertebra (figure 49) is preserved missing its neural spine, the distal ends of the prezygapophyses, its postzygapophyses and the distal end of the right diapophysis. This vertebra is a cervicodorsal based on the positions of the parapophyses and diapophyses (see below), including the presence of diapophyseal laminae. The proximal end of a rib is preserved ventral to the left diapophysis, obscuring any laminae.

The centrum is weakly amphicoelous. The anterior articular surface is dorsoventrally taller than mediolaterally wide, and its posterior articular surface is mediolaterally wider than dorsoventrally tall. It has a concave ventral margin and is hourglass-shaped in ventral view. The centrodiapophyseal fossa is circular and shallow and located on the lateral surfaces of the centrum located ventral to the diapophyses. A prominent ventral keel is present along almost the entire ventral margin of the centrum but does not extend to the posterior margin. This is similar to the condition in *Adeopapposaurus*, whereas *Coloradisaurus* lacks a ventral keel, and *Jingshanosaurus* has a keel that forms a ventrally extending sheet.

The diapophyses are positioned on the neural arch. They are anteroposteriorly expanded at their base and are mediolaterally broad as they extend laterally. The anterior and posterior centrodiapophyseal laminae are prominent. The prezygodiapophyseal and postzygodiapophyseal laminae are well-developed and sheet-like. The distinct prezygodiapophyseal and postzygodiapophyseal laminae are similar to those present in *Coloradisaurus*. The postzygocentrodiapophyseal fossa is deep. Within the prezygocentrodiapophyseal fossa, a weakly developed accessory lamina is present. The parapophysis is located at mid-height on the centrum and one-third of the centrum length from the anterior centrum margin. Its elliptical articular facet is dorsoventrally tall and larger than those of the cervical vertebrae.

#### 5.9.8. Anterior-to-middle dorsal vertebra

An anterior-to-middle dorsal vertebra (figure 50) is preserved missing its neural spine, prezygapophyses, portions of the distal ends of the postzygapophyses and the diapophyses.

The centrum is amphicoelous, and the articular surfaces are rounded. It is slightly anteroposteriorly longer than its dorsoventral height. A shallow, dorsoventrally tall centrodiapophyseal fossa is present on its lateral surface ventral to the diapophysis. The centrum is spool-shaped in ventral view and has a concave ventral margin in lateral view, similar to that of *Unaysaurus*.

Anterior centrodiapophyseal, posterior centrodiapophyseal and postzygodiapophyseal laminae are present. An accessory lamina lies within the postzygocentrodiapophyseal fossa. The parapophysis lies on the anterolateral corner of the neural arch but is largely obscured by matrix.

The base of the postzygapophyses extends posteriorly, and a thin, vertical hyposphene is present ventral to the postzygapophyses. The postzygapophyseal articular facets are directed ventrally based on the base of the postzygapophyses. Although incomplete, the postzygapophyses extend further posteriorly than the extent of the centrum in lateral view.

#### 5.9.9. Posterior dorsal neural arch

A neural arch (figure 51) from the posterior dorsal series is preserved missing its neural spine, diapophyses, prezygapophyses and the distal ends of the postzygapophyses.

The base of the left diapophysis extends laterally and is angled dorsally owing to deformation. Prezygadiapophyseal, paradiapophyseal, posterior centrodiapophyseal and postzygodiapophyseal laminae are well-developed and sheet-like. The postzygodiapophyseal laminae are the largest. The development of the laminae is similar to that seen in *Melanorosaurus*, *Kholumolumo*, *Ledumahadi* and *Antetonitrus*. The laminae help define a series of deep prezygocentrodiapophyseal, centrodiapophyseal and postzygocentrodiapophyseal fossae. A weakly developed accessory lamina divides the postzygocentrodiapophyseal fossa. The parapophyses are located on the anteroventral corner of the neural arch and posterior to the prezygapophyses. They are large, ovoid facets with a distinct rim along their dorsal margin.

The postzygapophysis extends posteriorly, and the articular facets are directed ventrolaterally. They are joined ventromedially by a small, mediolaterally thin hyposphene.

#### 5.9.10. Primordial sacral vertebra 1

A sacral vertebral centrum (figure 52) is preserved missing its neural arch. It is probably the first primordial sacral vertebra based on the position of its possible rib facets, which protrude from the anterodorsal corners of its lateral surfaces, whereas a dorsosacral would be near the midline and the second primordial sacral would be posteriorly located. The centrum is amphicoelous. It is anteroposteriorly short and has rounded anterior and posterior articular surfaces. The centrum is smooth with circular articular faces.

#### 5.9.11. Middle-to-posterior caudal vertebra

The centrum of a middle-to-posterior caudal vertebra (figure 53) is preserved, missing most of its neural spine, zygapophyses and diapophyses.

The centrum is amphicoelous, and both articular surfaces are dorsoventrally taller than they are mediolaterally wide. The articular faces are ovoid in outline. Chevron facets are absent. The base of the neural spine is directed posterodorsally. The bases of the diapophyses are directed dorsolaterally.

#### 5.9.12. Posterior caudal vertebra

A posterior caudal centrum (figure 54) is preserved. It is amphicoelous with subrectangular articular surfaces that are dorsoventrally taller than they are mediolaterally wide. The centrum is anteroposteriorly longer than dorsoventrally tall. An anteroposteriorly long depression of moderate depth occupies the lateral surfaces of the centrum. The ventral margin of the centrum is flat. Poorly developed chevron facets are present posteroventrally.

#### 5.9.13. Ulna

The proximal end of a left ulna is preserved (figure 55). The olecranon process is weakly developed. The medial margin of the ulna is straight, its lateral margin is convex, and its anterior margin is concave in proximal view. The lateral process is a small protuberance that extends laterally. The anterior process extends anteriorly and is almost half the length of the proximal end in proximal view. The radial fossa is shallow. The proximal ulna differs from that of *Unaysaurus* and *Massospondylus*, which has an anteroposteriorly elongated proximal ulna, but is somewhat similar to those of *Adeopapposaurus*, *Plateosauravus* and *Melanorosaurus* as it is proportionally wider mediolaterally and subtriangular in proximal view.

#### 5.9.14. Manual phalanx

A manual phalanx (figure 56*a*–*e*) is preserved, but it is unclear which digit it belongs to. The proximal end is gently twisted and offset from the horizontal in proximal view. The distal articular condyles are weakly asymmetrical with the right condyle larger than the left; this can be seen clearer in ventral view. The condyles are separated by an intercondylar groove. Each ginglymus bears a collateral ligament fossa.

#### 5.9.15. Manual ungual

A manual ungual (figure 56*f*–*h*) is preserved missing its distal end. It is unclear which digit it belongs to. It is recurved and has clear ridges for the attachment grooves on its lateral surfaces. Its proximal surface is dorsoventrally taller than its mediolateral width.

#### 5.9.16. Femur

The femoral head and distal end of a right femur (figure 57) are preserved. The femoral head has a weakly developed posterior tubercle and a convex dorsal margin. The ventral margin of the femoral head in lateral view is proportionally mediolaterally shorter than that of *Sarahsaurus* and *Yunnanosaurus*. The greater trochanter, although broken, is weakly developed.

The distal end is subrectangular in distal view. The medial condyle of the distal end is anteroposteriorly longer than its mediolateral width. The tibiofibular crest is subrectangular in distal view and is shorter in all of its dimensions relative to the lateral condyle. This differs from the tibiofibular crest of *Adeopapposaurus* and *Sarahsaurus* in which it is posteriorly pointed in distal view. The lateral condyle extends laterally with a convex lateral margin. The anteroposterior length of the medial condyle is approximately the same as the combined anteroposterior length of the tibiofibular crest and lateral condyle. The medial and lateral condyles are separated by an anteroposteriorly deep popliteal fossa. The extensor depression on the anterior surface is shallow.

#### 5.9.17. Tibia

The distal end of the left tibia (figure 58) is preserved. The anterior margin of the distal end is mediolaterally wider than its posterior margin, resulting in a subtrapezoidal cross-section in distal view. The anterolateral process is anteroposteriorly wider than the posterolateral process in distal view. This is similar to *Yunnanosaurus*, *Sarahsaurus* and *Adeopapposaurus*, as well as other lEF taxa. The anterolateral and posterolateral processes extend laterally to the same extent in anterior or posterior view. An anteroposteriorly narrow furrow separates the anterolateral and posterolateral processes and is best observed in lateral or proximal view.

#### 5.9.18. Pedal ungual

A left pedal ungual (figure 59) is preserved. It is unclear which digit it belonged to and is missing its distal end. The proximal end is weakly asymmetrical and subtriangular in proximal view, and its apex is directed laterally in dorsal view, and it has a flat ventral surface. It is unclear if attachment grooves were present or not on its lateral surfaces.

## 6. Osteohistology

### 6.1. Ulna

The ulna (figure 60) has a large medullary cavity surrounded by a thin cortex. The middle of the medullary cavity is open, but the rest of this region is filled with bony trabeculae, many of which are broken into fragments. Secondary remodelling, evidenced by the presence of secondary osteons and numerous resorption cavities, is observed in the inner cortex. Woven bone containing numerous, oval, haphazardly arranged osteocyte lacunae in association with numerous primary osteons throughout the cortex indicates that the bone tissue is a rapidly forming woven-parallel complex (WPC). There is a combination of vascular arrangements throughout the cortex from longitudinally oriented primary osteons, some with short anastomoses, to patches of laminar vascular canals.

**Figure 60.**
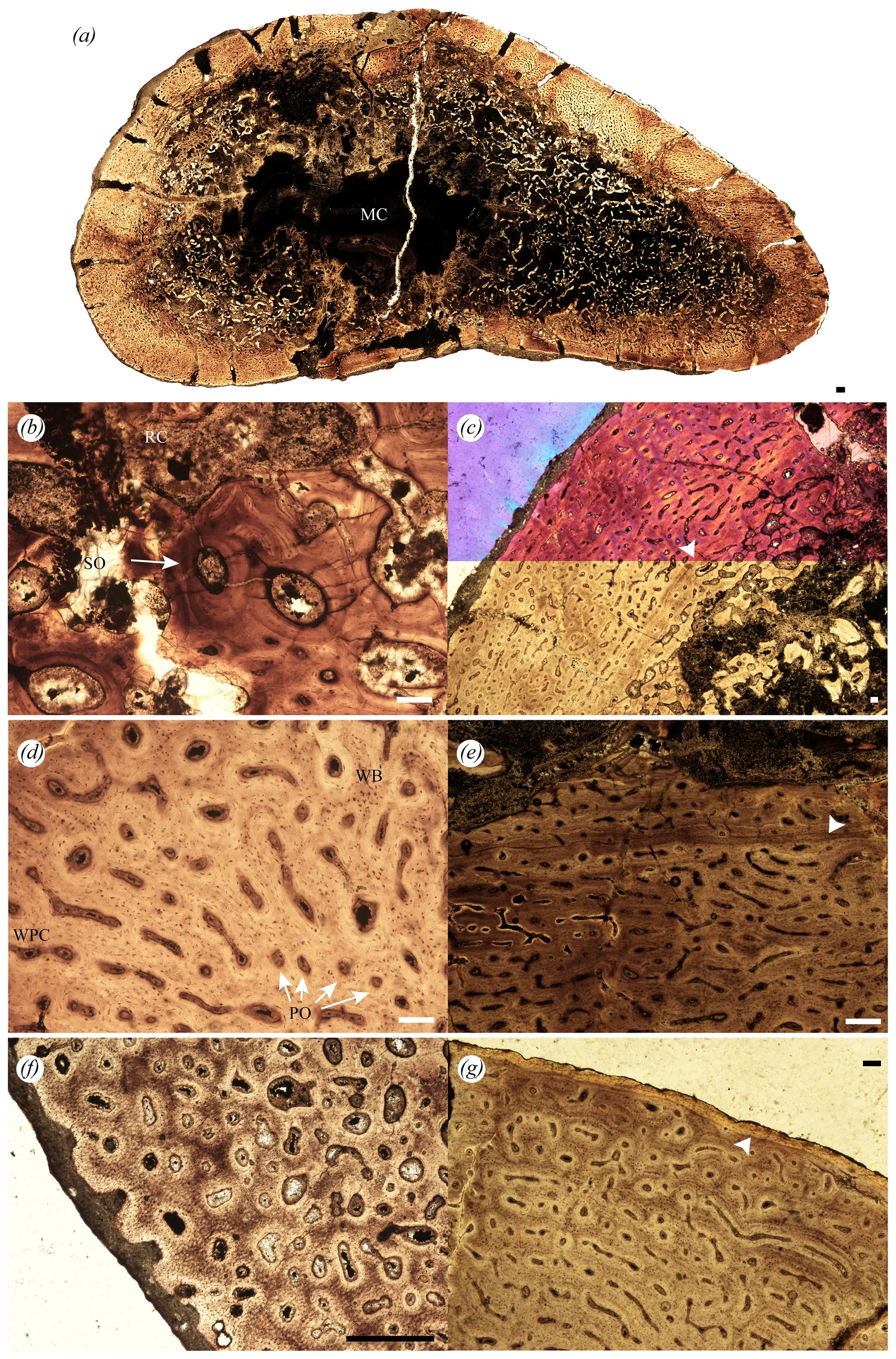
BP/1/8468 ulnar osteohistology. (*a*) Overall transverse section showing the many trabeculae. (*b*) Secondary osteons in the inner cortex. (*c*) Overall cortex showing the resorption cavities, a LAG in the inner cortex and the vascular arrangement in normal and cross-polarized light. (*d*) Close-up showing the woven-parallel complex. (*e*) Close-up of the LAG in the inner cortex. (*f*) Close-up of the crenulated subperiosteal surface. (*g*) The possible second LAG close to the subperiosteal surface. Arrowhead indicates the LAG. Abbreviations: MC, medullary cavity; PO, primary osteon; RC, resorption cavity; SO, secondary osteon; WB, woven bone; WPC, woven-parallel complex. Scale bars: (*a,e,f*) = 500 μm, (*b,c,d,e,g*) = 100 μm.

There is no evidence for decreased vascularization towards the outer cortex. Regions along the subperiosteal surface are crenulated indicating that the animal was still growing at the time of death. A growth mark (GM) in the form of a line of arrested growth (LAG) interrupts the inner cortex. Another growth line runs along the subperiosteal surface (figure 60*g*), but only for a short distance, making it difficult to definitively confirm it as a second LAG. The inner LAG splits in places but merges in others and is considered to represent one season. Outer circumferential lamellae (OCL; also known as an external fundamental system) are absent.

### 6.2. Tibia

The tibia (figure 61) has a large, open medullary cavity surrounded by a thin cortex. The subperiosteal surface is poorly preserved. Secondary remodelling (resorption cavities and secondary osteons) is observed in the inner cortex, with some of the secondary osteons overlapping, indicating two generations. A small region of compacted coarse cancellous bone is present in the inner cortex. The bone tissue varies between a WPC and parallel-fibred bone (PFB) with an increase in PFB towards the outer cortex. Large areas in the mid-to-outer cortex comprise numerous, haphazardly arranged osteocyte lacunae with primary osteons. However, there are also large regions in the mid-cortex where woven bone is absent but which contain osteocyte lacunae that are less randomly arranged. Although there are small patches of other arrangements, the vascular canals form a laminar arrangement throughout most of the cortex.

**Figure 61.**
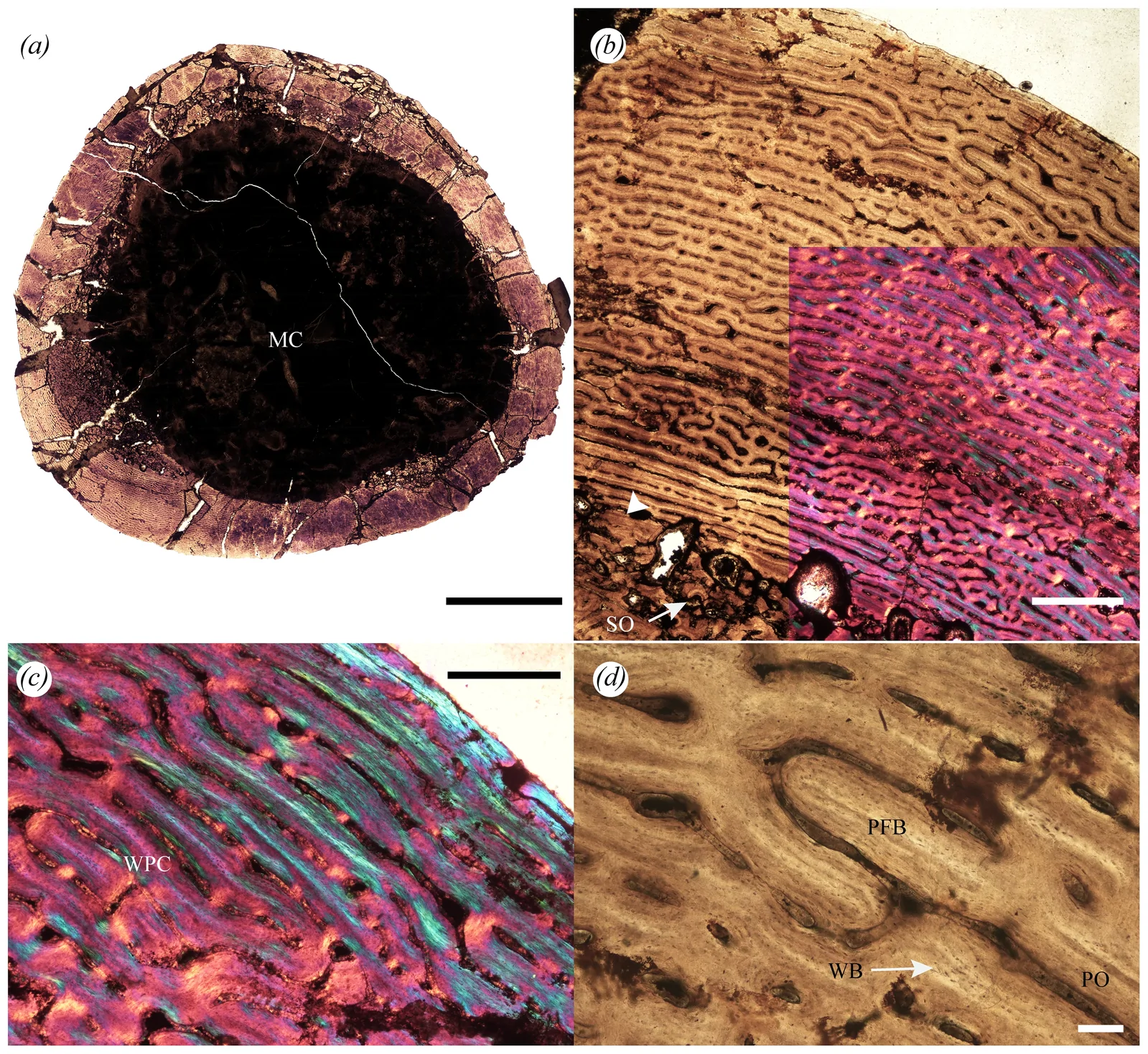
BP/1/8468 tibial osteohistology. (*a*) Overall transverse section. (*b*) A column through the cortex showing the LAG in the inner cortex and the vascular arrangement in normal light and cross-polarized light. (*c*) A close-up of the outer cortex in cross-polarized light showing the woven-parallel complex. (*d*) A close-up of the osteocytes in the outer cortex. Arrowhead indicates the LAG. Abbreviations: MC, medullary cavity; PFB, parallel-fibred bone; PO, primary osteon; SO, secondary osteon; WB, woven bone; WPC, woven-parallel complex. Scale bars: (*a*) = 10 mm, (*b*) = 1000 μm, (*c*) = 500 μm, (*d*) = 100 μm.

One very faint LAG is present in the inner cortex. Similar to the ulna, the cortex of the tibia displays a high vascular density throughout the cortex to the subperiosteal surface, further supporting the conclusion that the individual was actively growing at the time of death.

## 7. Phylogenetic analysis

The initial maximum parsimony (MP) analysis resulted in six most parsimonious trees (MPTs), length = 1741 steps. The full search resulted in ≥ 10 000 MPTs (tree-storage overflow). These MPTs have a Consistency Index of 0.28 and a Retention Index of 0.63. Bremer and bootstrap support values are low for most nodes, with Bremer support values ranging from 1 to 3 and bootstrap support values highest for *Pantydraco* + *Thecodontosaurus* (70%). The strict consensus tree (see supplementary material, S1) is poorly resolved with no resolution among EBSMs. Application of iterPCR identified multiple taxa as having multiple most parsimonious positions among the MPTs. *A posteriori* removal of some of these taxa (*Schleitheimia*, *Blikanasaurus* and *Tuebingosaurus*) produced a better-resolved reduced majority-rule consensus topology (figure 62) that we describe here. In these resulting reduced trees, *N. rooiwalensis* is variably recovered as either a sister to *Sarahsaurus* and nested within Massospondylidae (figures 62 and 63*a*), a sister to *Ngwevu* and nested within Massospondylidae (figure 63*b*) or a sister to *Sarahsaurus* as an early-branching massopodan outside of Massospondylidae (figure 63*c*).

**Figure 62.**
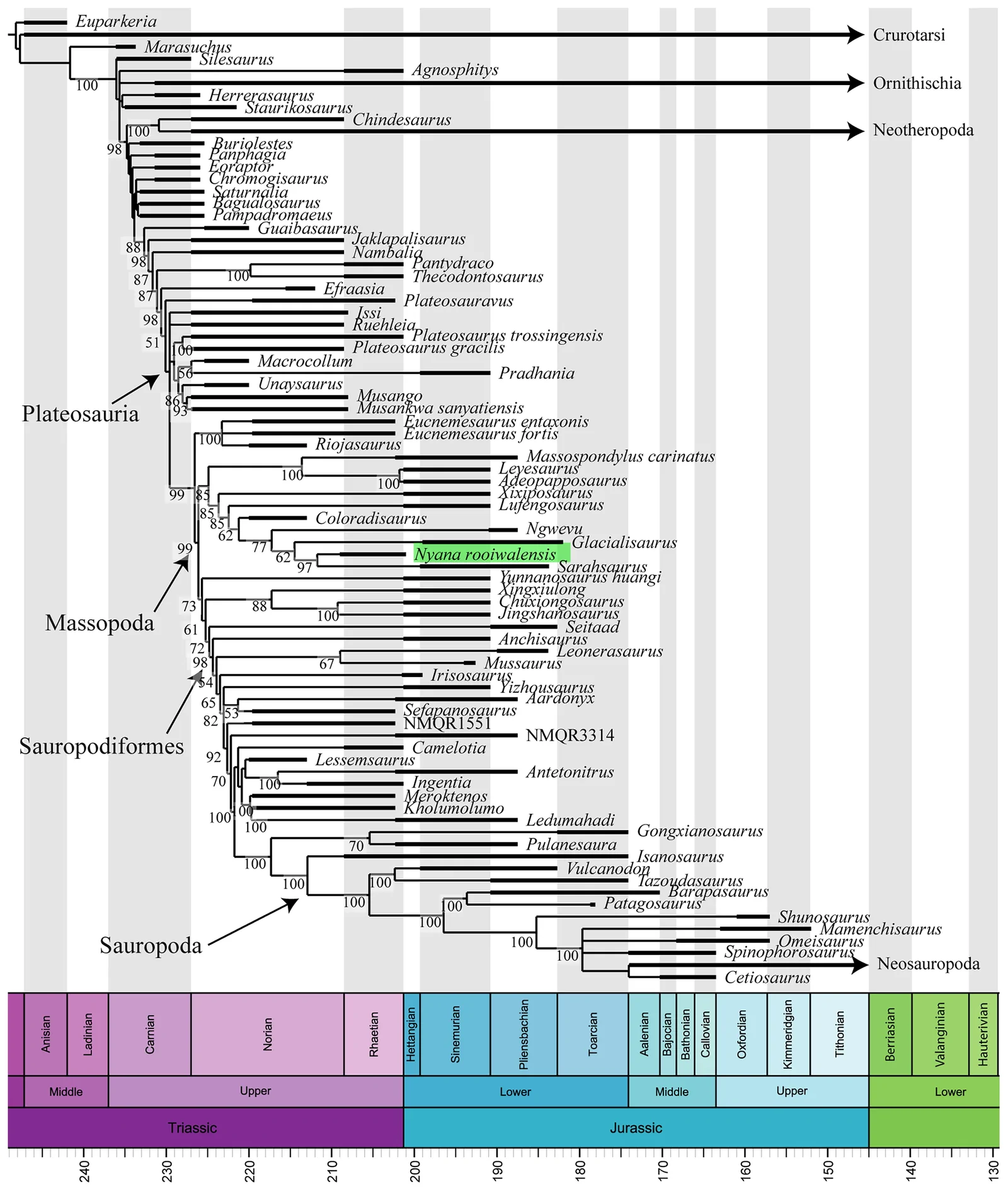
Time-calibrated reduced majority-rule consensus tree depicting the position of BP/1/8468. Numbers at nodes indicate the percentage for which the node was recovered across the most parsimonious trees.

**Figure 63.**
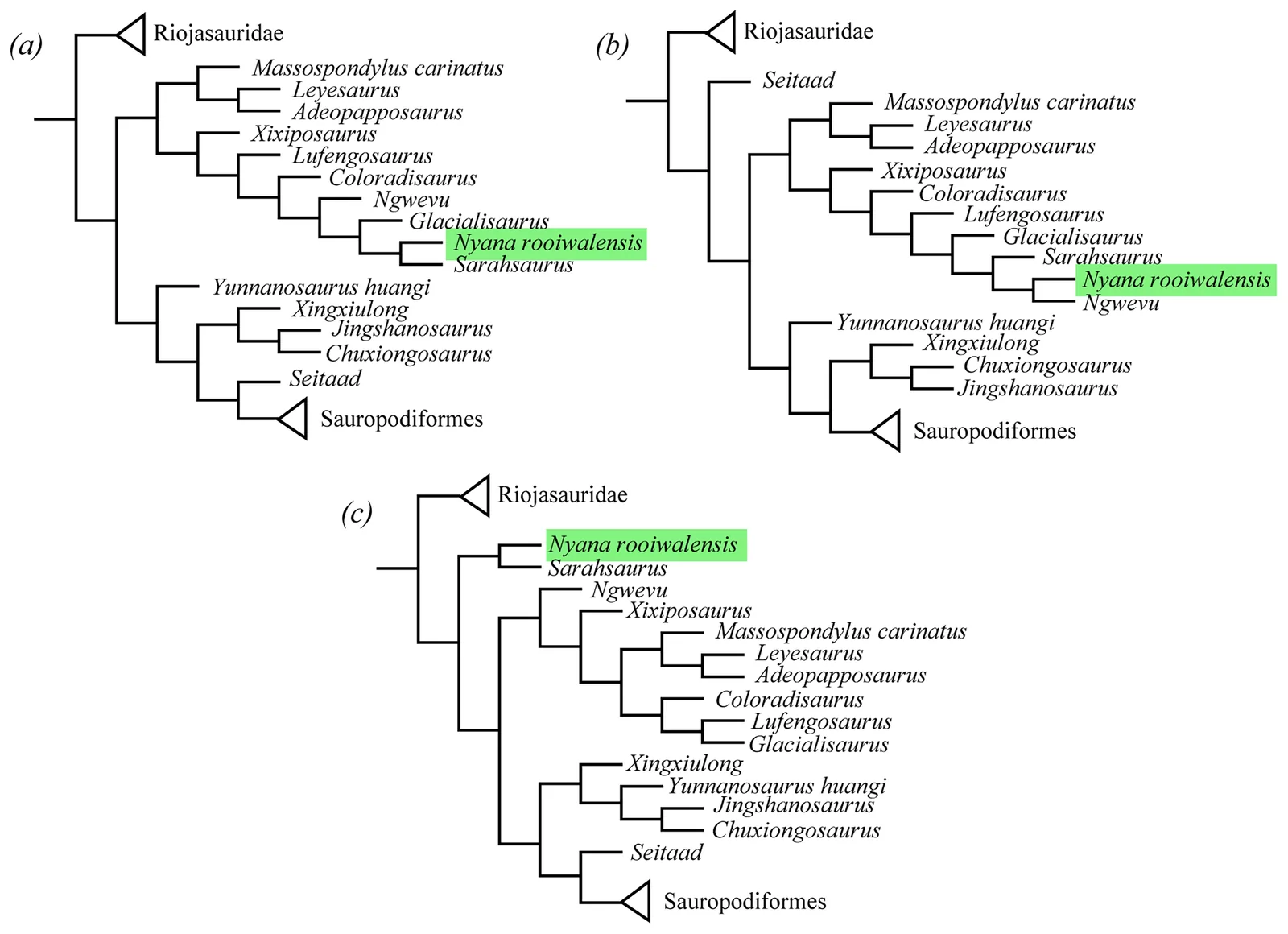
Alternative positions of BP/1/8468 (*a,b*) within Massospondylidae and (*c*) as an early-branching sauropodomorph.

The sister taxon relationship between *Nyana* and *Sarahsaurus* is supported by (character and state numbers indicated in parentheses): similarly sized neurovascular foramina in the lateral maxillary row (33:0; also present in *Herrerasaurus*, *Buriolestes* and *Shunosaurus*); the dorsal margin of the postorbital is straight to gently curved in lateral view (54:0; also present in *Pantydraco*, *Thecodontosaurus*, *Plateosaurus gracilis* and *Plateosaurus engelhardti*); the supraoccipital is semilunate and wider than high (75:1; a common feature across sauropodomorphs); the paroccipital processes are oriented slightly dorsolaterally to horizontal in occipital view (77:0; also present in *Ngwevu*); the parapophyses in the first two dorsals are located at the anterior end of the centrum (153:0; also present in *Saturnalia* and later diverging sauropodomorphs such as *Anchisaurus*, *Mussaurus*, *Leonerasaurus* and some sauropods); the transverse width of the distal tibia is subequal its anteroposterior length (306:0; also present in later diverging taxa such as *Kholumolumo*, *Pulanesaura* and *Tazoudasaurus*); the distal surface of the tibiofibular crest is deep anteroposteriorly as it is wide mediolaterally (355:0; common across sauropodomorphs); and the forked distal end of the frontal ramus of the postorbital into parietal and frontal processes separated by a distinct notch (405:1; also present in *Macrocollum*, *Coloradisaurus*, *Lufengosaurus* and *Ngwevu*).

The massospondylid position of *Nyana* is further supported by the following: the length of quadrate that is occupied by the pterygoid wing is at least 70% (71:0; common across sauropodomorphs); the foramen for the mid-cerebral vein is located on the supraoccipital (73:1; also present in *Panphagia*, *Adeopapposaurus*, *Massospondylus*, *Coloradisaurus* and NMQR 3314); a semilunate supraoccipital that is wider than high (75:1; also present in *Buriolestes*, *Panphagia*, *Eoraptor*, *Thecodontosaurus*, *Pantydraco*, *Ngweuv*, *Sarahsaurus* and *Anchisaurus*); an erect to gently sloping supraoccipital plate (76:0; also present in other massospondylids and *P. trossingensis*); paroccipital processes that are oriented slightly dorsolaterally in occipital view (77:0); the frontal ramus of the laterosphenoid extends medially (414:0); and the distal end of the jugal ramus of the ectopterygoid tapers (415:0).

Characters supporting the earlier-branching position of *Nyana* outside of Massospondylidae include the following: the presence of ventral keels on the anterior cervical centra (132:0) and the poorly defined radial fossa bounded by an anterolateral process on the proximal ulna (214:1). Characters supporting the sister relationship between *Nyana* and *Ngwevu* include the following: the lack of frontal contribution to the supratemporal fenestra (58:1; also present in *Panphagia*, *Adeopapposaurus*, *Yizhousaurus* and *Aardonyx*); the length of the quadratojugal ramus of the squamosal is four times its width at its base (61:0; also present in *Herrerasaurus* and *Staurikosaurus*); and the jugal process of the ectopterygoid is strongly recurved and hood-like (87:1; also present in *Pantydraco*).

## 8. Discussion

### 8.1. *Nyana rooiwalensis* as a new genus and species

*Nyana rooiwalensis* includes the first near-complete dinosaur skull recovered from the Late Triassic of South Africa and one of the earliest from the main Karoo Basin. Its discovery, together with other recently noted material [97–100], highlights the considerable potential of the lEF to yield important new fossils despite its long history of fossil collection [2,101].

Although the skeleton of *Nyana* is incomplete, it shares many features with *Ngwevu*, *Adeopapposaurus*, *Coloradisaurus* and *Sarahsaurus*, consistent with massopodan affinities. However, it differs from these taxa by its uniquely robust postorbital and high number of maxillary alveoli (23). The postorbital forms part of the margins of most major skull openings (orbit, supratemporal and infratemporal fenestrae) and influences their proportions. The angles between the postorbital rami (see supplementary material, S4 for details) in some EBSMs create a rotated ‘Y’-shaped outline in lateral view, with the squamosal ramus directed posteriorly, the ventral margin of the frontal ramus positioned dorsal to the squamosal ramus and the jugal ramus directed ventrally (e.g. *Massospondylus*, *Lufengosaurus*, *Coloradisaurus*, *Adeopapposaurus*, *Plateosaurus*, *Yunnanosaurus* and *Ngwevu*). The postorbital rami in other taxa display a ‘T’-shaped morphology in lateral view, with the squamosal ramus directed posteriorly, the ventral margin of frontal ramus set approximately level to the squamosal ramus and the jugal ramus extending ventrally (e.g. *Sarahsaurus* and *Riojasaurus*). These processes also determine the anterior extent of the infratemporal fenestra relative to the orbit, with the ventral margin of the fenestra either posterior to the orbit (e.g. *Ngwevu* and NMQR 3314) or extending ventral to the orbit (e.g. *Massospondylus* and *Xixiposaurus*). *Nyana* displays the ‘T’-shaped morphology, and the anterior margin of the infratemporal fenestra is posterior to the orbit.

Quantitative comparisons of postorbital rami proportions (mediolateral width vs dorsoventral height or mediolateral width vs anteroposterior height; supplementary material, S4) indicate that the frontal and squamosal rami overlap in robusticity with those of other taxa (e.g. *Sarahsaurus*, *Xixiposaurus*, *Coloradisaurus*, *Riojasaurus* and *Plateosaurus*). However, the jugal ramus of *Nyana* is more robust than in *Plateosaurus*, *Massospondylus*, *Sarahsaurus* and *Ngwevu*. These proportions are similar to those in other massopodan taxa.

The postorbital also changes in shape during ontogeny and across sauropodomorph evolution. Comparisons with taxa included in ontogenetic studies [102] show that *Nyana*’s cranial proportions resemble those of adult *Massospondylus*, juvenile *Anchisaurus* and multiple individuals of *Mussaurus*. As *Nyana* is a juvenile, the morphology of the postorbital and overall cranial shape probably changed later in ontogeny, but current understanding of these character distributions supports this as a distinct taxon.

The maxilla of *Nyana*, although incomplete, preserves 23 alveoli. This count exceeds that of many EBSMs, which typically possess 14–20 alveoli (e.g. *Massospondylus*, *Yunnanosaurus*, *Jingshanosaurus*, *Sarahsaurus*, *Ngwevu*, *Aardonyx*, *Lufengosaurus*, NMQR 3314, *Lufengosaurus* and *Xixiposaurus*). Other taxa with a similarly high maxillary alveoli count include *Coloradisaurus* (22), *Riojasaurus* (24) and *Plateosaurus* (25), but *Nyana* can be differentiated from these taxa based on other cranial features. An increase in tooth count through ontogeny has been reported in taxa like *Massospondylus*, *Plateosaurus*, *Mussaurus* and *Lufengosaurus* [20,22,90,103–105]. As *Nyana* had not reached skeletal maturity and its maxilla is incomplete, it is possible that it had comparable or higher tooth counts to *Plateosaurus*.

Additionally, the inner ear of *Nyana* is anteroposteriorly longer and dorsoventrally shorter relative to the reconstructed semicircular canals of other sauropodomorphs, contrasting with the anteroposteriorly short and dorsoventrally taller semicircular canals of *Massospondylus*, *Sarahsaurus* and later sauropods [68,92,106,107]. This provides additional evidence that *N. rooiwalensis* represents a new taxon.

### 8.2. Ontogenetic growth of *Nyana rooiwalensis*

Multiple lines of evidence indicate a juvenile-to-late-juvenile ontogenetic age for *N. rooiwalensis*. Osteohistology shows active growth at the time of death, indicated by dense cortical vascularization and subperiosteal deposition. At least one clear LAG is present, with a possible second near the subperiosteal surface. Taken together, the thin cortex, high vascular density, crenulated subperiosteal surface, open cranial sutures and absence of vertebral neurocentral suture fusion collectively support a juvenile age. However, the presence of secondary remodelling and medullary expansion [108] may obscure additional LAGs, suggesting that this individual was at least in its third year of growth.

Cranial proportions further support the interpretation of BP/1/8468 as a juvenile. In embryos, hatchlings and early juveniles, the orbit is proportionally large relative to skull length (figure 64), but its size decreases progressively in late juveniles, subadults and adults [102,109]. Similarly, the base of the antorbital fenestra is anteroposteriorly short in hatchlings and juveniles, elongating in subadults and adults. Although parts of *Nyana*’s skull are missing, the preserved elements exhibit proportions comparable to those seen in juvenile, subadult and adult *M. carinatus* specimens (BP/1/4998, BP/1/5241, BP/1/4934) as well as in adult *Mussaurus* (MPM-PV 1868). These observations not only clarify *Nyana*’s ontogenetic stage but also indicate that it represents a larger-bodied juvenile.

**Figure 64.**
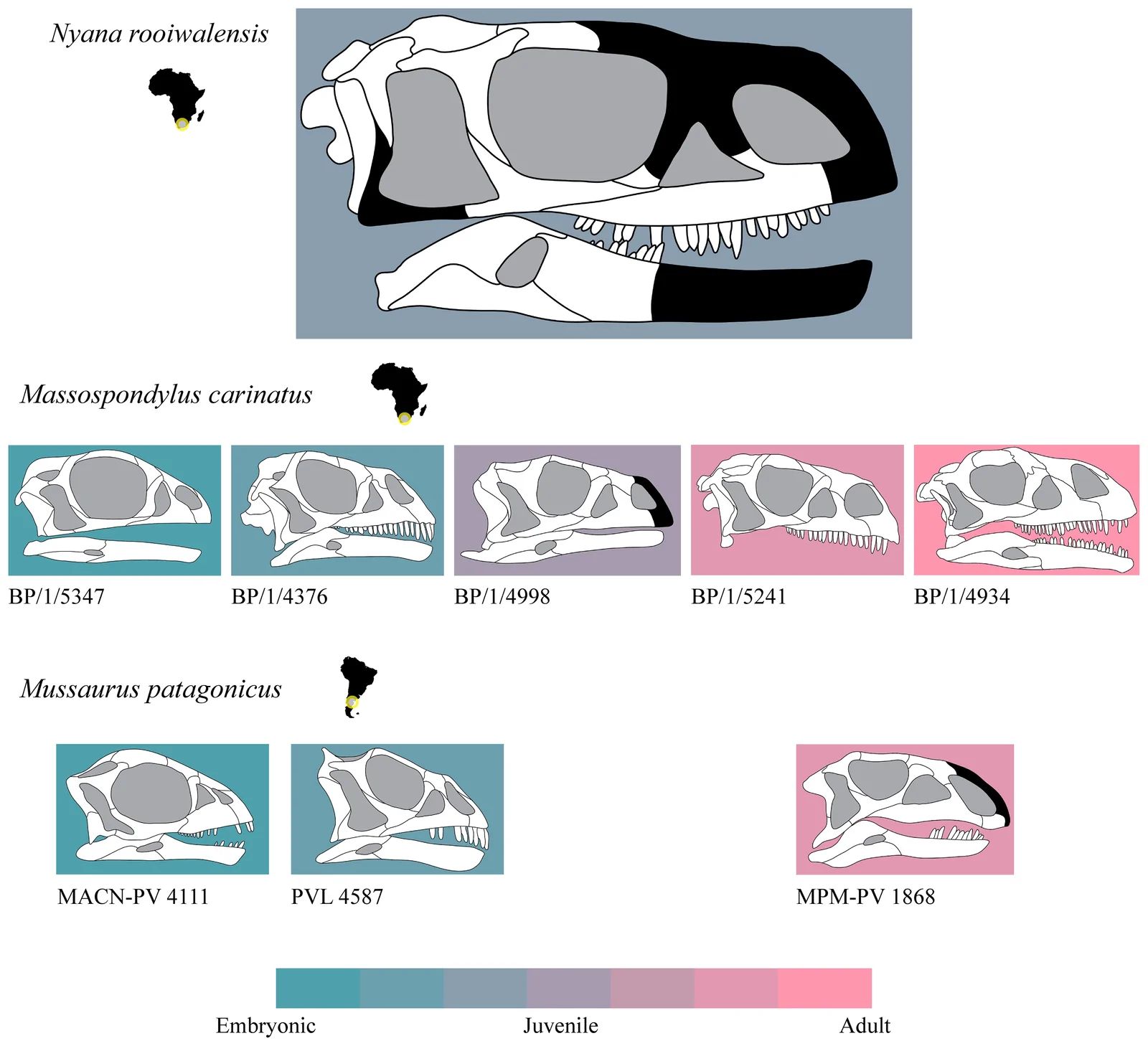
Ontogenetic stage comparison of *Nyana rooiwalensis* using the growth series of *Massospondylus carinatus* and *Mussaurus patagonicus*. Colour-coded according to ontogenetic age. Details on specimens and data used for determining ontogenetic age refer to S4 in the supplementary material.

### 8.3. Palaeobiology, biogeography and evolutionary significance of *Nyana rooiwalensis*

The holotype of *N. rooiwalensis* represents a large juvenile individual with a femoral shaft circumference (measured near its proximal end rather than the minimum mid-shaft) of 194 mm. This is slightly smaller than that of the largest known adult individual of *M. carinatus* (BP/1/4934), which has an estimated mid-shaft circumference of 214 mm and an estimated body mass of 543 kg [20], and a subadult *Mussaurus* [110] (MPM-PV-1902) with a mid-shaft circumference of 206 mm and an estimated body mass of approximately 400 kg. Other specimens of comparable ontogenetic age based on the number of LAGs include other individuals of *M. carinatus* [111], including BP/1/5238 (37 kg; 5 LAGs), BP/1/4751 (54 kg; 5 LAGs) and BP/1/4693 (91 kg; 8 LAGs). *Nyana* probably had a body mass of approximately 400 kg at this ontogenetic stage based on growth curves derived for *M. carinatus* and *Mussaurus* [112,113]. By comparison, *Nyana* was over four times the size of juvenile individuals of *M. carinatus* and *Mussaurus* based on femoral circumference or LAG counts, marking *Nyana* as one of the largest juvenile dinosaurs known from the Late Triassic. Extrapolation of these growth curves suggests that adult *Nyana* may have reached multi-tonne body masses.

Limb material for robust quantitative postural estimations is insufficient, but proportions can be estimated based on the preserved material. By comparing the ratio between log shaft circumferences from the proximal ulna and proximal femur, *Nyana* has a ratio of 0.86, which is within the range of other bipedal sauropodomorphs such as *Lufengosaurus* and *Plateosaurus* [56]. This morphological evidence is consistent with *Nyana* being a large-bodied, bipedal juvenile.

Other non-sauropodiform massopodans (e.g. *Xingxiulong*, *Riojasaurus* and *Jingshanosaurus*) and sauropodiforms (e.g. *Mussaurus*, NMQR 1551, NMQR 3314 and Lessemsauridae) are estimated to have adult body masses of at least 1 tonne (figure 65) [56]. Earlier-branching non-massopodan taxa, such as *Plateosauravus* and *Ruehleia*, are also estimated to have body masses of at least 1 tonne [56]. Massospondylids (body mass range of 170–500 kg) are bracketed by sauropodomorphs crossing the 1 tonne boundary (figure 65). If *Nyana* is indeed a massospondylid, as recovered in some of the MPTs (figure 63), it suggests multiple origins of more than 1 tonne body masses within the clade. This implies that massospondylids might have possessed a different ecological and life history strategy (slower growth, bipedality, smaller body size classes) than other sauropodomorphs that display rapid growth [114] and supports rapid growth arising early in sauropodomorph evolution. If *Nyana* is instead an early-branching massopodan with its sister taxon, *Sarahsaurus* with an estimated body mass of approximately 170 kg [56], it suggests body mass disparity among closely related early-branching massopodan taxa.

**Figure 65.**
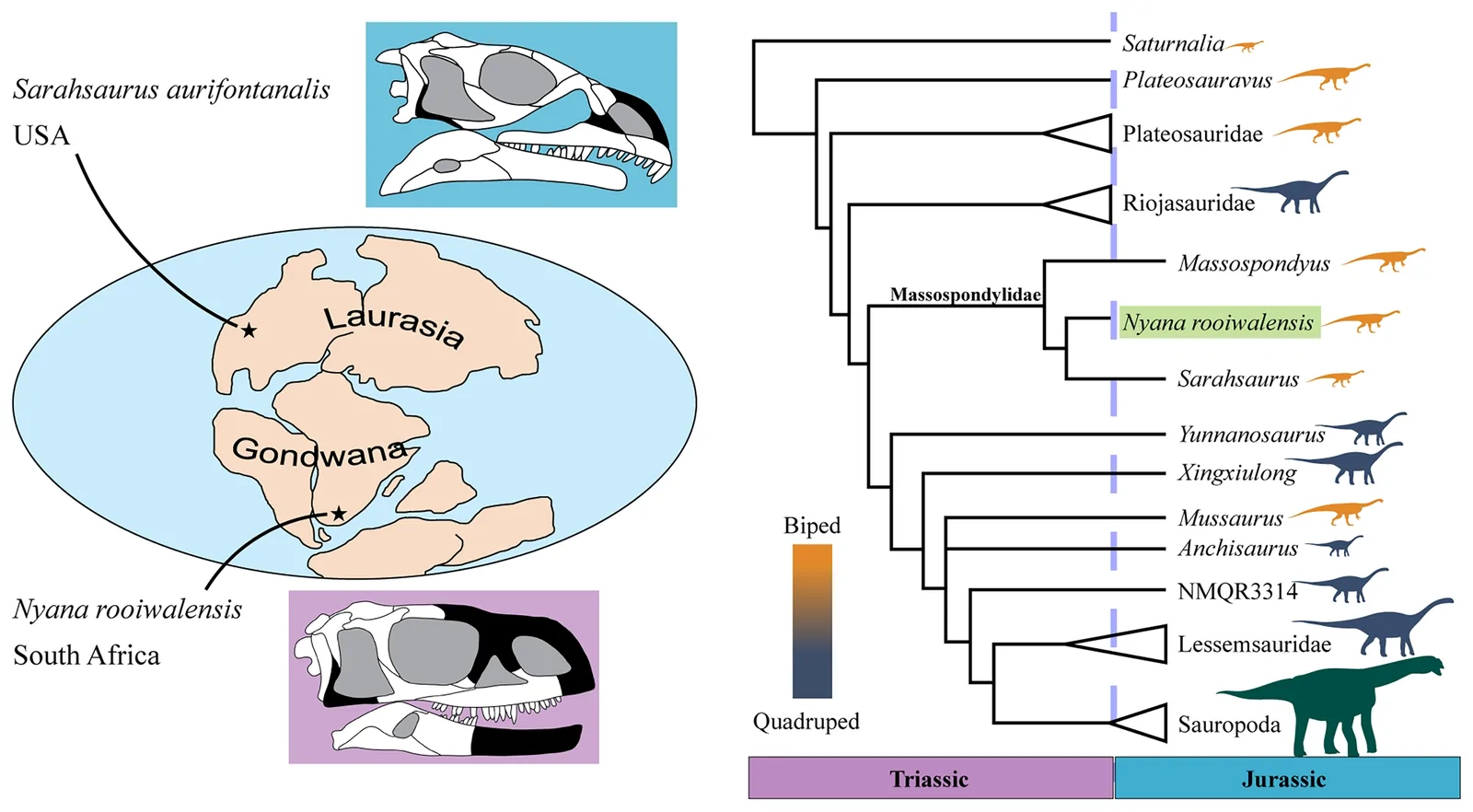
A map of Pangaea (left) at 200 million years ago depicting the location of the landmasses and the location of *Sarahsaurus* and *Nyana*. A simplified cladogram (right) with *Nyana* considered as a massospondylid, illustrating the major relationships of non-sauropodan sauropodomorphs and the distribution of body size and posture across the clades.

The placement of *N. rooiwalensis*, as the sister taxon of *Sarahsaurus aurifontanalis*, provides a biogeographic link between the early-diverging sauropodomorphs of southern Africa and western North America (figure 65). If *Nyana* is a massospondylid, it is the first Triassic member of the clade recovered from South Africa and indicates that the clade had a southern Gondwanan distribution in the Late Triassic prior to the radiation across Pangaea in the Early Jurassic. If *Nyana* is considered an early-branching massopodan, this suggests that this clade was widely distributed prior to the massospondylid radiation. Given the current low count of occurrences of this group, it is also likely that related species were present, but remain unsampled or unrecognized, in other regions. A similar potential faunal link also occurs between the Early Jurassic theropods, *Dracovenator regenti* from South Africa [115] and *Dilophosaurus* from the western USA [116]. This implies widespread dispersal of these dinosaurs across Pangaea during the Late Triassic and suggests a widespread, global dinosaur community with a similar composition at this time, even though these links are currently strongest between Early Jurassic taxa [68].

## 9. Conclusion

*Nyana rooiwalensis* is a new taxon of EBSM from the Upper Triassic lEF of South Africa, adding to the species richness and lineage diversity present in this part of Gondwana. The holotype and only specimen (BP/1/8468) includes the first almost complete and articulated dinosaur skull from the South African Late Triassic and is one of only a handful of such skulls from this time in the southern continents. Osteohistology indicates that this individual was a juvenile but was comparable in size to an adult *M. carinatus* (estimated at approx. 400 kg), suggesting that *Nyana* could have grown considerably larger as an adult. It provides critical new data on skull morphology in early-diverging massopodans that will be of use to future phylogenetic studies, suggests a novel biogeographical link between southern Africa and North America and supports the adoption of rapid growth strategies early in sauropodomorph evolution.

## Supplementary Material



## Data Availability

All data generated or analysed during this study are included in the supplementary material [117]. The 3D models and osteohistological thin sections are available from MorphoSource at https://doi.org/10.17602/M2/L899578.
