# Supplementary material for: Nyana rooiwalensis: South Africa's earliest near-complete sauropodomorph dinosaur skull from the Late Triassic of the Eastern Cape Province
Source: R Soc Open Sci. 2026 Nov 11;13(11):261002. doi: 10.1098/rsos.261002 (PMC13750947; doi:10.1098/rsos.261002)
Supplement: Supplementary file 1 [file rsos.261002.zip › rsos.261002/rsos261002_si_001.docx]

Character matrix:

BEGIN TAXA;

TITLE Taxa;

DIMENSIONS NTAX=83;

TAXLABELS

Euparkeria Crurotarsi Marasuchus Ornithischia Agnosphitys Silesaurus Neotheropoda Staurikosaurus Chindesaurus Herrerasaurus Guaibasaurus Eoraptor Saturnalia Panphagia Chromogisaurus Buriolestes Pampadromaeus Bagualosaurus Thecodontosaurus Pantydraco Efraasia Nambalia Jaklapalisaurus Pradhania Macrocollum Unaysaurus Ruehleia Plateosaurus_gracilis Plateosaurus_trossingensis Glacialisaurus Coloradisaurus Lufengosaurus Xixipiosaurus Massospondylus_carinatus Adeopapposaurus Leyesaurus Ngwevu Plateosauravus Riojasaurus Eucnemesaurus_fortis Eucnemesaurus_entaxonis Seitaad Sarahsaurus Yunnanosaurus_huangi Xingxiulong Jingshanosaurus Chuxiongosaurus Yizhousaurus Anchisaurus Mussaurus Leonerasaurus Sefapanosaurus Aardonyx Irisosaurus NMQR3314 NMQR1551 Meroktenos Ingentia Lessemsaurus Antetonitrus Ledumahadi Kholumolumo Blikanasaurus Camelotia Schleitheimia Pulanesaura Gongxianosaurus Isanosaurus Tazoudasaurus Vulcanodon Shunosaurus Spinophorosaurus Patagosaurus Barapasaurus Cetiosaurus Omeisaurus Mamenchisaurus Neosauropoda Musankwa_sanyatiensis Musango Issi Tuebingosaurus BP_1_8468

;

END;

BEGIN CHARACTERS;

TITLE Character_Matrix;

DIMENSIONS NCHAR=418;

FORMAT DATATYPE = STANDARD RESPECTCASE GAP = - MISSING = ? SYMBOLS = " 0 1 2 3";

MATRIX

Euparkeria	00000000?0000?0000000?000?100100000000000000000?0000000001001000000000000?000?000000??00?000?000000000?1000000000000000000?00000000000???0000??000000000?00?0?00??00000000000000000000000?0?0?????????000?0020000000000??????00000000001?0?0?0000???00?0010?0000?0110000000000010000000000?02?000??11000001?000?100000?0000000??0?00010??0000??0001?100000000?00?????????0?00000?0000000000000?1?000??20?00?0100000000??00????2?{0 1}0

Crurotarsi	0000000000000?0{0 1}000000000?0000000000{0 1}0000000000000000000010000000000000000000?00000??0000000000000000000000000000000000000000000000000???0000??000002000?00?0?00??00002000000000000000000000000000{0 1}00?0{0 1}0?002000000000000000000000000000000000000{0 1}000000010?0000?0{0 1}00000000000000000000000?00?0000010000001?0000000000?0000000??0?00000000000000000010000000000000?0??00???0?0?0100000000000{0 1}00?0000?????{0 1}?01{0 1}00??0????????????0??

Marasuchus	00???????0?0????????????0?00???????0???????????0???????????????????????????00???0000????????????????????????00?00000000?1?100000000010???00?0??00?000000?00???00??000?00000000?000000000??01010000000?000?0121000?0000??????????????????????????????000000000000?0100010000000000000001000000011000100000000000000001000100100000?00000000000000000?00?0??00???1????????0?0000?0?000000???00000?0000?020??????????0???????????????

Ornithischia	0010000000100?0000000?000?1011000100100?1000000100100001000000{0 1}000100100000000100001000010010{0 1}01{0 1}000000100101001110100001000000000010100{0 1}0000??010000000?00?0?00?000000000000000{1 2}10000000000010000100001001111100000100000000000000000000000000000000110112100010001??000?000110000000100001?010111102000000000{0 1}011110002?01?{0 1}100000111100000000000{0 1}000000000001?000???????000?0001{0 1}{0 1 2}000001011010200?????{0 1}?01?00000?????2??????1{0 1}{0 1}

Agnosphitys	??????????????????????????????????????????????????????????????????????????????????????????????????????????????????????????????????????????????????????????????????????????????????0?????????????????????????????????????????????????????????????????00000020000100???????????????????????????????????????????????????????????????????????????????????????????????????????????????02???????1???????????????????????????????????????

Silesaurus	00?0??00?0?00?00100000??0?00?0?01010?????????0010??0????00?????????00?0?0?0000??000110?????1?00?0?010???0?0000010001000010?00000000000???0010000000000010100001100000000000000??00000100000?0?0???????111?1110000100000?????????????????????????????000000000000000000000000?010100?0?10000110110001011000100011000000000?0000000??11?1??00??000000?00???000?00100000?0?1?00000??010000???11000?0000????11000000??0?????101?1???0{0 1}

Neotheropoda	00{0 1}0{0 1}002{0 1}000201001100001{0 1}000000000000110000000010{0 1}100{0 1}01000001{0 1}0001{0 1 2}0100000010110000101110001000000001?00000000000000000101100110000010000{0 1}1000010200001010110010000001000000000{1 2}10001100000000010100001000101100000100010001200000010?0001001101{1 2}010101013000211101010000111110000110100001?011000100000000{0 1}011011111002001011000011111000?0?00000100000000000100000?1?00?0001000111111{0 1}120111{0 1}0000?????{0 1}?01000000????????????0{0 1}{0 1}

Staurikosaurus 00???????????????????????????????????????????????????????????????????????????????????????????0000000001000?00?0000000000????????00010????00?01001?000011?000?00000000020000001??010101100000110010????1?1???????????????????????????????????????????000000200110000110?010?00010?00000100001??000?0101?000000001000000001????01????????????????????????????????????????????01????01???????1?110?010?00????????????????????????????

Chindesaurus	???????????????????????????????????????????????????????????????????????????????????????????????????????????????????????????????100?????????????01?0??0?1??0?1??????000??00???????????1??0????1?00??????????????????????????????????????????????????????????0???101?1????0??????????????00?0101110001000100??00?10111100???011110100??1?????????????????????????????????????00?????1???????10?10???00??????????????????????????????

Herrerasaurus 00000000?0001000000000000?00010000000000100000010001010100000100001101000000101?0001000?1000?00000000?1000000000000000001010001100000100000?000010000011??0010{0 1}10000002000000100000101100000110010100?1110??01100000000000001200000110000011011010010000002000000000000010???01?010100100001011100010110001000010000100010010010100000011000100001000000000000?0000000000?00001000100000111011{0 1}011000000??0??0?0000101?20?1???1011

Guaibasaurus	??????????????????????????????????????????????????????????????????????????????????????????????????????????????????????????????????????????????????0???01??011001000000??00000??000000?00000?01?0??{0 1}00??0?????110????1?0???????0????110??0?1?00??0?0?000?00{1 2}100210100010000000010?1011000000100100001011000100?0?01111100100?01101?00101110000000010100000000000100?00??0?0?0?010102???????111?1111??00?0??????????????????????????

Eoraptor	0001?001?0001000011000111010000010000110100000?101100?0100?01100001101????1?1????????????????10000010???0?0100?10{0 1}0000001?????110000000000?1011??000??00?00???00?000??00000011??10???100??001?00??000?0000??1?10??00101??????20?00011?00010?00000101101000{1 2}0002101??0??00?0???10?1011?10??0?0111000101100000?1?00{0 1}1010??1?0??0?????01????000???00?0000000000??01????????????00100021011110111100110???10?0?????0?00?0?0?1?1???101?

Saturnalia	10????????????????????????1???????00?100?0??????????????????10?????????0???0?0??0101?0??????????0?0?0??????0000100000000????????0010010010??01101?00000100001100000000000000000001000101??0??1000??0??10101111100010100?????????????????????????????00?0001100210000000000100110010010000001011100010110001000000010100010010010100000001000000001010000?000?001000000000000000??12??000??11111?1100??101?00?0000?0?????????????01

Panphagia	?0???????????????????0?1???????????????????0??1????????1?1?0???????10?0?1?1????0?????????????1?0?0?1010000?10?0101000000????????00000100011?01101?000?01???01?10000?0?0000000??0???00??????????00??0??000???????????????????????????????????????????0??0??11002100?00??000????1??0??10?????????????????????001000{0 1}10100???010?1010?????????????0?????????????????????????????????02???????111?1?0?????????????00??0??1?211????????

Chromogisaurus ????????????????????????????????????????????????????????????????????????????????????????????????????????????????????????????????????????????????????????????????????????????????01000??1100??1???????????????????????0??????????????????????????????0????0{1 2}1002100??????????????????????0??????1???10110??1000000010100010????????????????????????0??0???000???1?????????????????12???????11?11?????0?????????????????????????????

Buriolestes	0?01000210101010011000111000000000000100100000?10110010100001?00001?01100110000???0?0??1??00100000010100000100010000000010100010010001001011?1101000000100011?00000000000000000?0100010?0?000?000??0???1????111010011?0???????????01????????????????00000?10002100?0??0?001?011?0001101000000111000101101000?1?000?01?0?1??1?0?????0100???000??00?0100000000??010?10??10???????????1????????????????0010?{0 1}????????0?01?{1 2}??1???????

Pampadromaeus 0001000210101?10?11000?11000??00100001100000????0?10010??0??1?????110?1??????????????????000???000000??00001100101000000??10001????????????1??????0??001??0?1?10??0000000000{0 1}0?0??0001?10?0??1000??0??1110??1?101010000?????????????????????????????0000001110?10??????????0?1?????????00001011100010110?0?00?00???????01?????????????????000???0??1?0???0??????0??????0??????????10?100??????????????00???????0??????????????????

Bagualosaurus 10?1?00{1 2}??0?{1 2}0???1??????001?000?1000?10??0???0?10010?????????????0??????????????????????????010000010?????01?00101000?00??????????????????????????0??001??0?1???10?0?0000000?0??01?001???????01???0??????????????????????????????????????????????????0?00?1100?10{0 2}?00?00?0?????????????000??0111000101110?10000000?01??010????????????????0?1??00?0100?????????1??0??110??????????10??0????1???????????0???????????????????????0??

Thecodontosaurus ?0????????????????????????1??1??1000??????0?0?0?0???000?????????????0???0?1010?10011?0???????00?10010??????1100101010000?1??????000101010?1?01101?000001??00110000000000000000?1000?0101100?01000??00?010011211000101001100012010001100001010000010100?00121000001???0?000??011??01010?000011110000101100010000000101100??011010100????110001110?1?000??00?0????????????????1??00010000??01?110???000?20?000?10???0???????????????

Pantydraco	?0???0????????????{0 1}00?0???1??1????000100100000010?100001?00????????00?000?1?10??00110011?000?00?00010??0000?1001010100001001??1100?10101001101101?00???1????1???????????????????????0??11?00010000100?????11?1?00???????????????????????????????????00100121100001?????????0011??10110?00??????????1?1?000?0000000?01?001?????????????????001110011?0000000010010??00000????0????0100??1??1????0??0??0??1100??00??0?0??2101?1?00??

Efraasia	100?1001?000??1?111?112?1110?100???00100100000??0?10??01?0??1??????10?000?1????10011100??????10010010??11?011001010100001???1?11001101001011011010000001??001?00000000000000000110000101000?0110000?0?010011212000111001101012010?11000001110100010000100131000101000010000001100101101000011110000101100010000001101?001?011010?0?01?1110001??0011?001000001000??0???000?10001000100001??1111??1000002011001?00??0????2?11?0?????

Nambalia	??????????????????????????????????????????????????????????????????????????????????????????????????????????????????????????????????????????????????????????????????????????????????????????????000????????????????????????????????????????0?10??0110??????02?0??10??0??????????1????111?0100110101001011010???????1101?0???011010??00101???00101?011100??00????0000000010????????????????11?10?0?00????????????????????????????????

Jaklapalisaurus ??????????????????????????????????????????????????????????????????????????????????????????????????????????????????????????????????????????????????0???0??????????????????????????????????????1??????????????????????????????????????????????????????????????????????????????????????????????????????????00?000010110110???011010??0???????00??1?0??1????????????00??101???????????????????????1???????????????????????????????????

Pradhania	????????????????????????0?1???????00??????????????????????????????????????00??????????????????0???1?1??????????1?1010?????????????20??????????10??????????????????????????????????????????????????????????????????????????????????01?????20?0?????????????????????????????????????????????????????????????????????????????????????????????????????????????????????????????????????????????????????????????????????????????????????

Macrocollum	1001100210002111111111211110010010000100100000110010010100001000001101??????101???????0?100??1001011010?1?0110010101000011?0111100200100111101101000000111011101000000000000100110?0010?0000001000100?01001111200001100110111201010101000211000001000010012100010200?0?0001?01100101110100000110000101100010000{0 1}11101100100110101000100110001010011100000000100000000011001?00000??????????1??????110010???????0?00?1?????????????

Unaysaurus	?001100?1010??1?111??1??1010??011000????????????0??00?????01???000??0?0?????????????10????????0?10110?????01100101010000???0?1?????0?1????????????0??0011100110000000000000???????????????????1000?00?010001?1101001100??????10?0?110????1110??0?????????????????????????????????????????????????????????????????110110???01?010?00?????????11???????0???0??????0????????????????????????????????????01?110??????????1????1???????

Ruehleia	???????????????????????????????????????????????????????????????????????????????????????????????????????????????????????????????10???0????01???101?002001??00110010000011000000?1100001?00?0?0110??????01001111200011100?0100??01011101000???0??00???001001311000020??11000110110?10110?101011010000102100010?00?011011001?000010100??????????????????????????????????????0?00010??????????????????0???1???????????0???????????????

Plateosaurus_gracilis ?00??001?0002?110111?1??1110?101??00?10?1010?0??0?10001100??10??????????????????1?????????????0??0110?????11100101010?001?????1100??01???01101101?00?001?100110000000000000010?1010001010000001000?00????????1200?11100?1011?10101010000???10?????0?001001311001020010100011111001011??00001111000010110?010000?0??0110????????????????????0?????????0?0????????????????001000?000?00001101111?1?01?00????????????0????????????0??

Plateosaurus_trossingensis 1001100110002011{0 1}11111211110010110000100101010110011001100011001001101100101101110011000101111001011010111111001010100001100111100110100{0 1}011011010002001110011000000000000001011010001{0 1}000000010000000010011112000111{0 1}011011110101110010020100000100001001311001020010100011111001011110000111100001011000100001011111001001101010001011100011000111001000001000000000111010000000{1 2}000001011110?1010001002000100000001?01111102001

Glacialisaurus ????????????????????????????????????????????????????????????????????????????????????????????????????????????????????????????????????????????????????????????????????????????????????????????????????????????????????????????????????????????????????????????????????????????????????????0????????????????0????????????????0???1010{0 1}????11010111?01??????????????101110111?0????????????????????????0???0??????????????????????????

Coloradisaurus ?00?1002??002?111111?1??1110??111000?10??01?10?101110111?000100100?1011011011010100111?????1?1001011010011??000101010001?11?001100200100101?01100?00?001?1001100000000000000101????00?10100?001???????00001011210011?1???????????????1???????????????0?0?131100???011?11002001100101110000011010000102111010000001101100??011110?00??????000111001200011000021001111101100111????0?0000??????????0?00?101100?100000?1002100001??00

Lufengosaurus 100???02???0211?1?11?1111?111110100101011010000101110101100010?1????0110??01{0 1}0??1001?1001001010010????0111?11001010100011?00011100100110111?0110100020011100110000000000000010011010011000000{0 1}10000000001011112{0 1}1011110110111101113100101201000102000010013110000201101100210110010110000101101000010211101010000110110010011110?0001?1110101110012000110000{1 2}{0 1}0010111011???00?0000100000??1????1????0010??0????????111??1?????0???

Xixipiosaurus 100?10?????0??111?11?1111?1111?0??000101?11000?10?11?1111?0?1001????01?0??01?01??????????????1?01?010???1??11001???1????????????????????????????????????????????????????????????????????????????????????????????????????????????????????????????????????????????????????????????????????????????????????????????????????????????????????????????????????????????????????????????????????????????????????????????????00????????????

Massospondylus_carinatus 1001100210002111111111211011111010000101101000110{0 1}11011110001000102?01101100{0 1}0110001{0 1}1?01000010010110101010110{0 1}1010100011100?111002001001111011010002001110011000000000000001011101001{0 1}00000000000000?01{0 1}011112010111{0 1}0110111101112100101211000002000010013100010001101100100110010111000101101000010210101000000110110010011110100011111000111001200010000021000?00?011001000001?100000????????1???0010100111000000000{0 1}1000010000

Adeopapposaurus 1001100210002111011111011011111010000101101000110111111111001000102101{0 1}01100101100011100101000001011{0 1}10101011111110{0 1}00011100111100200100011101101100200111001100000000000000100110100100000000010000000000101110001110011011110111210110011100000200001001311001000110110010?1100101100000011010000102101010000001101100100111101000111010001110012000110000110000000011010010000??00000??1?11?11???00101001?100000?010?11100?0000

Leyesaurus	?001?00?1000211??11?????1010?11010000101101?00?1011111111?00100010{0 2}1010?????????00?11???0??1?00?10110??1010?111101000001?1??1111002001000111011????0???????????????????????????????????0000?001???000??????????????????????????????????????????????????????????????????100?????????????????????????????????????????????????????????????010???????12000????0???0?????00?????????????000??????????????0??0?00??1?0?00?0??11?1?????00

Ngwevu	1?01010??10021111111????111?10101000?10110?000?10?110111111?0001100101001111001??00??111?001011?11010?????011011010100011????111?020?11001110?1??1?00?0???0??????0???0000?0010000???????????????????????0???1121?101??0???????????11?????2010?????????????????????0?1?1100?????????????1010?101000010??1??1???????????0??????0?0?????????0001??0??2?00??000???0??0?????1????????0??????????????????????????????????011011010100???

Plateosauravus ???????????????????????????????????????????????????????????????????????????????????????????????????????????????????????????????10???0100101?01101?00{1 2}0011?00110000000001000000??10??011???0??110????????0???{2 3}1110011110????????????1????????????????00100131100101?01????????11??10110?00001111000010110001000000110110??????0???????????????11?0????0??????????????????????000???????????????????0???0???????????0???????????????

Riojasaurus	1001?00??0002011?110??00111011011000?100001000?10010?10110001000001101?0?100101?0001???????1?001000?1??101?100010101000{0 1}10??11110011010010110110100010011100110000000000000010?1101001{0 1}00000011000??0?01001121210111100111111?010111011001010001020?0010013110010201101000110110010110010110111010110201101000000110110010011010100??011?00011100121011{0 1}00001??1????????0?10100010100000?011110?00110020??0?????000?????????????00

Eucnemesaurus_fortis ??????????????????????????????????????????????????????????????????????????????????????????????????????????????????????????????????????????????????0???01??001100{0 1}10000??00?0??????????10000?01100?????????11???????????????????????????????????????????????????????110???0??????????????0110111010?1010110?000001110110???????????????????????????????????????????????????????????????????????????1?0?????????????????????????????

Eucnemesaurus_entaxonis ??????????????????????????????????????????????????????????????????????????????????????????????????????????????????????????????????????????????????0???01??0????0???000??0000?00?1011?0100?0?01??????????????????????????????????????????????????????0????13??{0 1}0100??1??0?0?0?11?010110?1011011101001010110?0?????110111?1?0??0?????0??????00111?01{1 2}?0?11000?????0???0?????????????????????????????1???????????????????????????????

Seitaad	??????????????????????????????????????????????????????????????????????????????????????????????????????????????????????????????????????????????????00?{0 1}?1??00??00?001????0000?????????????????????????0000011?11000111{0 1}011?11100101310?00?2110??0???0??????????????0?1000001????????????????????????????????0?00?01101?0???01101010?0??11?00?1??001???11{0 1}0000010??0???0?10??0??001??????????????????????0??????????????????????????

Sarahsaurus	10?1000?10?11010?11?1?1?0110110100000100001010010?110001100010?0???101000111001???01?1001??1?10?10010?????011001010100001?1011110110011101110111110010010100101110000010000010111000011000010010000001000010102100111111101111011021111?011100001200100001311101021001110011111011011102?1001100010101110010000010{0 1}01000100011101001?1111011?110011000?100011100??0???11010??000????????????????????0010??????????0010?0??1?1010??

Yunnanosaurus_huangi 100?1002??00??10?110?1??111?111????00101111?00??0?1101111?001000?01?01?0?00010???????????????10010010???1???00010?21000?????111100110110100?01101000?0011?00110000000000000010?0101101{0 1}0000?00?0??0??000?0??11200?11110??01??0011?2101100201000{0 1}020?0010013100000001101{0 1}00{1 2}0?110010111?0010110100001011010101000011011001?01101010001111?010111001????1?0000110??0???11??0??1??010100000?01?1???00??0?10??????????0?000????????1??

Xingxiulong	??????????????????????1???1?111????0?10?00???0110?11001100001000101?01?00100101?2??1?1???????0?????????0{0 1}1???00?????????1100111100100110001?011010002001110011000000000100001100110??11?1000001????00?0000???120?011110????1??????1101??????????????001001311000020?1011002??11??0001000000111100001011010?00000{0 1}101110010011010?0?1011000101100012101110000210000000?11????????????????????????????0??0??????????0?00????????????

Jingshanosaurus 1001?002??00211111100???11101{0 1}101100?11?001??1?10?11011110001000????01100000101?2?1??10?0??1?10110010??11101100101011001????111??010??????1???10100??0011100?1???00000000000?0??10??01100000001000000?0010??11201011110??00??0011?3100100?010001020000100131000002011001002??110010110?00001101000010111101010000110110?12011010?00??????01011110120011{1 2}0000010?????????????????1???????????????????0010??????????????????1????1??

Chuxiongosaurus ?00??002??00??111111?1{0 1}?11101{0 1}10??00?11100???1??0?1101111?0?1000????01???????????????????????101?00?0??111?1100?0101?????????????????????????????????????????????????????????????????????????????????????????????????????????????????????????????????????????????????????????????????????????????????????????????????????????????????????????????????????????????????????????????????????????????????????????????????0????????????

Yizhousaurus	110110021010211111101111111111011000011100100011001111111100??00???10?100?00101?2?????000001010110010??0000110{0 1}1010110011?10111100100110101?011010{0 1}000011100{1 2}10000000001000010?01000011?000?01???????001001111100011110??????0010?21011011010001020000100?31100000010011002?0110010110?1110111100001011110????????????????????????????????????????????????????????1???1?????????????????????????????0?2???????????0???????????????

Anchisaurus	10???00??0002?1??11?????111011?010?001010?100011001111011?0?10??????01?001101?1?2001110??????10?10?00??10000000101?1{1 2}?0111?0011???1001001111011?10000?010?001100?000??0000000??0100?0??0?01?00?10?????10{0 1}0??211001011?0??0??1101011100100201000002000{0 1}10113100000?0111100010011?001010000101101000010{0 1}11101000000110111?1?01?010???10111?0?01??0011?001000001001??0????10??0?0?01020000??01?11?110100?20?10????000??01????1???????

Mussaurus	10011002?000??111111?11?11101110100001010{0 1}?000?10011?1011000{0 1}0?0?01?0?1??????????????????????10101111???1?0??0?1?1111001????????00100????0?10110??0000?10000100000000?00000010??111?011?1?0001110?????0010?011100111110?1?11?00101210110120100000200???0113100010?011??1001?011??10110?10101101000010111101000000110111011011010001??????0101110012001110000110000?00111111?011010{1 2}???????111??11?????10??????????0?01?????????1{0 1}{0 1}

Leonerasaurus ?0????????????????????????????????????????????????????????????????????????????????????????????????0?0???????1?11010010011???001??010?1???????1100?00?0?10000110000000??00000100?111001????????????????0000???1100000?????????????????????????????????1101?3100?????????0?????11??1?1??????????????????????????????????????????????????????10111??????????0??????0??0???1?0????????1???????1???????????????????????{0 1}????????????100

Sefapanosaurus ????????????????????????????????????????????????????????????????????????????????????????????????????????????????????????????????00??01100???0?10??00?001????1?00000001??0000????1?????11?00??011????0?0?0010?1?00????10?1011??010131010??1010???????????????????????????????????????????0?111110000??11{0 1}???000000??????0?00110101010001??0???11?0?2?0???????????00?0????011?01111?????????????????????????????????????????????????

Aardonyx	1101?00210002?11?11?????1110?1011000??????1010??0?11010??10?1????????1?0???????????????????1??0?0?1?0?????11?00101011001?1??????0010?110100101101?0000011?00110000000020000010?1100?0110000?01110??00?????11?????????10??00??????13101???1?10??10?????????????????1110100010011??10110?101?111100001111000???????1101110?0???01??????????010111?01?0?2??00?0???0???00011???0?111????????????????????0??0??????????0???????????????

Irisosaurus	?????????011??1???1?????10????1??????????????????????????????????????????????????????????????????????????????0?10?21100?????????00???????????110??00?001???????00?0???0?0?00???????????????????????????1?010??1??01?1?0??????1010121011012010000020???????????????????????????1??10110???????????????????????????????????????????????????????????????????0?0??????????????????????????????????????????0???????????????????????????

NMQR3314	1001?1031000211111101?1110101111???1010101101011011101011?00000010220110110010????0??10011011001?00101?1000110?10021100110?01011001?01101001011{0 1}10000001??0??100??0?0?10000000??111001??010?0?1??0?????1????{2 3}1100?0?110??????0010?2100101{1 2}0?0001020000100?31000{0 1}0{1 2}????????????1??10110?????????????1???11010000011?011?01?0??0?0???0011100101111012?011{1 2}0000110001100?11????????????????????????????0002?{0 1}0???????000?????1???????

NMQR1551	?????????????????????????????????????????????????????????????????????????????????????????????????????????????????????????????????????????????11???00?001??00210000000010000000??2?10012?000?0{0 1}1100????0100??2????000110???????????21????????????????00100?3100000{0 1}011010001????????????11101111001110{0 1}111010000011101100100?10101000??????1011100????1120000????001?0?11????????????????????????????00?1??????????????????????????

Meroktenos	????????????????????????????????????????????????????????????????????????????????????????????????????????????????????????????????????????????????????????????????????????????????????????????????????????????????????????????????????????????????????0??0?131?00001011?00001????????????111111100?001111110???????????????????????????????????01?????????????????1??0???????0??????????????????????????????????????????????????????

Ingentia	???????????????????????????????????????????????????????????????????????????????????????????????????????????????????????????????????111????01011?0??0?????????????????????????????????????????????????????????11{0 1}0010110?1?10?00111310110???????????0?????????????????????????????????????????????????????????????????????????????????????????????????????????????????????????111????????????????????????????????????????????????01

Lessemsaurus	????????????????????????????????????????????????????????????????????????????????????????????????????????????????????????????????0?011110100??1101?0000011100{1 2}10000010121000000????????????????????????0001???11{0 1}0010110???????0?1?310?????0?0??1????0010013100000?011110001???1??10110?111011?10???11111?0?000000100101???011011101??????????11??12101??00??0???11?????10?201??1001?????1?1111??1010????????????????????????????01

Antetonitrus	?1?????????????????????????????????????????????????????????????????????????????????????????????????????????????1?1012000????????0?{0 1}0???????????00?000001??00{1 2}10001010121001000???1????100?0?01100???0?0011??311000{0 1}0110??????0011?3101???1010???????00100?3???0002011010001????????????11101111001111111101000000100111010????????????????1111110????{1 2}1200?0????0?100??1?0?00111????????????110??0????2???????????????????????????

Ledumahadi	????????????????????????????????????????????????????????????????????????????????????????????????????????????????????????????????????????????????1?0??0011???{1 2}?0000010121001000????1???1??00?01???????????????????????10???????????{2 3}1????????????????????????????????????????????????????0???????????????????????????????????????????????????????????????0?????????0???1?????????????????????????????0?????????????0???????????????

Kholumolumo	????????????????????????????????????????????????????011?????????????????????????????????????????????????????????????????????????0101?1?1????0??01?00?0011???2??0000101??????????????0110??0?111100????010????11000{0 1}??00???????????2101???1?11???????10000131110000010?10001????????????00011110000011110?1?0010010001000?1?????????????????0111??1200???????????1?000011?1?001010???????????????????????????????????????????????01

Blikanasaurus ???????????????????????????????????????????????????????????????????????????????????????????????????????????????????????????????????????????????????????????????????????????????????????????????????????????????????????????????????????????????????????????????????????????????????????????????????????????0000?0110111?1?0100101001011100101111112102120000100?01?0?1?11?0?0??????????????????????0???0??????????????????????????

Camelotia	???????????????????????????????????????????????????????????????????????????????????????????????????????????????????????????????????1???????????0??0??001??002100000000??00?0??????????1???0??011???????????????????????????????????????????????????????????????????1??????????1??00111?111011110111?1??110?000000????????????????????????????????????{1 2}???000??????????????????????????????????????1???????????????????????????????

Schleitheimia ????????????????????????????????????????????????????????????????????????????????????????????????????????????????????????????????0??????????????01?10?0111?0???????000??1??????????????????1??110?????????????????0?0??????????????????????????????????????3111{0 2}?02???????????????????????????????????01110??????????????????????????????????????????????0?????????1???????????????????????????????????????????????????????????????

Pulanesaura	???????????????????????????????????????????????????????????????????????????????????????????????????????????????10?012011????????001?11?0100??11?????0??1??00?0010?01?0001000????????112?11011???0??????????????10??1?????????????????????????????????????????????????????0?11??1?11???????????????????????00000100101?????????????????????????????????1???????????0?????????????????????????????????1?????????????1???????????????

Gongxianosaurus 1?????0??????????12???????????????????????????????????????????????????????????????????????????????????????00???1??2121????????????0????????????1??0??001??0??????0?0??00000????????????0??0?0?1?0?000?011???2110????1?1?????????????????????????????0?100????0?????????????????????????{1 2}111?2??????1???1101??????1?0??1???0?1?1????1?11??010???10?2?111200002000??????????????????????????????????????22??????????????????????????

Isanosaurus	????????????????????????????????????????????????????????????????????????????????????????????????????????????????????????????????0??0???????????2??10??0???????????????2?011000???????????????????????101?0?????????????????????????????????????????????????????????????????????????????211112??0???101?1?0???????????????????????????????????????????????????????110??1??????????????????????0????1??????????????????????????????1

Tazoudasaurus 11??????????????????????????????????????????????????010??1?????????{0 2}011???1???1??????????????00?00000???0??10?1111012101???000??001010??????0002??100001??00200100011020121000????????211?1?111????11?????01?1100100?2????????010?210000100?0001?310?1???03101??0?111001002??11??1??1??21100111001101011?0?0010000101010?1111011111?011??????????????1??10?1????1??0????1?0??1001?????????????????101?????????????2???????????????

Vulcanodon	????????????????????????????????????????????????????????????????????????????????????????????????????????????????????????????????????????????????????????????{1 2}????????????????????1??????0111?0?00?0?0??01???2110?10?021???????????????????????????????????310{1 2}?00?01111000100111110010?111011110010011?1??20?10001?0???11?111011?111011??11?01010120111110?121?0?1??00?11?00000?????????????1???11?01??1??????????????????????????

Shunosaurus	11000113?110201101200000001011?20100111201010101001101012000011011121010001011?1200?????110200100000120100011111112121112??01011?1001?1?10010012?1100001011021?00001?0201210000?11?00?100111011001?111?1?000211001100?11000?1011001?000110000??1?3101111013101100011111?01100?111100100211112??0???000?01121010?01?0????110?101???11011??1111101002?12111?102110???????????0?00020?00000??1?10???0??1121???0??11?12?0???1?101??1??

Spinophorosaurus 1????1?????????????????????????????????2????????????0????01?????????0??101111111{1 2}?0???????????1?????????????100111012?112?????11?11011?0?01?0002?110??01????{1 2}?11???1?12?1010?0????????{1 2}1??11011?00?1??1110?03?{1 2}0?10??{1 2}???????????????????????????????????????{1 2}????1?1???01{1 2}??011??001??211111110?1101??0?020?100???0??11?10??0?0?01??????????????????????????????????????????????????????????????????1???21?0?1?010??1????1???????

Patagosaurus	11????????????????????????????????????????????????????????????????????????????????????????????1?00001??????0??0111012111??????1100101100000110020?2001010?00201?01111120121?00?0210011{1 2}00?1?0?100???0?111?00?1100100?{1 2}1?????????????????????????????1111013101000011111001100111110010?211112??0???0011111?101000??0???????????????????????????????????????????????????????0?????02???????1?11???110??????????????2?????????????11

Barapasaurus	???????????????????????????????????????????????????????????????????????????????????????????????????????????????1?111211???????????1?1????00??002??100101??00201101111120121100?0{1 2}1001?10?11??1????????101?00?1???????11?????????????????????????????11110131011000111110011???11110010???????????????????1?1?1?????????1?1???????1?????????0???????????2?1??????????????0000?000?01???????0?11?1?1?01?????????????????????????????

Cetiosaurus	1????????????????????????????????????????????????????????11?????????????????????????????????????????????????????????????2??0011110111110100100020?2001?10?1020??1011102010100???{1 2}?????2001??00100???011110103110010002????????1?????????????????????1111013?0210?0110?1?011??1101?00???211112??0???010?111210100010010111????????????????????????????????????????????????1?000??????????????????????1?2???????????0???????????????

Omeisaurus	110001131111201101200000101011?201001112010101011111010121100000112221110010111???0??0???1???11101001?0?00011101111121113??001110120011010111002?12101010110201100011020121100?021??1?20011100100?011111100031100100021??????0100001000110001001?31011110131011000111110011000111100100211012??0???010?01021010011?0??111?011010?11??1????1111011020121111102?10?????????0?0?0?0???00000??1?1????1??1?2???????????0?0???1?????????

Mamenchisaurus 11000113?11120110120000000101112??001112010101011111110121100000111221010000111?201??0?????2?11{0 1}0000100100011111111121113?0001110121010000110002?1110101010020110001102012100000110111211111011001011111100031100100120110011011000100011?0?10?1?31?11110131011?00111110011000110100100211112??0???010?01121010?1??0??????101011111??????1101??11?2?121111102??0?1?????????0?00?????????????????????11211?1?0??1010?0?1?1?0???????

Neosauropoda	1100011311112011012000001010111{0 2}11101112010101011{0 1}11110121100{0 1}{0 1}011222111001011112{0 1}{0 1}0{0 1}000010201110{0 1}001201000{0 1}111111{0 2}12{0 1}112{0 1}00001110011110100110020121010101{0 1}0201110011020121100002100112001111{0 1}10000100111000311001{0 1}00211100010100000000110001??1?30011110131021000111110011001110{0 1}10{0 1}00211012??0???010?11121010?11000011111010111111011??11110011020121111102110?11???110?20?00020200000?01?100??110????{0 1}{0 1 2}1000{0 1}111????1{1 2}1?{0 1}???{1 2}?11

Musankwa_sanyatiensis ???????????????????????????????????????????????????????????????????????????????????????????????????????????????????????????????????????????????????????????????????????????????????????????????????????????????????????????????????????????????????????????????????????????????????????100101110?00?0??00?10000001101?0???11?11???0??????????????????????????????10???1?0???1????????????????1????1???????????????????????????????

Musango	??????????????????????????????????????????????????????????????????????????????????????????????????????????????????????????????????????????????????????01????1?1?00?000???0?????????0?1?1000?00100??????10?00???110?1????????????????????????????????0??0?02??02102?00?10?0????1??0??0??????????????????????001000??????1110111101010010???????????1??????????????1??????01100????1????????111????0?00???????????????????????????01

Issi	?001100210001011101011211110110110000100110000110010011100011000?01?010?????10?????????????????????????????????????????????????????????????????????????????????????????????????????????????????????????????????????????????????????????????????????????????????????????????????????????????????????????????????????????????????????????????????????????????????????????????????????00000???????????????????????0?0??00??1??00120??

Tuebingosaurus ???????????????????????????????????????????????????????????????????????????????????????????????????????????????????????????????????????????????????????????????????????????????101101110101?01?0??0?????????????????????????????????????????????????0010002111010200100000100110010110?00101111000000111?01000000110101?1101001110000?1????011???0?1?0???00??0???10??01?0?000?????2???????11100110001???????????????????????????01

BP_1_8468	?0????????????????????????1?????010???0??????0?10?100001110001?????11?0011100011110101101101?1??1????10?????1001?1010000????????00000?????1?00101?00?0?10?0?1??00?0??0????????????????????????1??????????????????????10???????????????????????????????????????????????????????????????????01?????????????0???????01?110??????????????????????????????0????????????0???1?????1?????????????????1?????????010???0?001?11??1010000???

;

END;

ctype ord : 8 13 19 23 40 57 69 92 102 108 117 121 134 144 147 149 150 157 167 170 171 177 183 205 207 214 222 227 242 251 254 277 299 336 342 349 369 391 403 408;

Age Ranges:

FAD	LAD

Euparkeria	247.2	242

Crurotarsi	247.2	145.5

Marasuchus	236.1	233.7

Ornithischia	231.4	145.5

Silesaurus	236	227

Neotheropoda	227	145.5

Staurikosaurus	235	221.5

Chindesaurus	227	208.5

Herrerasaurus	231.4	225.9

Guaibasaurus	225.4	220

Eoraptor	231.4	225.9

Saturnalia	233.2	225.4

Panphagia	231.4	225.9

Chromogisaurus	231.4	225.9

Buriolestes	233.2	225.4

Pampadromaeus	233.2	225.4

Bagualosaurus	233.2	225.4

Thecodontosaurus	208.5	201.3

Pantydraco	208.5	201.3

Efraasia	215.56	212

Nambalia	227	208.5

Jaklapalisaurus	227	208.5

Pradhania	199.3	190.8

Macrocollum	225.4	220

Unaysaurus	225.4	220

Ruehleia	227	208.5

Plateosaurus_gracilis	227	208.5

Plateosaurus_trossingensis	227	201.3

Glacialisaurus	199	182

Coloradisaurus	220	213

Lufengosaurus	201.3	190.8

Xixipiosaurus	201.3	190.8

Massospondylus_carinatus	202.3	187.5

Adeopapposaurus	201.3	190.8

Leyesaurus	201.3	190.8

Ngwevu	191	187.5

Plateosauravus	219.6	202.3

Riojasaurus	220	213

Eucnemesaurus_fortis	219.6	202.3

Eucnemesaurus_entaxonis	219.6	202.3

Seitaad	190.8	182.7

Sarahsaurus	199.3	183.7

Yunnanosaurus_huangi	201.3	190.8

Xingxiulong	201.3	190.8

Jingshanosaurus	201.3	190.8

Chuxiongosaurus	201.3	190.8

Yizhousaurus	201.3	190.8

Anchisaurus	201.3	190.8

Mussaurus	194	192.6

Leonerasaurus	190	183.8

Sefapanosaurus	219.6	202.3

Aardonyx	202.3	187.5

Irisosaurus	201.5	199

NMQR3314	202.3	187.5

NMQR1551	219.6	202.3

Meroktenos	219.6	202.3

Ingentia	213	201.3

Lessemsaurus	220	213

Antetonitrus	202.3	187.5

Ledumahadi	202.3	187.5

Kholumolumo	219.6	202.3

Camelotia	208.5	201.3

Pulanesaura	202.3	187.5

Gongxianosaurus	182.7	174.1

Isanosaurus	208.5	174.1

Tazoudasaurus	190.8	174.1

Vulcanodon	199.3	182.7

Shunosaurus	161	157

Spinophorosaurus	174.1	163.5

Patagosaurus	178.7	178.1

Barapasaurus	190.8	170.3

Cetiosaurus	170.3	163.5

Omeisaurus	168.3	157

Mamenchisaurus	163	152

Neosauropoda	174	145

Musankwa_sanyatiensis	227	208

Musango	227	208

Issi	227	208

BP_1_8468	209	201
