# Supplementary material for: Nyana rooiwalensis: South Africa's earliest near-complete sauropodomorph dinosaur skull from the Late Triassic of the Eastern Cape Province
Source: R Soc Open Sci. 2026 Nov 11;13(11):261002. doi: 10.1098/rsos.261002 (PMC13750947; doi:10.1098/rsos.261002)
Supplement: Supplementary file 1 [file rsos.261002.zip › rsos.261002/rsos261002_si_002.docx]

Table S2: Morphological measurements of BP/1/8468

| **Element** | **Measurement** | **mm** |
| --- | --- | --- |
| Skull | Maximum anteroposterior length | 213.02 |
|  | Dorsoventral height at orbit excluding mandible | 58.48 |
|  | Greatest mediolateral width | 94.07 |
|  | Anteroposterior length of reconstructed mandible | 111.77 |
|  |  |  |
| Supratemporal fenestra | Anteroposterior length | 29.93 |
|  | Mediolateral width | 28.61 |
|  |  |  |
| Parabasisphenoid | Cultriform process base, dorsoventral height | 13.88 |
|  | Cultriform process, base mediolateral width | 13.32 |
|  | Cultriform process, anteroposterior length | 52.46 |
|  |  |  |
| Otoccipital | Paraoccipital process diverges from each other | 115 |
|  |  |  |
| Frontal | Maximum mediolateral width | 60.7 |
|  | Maximum anteroposterior length | ~38.2 |
|  |  |  |
| Hyoid | Proximodistal length | 54.95 |
|  |  |  |
| Jugal - left | Dorsoventral height under orbit | 10.43 |
|  | Anteroposterior length | ~51.86 |
|  |  |  |
| Jugal - right | Anteroposterior length | ~70.88 |
|  |  |  |
| Maxilla - right | Anteroposterior length | 111.88 |
|  |  |  |
| Orbitosphenoid - left | Anteroposterior length | 16.28 |
|  | Dorsoventral height | 13.46 |
|  |  |  |
| Parietal | Maximum anteroposterior length | 40.86 |
|  | Maximum mediolateral width | 67.96 |
|  | Minimum mediolateral width | 27.55 |
|  | Angle between squamosal rami in dorsal view | 116 |
|  |  |  |
| Postorbital - left | Jugal process, base anteroposterior length | 13.19 |
|  | Jugal ramus, dorsoventral height | 40.85 |
|  | Squamosal ramus, anteroposterior length | 25.56 |
|  | Frontal ramus, anteroposterior length | 23.55 |
|  |  |  |
| Pterygoid - left | Anteroposterior length | 100.23 |
|  | Mediolateral width of main body | 28 |
|  |  |  |
| Pterygoid - right | Anteroposterior length | 123.25 |
|  | Mediolateral width of main body | 28.61 |
|  |  |  |
| Quadrate - right | Angle between quadratojugal and pterygoid rami | 95 |
|  | Maximum mediolateral width of the condylar region | 16.79 |
|  | Maximum dorsoventral height | 53.26 |
|  | Pterygoid ramus, dorsoventral height | 36.11 |
|  | Pterygoid ramus, anteroposterior length | 19.52 |
|  |  |  |
| Squamosal | Quadratojugal ramus base anteroposterior length | 12.1 |
|  | Quadratojugal ramus dorsoventral height | 28.45 |
|  | Angle between parietal ramus and postorbital ramus | 70 |
|  |  |  |
| Stapes - right | Proximodistal length | 31 |
|  |  |  |
| Supraoccipital | Mediolateral width in posterior view | 34 |
|  | Dorsoventral height in posterior view | 21.16 |
|  | Anteroposterior length | 25.49 |
|  |  |  |
| Proatlas | Length along long axis | 31.01 |
|  | Length along shortest axis | 13.62 |
|  |  |  |
| Cervical 4 | Anteroposterior length | 94 |
|  | Maximum dorsoventral height | 64 |
|  | Centrum, anterior: dorsoventral length | 35 |
|  | Centrum, anterior: mediolateral length | 36 |
|  | Centrum, posterior: dorsoventral length | 34 |
|  | Centrum, posterior: mediolateral length | 38 |
|  | Centrum, anteroposterior length | 91 |
|  | Neural spine, dorsoventral length | 11 |
|  | Neural spine, anteroposterior length | 44 |
|  |  |  |
| Cervical 6 | Anteroposterior length | 86 |
|  | Maximum dorsoventral height | 70 |
|  | Centrum, anterior: dorsoventral length | 36 |
|  | Centrum, anterior: mediolateral length | 40 |
|  | Centrum, posterior: dorsoventral length | 43 |
|  | Centrum, posterior: mediolateral length | 40 |
|  | Centrum, anteroposterior length | 82 |
|  | Neural spine, dorsoventral length | 20 |
|  | Neural spine, anteroposterior length | 48 |
|  |  |  |
| Cervical 7 | Anteroposterior length | 80 |
|  | Maximum dorsoventral height | 66 |
|  | Centrum, anterior: dorsoventral length | 40 |
|  | Centrum, anterior: mediolateral length | 43 |
|  | Centrum, posterior: dorsoventral length | 43 |
|  | Centrum, posterior: mediolateral length | 48 |
|  | Centrum, anteroposterior length | 77 |
|  |  |  |
| Cervical 9 | Anteroposterior length | 90 |
|  | Centrum, anterior: dorsoventral length | 42 |
|  | Centrum, anterior: mediolateral length | 46 |
|  | Centrum, posterior: dorsoventral length | 45 |
|  | Centrum, posterior: mediolateral length | 50 |
|  | Centrum, anteroposterior length | 75 |
|  | Neural spine, anteroposterior length | 32 |
|  |  |  |
| Cervicodorsal | Centrum, anterior: dorsoventral length | 50 |
|  | Centrum, anterior: mediolateral length | 44 |
|  | Centrum, posterior: dorsoventral length | 47 |
|  | Centrum, posterior: mediolateral length | 54 |
|  | Centrum, anteroposterior length | 71 |
|  |  |  |
| Anterior-to-mid dorsal | Centrum, anterior: dorsoventral length | 52 |
|  | Centrum, anterior: mediolateral length | 45 |
|  | Centrum, posterior: dorsoventral length | 55 |
|  | Centrum, posterior: mediolateral length | 48 |
|  | Centrum, anteroposterior length | 68 |
|  |  |  |
| Sacral 1 | Centrum, anterior: dorsoventral length | 78 |
|  | Centrum, anterior: mediolateral length | 80 |
|  | Centrum, posterior: dorsoventral length | 80 |
|  | Centrum, posterior: mediolateral length | 82 |
|  | Centrum, anteroposterior length | 74 |
|  |  |  |
| Mid-to-posterior caudal | Centrum, anterior: dorsoventral length | 50 |
|  | Centrum, anterior: mediolateral length | 37 |
|  | Centrum, posterior: dorsoventral length | 45 |
|  | Centrum, posterior: mediolateral length | 36 |
|  | Centrum, anteroposterior length | 53 |
|  |  |  |
| Posterior caudal | Centrum, anterior: dorsoventral length | 43 |
|  | Centrum, anterior: mediolateral length | 40 |
|  | Centrum, posterior: dorsoventral length | 42 |
|  | Centrum, posterior: mediolateral length | 35 |
|  | Centrum, anteroposterior length | 58 |
|  |  |  |
| Ulna | Proximal, anteroposterior length | 61 |
|  | Proximal, mediolateral length | 32 |
|  | Proximal, circumference | 130 |
|  |  |  |
| Manual phalanx | Proximodistal length | 40 |
|  | Proximal, mediolateral width | 27 |
|  | Proximal, dorsoventral height | 24 |
|  | Distal, mediolateral width | 27 |
|  | Distal, dorsoventral height | 19 |
|  |  |  |
| Manual ungual | Proximodistal length | 27 |
|  | Proximal, mediolateral width | 10 |
|  | Proximal, dorsoventral height | 17 |
|  |  |  |
| Femur | Proximal, circumference | 198 |
|  | Distal, circumference | 212 |
|  | Distal, anteroposterior length | 85 |
|  | Distal, mediolateral length | 113 |
|  | Medial condyle, anteroposterior length | 82 |
|  | Medial condyle, mediolateral length | 42 |
|  | Lateral condyle, anteroposterior length | 47 |
|  | Lateral condyle, mediolateral length | 45 |
|  | Tibiofibular crest, anteroposterior length | 45 |
|  | Tibiofibular crest, mediolateral length | 35 |
|  |  |  |
| Tibia | Distal, circumference | 164 |
|  | Distal, maximum anteroposterior length | 68 |
|  | Distal, mediolateral length | 87 |
|  |  |  |
| Pedal ungual | Proximodistal length | 28 |
|  | Proximal, mediolateral width | 23 |
|  | Proximal, dorsoventral height | 26 |
