# Supplementary material for: Nyana rooiwalensis: South Africa's earliest near-complete sauropodomorph dinosaur skull from the Late Triassic of the Eastern Cape Province
Source: R Soc Open Sci. 2026 Nov 11;13(11):261002. doi: 10.1098/rsos.261002 (PMC13750947; doi:10.1098/rsos.261002)
Supplement: Supplementary file 1 [file rsos.261002.zip › rsos.261002/rsos261002_si_003.docx]

Figure S3: The right semicircular canal of BP/1/8468 in (A) medial and (B) lateral views. Abbreviations: ascc, anterior semicircular canal; cc, common crus; lscc, lateral semicircular canal; pscc, posterior semicircular canal; sa, sacculus. Scale bar = 5mm. Dashed lines in (A) indicate where the diameter was taken and (B) indicates how the angles were taken with respect to ear.

Table S3: Measurements of the right semicircular canal.

| **Measurement** | **mm** |
| --- | --- |
| Diameter of crus communis | 3 |
| Diameter of anterior semicircular canal | 2.71 |
| Diameter of posterior semicircular canal | 2.63 |
|  |  |
| Angle of crus communis | 80° |
| Angle of anterior semicircular canal | 72° |
| Angle of posterior semicircular canal | 32° |
| Angle of lateral semicircular canal | ~0/180° |
