# Supplementary material for: Nyana rooiwalensis: South Africa's earliest near-complete sauropodomorph dinosaur skull from the Late Triassic of the Eastern Cape Province
Source: R Soc Open Sci. 2026 Nov 11;13(11):261002. doi: 10.1098/rsos.261002 (PMC13750947; doi:10.1098/rsos.261002)
Supplement: Supplementary file 1 [file rsos.261002.zip › rsos.261002/rsos261002_si_004.docx]

Figure S4.1: Location (blue) for where measurements were taken of the postorbital. Direction (black) of how the angles were taken. (A) lateral, (B) posterior, and (C) dorsal views. Scale bar = 10 mm.

When measuring the angles of the postorbital, they were taken in an anti-clockwise direction starting with the squamosal ramus to the frontal ramus and then jugal ramus. Lengths were taken perpendicular to the axis of direction.

Table S4.1: Postorbital measurements of comparative taxa, their ratios, and the number of maxillary alveoli. Abbreviations: Frr, frontal ramus; Jr, jugal ramus; Sqr, squamosal ramus; ap, anteroposterior; dv, dorsoventral; ml, mediolateral.

|  | **Specimen #** | **Maxillary alveoli** | **Frr (ml/dv)** | **Sqr (ml/dv)** | **Jr (ml/ap)** |
| --- | --- | --- | --- | --- | --- |
| *Nyana rooiwalensis* | BP/1/8468 | 23 | 1.86 | 1.03 | 0.89 |
| *Adeopapposaurus* | PVSJ 610 | 15 | 1.10 | 0.51 | - |
| *Coloradisaurus* | PVL 3967 | 22 | 1.67 | 0.98 | - |
| *Jingshanosaurus* | LFGT-ZLJ0113 | 16 | - | - | - |
| *Lufengosaurus* | IVPP V15 | 20 | 1.95 | 0.80 | - |
| *Massospondylus carinatus* | BP/1/5241 | 17 | 1.48 | 0.51 | 0.73 |
| NMQR 3314 | NMQR 3314 | 19 | 1.22 | 0.84 | - |
| *Plateosaurus* | AMNH FARB | 25 | 2.40 | 0.69 | 0.78 |
| *Riojasaurus* | ULR 56 | 24 | 1.75 | 0.49 | - |
| *Sarahsaurus* | MCZ 8893 | 16 | 1.82 | 0.59 | 0.70 |
| *Yunnanosaurus* | NGMJ 004546 | 15 | - | 0.54 | - |
| *Mbiresaurus* | NHMZ2222 | 20 | - | - | - |
| *Kholumolumo* (left) | MNHN.F.LES397 | - | - | - | - |
| *Kholumolumo* (right) | MNHN.F.LES397 | - | - | - | - |
| *Ngwevu* | BP/1/4779 | 18 | 1.50 | 0.63 | 0.38 |
| *M. kaalae* | SAM-PK-K1325 | 14 | - | - | - |
| *Xixiposaurus* | ZLJ0108 | 20 | 1.82 | 0.38 | - |

|  | **Sqr angle** | **Frr angle** | **Jr angle** |  | **frr, dv** | **frr, ml** | **sqr, dv** | **sqr, ml** | **jr, ap** | **jr, ml** |
| --- | --- | --- | --- | --- | --- | --- | --- | --- | --- | --- |
| *Nyana rooiwalensis* | 16 | 153 | 272 |  | 9.9 | 18.4 | 7 | 7.2 | 11 | 9.8 |
| *Adeopapposaurus* | 350 | 123 | 253 |  | 6.1 | 6.7 | 6.8 | 3.5 | 6.7 | - |
| *Coloradisaurus* | 14.5 | 135 | 273 |  | 9.9 | 16.5 | 8.6 | 8.4 | 9.9 | - |
| *Jingshanosaurus* | - | - | - |  | - | - | - | - | - | - |
| *Lufengosaurus* | 19 | 133 | 275 |  | 13.4 | 26.1 | 16.1 | 12.8 | 17.9 | - |
| *Massospondylus carinatus* | 7 | 108 | 243 |  | 8.4 | 12.4 | 7 | 3.6 | 7.8 | 5.7 |
| NMQR 3314 | 17.5 | 149 | 283 |  | 18.5 | 22.5 | 17.5 | 14.7 | 16.2 | - |
| *Plateosaurus* | 3 | 135 | 268 |  | 11.7 | 28.1 | 9.8 | 6.8 | 10.7 | 8.30 |
| *Riojasaurus* | 28.6 | 145 | 274 |  | 10.2 | 17.9 | 15.05 | 7.4 | 12.5 | - |
| *Sarahsaurus* | 2.5 | 147 | 266 |  | 9.4 | 17.09 | 9.3 | 5.5 | 10 | 7 |
| *Yunnanosaurus* | 2 | 122 | 257 |  | 12.2 | - | 11.8 | 6.4 | 9.4 | - |
| *Mbiresaurus* | 20 | 138 | 278 |  | 4.5 | - | 3.6 | - | 4.9 | - |
| *Kholumolumo* (left) | 27 | 147 | 282 |  | 16.9 | - |  | - | 17.6 | - |
| *Kholumolumo* (right) | 6 | 132 | 260 |  | 17.3 | - | 11.4 | - | 15.8 | - |
| *Ngwevu* | 6 | 136 | 273 |  | 7 | 10.5 | 7 | 4.4 | 10 | 3.75 |
| *M. kaalae* | - | - | - |  | - | - | - | - | - | - |
| *Xixiposaurus* | 16 | 130 | 250 |  | 10.6 | 19.3 | 16.3 | 6.2 | 10.9 | - |

Fig. S4.2: Simplified comparison illustrating the angles of the postorbital. Blue, squamosal ramus; Red, frontal ramus; Green, jugal ramus.

Table S4.2: Information of the taxa included in the silhouette figures throughout.

| **Taxa** | **Publication** | **Specimen #** | **Period** | **Stage** | **Country** | **Formation** |
| --- | --- | --- | --- | --- | --- | --- |
| *Nyana* *rooiwalensis* | Current | BP/1/8468 |  | Rhaetian | South Africa | Elliot Formation |
| *Aardonyx celestae* | Yates 2010 |  | Jurassic | Hettangian-Pliensbachian | South Africa | Elliot Formation |
| *Kholumolumo ellenbergerorum* | de Fabregues and Allain 2020 | MNHN.F.LES153 | Triassic | Norian-Rhaetian | Lesotho | Elliot Formation |
| *Massospondylus carinatus* | Reisz et al 2010 | BP/1/5347 | Jurassic | Hettangian-Pliensbachian | South Africa | Elliot Formation |
| *Massospondylus carinatus* | Gow et al 1990 | BP/1/4376 | Jurassic | Hettangian-Pliensbachian | South Africa | Elliot Formation |
| *Massospondylus carinatus* |  | BP/1/4998 | Jurassic | Hettangian-Pliensbachian | South Africa | Elliot Formation |
| *Massospondylus carinatus* | Chapelle and Choiniere 2018 | BP/1/5241 | Jurassic | Hettangian-Pliensbachian | South Africa | Elliot Formation |
| *Massospondylus carinatus* | Barrett et al 2019 | BP/1/4934 | Jurassic | Hettangian-Pliensbachian | South Africa | Elliot Formation |
| *Massospondylus kaalae* | Barrett et al 2009 | SAM-PK-K1325 | Jurassic | Hettangian-Toarcian | South Africa | Elliot Formation |
| *Mbiresaurus raathi* | Griffin et al 2022 | NHMZ 2222 | Triassic | Carnian | Zimbabwe | Pebbly Arkose Formation |
| *Mussaurus patagonicus* | Pol et al 2021 | MACN-PV 4111 | Jurassic | Sinemurian | Argentina | Laguna Colorada Formation |
| *Mussaurus patagonicus* | Otero et al 2019 | PVL 4587 | Jurassic | Sinemurian | Argentina |  |
| *Mussaurus patagonicus* | Otero and Novas 2022 | MPM-PV 1868 | Jurassic | Sinemurian | Argentina | Laguna Colorada Formation |
| *Ngwevu intloko* | Chapelle et al 2019 | BP/1/4779 | Jurassic | Pliensbachian | South Africa | Elliot Formation |
| NMQR 3314 | Yates 2007 | NMQR 3314 | Jurassic | Hettangian-Pliensbachian | South Africa | Elliot Formation |

Table S4.3: Information on the specimens used in the ontogenetic range.

| **Taxa** | **Specimen #** | **Ontogenetic age** | **Sources for age** | **Reasons** |
| --- | --- | --- | --- | --- |
| *Massospondylus carinatus* | BP/1/5347 | Embryonic | Reisz et al 2010; Chapelle et al 2021 | Preserved within an egg. Part of an egg cluster. No LAGs observed. All sutures in cranial and postcrania are unfused. |
| *Massospondylus carinatus* | BP/1/4376 | One year old | Gow et al 1990; Chapelle et al 2022 | Most cranial sutures unfused. Plexiform and laminar vascular arrangement. WPC. 1 annulus. |
| *Massospondylus carinatus* | BP/1/4998 | Juvenile | Chapelle et al 2021, 2022 | 5 LAGs. Decreased LAG spacing towards periphery. WPC. Lack resorption cavities. Laminar and longitudinal canals in outer cortex. Lamellar bone. No EFS. |
| *Massospondylus carinatus* | BP/1/5241 | subadult | Chapelle and Choiniere 2018; Chapelle et al 2021, 2022 | Lamellar bone in outer cortex. Longitudinally-oriented primary osteons at the sub-periosteal surface. Have double and triple LAGs. Laminar vascular arrangement in inner cortex. 9 LAGs. No EFS. |
| *Massospondylus carinatus* | BP/1/4934 | Adult | Barrett et al 2019 | Rapidly forming well-vascularized fibrolamellar bone, with a transition to slower forming parallel-fibred bone towards the sub-periosteal surface. Decrease in spacing between LAGs. No EFS. Secondary remodelling. Double and triple LAGs. HOS 12. 10 LAGs. |
| *Mussaurus patagonicus* | MACN-PV 4111 | Hatchling | Pol et al 2021 | Cranial bones unfused. Long bones unfused. |
| *Mussaurus patagonicus* | PVL 4587 | One year old | Otero et al 2019; Cerda et al 2014 | Laminar or plexiform vascularization. No GM. Woven-fibred bone in cortex. |
| *Mussaurus patagonicus* | MPM-PV 1868 | Adult | Otero and Novas 2022; Pol et al 2021; | Cranial sutures fused. |
